# A General Acid‐Mediated Hydroaminomethylation of Unactivated Alkenes and Alkynes

**DOI:** 10.1002/anie.201906910

**Published:** 2019-09-04

**Authors:** Daniel Kaiser, Veronica Tona, Carlos R. Gonçalves, Saad Shaaban, Alberto Oppedisano, Nuno Maulide

**Affiliations:** ^1^ University of Vienna Institute of Organic Chemistry Währinger Strasse 38 1090 Vienna Austria

**Keywords:** amines, hydroaminoalkylation, hydroaminomethylation

## Abstract

In comparison to the extensively studied metal‐catalyzed hydroamination reaction, hydroaminomethylation has received significantly less attention despite its considerable potential to streamline amine synthesis. State‐of‐the‐art protocols for hydroaminomethylation of alkenes rely largely on transition‐metal catalysis, enabling this transformation only under highly designed and controlled conditions. Here we report a broadly applicable, acid‐mediated approach to the hydroaminomethylation of unactivated alkenes and alkynes. This methodology employs cheap, readily available, and bench‐stable reactants and affords the desired amines with excellent functional group tolerance and impeccable regioselectivity. The broad scope of this transformation, as well as mechanistic investigations and in situ domino functionalization reactions are reported.

## Graphical abstract

**Amines on the cheap**: In comparison to the hydroamination reaction, hydroaminomethylation, the addition of a hydrogen and a (methyleneamino) unit across an olefin, has received significantly less attention. A broadly applicable, acid‐mediated strategy for the hydroaminomethylation of unactivated alkenes and alkynes has been developed using cheap and bench‐stable reactants.

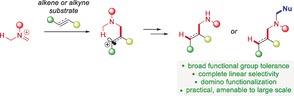

Amines are key structural moieties in pharmaceuticals, agrochemicals, natural products, and materials. Probably the most powerful approach for the formation of C(sp^3^)−N bonds, apart from classical S_N_2 reactions, is the direct addition of nitrogen‐containing fragments to olefins.^1^ While, in this regard, the direct metal‐catalyzed hydroamination of alkenes has been extensively studied,^2^ hydroaminomethylation, the addition of a hydrogen and a (methyleneamino) unit across an olefin, is often overlooked.^3^ Traditional methods to effect net hydroaminomethylation involve the two‐step sequence of alkene hydroformylation followed by reductive amination (Scheme 1, A).^4^ More recently, several metal‐catalyzed direct hydroaminoalkylation reactions of alkenes have been reported that employ simple alkylamines as the starting materials (Scheme 1, B).^5^ These approaches, dominated by early transition‐metal catalysts, have shown some restrictions, in part as a consequence of the thermodynamically challenging C−H activation step α to secondary amines and of the traditional incompatibility of early transition metals with oxygen functional groups (with only few notable exceptions5a, 5b). Attempts to move towards late‐transition‐metal catalysis typically rely on the use of directing groups.^6^ An elegant exception to this requirement is the ruthenium‐catalyzed hydroaminoalkylation of dienes and allenes developed by Krische and co‐workers (not shown).^7^ Recent studies have harnessed photoredox catalysis,^8^ or dual catalysis^9^ (Scheme 1, C): However, while displaying impressive functional group tolerance and mild reaction conditions, such approaches remain limited to conjugated olefins or otherwise electronically activated alkenes.^7^, ^8^

**Scheme 1:**
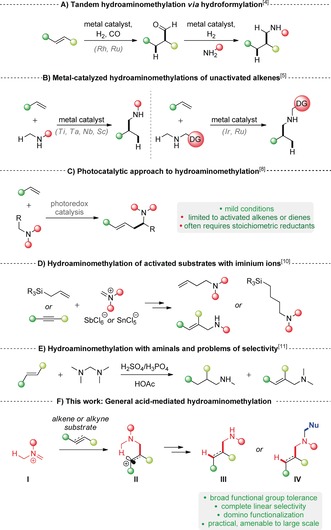
Previous approaches to hydroaminomethylation and development of a metal‐free general hydroaminomethylation of alkenes and alkynes.

Challenges commonly associated with metal‐catalyzed hydroaminomethylation range from the control of regioselectivity and the threat of double functionalization to the development of conditions that allow the functionalization of alkynes. Seeking to address these challenges, we became interested in the development of a hydroaminomethylation protocol using iminium ions. This transformation was envisioned to proceed by an entirely different mechanism, thus circumventing the need for transition‐metal catalysis. Mayr and co‐workers have shown that iminium ions paired with noncoordinating anions can be employed for hydroaminomethylation of highly activated π‐systems (Scheme 1, D).^10^ Cohen and Onopchenko investigated the in situ formation of iminium ions by treating bis(dimethylamino)methane with a Brønsted acid (Scheme 1, E).^11^ The reaction of simple alkenes under these conditions resulted in a mixture of aliphatic secondary and allylic tertiary amines formed through competing hydride transfer and ene reaction.^12^, ^13^, ^14^ Similar reactivity was observed serendipitously during Heathcock and co‐workers’ synthesis of methyl homosecodaphniphyllate,^15^ as well as by Manninen and co‐workers, who reported reactions of iminium ions with norbornene derivatives, obtaining mixtures of products in most cases.^16^ Hoping to develop a more general approach, we designed a system able to suppress competing elimination which would result in undesired alkene formation. In our synthetic plan, the nucleophilic addition of a π‐system onto an iminium ion (**I**) would trigger an internal redox event by 1,5‐hydride transfer to the resulting carbenium ion of intermediate **II**, affording the desired hydroaminomethylated products (**III**) or products of direct functionalization with nucleophiles (**IV**) (Scheme 1, F).^17^

At the outset, we hypothesized that the use of Eschenmoser's salt (*N*,*N*‐dimethylmethylideneammonium iodide) or derivatives thereof would be amenable to realizing the plan outlined in Scheme 1. While we were pleased to find that the treatment of styrene with Eschenmoser's salt led to the formation of amine **3 m** in up to quantitative yield (Method A), we soon became aware of limitations in the reaction scope accessible with this class of reagents (see Scheme 2 and the Supporting Information for further information).

**Scheme 2:**
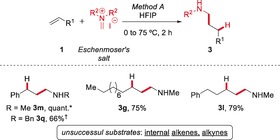
Eschenmoser's salt enabled initial success. Reactions were run on 0.5 mmol scale. Yields refer to isolated material after purification. *: Yield determined by ^1^H NMR analysis using an internal standard. †: Eschenmoser's chloride was employed. HFIP=1,1,1,3,3,3‐hexafluoro‐2‐propanol.

We therefore turned to alternative iminium ion precursors such as the commercially available aminal bis(dimethylamino)methane (**2 a**), derivatives of which are common reagents in transition‐metal catalysis.7a, 7c, 18 When trifluoroacetic acid (TFA) was employed at slightly elevated temperatures (see the Supporting Information for details on optimization), the desired secondary amine product was isolated in high yield after aqueous, hydrolytic workup (Scheme 3, Method B). While the precise reasons for the increased selectivity observed when using this solvent remain unclear, we suspect the solvating properties of TFA, as well as the low nucleophilicity and low basicity of the corresponding conjugate base, to play important roles in dictating the reaction outcome by facilitating the hydride transfer event.^19^ Using these conditions, a wide range of aminals and alkenes were found to be suitable reactants in this novel hydroaminomethylation protocol (Scheme 3). Initially focusing on substitution at nitrogen, we observed the formation of a range of secondary amines in high yields (**3 a**–**f**, 73–92 % yield) starting from several readily available aminals. Particularly appealing is the possibility of obtaining benzylated or allylated amines (**3 e**, **3 f**), ripe for easy deprotection and downstream functionalization (**4 f**). The formation of **3 e** is complementary to transition‐metal catalysis, where the use of benzylamines tends to lead to branched products resulting from activation of the benzylic C−H bond,5b, 5e and which thus relies most commonly on less flexible *N*‐aryl substrates (and products). With respect to the olefinic substrates, a wide variety of substitution patterns were accommodated by our protocol, including unactivated linear, branched, and cyclic alkenes (**3 g**–**l**, 36–91 % yield). It is worth mentioning that all linear and cyclic alkenes provided only a single isolable product, while the reaction of a branched alkene showed the occurrence of an ene‐reaction‐derived side product, leading to a diminished yield of **3 h**. Styrenes also proved to be viable partners for this transformation, providing the products of hydroaminomethylation in good to excellent yields (**3 m**–**u**, 39–86 % yield, up to 50 mmol scale). As shown by **3 t**, the effect of the double‐bond geometry is negligible, as the amine was isolated in good yield starting from either stereoisomer. More telling, however, are the results of a functional‐group tolerance study: an unusually broad range of polar moieties is tolerated by this protocol, including esters, phosphonates, amides, nitriles and halides (**3 v**–**3 aa**, 40–93 % yield). Remarkably, an alkene containing a free alcohol also proved to be a viable substrate for this transformation, affording the corresponding product in 82 % yield (**3 ab**). This stands in strong contrast with the early‐transition‐metal‐catalyzed procedures that are state of the art in hydroaminomethylation: the oxophilicity of the catalysts employed is difficult to reconcile with a free alcohol. Even more striking is the successful hydroaminomethylation of amine‐containing substrates under our conditions (**3 ac** and **3 ad**, 60 % and 75 % yield), as free amine functionalities generally lie beyond the scope of state‐of‐the‐art direct hydroaminomethylation through metal‐ and photoredox‐catalytic procedures.^8^, ^9^ Furthermore, it is worth noting that complete selectivity for the linear (vs. branched) products is observed throughout. Having explored the performance of this approach with alkenes, we became interested in the possibility of achieving the hydroaminomethylation of alkynes. Pleasingly, our protocol enables this reaction with no trace of double hydroaminomethylation. In this context, we were delighted to find that terminal alkynes afforded the secondary allylic amines **6 a** and **6 b** in good yields exclusively as the *E*‐stereoisomers, as a consequence of the *syn*‐nature of the hydride transfer. The reaction proceeded selectively even in the presence of an additional alkene (**6 c**, 68 % yield) or a cyclopropane moiety (**6 d**, 85 % yield). Importantly, owing to the formation of the most stable carbocationic intermediate, the use of internal alkynes leads to single isomeric trisubstituted alkene products (**6 e** and **6 j**, 41 % and 48 % yield). As before, aryl substitution (**6 f**–**j**, 31–57 % yield) and a range of functional groups were tolerated (**6 k**–**m**, 41–97 % yield), including a propargyl silane (**6 n**). These results establish our procedure for hydroaminomethylation as a simple synthesis of stereodefined di‐ and trisubstituted allylic amines.

**Scheme 3:**
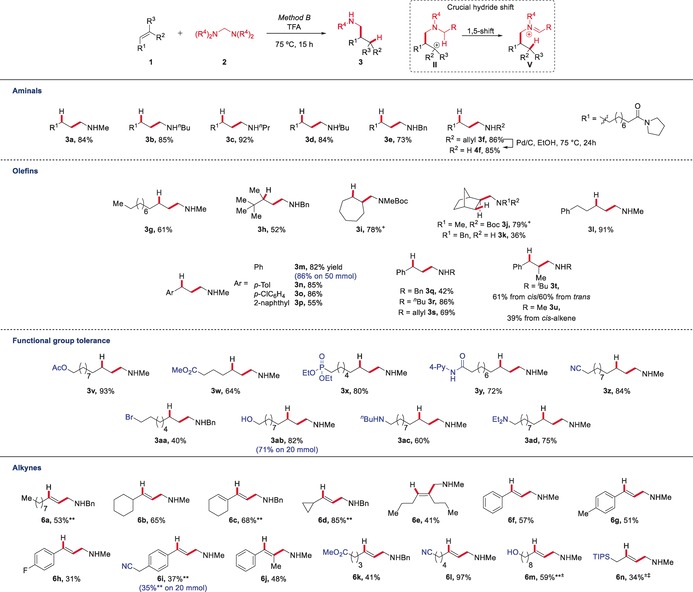
Reaction development and scope of the hydroaminomethylation reaction. Reactions were run on 0.5 mmol scale. Yields refer to isolated material after purification. ^+^: Yield after acylative protection with Boc_2_O to facilitate isolation. **: DCE used as co‐solvent. ^±^: Reaction was run for 5 h. ^≠^: Reaction was run at room temperature. See Supporting Information for details.

The iminium ion **V,** ultimately resulting from the pivotal 1,5‐hydride shift, can be easily engaged in domino functionalization processes (Scheme 4). The addition of simple ketones to the acidic reaction mixture results in the formation of trisubstituted amines in good yields via Mannich reaction (**7 a**–**g**, 52–71 % yield), whereas capture with an electron‐rich arene via Friedel–Crafts reaction delivers a tertiary benzylic amine product (**7 h**, 54 % yield). Furthermore, employing Method A, we were able to perform domino hydroaminomethylation–functionalization reactions using hydridic reductants (**7 i**, 74 % yield) and organozinc nucleophiles (**7 j** and **7 k**, 66 % and 63 % yield, respectively) that are incompatible with more acidic conditions.

**Scheme 4:**
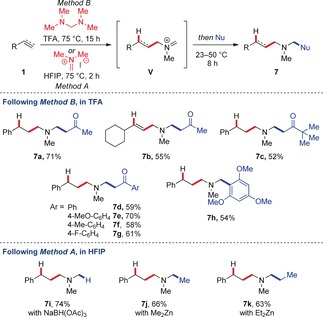
Domino C−C and C−H bond formation. Reactions were run on 0.2 mmol scale. Yields refer to isolated material after purification.

The presented method also enables a straightforward and unusual disconnection towards bioactive amines such as the broad‐spectrum antimycotic Naftifine (**8**) (Scheme 5),^20^ accessible in a simple two‐step sequence from phenylacetylene.

**Scheme 5:**
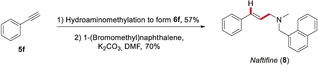
Concise synthesis of Naftifine (**8**).

Additional observations provide support for the mechanism proposed above and showcase interesting features of the system at hand (Scheme 6). As expected, the use of perdeuterated bis(dimethylamino)methane (**2 a**‐**d_12_**) led to regioselective and complete deuterium incorporation, confirming an intramolecular hydride transfer event (**9 a**, Scheme 6, A). Additionally, treatment of both styrene (**1 m**) and aliphatic olefin **1 a** with the isotopically mixed aminal (**2 b**‐**d_18_**) showcases a kinetic isotope effect (*K*_H_/*K*_D_), shown to be slightly higher for styrene (Scheme 6, B). This result suggests that, while the hydride transfer event is not rate‐determining, it has a higher relative contribution to the reaction rate in the case of the more electron‐rich, and therefore more reactive, styrene substrate.^21^ Moreover, it is likely that the relative increase in stabilization of the benzylic cation contributes to the higher *K*_H_/*K*_D_ observed for styrene.

**Scheme 6:**
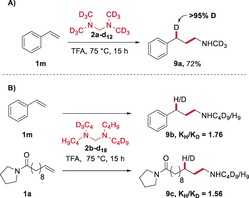
Mechanistic considerations.

In conclusion, we have developed an acid‐mediated hydroaminomethylation of unactivated alkenes and alkynes. The methodology displays an unconventionally broad functional group tolerance and allows the synthesis of secondary and, after nucleophilic quenching, tertiary amines. Given the natural abundance of both alkenes and alkynes, we anticipate that this practical, cheap, and direct amination will find broad application in synthetic chemistry and feedstock‐chemical processing alike.

## Conflict of interest

N.M. and co‐workers own a patent on the hydroaminomethylation process (Pct/EP2019/062867).

## Supporting information

As a service to our authors and readers, this journal provides supporting information supplied by the authors. Such materials are peer reviewed and may be re‐organized for online delivery, but are not copy‐edited or typeset. Technical support issues arising from supporting information (other than missing files) should be addressed to the authors.

Supplementary

## References

[1a] J. Bariwal , E. Van der Eycken , Chem. Soc. Rev. 2013, 42, 9283–9303; 24077333 10.1039/c3cs60228a

[1b] P. Ruiz-Castillo , S. L. Buchwald , Chem. Rev. 2016, 116, 12564–12649. 27689804 10.1021/acs.chemrev.6b00512 PMC5070552

[2] For reviews on hydroamination, see

[2a] C. Michon , M.-A. Abadie , F. Medina , F. Agbossou-Niedercorn , J. Organomet. Chem. 2017, 847, 13–127;

[2b] C. Leporia , J. Hannedouche , Synthesis 2017, 49, 1158–1167;

[2c] V. Isaeva , L. M. Kustov , Top Catal. 2016, 59, 1196–1206;

[2d] M. Patel , R. K. Saunthwal , A. K. Verma , Acc. Chem. Res. 2017, 50, 240–254; 28128923 10.1021/acs.accounts.6b00449

[2e] B. J. Li , C. El-Nachef , A. E. Beauchemin , Chem. Commun. 2017, 53, 13192–13204; 10.1039/c7cc07352f 29131221

[2f] L. Huang , M. Arndt , K. Gooßen , H. Heydt , L. Gooßen , Chem. Rev. 2015, 115, 2596–2697; 25721762 10.1021/cr300389u

[2g] A. L. Reznichenko , A. J. Nawara-Hultzsch , K. C. Hultzsch , Top. Curr. Chem. 2013, 343, 191–260; 10.1007/128_2013_500 24276959

[2h] A. Reznichenko , K. C. Hultzsch , Top. Organomet. Chem. 2011, 43, 51–114;

[2i] K. D. Hesp , M. Stradiotto , ChemCatChem 2010, 2, 1192–1207;

[2j] T. E. Müller , K. C. Hultzsch , M. Yus , F. Foubelo , M. Tada , Chem. Rev. 2008, 108, 3795–3892. 18729420 10.1021/cr0306788

[3a] P. M. Edwards , L. L. Schafer , Chem. Commun. 2018, 54, 12543–12560; 10.1039/c8cc06445h 30334025

[3b] E. Chong , P. Garcia , L. L. Schafer , Synthesis 2014, 46, 2884–2896;

[3c] P. W. Roesky , Angew. Chem. Int. Ed. 2009, 48, 4892–4894; 10.1002/anie.200900735 19437521

[4] For reviews on metal-catalyzed hydroamination via hydroformylation, see

[4a] P. Kalck , M. Urrutigoïty , Chem. Rev. 2018, 118, 3833–3861; 29493233 10.1021/acs.chemrev.7b00667

[4b] C. Chen , X.-Q. Dong , X. Zhang , Org. Chem. Front. 2016, 3, 1359–1370;

[4c] D. Crozet , M. Urrutigoïty , P. Kalck , ChemCatChem 2011, 3, 1102–1118;

[4d] M. Beller , J. Seayad , A. Tillack , H. Jiao , Angew. Chem. Int. Ed. 2004, 43, 3368–3398; 10.1002/anie.200300616 15221826

[4e] P. Eilbracht , L. Bärfacker , C. Buss , C. Hollmann , B. E. Kitsos-Rzychon , C. L. Kranemann , T. Rische , R. Roggenbuck , A. Schmidt , Chem. Rev. 1999, 99, 3329–3366; For recent examples, see:11749518 10.1021/cr970413r

[4f] S. Hanna , J. C. Holder , J. F. Hartwig , Angew. Chem. Int. Ed. 2019, 58, 3368–3372; 10.1002/anie.201811297 PMC6548469 30635956

[4g] H. Liu , D. Yang , D.-L. Wang , P. Wang , Y. Lu , V.-T. Giang , Y. Liu , Chem. Commun. 2018, 54, 7979–7982; 10.1039/c8cc03431a 29963658

[4h] T. A. Faßbach , F. O. Sommer , A. J. Vorholt , Adv. Synth. Catal. 2018, 360, 1473–1482;

[4i] C. Chen , S. Jin , Z. Zhang , B. Wei , H. Wang , K. Zhang , H. Lv , X.-Q. Dong , X. Zhang , J. Am. Chem. Soc. 2016, 138, 9017–9020. 27385399 10.1021/jacs.6b03596

[5] For recent examples of early-transition-metal-catalyzed direct hydroaminomethylation, see:

[5a] R. C. DiPucchio , S.-C. Rosca , L. L. Schafer , Angew. Chem. Int. Ed. 2018, 57, 3469–3472; 10.1002/anie.201712668 29330909

[5b] J. M. Lauzon , P. Eisenberger , S.-C. Roşca , L. L. Schafer , ACS Catal. 2017, 7, 5921–5931;

[5c] J. Bielefeld , S. Doye , Angew. Chem. Int. Ed. 2017, 56, 15155–15158; 10.1002/anie.201708959 28994176

[5d] F. Liu , G. Luo , Z. Hou , Y. Luo , Organometallics 2017, 36, 1557–1565;

[5e] L. H. Lühning , J. Strehl , M. Schmidtmann , S. Doye , Chem. Eur. J. 2017, 23, 4197–4202; 28124797 10.1002/chem.201605923

[5f] E. Chong , J. W. Brandt , L. L. Schafer , J. Am. Chem. Soc. 2014, 136, 10898–10901; 25041474 10.1021/ja506187m

[5g] A. L. Reznichenko , K. C. Hultzsch , J. Am. Chem. Soc. 2012, 134, 3300–3311; 22264172 10.1021/ja211945m

[5h] S. B. Herzon , J. F. Hartwig , J. Am. Chem. Soc. 2008, 130, 14940–14941. 18937477 10.1021/ja806367e PMC4852863

[6] For recent examples of late-transition-metal-catalyzed direct hydroaminomethylation, see:

[6a] A. T. Tran , J.-Q. Yu , Angew. Chem. Int. Ed. 2017, 56, 10530–10534; 10.1002/anie.201704755 PMC5561469 28620981

[6b] H. Jiang , J. He , T. Liu , J.-Q. Yu , J. Am. Chem. Soc. 2016, 138, 2055–2059; 26796676 10.1021/jacs.5b13462 PMC4817546

[6c] M. Schinkel , L. Wang , K. Bielefeld , L. Ackermann , Org. Lett. 2014, 16, 1876–1879. 24635222 10.1021/ol500300w

[7a] S. Oda , J. Franke , M. J. Krische , Chem. Sci. 2016, 7, 136–141; 29861974 10.1039/c5sc03854e PMC5950558

[7b] T.-J. Chen , R. Tsutsumi , T. P. Montgomery , I. Volchkov , M. J. Krische , J. Am. Chem. Soc. 2015, 137, 1798–1801; 25642996 10.1021/ja5130258

[7c] S. Oda , B. Sam , M. J. Krische , Angew. Chem. Int. Ed. 2015, 54, 8525–8528; 10.1002/anie.201503250 26031224

[8a] A. Trowbridge , D. Reich , M. J. Gaunt , Nature 2018, 561, 522–527; 30258135 10.1038/s41586-018-0537-9

[8b] J. Ye , I. Kalvet , F. Schoenebeck , T. Rovis , Nat. Chem. 2018, 10, 1037–1041; 30061617 10.1038/s41557-018-0085-9 PMC6150826

[8c] J. Xie , J. Yu , M. Rudolph , F. Rominger , A. S. K. Hashmi , Angew. Chem. Int. Ed. 2016, 55, 9416–9421; 10.1002/anie.201602347 27351709

[8d] L. Chu , C. Ohta , Z. Zuo , D. W. C. MacMillan , J. Am. Chem. Soc. 2014, 136, 10886–10889; 25032785 10.1021/ja505964r PMC4132975

[8e] Y. Miyake , K. Nakajima , Y. Nishibayashi , J. Am. Chem. Soc. 2012, 134, 3338–3341. 22296639 10.1021/ja211770y

[9a] J. Zheng , B. Breit , Angew. Chem. Int. Ed. 2019, 58, 3392–3397; 10.1002/anie.201813646 30620131

[9b] S. M. Thullen , T. A. Rovis , J. Am. Chem. Soc. 2017, 139, 15504–15508. 29048886 10.1021/jacs.7b09252

[10a] S. Rehn , A. R. Ofial , H. Mayr , Synthesis 2003, 12, 1790–1796;

[10b] A. R. Ofial , H. Mayr , Liebigs Ann. 1997, 333–335;

[10c] A. R. Ofial , H. Mayr , Angew. Chem. Int. Ed. Engl. 1997, 36, 143–145;

[10d] N. Arai , T. Ohkuma , Tetrahedron 2011, 67, 1617–1622;

[10e] A. R. Ofial , H. Mayr , J. Org. Chem. 1996, 61, 5823–5830.

[11] T. Cohen , A. Onopchenko , J. Org. Chem. 1983, 48, 4531–4537.

[12] W. J. Drury III , D. Ferraris , C. Cox , B. Young , T. A. Lectka , J. Am. Chem. Soc. 1998, 120, 11006–11007.

[13] C. J. Schmidle , R. C. Mansfield , J. Am. Chem. Soc. 1955, 77, 4636–4638.

[14] The literature discriminates between two types of ene reaction in this context: While Cohen (ref. [11]) refers to the reaction of the iminium ion with the allylic system, leading to the formation of allylic amines, Mayr (ref. [10]) describes inverse electron-demand ene reactions of the alkene with the H_2_C=N−CHR unit of the iminium ion, leading to the desired products.

[15] C. H. Heathcock , Angew. Chem. Int. Ed. Engl. 1992, 31, 665–681;

[16a] H. Krieger , K. Manninen , Tetrahedron Lett. 1966, 7, 6483–6488;

[16b] K. Manninen , J. Haapala , Acta Chem. Scand. B 1974, 28, 433–440.

[17] For a review on intramolecular hydride transfer reactions, see: M. C. Haibach , D. Seidel , Angew. Chem. Int. Ed. 2014, 53, 5010–5036; 10.1002/anie.201306489 24706531

[18a] Y. Xie , J. Hu , Y. Wang , C. Xia , H. Huang , J. Am. Chem. Soc. 2012, 134, 20613–20616; 23234468 10.1021/ja310848x

[18b] J. Hu , Y. Xie , H. Huang , Angew. Chem. Int. Ed. 2014, 53, 7272–7276; 10.1002/anie.201403774 24891234

[19a] A. J. Parker , U. Mayer , R. Schmid , V. Gutmann , J. Org. Chem. 1978, 43, 1843–1854;

[19b] P. E. Peterson , J. Am. Chem. Soc. 1960, 82, 5834–5837;

[19c] P. E. Peterson , G. Allen , J. Org. Chem. 1962, 27, 1505–1509.

[20a] A. Stuetz , A. Georgopoulos , W. Granitzer , G. Petranyi , D. Berney , J. Med. Chem. 1986, 29, 112–125; 3510297 10.1021/jm00151a019

[20b] G. Petranyi , N. S. Ryder , A. Stutz , Science 1984, 224, 1239–1241. 6547247 10.1126/science.6547247

[21] Our experiments suggest that the mechanism of the reaction is strongly dependent on the substrate. For a concerted mechanism, a higher *K* _H_/*K* _D_ than that observed would be expected (ref. [12]); however, based on our experiments, we cannot exclude a concerted ene-type reaction mechanism. For more experiments, see the Supporting Information.

