# Supplementary material for: A General Acid‐Mediated Hydroaminomethylation of Unactivated Alkenes and Alkynes
Source: Angew Chem Int Ed Engl. 2019 Sep 4;58(41):14639–43. doi: 10.1002/anie.201906910 (PMC6790944; doi:10.1002/anie.201906910)
Supplement: Supplementary file 1 — Supplementary [file ANIE-58-14639-s001.pdf]

Angewandte Chemie
Eine Zeitschrift der Gesellschaft Deutscher Chemiker

Supporting Information

A General Acid-Mediated Hydroaminomethylation of Unactivated Alkenes and Alkynes

*Daniel Kaiser[+], Veronica Tona[+], Carlos R. Gonçalves[+], Saad Shaaban, Alberto Oppedisano, and Nuno Maulide**

anie_201906910_sm_miscellaneous_information.pdf

Supporting Information

Table of Contents

1 General Information 3

2 Experimental 4

2.1 Optimization Table 4

2.2 Procedures for the Synthesis of Starting Materials 5

2.2.1 General Procedure for the Synthesis of Aminals 5

2.2.2 General Procedure for the Synthesis of Eschenmoser's Salts 8

2.2.3 Synthesis of Alkene Substrates 9

2.3 General Procedures for Hydroaminomethylation with Eschenmoser's Salts (Method A) 13

2.4 General Procedures for Hydroaminomethylation with Aminals (Method B) 13

2.4.1 General Procedure A for Hydroaminomethylation of Alkenes 13

2.4.2 General Procedure B for Hydroaminomethylation of Alkynes 14

2.4.3 General Procedure C for Hydroaminomethylation of Alkynes 14

2.5 Analytical Data 16

2.6 C–C Bond Formation on the Reaction Intermediate 32

2.7 Synthesis of Naftifine 39

2.8 Mechanistic Experiments 40

2.8.1 Deuteration Experiments 40

2.8.2 Kinetic Isotope Effect Measurements 40

2.8.3 Reactions with Bis(piperidinyl)methane: Domino Hydroaminomethylation/C–H Functionalization 42

3 NMR Spectra 45

4 References 118

1 General Information

All glassware was oven dried at 100 °C before use. All solvents were distilled from appropriate drying agents prior to use. All reagents were used as received from commercial suppliers unless otherwise stated. Neat infrared spectra were recorded using a Perkin-Elmer Spectrum 100 FT-IR spectrometer. Wavenumbers ($\nu = 1/\lambda$) are reported in cm^{-1}. Mass spectra were obtained using a Finnigan MAT 8200 or (70 eV) or an Agilent 5973 (70 eV) spectrometer, using electrospray ionization (ESI). All ^{1}H NMR and ^{13}C NMR experiments were recorded using Bruker AV-400, AV-600 and AV-700 spectrometers at 300 K. Chemical shifts (δ) are quoted in ppm and coupling constants (*J*) are quoted in Hz. The 7.26 ppm resonance of residual $CHCl_3$ for proton spectra and 77.16 ppm resonance for carbon spectra were used as internal references. ^{1}H NMR splitting patterns were designated as singlet (s), doublet (d), triplet (t), quartet (q) or combinations thereof, as well as broad (br). Splitting patterns that could not be interpreted were designated as multiplet (m). Reaction progress was monitored by thin layer chromatography (TLC) performed on aluminum plates coated with kieselgel F254 with 0.2 mm thickness. Visualization was achieved by a combination of ultraviolet light (254 nm) and acidic potassium permanganate or ninhydrin. Flash column chromatography was performed using silica gel 60 (230–400 mesh, Merck and co.).

2 Experimental

2.1 Optimization Table

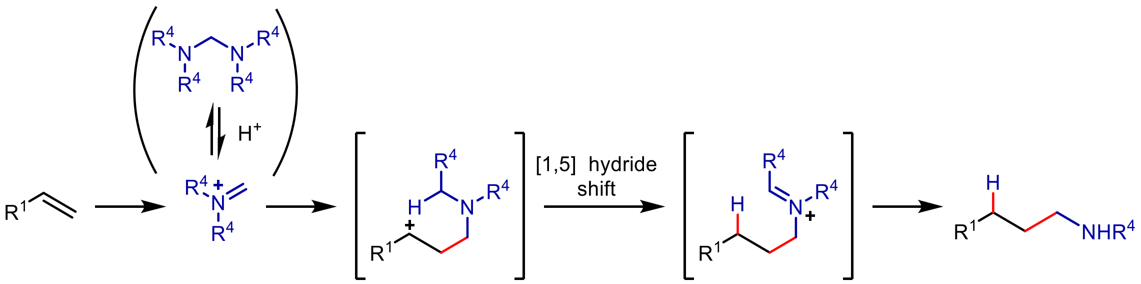

ENTRY	SUBSTRATE	N SOURCE	SOLVENT/ACID	TEMPERATURE (°C)	TIME (h)	NMR YIELD[a] (%)
**1**	Styrene	Eschenmoser's Salt	MeCN (0.2 M)	75	16	35
**2**	Styrene	Eschenmoser's Salt	AcOH (0.6 M)	75	16	33
**3**	Styrene	Eschenmoser's Salt	AcOH (0.6 M)	23	15	0
**4**	Styrene	Eschenmoser´s Iodide	HFIP (0.6 M)	75	16	100
**4**	Styrene	TMAM	AcOH (0.6 M)	75	16	40
**5**	Styrene	TMAM	Trichloroacetic acid (22.4 equiv.)	75	16	24
**6**	1-Undecene	TMAM	AcOH (0.6 M)	115	16	50
**7**	1-Undecene	TMAM	AcOH (0.6 M) (+ 1.5 equiv. TfOH)	115	16	20
**8**	**1-Undecene**	**TMAM**	**TFA (0.6 M)**	**75**	**16**	**80 (isolated)**

[a]NMR yield determined with 1,3,5-trimethoxybenzene as internal standard. Eschenmoser's Salt = Dimethylmethylideneammonium chloride; TFA = Trifluoroacetic acid; TfOH = Trifluoromethanesulfonic acid; TMAM = Tetramethyldiaminomethane

2.2 Procedures for the Synthesis of Starting Materials

2.2.1 General Procedure for the Synthesis of Aminals

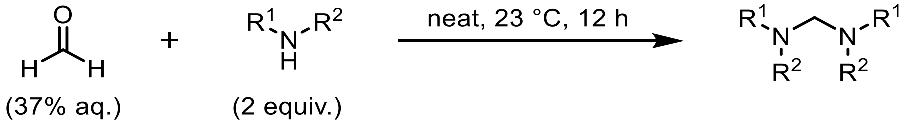

To a round-bottom flask charged with the corresponding free secondary amine[A] (2.00 equiv.) and a magnetic stir-bar at 0 °C, an aqueous solution of formaldehyde (37%, 1.00 equiv.) was added dropwise and the resulting biphasic mixture was stirred vigorously at ambient temperature (23 °C) for 12 h. The following work-up was dependent on the volatility of the resulting aminal.

Work-up **A** (for volatile products): Solid potassium hydroxide was added to the reaction mixture until saturation of the aqueous layer was observed. The phases were subsequently separated, and the aqueous phase was extracted with diethyl ether (2 ×). The organic phases were combined, dried over anhydrous potassium carbonate and filtered. The filtrate was then carefully concentrated under reduced pressure with mild heating, affording the title compound is sufficient purity for further use.

Work-up **B** (for non-volatile products): The biphasic mixture was separated, and the aqueous phase was extracted with ethyl acetate (3 ×). The organic phases were combined, dried over anhydrous sodium sulfate and concentrated under reduced pressure, in most cases affording the title compound in sufficient purity for further use. In the case of incomplete conversion, remaining unreacted amine could be removed by subjection to high vacuum.

***N,N,N',N'*-Tetrabutyldiaminomethane (2b)**

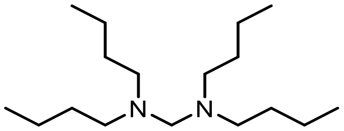

quant.; **^{1}H NMR (400 MHz, $CDCl_3$)** δ 2.99 (s, 2H), 2.43 (t, *J* = 7.5 Hz, 8H), 1.42–1.23 (m, 16H), 0.90 (t, *J* = 7.3 Hz, 12H); **^{13}C NMR (100 MHz, $CDCl_3$)** δ 75.7, 52.1 (4C), 29.5 (4C), 20.9 (4C), 14.3 (4C); **IR (neat) ν_{max}:** 2955, 2928, 2860, 2799, 2737, 1462, 1375, 1304, 1266, 1240, 1182, 1079; **HRMS (ESI+):** exact mass calculated for $[M+H]^+$ ($C_{17}H_{39}N_2$) requires *m/z* 271.3108, found *m/z* 130.1592 (corresponding to *N,N*-dibutylamine).

[A] For amines available only as the corresponding salts, 2.00 equiv. of potassium carbonate are added to the reaction mixture.

***N,N,N',N'*-Tetrapropyldiaminomethane (2c)**

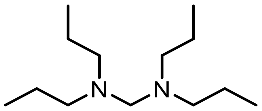

Used as a crude mixture containing residual dipropylamine. Purity 83 wt%. **^{1}H NMR (400 MHz, $CDCl_3$)** δ 3.01 (s, 2H), 2.42–2.39 (m, 8H), 1.45–1.39 (m, 8H), 0.86 (t, *J* = 7.5 Hz, 12H); **^{13}C NMR (100 MHz, $CDCl_3$)** δ 75.7, 54.4 (4C), 20.5 (4C), 12.6 (4C); **IR (neat) ν_{max}:** 2957, 2932, 2872, 2800, 1463, 1377, 1192, 1172; **HRMS (ESI+):** exact mass calculated for $[M+H]^+$ ($C_{13}H_{31}N_2$) requires *m/z* 215.2482, found *m/z* 102.1278 (corresponding to *N,N*-dipropylamine).

***N,N,N',N'*-Tetraisobutyldiaminomethane (2d)**

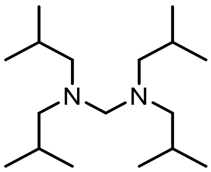

approx. 30% yield, used as a crude mixture. Purity 75 wt%. **^{1}H NMR (400 MHz, $CDCl_3$)** δ 3.26 (s, 1H), 2.13 (d, *J* = 7.2 Hz, 4H), 1.74 (dt, *J* = 13.4, 6.7 Hz, 3H), 0.87 (d, *J* = 6.6 Hz, 14H); **^{1}H NMR (400 MHz, $CDCl_3$)** δ 3.28 (s, 2H), 2.37 (d, *J* = 7.3 Hz, 8H), 1.78–1.70 (m, 4H), 0.90 (d, *J* = 6.6 Hz, 24H).

***N,N,N',N'*-Tetrabenzyldiaminomethane (2e)**

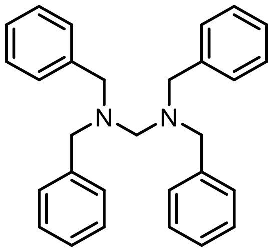

92% yield; **^{1}H NMR (400 MHz, $CDCl_3$)** δ 7.33–7.26 (m, 16H), 7.26–7.22 (m, 4H), 3.63 (s, 8H), 3.11 (s, 2H); **^{13}C NMR (100 MHz, $CDCl_3$)** δ 139.9 (4C), 129.1 (8C), 128.3 (8C), 126.9 (4C), 72.4, 56.3 (4C); **IR (neat) ν_{max}:** 3060, 3026, 2927, 2795, 1493, 1451, 1245, 1125, 1072, 1028, 974, 914; **HRMS (ESI+):** exact mass calculated for $[M+H]^+$ ($C_{29}H_{31}N_2$) requires *m/z* 407.2482, found *m/z* 198.1281 (corresponding to *N,N*-dibenzylamine).

***N,N,N',N'*-Tetraallyldiaminomethane (2f)**

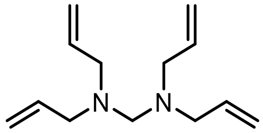

70% yield; **^{1}H NMR (400 MHz, CDCl$_3$)** δ 5.89–5.77 (m, 4H), 5.17–5.06 (m, 8H), 3.15 (dt, *J* = 6.4, 1.3 Hz, 8H), 3.11 (s, 2H); **^{13}C NMR (100 MHz, CDCl$_3$)** δ 136.4 (4C), 116.8 (4C), 72.5, 54.7 (4C); **IR (neat) ν_{max}:** 3077, 2978, 2920, 2801, 1642, 1446, 1417, 1399, 1350, 1259, 1159, 994, 913; **HRMS (ESI+):** exact mass calculated for $[M+H]^+$ ($C_{13}H_{23}N_2$) requires *m/z* 207.1865, found *m/z* 98.0694 (corresponding to *N,N*-diallylamine).

***N,N,N',N'*-Tetrakis(methyl-d$_3$)diaminomethane (2a-d$_{12}$)**

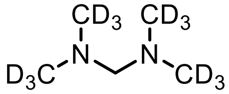

yield not determined–isolated as 35 wt% solution in diethyl ether and used without further purification; **^{1}H NMR (600 MHz, CDCl$_3$)** δ 2.69 (s, 2H).

***N,N'*-Dibutyl-*N,N'*-bis(butyl-d$_9$)diaminomethane (2b-d$_{18}$)**

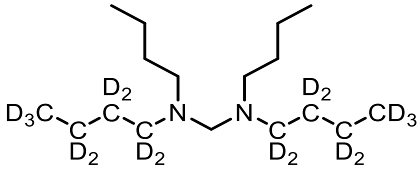

87% yield; **^{1}H NMR (400 MHz, CDCl$_3$)** δ 2.99 (s, 2H), 2.43 (t, *J* = 7.5 Hz, 4H), 1.42–1.24 (m, 8H), 0.90 (t, *J* = 7.2 Hz, 6H); **^{13}C NMR (100 MHz, CDCl$_3$)** δ 75.6, 52.0 (2C), 51.1 (t, *J* = 20.3 Hz), 29.6 (2C), 20.9 (2C), 14.3 (2C), 13.0 (t, *J* = 19.1 Hz)–two signals could not be identified; **IR (neat) ν_{max}:** 2956, 2929, 2861, 2215, 1463, 1376, 1190, 1057; **HRMS (ESI+):** exact mass calculated for $[M+H]^+$ ($C_{17}H_{21}D_{18}N_2$) requires *m/z* 289.4238, found *m/z* 139.2156 (corresponding to *N*-butylbutan-d$_9$-1-amine).

Di(piperidin-1-yl)methane (2g)

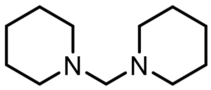

92% yield; **^{1}H NMR (400 MHz, CDCl$_3$)** δ 2.83 (s, 2H), 2.40 (t, *J* = 5.5 Hz, 8H), 1.54 (pent, *J* = 5.5 Hz, 8H), 1.46–1.39 (m, 4H). All spectral data were in good accordance with those reported in the literature.[1]

2.2.2 General Procedure for the Synthesis of Eschenmoser's Salts

***N*-benzyl-*N*-methylene-1-phenylmethanaminium chloride (11e)**

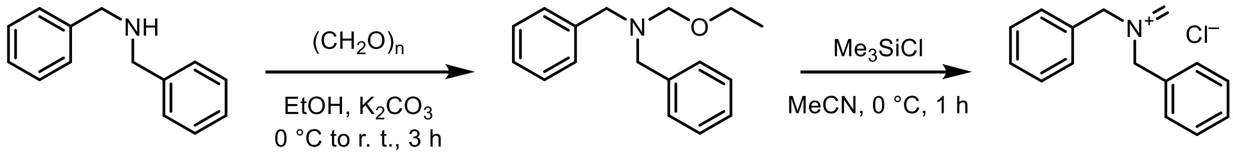

Following the reported procedure,[2] dibenzylamine (2.327 g, 2.31 mL, 12 mmol, 1.20 eq.), dry K_2CO_3 (193 mg, 1.4 mmol, 0.14 eq.) and 2 mL ethanol were mixed in a round-bottom flask at 0 °C and stirred for 5 min. Paraformaldehyde (300 mg, 10 mmol, 1 eq.) was added and stirring was continued for 2 h at room temperature. The inorganic salt was filtered off and excess ethanol was distilled off on a rotary evaporator. The crude was then directly dissolved in 5 mL of dry acetonitrile and added dropwise at 0 °C under argon atmosphere to a solution of freshly distilled chlorotrimethylsilane (1.27 mL, 1.086 g, 10 mmol, 1.00 eq.) in 5 mL of acetonitrile. A white precipitate formed, and the mixture was stirred for 1 h under cooling. Subsequently, 10 mL of anhydrous ether were added, the precipitate was filtered off, washed with 20 mL anhydrous ether, and dried under vacuum, yielding *N*-benzyl-*N*-methylene-1-phenylmethanaminium chloride in 25% yield.

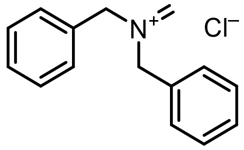

^{1}H NMR (400 MHz, DMSO) δ 9.79 (bs, 2H), 7.55 (bs, 4H), 7.41 (bs, 6H), 4.13 (s, 4H); **^{13}C NMR (100 MHz, DMSO)** δ 131.9 (2C), 130.1 (4C), 128.9 (2C), 128.6 (4C), 81.9 (2C), 49.8 (2C); **IR (neat) ν_{max}:** 2904, 2784, 2720, 2595, 1565, 1497, 1455, 1427, 1211, 1112, 1081, 744, 699; **HRMS (ESI+):** exact mass calculated for $[M]^+$ ($C_{15}H_{16}N$) requires *m/z* 210.1277, found *m/z* 210.1276.

2.2.3 Synthesis of Alkene Substrates

1-(1-Pyrrolidinyl)-10-undecen-1-one (1a)

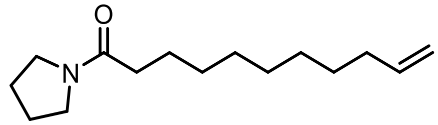

To a solution of pyrrolidine (0.452 mL, 0.391 g, 5.5 mmol, 1.1 equiv.) and triethylamine (1.39 mL, 1.012 g, 10 mmol, 2.00 equiv.) in dichloromethane (20 mL) at 0 °C, 10-undecenoyl chloride (1.08 mL, 1.014 g, 5 mmol, 1 equiv.) was added dropwise and the resulting reaction mixture was allowed to warm to room temperature while stirring overnight (14 h). After this time, a saturated aqueous solution of sodium bicarbonate was added, and the biphasic system was separated. The aqueous phase was extracted with dichloromethane (1 ×) and the organic phases were combined and dried over anhydrous sodium sulfate. The dried solution was filtered and concentrated under reduced pressure. The resulting crude material was purified by flash column chromatography on silica gel (heptane/ethyl acetate) to afford the desired compound; Quant.; **^{1}H NMR (400 MHz, $CDCl_3$)** δ 5.80 (ddt, *J* = 16.9, 10.2, 6.7 Hz, 1H), 4.97 (ddd, *J* = 17.1, 3.7, 1.6 Hz, 1H), 4.91 (ddt, *J* = 10.2, 2.3, 1.2 Hz, 1H), 3.45 (t, *J* = 6.9 Hz, 2H), 3.39 (t, *J* = 6.8 Hz, 2H), 2.25–2.03 (m, 2H), 2.01–1.99 (m, 2H), 1.97–1.95 (m, 2H), 1.87–1.80 (m, 2H), 1.65–1.59 (m, 2H), 1.39–1.22 (m, 10H). All spectral data were in good accordance with those reported in the literature.[3]

Undec-10-en-1-yl acetate (1v)

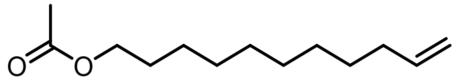

To a solution of 10-undecen-1-ol (0.50 g, 3.20 mmol, 1.00 equiv.) in pyridine (10 mL) was added acetic anhydride (0.91 mL, 9.60 mmol, 3.00 equiv.). After stirring for 3 h at 60 °C, the reaction mixture was diluted with ethyl acetate (25 mL) and washed sequentially with 1 N HCl (25 mL x 5) and brine (25 mL). The organic layer was dried over anhydrous magnesium sulfate, filtered and the filtrate was concentrated. The crude material was purified by flash column chromatography on silica gel (pentane/ethyl acetate) to afford the title compound. 75% yield; **^{1}H NMR (600 MHz, $CDCl_3$)** δ 5.83–5.78 (m, 1H), 5.00–4.91 (m, 2H), 2.04 (s, 3H), 1.62–1.59 (m, 2H), 1.38–1.27 (m, 14H). All spectral data were in good accordance with those reported in the literature.[4]

Diethyl oct-7-en-1-ylphosphonate (1x)

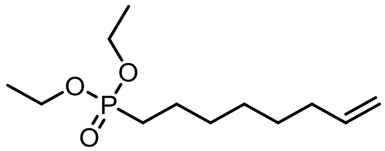

To a round-bottomed flask under inert atmosphere, sodium hydride (60% dispersion in mineral oil, 520 mg, 13.0 mmol, 1.30 equiv.), anhydrous THF (70 mL), and diethyl phosphite (1.80 g, 13.0 mmol,

1.30 equiv.) were added. After stirring at 0 °C for 0.5 h, and subsequently at reflux (66 °C) for 1.5 h, 8-bromo-1-octene (1.91 g, 10.0 mmol, 1.00 equiv.) was added at 0 °C. After stirring at ambient temperature (23 °C) for 24 h, water (50 mL) was added. The phases were separated, and the aqueous phase was extracted with dichloromethane (3 × 50 mL). The organic phases were dried over anhydrous magnesium sulfate, filtered and concentrated. The crude residue was purified by flash column chromatography over silica gel (n-heptane/ethyl acetate). 90% yield; **^{1}H NMR (600 MHz, $CDCl_3$)** δ 5.80 (ddt, *J* = 16.9, 10.2, 6.7 Hz, 1H), 5.01–4.91 (m, 2H), 4.15–4.04 (m, 4H), 2.06–2.01 (m, 2H), 1.76–1.68 (m, 2H), 1.68–1.54 (m, 2H), 1.40–1.24 (m, 11H). All spectral data were in good accordance with those reported in the literature.[5]

_N_-(Pyridin-4-yl)undec-10-enamide (1y)

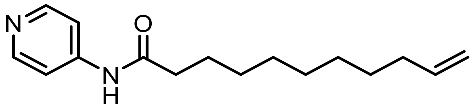

To a mixture of pyridin-4-amine (471 mg, 5.00 mmol, 1.00 equiv.) and triethylamine (1.50 mL, 11.0 mmol, 2.20 equiv.) in dichloromethane (20 mL) was added 10-undecenoylchloride (1.00 mL, 5.00 mmol, 1.00 equiv.) at 0 °C. The mixture was stirred for 12 h at ambient temperature (23 °C), after which aqueous HCl (1 M, 10 mL) was added. The phases were separated, and the aqueous phase was extracted with dichloromethane (3 × 15 mL). The combined organic phases were dried over anhydrous sodium sulfate, filtered and subsequently concentrated under reduced pressure. Flash column chromatography on silica gel (heptane/ethyl acetate) of the resulting crude material afforded the title compound. 85% yield; **^{1}H NMR (600 MHz, $CDCl_3$)** δ 8.51 (dd, *J* = 4.8, 1.5 Hz, 2H), 7.66 (s, 1H), 7.51 (dd, *J* = 4.8, 1.5 Hz, 2H), 5.82 (ddt, *J* = 16.9, 10.2, 6.7 Hz, 1H), 5.01 (ddd, *J* = 17.1, 3.5, 1.5 Hz, 1H), 4.96–4.94 (m, 1H), 2.42–2.39 (m, 2H), 2.07–2.04 (m, 2H), 1.74–1.73 (m, 2H), 1.39–1.28 (m, 10H); **^{13}C NMR (150 MHz, $CDCl_3$)** δ 172.3, 150.7, 150.6, 145.3, 139.3, 114.3, 113.6, 113.6, 38.0, 33.9, 29.4 (2C), 29.3, 29.2, 29.0, 25.4; ; **IR (neat) ν_{max}:** 2925, 2854, 1707, 1683, 1592, 1517, 1415, 1329, 1294, 1210, 999; **HRMS (ESI+):** exact mass calculated for $[M+H]^+$ ($C_{16}H_{25}N_2O$) requires *m/z* 261.1961, found *m/z* 261.1958.

Dodec-11-enenitrile (1z)

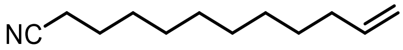

A mixture of 9-decen-1-ol (0.892 mL, 0.781 g, 5 mmol, 1 equiv.), triethylamine (0.767 mL, 0.557 g, 5.5 mmol, 1.5 equiv.), and *p*-toluenesulfonyl chloride (0.953 g, 5 mmol, 1 equiv.) in dichloromethane (20 mL) was stirred for 12 h at ambient temperature (23 °C). After this time, the reaction mixture was diluted with water (20 mL) and extracted with chloroform (3 × 20 mL). The combined organic phases were washed with a saturated aqueous solution of ammonium chloride, and subsequently dried over anhydrous magnesium sulfate. After removal of the solvent under reduced pressure, potassium cyanide (0.358 g, 5.5 mmol, 1.1 equiv.) and DMSO (30 mL) were added and the resulting suspension was stirred at ambient temperature (23 °C) for 14 h. After this time, water (100 mL) was added and the resulting

solution was extracted with diethyl ether (4 × 30 mL). The combined organic phases were washed with a saturated aqueous solution of sodium bicarbonate (70 mL) and subsequently dried over anhydrous magnesium sulfate. The dried solution was filtered and the filtrate was concentrated under reduced pressure to afford the crude product. The crude material was purified by flash column chromatography on silica gel (heptane/ethyl acetate) to afford the title compound. 67% yield; **^{1}H NMR (600 MHz, $CDCl_3$)** δ 5.86–5.80 (m, 1H), 5.03–4.95 (m, 2H), 2.37–2.35 (t, *J* = 7.8 Hz, 2H), 2.08–2.04 (q, *J* = 7.2 Hz, 2H), 1.70–1.65 (q, *J* = 6.6 Hz, 2H), 1.47–1.45 (m, 2H), 1.40–1.38 (m, 2H), 1.28 (m, 8H). All spectral data were in good accordance with those reported in the literature.[6]

N-Butylundec-10-en-1-amine (1ac)

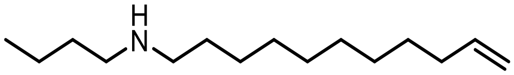

To a stirred solution of 10-undecenal (505 mg, 3.00 mmol, 1.00 equiv.) in MeOH (3 mL) was added *n*-butylamine (594 µL, 6.00 mmol, 2.00 equiv.), and the mixture was stirred at ambient temperature (23 °C) for 2 h. After this time, the reaction mixture was cooled to 0 °C and sodium borohydride (57.0 mg, 1.50 mmol, 0.75 equiv.) was added. The resulting mixture was stirred at 0 °C for 30 min, after which excess reductant was quenched by the addition of water (3 mL). The crude mixture was extracted with dichloromethane (3 × 5 mL), the combined organic phases were dried over anhydrous magnesium sulfate and the dried solution was filtered. The filtrate was concentrated under reduced pressure and the resulting crude material was purified by flash column chromatography on silica gel (dichloromethane/MeOH/NH_4OH) to afford the title compound. 66% yield; **^{1}H NMR (600 MHz, $CDCl_3$)** δ 5.81 (ddt, *J* = 16.9, 10.2, 6.7 Hz, 1H), 4.99 (dd, *J* = 17.1, 1.4 Hz, 1H), 4.93–4.90 (m, 1H), 2.67 (ddd, *J* = 12.7, 8.0, 3.0 Hz, 3H), 2.03 (q, *J* = 7.0 Hz, 2H), 1.67–1.51 (m, 3H), 1.43–1.20 (m, 15H), 0.92 (t, *J* = 7.3 Hz, 3H). All spectral data were in good accordance with those reported in the literature.[7]

N,N-Diethylundec-10-en-1-amine (1ad)

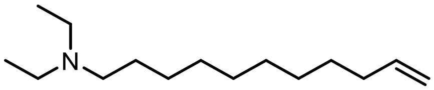

To a stirred solution of 10-undecenal (505 mg, 3.00 mmol, 1.00 equiv.) in MeOH (3 mL) was added *N,N*-diethylamine (621 µL, 6.00 mmol, 2.00 equiv.), and the mixture was stirred at ambient temperature (23 °C) for 2 h. After this time, the reaction mixture was cooled to 0 °C and sodium borohydride (57.0 mg, 1.50 mmol, 0.75 equiv.) was added. The resulting mixture was stirred at 0 °C for 30 min, after which excess reductant was quenched by the addition of water (3 mL). The crude mixture was extracted with dichloromethane (3 × 5 mL), the combined organic phases were dried over anhydrous magnesium sulfate and the dried solution was filtered. The filtrate was concentrated under reduced pressure and the resulting crude material was purified by flash column chromatography on silica gel (dichloromethane/MeOH/NH_4OH) to afford the title compound. 30% yield. **^{1}H NMR (600 MHz, $CDCl_3$)** δ 5.81 (ddt, *J* = 16.9, 10.2, 6.7 Hz, 1H), 4.99 (ddd, *J* = 17.1, 3.6, 1.6 Hz, 1H), 4.93–4.91 (m, 1H), 2.51 (q,

J = 7.2 Hz, 4H), 2.40–2.38 (m, 2H), 2.05–2.01 (m, 2H), 1.44–1.42 (m, 2H), 1.38–1.36 (m, 2H), 1.27–1.24 (m, 10H), 1.01 (t, *J* = 7.2 Hz, 6H); **^{13}C NMR (150 MHz, $CDCl_3$)** δ 139.4, 114.2, 53.2, 47.0 (2C), 34.0, 29.8, 29.7, 29.6, 29.3, 29.1, 27.9, 27.2, 11.8 (2C); **IR (neat) ν_{max}:** 2968, 2923, 2853, 2797, 1464, 1380, 1202, 1070, 991, 908; **HRMS (ESI+):** exact mass calculated for $[M+H]^+$ ($C_{15}H_{32}N$) requires *m/z* 226.2529, found *m/z* 226.2527.

2.3 General Procedures for Hydroaminomethylation with Eschenmoser's Salts (Method A)

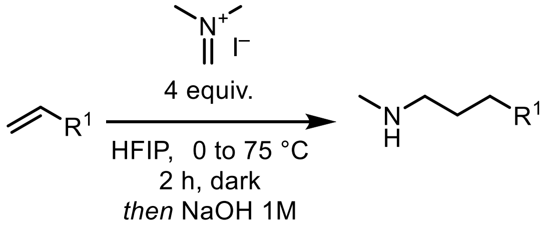

A round-bottom flask protected from light was charged with *N,N*-dimethylmethyleneiminium iodide (Eschenmoser´s iodide, 4 equiv.) and a magnetic stir-bar under argon-atmosphere and was cooled to 0 °C. After this, the alkene (1 equiv., 0.5 mmol) and 1,1,1,3,3,3-hexafluoroisopropanol (HFIP, 0.6 M with respect to the alkene) were added. After completed addition of the solvent, the flask was sealed and placed in an oil bath at 75 °C. The reaction was vigorously stirred at this temperature for 2 h, after which it was allowed to cool to room temperature. Subsequently, aqueous sodium hydroxide (1 M) was added until the reaction mixture reached pH 12. The resulting biphasic mixture was separated, and the aqueous phase was extracted with dichloromethane (3 x 200 mL/mmol). The combined organic phases were then dried over anhydrous potassium carbonate and filtered. The filtrate was concentrated under reduced pressure[B] to afford the crude product, which was purified by flash column chromatography on silica gel (dichloromethane/MeOH/NH_4OH 19:1:0.15) to afford the analytically pure desired product.

2.4 General Procedures for Hydroaminomethylation with Aminals (Method B)

2.4.1 General Procedure A for Hydroaminomethylation of Alkenes

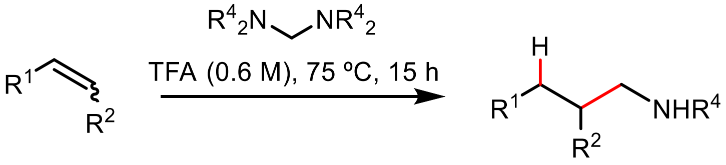

A round-bottom flask charged with *N,N,N',N'*-tetraalkyldiaminomethane (4 equiv.) and a magnetic stir-bar under argon-atmosphere was cooled to 0 °C. After this, trifluoroacetic acid (TFA, 0.6 M with respect to the alkene) was added slowly, maintaining the low temperature of the contents of the flask. After completed addition of TFA, the alkene (1.0 equiv., 0.5 mmol) was added in one portion, the flask was sealed and placed in an oil bath at 75 °C. The reaction was vigorously stirred at this temperature for 15 h, after which it was allowed to cool to room temperature. Subsequently, volatile components were removed under reduced pressure.[B] The crude mixture was then treated with aqueous sodium hydroxide (1 M – 2 mL/1 mmol substrate) and dichloromethane (1 mL/1 mmol substrate) and stirred vigorously at room temperature for 1 h. After this time, aqueous sodium hydroxide (5 M) was added until the reaction

[B] Several compounds have a low boiling point. For volatile compounds, solvents were removed using a temperature of 40 °C and a minimum pressure of 100 mbar.

mixture reaches pH 12. The resulting biphasic mixture was separated, and the aqueous phase was extracted with dichloromethane (3 x 200 mL). The combined organic phases were then dried over anhydrous sodium sulfate and filtered. The filtrate was concentrated under reduced pressure to afford the crude product, which was purified by flash column chromatography on silica gel (dichloromethane/MeOH/NH_4OH 19:1:0.15) to afford the analytically pure desired product.

2.4.2 General Procedure B for Hydroaminomethylation of Alkynes

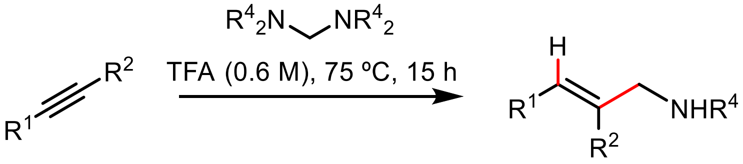

A round-bottom flask charged with *N,N,N′,N′*-tetraalkyldiaminomethane (4 equiv.) and a magnetic stir-bar under argon-atmosphere was cooled to 0 °C and dissolved in DCE (1.2 M with respect to the alkyne). After this, trifluoroacetic acid (11.2 equiv. with respect to the alkyne) was added slowly, maintaining the low temperature of the contents of the flask. After completed addition of TFA, the alkyne (0.5 mmol) was added in one portion, the flask was sealed and placed in an oil bath at 75 °C. The reaction was vigorously stirred at this temperature for 15 h, after which it was allowed to cool to room temperature. Subsequently, volatile components were removed under reduced pressure.[B] After this time, aqueous sodium hydroxide (1 M) was added until the reaction mixture reaches pH 12 and the mixture was extracted with dichloromehane (3 x 200 mL). The combined organic phases were then dried over anhydrous sodium sulfate and filtered. The filtrate was concentrated under reduced pressure to afford the crude product, which was purified by flash column chromatography on silica gel (dichloromethane/MeOH/NH_4OH 19:1:0.15) to afford the analytically pure desired product.

2.4.3 General Procedure C for Hydroaminomethylation of Alkynes

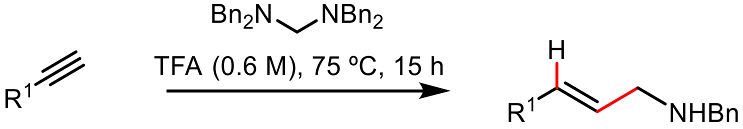

A round-bottom flask charged with *N,N,N',N'*-tetrabenzyldiaminomethane (1.5 equiv.) and a magnetic stir-bar under argon-atmosphere is cooled to 0 °C and dissolved in DCE (1.2 M with respect to the alkyne) slowly, maintaining the low temperature of the contents of the flask. After completed addition of TFA, the alkyne (0.5 mmol) was added in one portion, the flask was sealed and placed in an oil bath at 75 °C. The reaction was vigorously stirred at this temperature for 15 h, after which it is allowed to cool to room temperature. Subsequently, volatile components were removed under reduced pressure.[B] After this time, aqueous sodium hydroxide (1 M) was added until the reaction mixture reaches pH 12 and the mixture was extracted with dichloromethane (3 x 200 mL). The combined organic phases were then dried over anhydrous sodium sulfate and filtered. The filtrate was concentrated under reduced pressure to afford

the crude product, which was purified by flash column chromatography on silica gel (dichloromethane/MeOH/NH_4OH 19:1:0.15) to afford the analytically pure desired product.

2.5 Analytical Data

12-(Methylamino)-1-(pyrrolidin-1-yl)dodecan-1-one (3a)

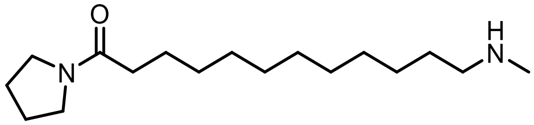

Prepared according to procedure A, method B with 4 equiv. *N,N,N′,N′*-tetramethyldiaminomethane; (119 mg, 84% yield); **^{1}H NMR (400 MHz, $CDCl_3$)** δ 3.45 (t, *J* = 6.8 Hz, 2H), 3.40 (t, *J* = 6.9 Hz, 2H), 2.54 (t, *J* = 7.2 Hz, 2H), 2.42 (s, 3H), 2.24 (t, *J* = 7.8 Hz, 2H), 1.93 (quin, *J* = 6.6 Hz, 2H), 1.83 (app quin, *J* = 6.6 Hz, 2H), 1.63 (app quin, *J* = 7.3 Hz, 2H), 1.46 (app quin, *J* = 6.9 Hz, 2H), 1.35–1.22 (m, 14H), 1.07 (br s, 1H); **^{13}C NMR (100 MHz, $CDCl_3$)** δ 171.9, 52.4, 46.7, 45.7, 36.7, 35.0, 30.1, 29.7 (2C), 29.7 (2C), 29.6, 29.6, 27.5, 26.3, 25.1, 24.6; **IR (neat) ν_{max}:** 3439, 2924, 2852, 1636, 1558, 1439, 1382, 1345, 1310, 1254, 1227; **HRMS (ESI+):** exact mass calculated for $[M+H]^+$ ($C_{17}H_{35}N_2O$) requires *m/z* 283.2744, found *m/z* 283.2743.

12-(Butylamino)-1-(pyrrolidin-1-yl)dodecan-1-one (3b)

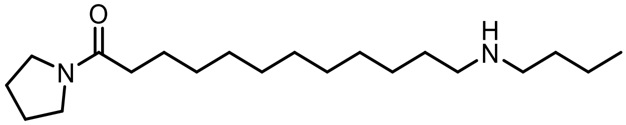

Prepared according to procedure A, method B with 4 equiv. *N,N,N′,N′*-tetrabutyldiaminomethane; (138 mg, 85% yield); **^{1}H NMR (400 MHz, $CDCl_3$)** δ 3.45 (t, *J* = 6.8 Hz, 2H), 3.40 (t, *J* = 6.9 Hz, 2H), 2.61–2.54 (m, 4H), 2.24 (t, *J* = 7.8 Hz, 2H), 1.98–1.90 (m, 2H), 1.87–1.80 (m, 2H), 1.63 (app quin, *J* = 7.3 Hz, 2H), 1.51–1.41 (m, 4H), 1.37–1.23 (m, 16H), 1.06 (br s, 1H), 0.91 (t, *J* = 7.4 Hz, 3H); **^{13}C NMR (100 MHz, $CDCl_3$)** δ 172.0, 50.3, 50.0, 46.7, 45.7, 35.0, 32.5, 30.4, 29.7 (2C), 29.7 (2C), 29.6, 29.6, 27.6, 26.3, 25.1, 24.6, 20.7, 14.2; **IR (neat) ν_{max}:** 2924, 2854, 1643, 1432, 1345; **HRMS (ESI+):** exact mass calculated for $[M+H]^+$ ($C_{20}H_{41}N_2O$) requires *m/z* 325.3213, found *m/z* 325.3214.

12-(Propylamino)-1-(pyrrolidin-1-yl)dodecan-1-one (3c)

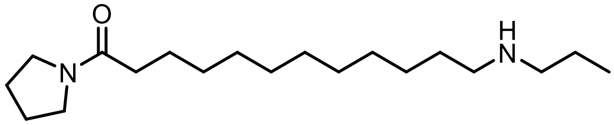

Prepared according to procedure A, method B with 4 equiv. *N,N,N′,N′*-tetrapropyldiaminomethane; (142 mg, 92% yield); **^{1}H NMR (600 MHz, $CDCl_3$)** δ 3.45 (t, *J* = 6.8 Hz, 2H), 3.40 (t, *J* = 6.9 Hz, 2H), 2.54 (app dt, *J* = 11.7, 7.3 Hz, 4H), 2.23 (t, *J* = 7.8 Hz, 2H), 1.93 (app quin, *J* = 6.8 Hz, 2H), 1.83 (app quin, *J* = 6.8 Hz, 2H), 1.63 (app quin, *J* = 7.4 Hz, 2H), 1.53–1.42 (m, 4H), 1.35–1.20 (m, 15H), 0.90 (t, *J* = 7.4 Hz, 3H); **^{13}C NMR (150 MHz, $CDCl_3$)** δ 171.9, 52.4, 50.3, 46.7, 45.7, 35.0, 30.4, 29.7 (2C), 29.7, 29.7, 29.6, 29.6, 27.6, 26.3, 25.1, 24.6, 23.4, 12.0; **IR (neat) ν_{max}:** 2924, 2853, 1641, 1432, 1343; **HRMS (ESI+):** exact mass calculated for $[M+H]^+$ ($C_{19}H_{39}N_2O$) requires *m/z* 311.3057, found *m/z* 311.3058.

11-(Isobutylamino)-1-(pyrrolidin-1-yl)undecan-1-one (3d)

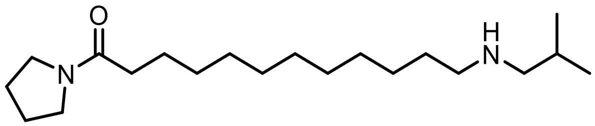

Prepared according to procedure A, method B with 4 equiv. *N,N,N′,N′*-tetraisobutyldiaminomethane, (136 mg, 84% yield); **^{1}H NMR (600 MHz, $CDCl_3$)** δ 3.45 (t, *J* = 6.9 Hz, 2H), 3.40 (t, *J* = 6.8 Hz, 2H), 2.58–2.56 (m, 2H), 2.41 (d, *J* = 6.8 Hz, 2H), 2.25–2.23 (m, 2H), 1.93 (dd, *J* = 13.5, 6.8 Hz, 2H), 1.85 (dd, *J* = 13.7, 6.8 Hz, 2H), 1.75 (m, 1H), 1.63 (dt, *J* = 15.1, 7.5 Hz, 2H), 1.49–1.46 (m, 2H), 1.35–1.22 (m, 15H), 0.90 (d, *J* = 6.6 Hz, 6H); **^{13}C NMR (150 MHz, $CDCl_3$)** δ 172.0, 58.3, 50.3, 46.8, 45.7, 35.0, 30.2, 29.7 (2C), 29.7 (2C), 29.6, 29.6, 28.4, 27.5, 26.3, 25.1, 24.7, 20.9 (2C) ppm; **IR (neat) ν_{max}:** 2922, 2852, 2808, 1641, 1429, 1365, 1194, 1127; **HRMS (ESI+):** exact mass calculated for $[M+H]^+$ ($C_{20}H_{41}N_2O$) requires *m/z* = 325.3213, found *m/z* 325.3209.

12-(Benzylamino)-1-(pyrrolidin-1-yl)dodecan-1-one (3e)

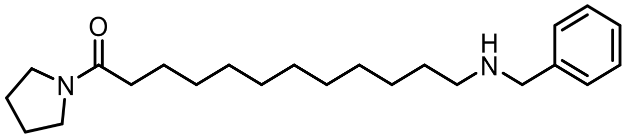

Prepared according to procedure A, method B with 1.5 equiv. *N,N,N′,N′*-tetrabenzyldiaminomethane, (131 mg, 73% yield). **^{1}H NMR (400 MHz, $CDCl_3$)** δ 7.27–7.25 (m, 4H), 7.21–7.15 (m, 1H), 3.73 (s, 2H), 3.39 (t, *J* = 6.9 Hz, 2H), 3.33 (t, *J* = 6.8 Hz, 2H), 2.58–2.54 (m, 2H), 2.20–2.16 (m, 2H), 1.98 (br s, 1H), 1.88–1.75 (m, 2H), 1.79–1.75 (m, 2H), 1.59–1.56 (m, 2H), 1.47–1.43 (m, 2H), 1.22 (m, 15H); **^{13}C NMR (100 MHz, $CDCl_3$)** δ 171.8, 140.1, 128.4 (2C), 128.2 (2C), 54.0, 53.2, 49.4, 46.6, 45.6, 34.8, 29.9, 29.5 (2C), 29.5 (2C), 29.5, 29.4, 27.3, 26.1, 24.9, 26.4; **IR (neat) ν_{max}:** 3061, 3026, 2917, 2812, 1602, 1494, 1453, 1115, 734, 697; **HRMS (ESI+):** exact mass calculated for $[M+H]^+$ ($C_{23}H_{39}N_2O$) requires *m/z* 359.3057, found *m/z* 359.3056.

12-(Allylamino)-1-(pyrrolidin-1-yl)dodecan-1-one (3f)

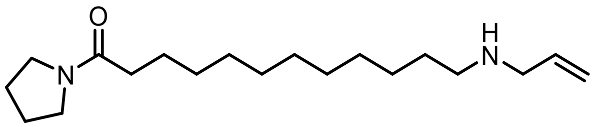

Prepared according to procedure A, method B with 4 equiv. *N,N,N′,N′*-tetraallyldiaminomethane, (133 mg, 86% yield). **^{1}H NMR (400 MHz, $CDCl_3$)** δ 5.96–5.86 (m, 1H), 5.12 (dddd, *J* = 32.1, 10.2, 3.2, 1.4 Hz, 2H), 3.46 (t, *J* = 6.9 Hz, 2H), 3.40 (t, *J* = 6.8 Hz, 2H), 3.24 (dt, *J* = 6.0, 1.4 Hz, 2H), 2.61–2.58 (m, 2H), 2.26–2.22 (m, 2H), 1.96–1.91 (m, 2H), 1.88–1.83 (m, 2H), 1.66–1.60 (m, 2H), 1.50–1.44 (m, 2H), 1.30–1.27 (m, 14H), 0.84 (br s, 1H); **^{13}C NMR (100 MHz, $CDCl_3$)** δ 172.0, 137.3, 115.7, 52.8, 49.7, 46.8, 45.7, 35.0, 30.3, 29.7 (2C), 29.7 (2C), 29.6, 29.6, 27.5, 26.3, 25.1, 24.6.; **IR (neat) ν_{max}:** 2922, 2852, 1641, 1432, 1168, 1033, 994, 914; **HRMS (ESI+):** exact mass calculated for $[M+H]^+$ ($C_{19}H_{37}N_2O$) requires *m/z* 309.2900, found *m/z* 309.2928.

12-Amino-1-(pyrrolidin-1-yl)dodecan-1-one (4f)

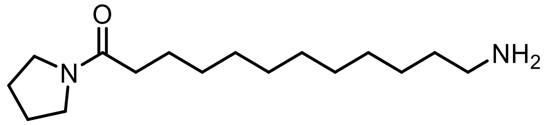

The *N*-allyl compound (**1f**, 0.095 mmol, 29.3 mg) was dissolved in 2 ml ethanol and palladium on charcoal (10%, 0.5 equivalents, 5 mg) was added. The mixture was refluxed for 24h. The solid was filtered off over célite, the ethanol was removed at reduced pressure and the crude residue was purified by flash column chromatography over silica gel (dichloromethane/MeOH/NH_4OH 18/1/0.15) to afford the title compound.

(21.6 mg, 85% yield). **^{1}H NMR (600 MHz, $CDCl_3$)** δ 3.45 (t, *J* = 6.9 Hz, 2H), 3.40 (t, *J* = 6.8 Hz, 2H), 2.66 (t, *J* = 7.1 Hz, 2H), 2.30–2.18 (m, 2H), 1.93 (p, *J* = 6.8 Hz, 2H), 1.84 (p, *J* = 6.9 Hz, 2H), 1.64–1.61 (m, 3H), 1.47–1.25 (m, 17H); **^{13}C NMR (150 MHz, $CDCl_3$)** δ 172.0, 46.7, 45.7, 42.4, 35.0, 34.0, 29.7, 29.7, 29.6, 29.6, 27.0, 26.3, 25.1, 24.6; **IR (neat) ν_{max}:** 3415, 2922, 2851, 1630, 1430, 1342, 1008; **HRMS (ESI+):** exact mass calculated for $[M+H]^+$ ($C_{16}H_{33}NO$) requires *m/z* 269.2587, found *m/z* 269.2590.

***N*-Methyldodecan-1-amine (3g)**

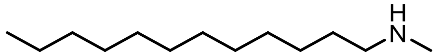

Prepared according to procedure A, method B with 4 equiv. *N,N,N′,N′*-tetramethyldiaminomethane; (61 mg, 61% yield); preparation with 4 equivalents of Eschenmoser's iodide afforded the product in 75% yield (see General Procedures for Redox-Neutral Hydroaminomethylation with Eschenmoser´s salts); **^{1}H NMR (400 MHz, $CDCl_3$)** δ 2.54 (t, *J* = 7.3 Hz, 2H), 2.42 (s, 3H), 1.46 (app quin, *J* = 7.1 Hz, 2H), 1.32–1.21 (m, 18H), 1.10 (br s, 1H), 0.87 (t, *J* = 7.0 Hz, 3H); **^{13}C NMR (100 MHz, $CDCl_3$)** δ 52.4, 36.8, 32.1, 30.1, 29.8, 29.8, 29.7 (2C), 29.7, 29.5, 27.5, 22.8, 14.3; **IR (neat) ν_{max}:** 2955, 2921, 2852, 1465, 1380, 1308, 722; **HRMS (ESI+):** exact mass calculated for $[M+H]^+$ ($C_{13}H_{30}N$) requires *m/z* 200.2373, found *m/z* 200.2367.

***N*-Benzyl-3,4,4-trimethylpentan-1-amine (3h)**

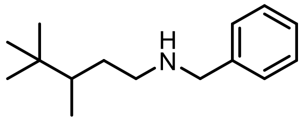

Prepared according to procedure A, method B with 1.5 equiv. *N,N,N′,N′*-tetrabenzyldiaminomethane; (57 mg, 52% yield). **^{1}H NMR (400 MHz, $CDCl_3$)** δ 7.36–7.27 (m, 5H), 3.87–3.79 (m, 2H), 2.75 (ddd, *J* = 10.9, 9.8, 4.5 Hz, 1H), 2.59 (ddd, *J* = 11.2, 8.8, 6.6 Hz, 1H), 1.80–1.73 (m, 1H), 1.65 (br s, 1H, NH), 1.28–1.14 (m, 2H), 0.87 (s, 9H), 0.85 (d, *J* = 6.5 Hz, 3H) ppm; **^{13}C NMR (100 MHz, $CDCl_3$)** δ 140.5, 128.5 (2C), 128.3 (2C), 127.1, 54.3, 49.0, 41.1, 33.1, 32.2, 27.4 (3C), 14.6 ppm; **IR (neat) ν_{max}:** 2958, 2865, 2831, 1453, 1364, 1119, 1028, 732, 697; **HRMS (ESI+):** exact mass calculated for $[M+H]^+$ ($C_{15}H_{26}N$) requires *m/z* 220.2060, found *m/z* 220.2057.

tert-Butyl (cycloheptylmethyl)(methyl)carbamate (3i)

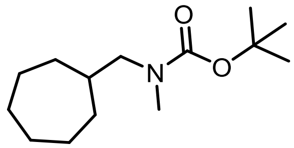

A round-bottom flask charged with *N,N,N′,N′*-tetramethyldiaminomethane (0.307 mL, 230 mg, 4.5 equiv.) and a magnetic stir-bar under argon-atmosphere was cooled to 0 °C. After this, trifluoroacetic acid (TFA, 0.6 M with respect to the alkene) was added slowly, maintaining the low temperature of the contents of the flask. After completed addition of TFA, cycloheptene (50.1 mg, 1 equiv.) was added in one portion, the flask was sealed and placed in an oil bath at 75 °C. The reaction was vigorously stirred at this temperature for 15 h, after which it was allowed to cool to room temperature. Subsequently, volatile components were removed under reduced pressure. The crude mixture was then treated with aqueous sodium hydroxide (1 M–2 mL/1 mmol substrate) and chloroform (1 mL/1 mmol substrate) and stirred vigorously at room temperature for 1 h. After this time, aqueous sodium hydroxide (5 M) was added until the reaction mixture reaches pH 12. The resulting biphasic mixture was separated, and the aqueous phase was extracted with chloroform (3 x 200 mL). The combined organic phases were then dried over anhydrous sodium sulfate and filtered. The filtrate was concentrated under reduced pressure to afford the crude product, which was subsequently protected. The crude mixture was dissolved in anhydrous chloroform (0.2 M), triethylamine (139 μL, 2 equiv.) and di-*tert*-butyldicarbonate (Boc anhydride, 218 mg, 2 equiv.) were added. The solution was stirred for 5 hours at 23 °C, then treated with aqueous sodium hydroxide (1 M–2 mL/1 mmol substrate) and chloroform (1 mL/1 mmol substrate). The resulting biphasic mixture was separated, and the aqueous phase was extracted with chloroform (3 x 20 mL). The combined organic phases were then dried over anhydrous sodium sulfate and filtered. The filtrate was concentrated under reduced pressure to afford the crude product, which was purified by flash column chromatography on silica gel (toluene) to afford the analytically pure desired product.

(98 mg, 78% yield); **^{1}H NMR (400 MHz, $CDCl_3$)** δ 3.01–2.99 (m, 2H), 2.81 (s, 3H), 1.82–1.72 (m, 1H), 1.70–1.46 (m, 8H), 1.45 (s, 9H), 1.41–1.34 (m, 2H), 1.17–1.07 (m, 2H); **^{13}C NMR (100 MHz, $CDCl_3$)** δ 156.3, 79.2, 55.1, 38.0, 34.5, 31.7 (2C), 28.8 (2C), 28.6 (3C), 26.3 (2C); **IR (neat) ν_{max}:** 2919, 2853, 1692, 1479, 1457, 1393, 1364, 1276, 1245, 1161, 1132, 881, 769; **HRMS (ESI+):** exact mass calculated for $[M+Na]^+$ ($C_{14}H_{27}NO_2Na$) requires *m/z* 264.1934, found *m/z* 264.1934.

tert-Butyl (((1*S*,2*S*,4*R*)-bicyclo[2.2.1]heptan-2-yl)methyl)(methyl)carbamate (3j)

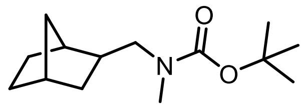

A round-bottom flask charged with *N,N,N′,N′*-tetramethyldiaminomethane (0.307 mL, 230 mg, 4.5 equiv.) and a magnetic stir-bar under argon-atmosphere was cooled to 0 °C. After this, trifluoroacetic acid (TFA, 0.6 M with respect to the alkene) was added slowly, maintaining the low temperature of the contents of the flask. After completed addition of TFA, the norbornene (47.1 mg, 1 equiv.) was added in one portion,

the flask was sealed and placed in an oil bath at 75 °C. The reaction was vigorously stirred at this temperature for 15 h, after which it was allowed to cool to room temperature. Subsequently, volatile components were removed under reduced pressure. The crude mixture was then treated with aqueous sodium hydroxide (1 M–2 mL/1 mmol substrate) and chloroform (1 mL/1 mmol substrate) and stirred vigorously at room temperature for 1 h. After this time, aqueous sodium hydroxide (5 M) was added until the reaction mixture reaches pH 12. The resulting biphasic mixture was separated and the aqueous phase was extracted with chloroform (3 x 200 mL). The combined organic phases were then dried over anhydrous sodium sulfate and filtered. The filtrate was concentrated under reduced pressure to afford the crude product, which was subsequently protected. The crude mixture was dissolved in anhydrous chloroform (0.2 M), triethylamine (139 μL, 2 equiv.) and Boc anhydride (218 mg, 2 equiv.) were added. The solution was stirred for 5 hours at 23 °C, then treated with aqueous sodium hydroxide (1 M – 2 mL/1 mmol substrate) and chloroform (1 mL/1 mmol substrate). The resulting biphasic mixture was separated, and the aqueous phase was extracted with chloroform (3 x 20 mL). The combined organic phases were then dried over anhydrous sodium sulfate and filtered. The filtrate was concentrated under reduced pressure to afford the crude product, which was purified by flash column chromatography on silica gel (toluene) to afford the analytically pure desired product.

(94 mg, 79% yield); **^{1}H NMR (400 MHz, $CDCl_3$)** δ 3.23–3.09 (m, 1H), 2.97–2.72 (m, 4H),2.21 (s, 1H), 2.02 (s, 1H), 1.75–1.68 (m, 1H), 1.53–1.46 (m, 2H), 1.45 (s, 9H), 1.33–1.28 (m, 2H), 1.16–1.06 (m, 3H), 1.00 (br s, 1H); **^{13}C NMR (100 MHz, $CDCl_3$)** δ 156.2, 79.2, 53.3, 40.8, 38.6, 36.6, 35.2, 34.9, 34.1, 29.9, 29.2, 28.6 (3C); **IR (neat) ν_{max}:** 2951, 2870, 1696, 1454, 1396, 1365, 1158, 1128; **HRMS (ESI+):** exact mass calculated for $[M+Na]^+$ ($C_{14}H_{25}NO_2Na$) requires *m/z* 262.1778, found *m/z* 262.1779.

_N_-Benzyl-1-((1*S*,4*R*)-bicyclo[2.2.1]heptan-2-yl)methanamine (3k)

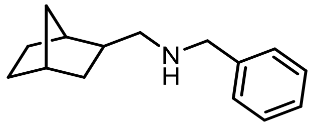

Prepared according to procedure A, method B with 1.5 equiv. *N,N,N′,N′*-tetrabenzyldiaminomethane; (39 mg, 36% yield); **^{1}H NMR (400 MHz, $CDCl_3$)** δ 7.35–7.29 (m, 4H), 7.27–7.21 (m, 1H), 3.78 (s, 2H), 2.50 (dd, *J* = 11.5, 8.1 Hz, 1H), 2.34 (dd, *J* = 11.5, 6.9 Hz, 1H), 2.19 (br s, 1H), 2.07 (br s, 1H), 1.67–1.58 (m, 1H), 1.54–1.46 (m, 2H), 1.46–1.30 (m, 2H), 1.29–1.24 (m, 1H), 1.21–0.99 (m, 4H); **^{13}C NMR (100 MHz, $CDCl_3$)** δ 140.9, 128.5 (2C), 128.2 (2C), 126.9, 55.3, 54.3, 42.6, 39.6, 36.4, 36.4, 35.5, 30.1, 29.0; **IR (neat) ν_{max}**: 2947, 2868, 2801, 1453, 1122, 733, 698; **HRMS (ESI+):** exact mass calculated for $[M+H]^+$ ($C_{15}H_{22}N$) requires *m/z* 216.1747, found *m/z* 216.1745.

N-Methyl-5-phenylpentan-1-amine (3l)

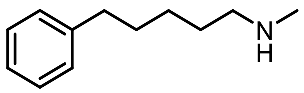

Prepared according to procedure A, method B with 4 equiv. *N,N,N′,N′*-tetramethyldiaminomethane; (81 mg, 91% yield); preparation with 4 equivalents of Eschenmoser's iodide (method A) afforded the product in 79% yield (see General Procedures); **^{1}H NMR (400 MHz, $CDCl_3$)** δ 7.30–7.24 (m, 2H), 7.21–7.15 (m, 3H), 2.62 (t, *J* = 7.8 Hz, 2H), 2.56 (t, *J* = 7.2 Hz, 2H), 2.42 (s, 3H), 1.69–1.60 (m, 2H), 1.56–1.47 (m, 2H), 1.42–1.33 (m, 2H), 0.91 (br s, 1H); **^{13}C NMR (100 MHz, $CDCl_3$)** δ 142.8, 128.5 (2C), 128.4 (2C), 125.7, 52.3, 36.7, 36.0, 31.5, 30.0, 27.1; **IR (neat) ν_{max}:** 3025, 2927, 2854, 2788, 1454, 1381, 1308, 811, 744, 698; **HRMS (ESI+):** exact mass calculated for $[M+H]^+$ ($C_{12}H_{20}N$) requires *m/z* 178.1590, found *m/z* 178.1590.

N-Methyl-3-phenylpropan-1-amine (3m)

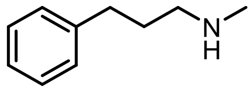

Prepared according to procedure A, method B with 4 equiv. *N,N,N′,N′*-tetramethyldiaminomethane; (61 mg, 82% yield) (50 mmol: 6.41 g, 86% yield); preparation with 4 equivalents of Eschenmoser's iodide (method A) afforded the product in quantitative yield (see General Procedures; yield determined by ^{1}H NMR anaylsis of the crude using mesytilene as internal standard); **^{1}H NMR (400 MHz, $CDCl_3$)** δ 7.30–7.25 (m, 2H), 7.22–7.15 (m, 3H), 2.66 (t, *J* = 7.8 Hz, 2H), 2.61 (t, *J* = 7.2 Hz, 2H), 2.43 (s, 3H), 1.86–1.77 (m, 2H), 1.02 (br s, 1H); **^{13}C NMR (100 MHz, $CDCl_3$)** δ 142.4, 128.5 (2C), 128.4 (2C), 125.9, 51.8, 36.7, 33.8, 31.7; **IR (neat) ν_{max}:** 3026, 2932, 2859, 1541, 1493, 1454, 1383, 1306, 1252, 748, 700; **HRMS (ESI+):** exact mass calculated for $[M+H]^+$ ($C_{10}H_{16}N$) requires *m/z* 150.1277, found *m/z* 150.1274.

N-Methyl-3-(*p*-tolyl)propan-1-amine (3n)

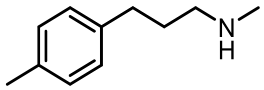

Prepared according to procedure A, method B with 4 equiv. *N,N,N′,N′*-tetramethyldiaminomethane; (69 mg, 85% yield); **^{1}H NMR (400 MHz, $CDCl_3$)** δ 7.09 (app s, 4H), 2.65–2.57 (m, 4H), 2.43 (s, 3H), 2.32 (s, 3H), 1.84–1.76 (m, 2H), 1.06 (br s, 1H); **^{13}C NMR (100 MHz, $CDCl_3$)** δ 139.3, 135.3, 129.1 (2C), 128.4 (2C), 51.8, 36.6, 33.3 31.8, 21.1; **IR (neat) ν_{max}:** 2025, 2855, 2794, 1515, 1457, 1381, 805; **HRMS (ESI+):** exact mass calculated for $[M+H]^+$ ($C_{11}H_{18}N$) requires *m/z* 164.1434, found *m/z* 164.1435.

3-(4-Chlorophenyl)-*N*-methylpropan-1-amine (3o)

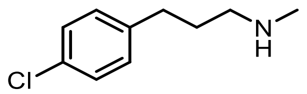

Prepared according to procedure A, method B with 4 equiv. *N,N,N′,N′*-tetramethyldiaminomethane; (78 mg, 86% yield); **^{1}H NMR (400 MHz, CDCl$_3$)** δ 7.26–7.21 (m, 2H), 7.13–7.09 (m, 2H), 2.65–2.56 (m, 4H), 2.42 (s, 3H), 1.82–1.73 (m, 2H), 0.99 (br s, 1H); **^{13}C NMR (100 MHz, CDCl$_3$)** δ 140.8, 131.6, 129.8 (2C), 128.5 (2C), 51.6, 36.7, 33.1, 31.6; **IR (neat) ν_{max}:** 2936, 2860, 1542, 1491, 1408, 1383, 1308, 1251, 1093, 1015, 833, 801; **HRMS (ESI+):** exact mass calculated for $[M+H]^+$ ($C_{10}H_{15}{}^{35}ClN$) requires *m/z* 184.0888, found *m/z* 184.0880.

***N*-Methyl-3-(naphthalen-2-yl)propan-1-amine (3p)**

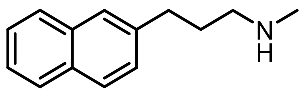

Prepared according to procedure A, method B with 4 equiv. *N,N,N′,N′*-tetramethyldiaminomethane; (55 mg, 55% yield); **^{1}H NMR (400 MHz, CDCl$_3$)** δ 7.83–7.74 (m, 3H), 7.63 (s, 1H), 7.48–7.38 (m, 2H), 7.36–7.32 (m, 1H), 2.87–2.80 (m, 2H), 2.69–2.62 (m, 2H), 2.44 (s, 3H), 1.97–1.86 (m, 2H), 1.10 (br s, 1H); **^{13}C NMR (100 MHz, CDCl$_3$)** δ 139.9, 133.8, 132.1, 128.0, 127.7, 127.5, 127.4, 126.5, 126.0, 125.2, 51.8, 36.7, 33.9, 31.6; **IR (neat) ν_{max}:** 2930, 2855, 1538, 1506, 1476, 1381, 1307, 816, 747; **HRMS (ESI+):** exact mass calculated for $[M+H]^+$ ($C_{14}H_{18}N$) requires *m/z* 200.1434, found *m/z* 200.1438.

***N*-Benzyl-3-phenylpropan-1-amine (3q)**

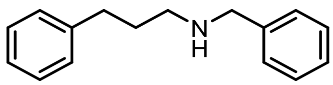

Prepared according to procedure A, method B with 4 equiv. *N,N,N′,N′*-tetrabenzyldiaminomethane, (47 mg, 42% yield). **^{1}H NMR (400 MHz, CDCl$_3$)** δ 7.27–7.26 (m, 3H), 7.22–7.20 (m, 4H), 7.14–7.11 (m, 3H), 3.73 (s, 2H), 2.62 (m, 2H), 1.86–1.73 (m, 2H,), 1.49 (br s, 1H); **^{13}C NMR (150 MHz, CDCl$_3$)** δ 142.3, 140.5, 128.5 (2C), 128.5 (2C), 128.5 (2C), 128.5 (2C), 128.3, 127.1, 125.9, 54.1, 49.0, 33.8, 31.8; **IR (neat) ν_{max}:** 3026, 2958, 2919, 2853, 1494, 1453, 1275, 1261, 749, 698; **HRMS (ESI+):** exact mass calculated for $[M+H]^+$ ($C_{16}H_{20}N$) requires *m/z* = 226.1590, found *m/z* 226.1590.

Alternative procedure for the synthesis with Eschenmoser's chloride: A round-bottom flask protected from light was charged with *N*-benzyl-*N*-methylene-1-phenylmethanaminium chloride (4 equiv.) and a magnetic stir-bar under argon-atmosphere, and was cooled to 0 °C. After this, the styrene (1 equiv.) and 1,1,1,3,3,3-hexafluoroisopropanol (HFIP, 0.6 M with respect to the alkene) were added. After completed addition of the solvent, the flask was sealed and placed in an oil bath at 75 °C. The reaction was vigorously stirred at this temperature for 2 h, after which it was allowed to cool to room temperature. Subsequently, aqueous sodium hydroxide (1 M) was added until the reaction mixture reached pH 12. The resulting biphasic mixture was separated and the aqueous phase was extracted with dichloromethane

(3 x 200 mL/mmol). The combined organic phases were then dried over anhydrous potassium carbonate and filtered. The filtrate was concentrated under reduced pressure to afford the crude product, which was purified by flash column chromatography on silica gel (dichloromethane/MeOH/NH_4OH 19:1:0.15) to afford the analytically pure desired product (74 mg, 66% yield).

***N*-(3-Phenylpropyl)butan-1-amine (3r)**

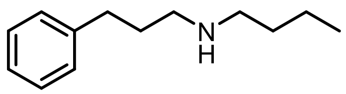

Prepared according to procedure A, method B with 4 equiv. *N,N,N′,N′*-tetrabutyldiaminomethane; (82 mg, 86% yield); **^{1}H NMR (400 MHz, $CDCl_3$)** δ 7.30–7.23 (m, 2H), 7.22–7.15 (m, 3H), 2.69–2.62 (m, 4H), 2.62–2.57 (m, 2H), 1.87–1.78 (m, 2H), 1.50–1.42 (m, 2H), 1.38–1.29 (m, 2H), 1.00–0.85 (m, 4H),; **^{13}C NMR (100 MHz, $CDCl_3$)** δ 142.4, 128.5 (2C), 128.4 (2C), 125.9, 49.9, 49.8, 33.9, 32.5, 32.0, 20.7, 14.2; **IR (neat) ν_{max}:** 2956, 2927, 2858, 1495, 1455, 1129, 699; **HRMS (ESI+):** exact mass calculated for $[M+H]^+$ ($C_{13}H_{22}N$) requires *m/z* 192.1747, found *m/z* 192.1752.

***N*-(3-Phenylpropyl)prop-2-en-1-amine (3s)**

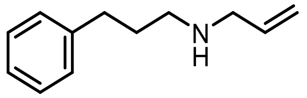

Prepared according to procedure A, method B with 4 equiv. *N,N,N′,N′*-tetraallyldiaminomethane, (60 mg, 69% yield). **^{1}H NMR (400 MHz, $CDCl_3$)** δ 7.30–7.26 (m, 2H), 7.20–7.16 (m, 3H), 5.91 (ddt, *J* = 16.3, 10.3, 6.0 Hz, 1H), 5.19–5.07 (m, 2H), 3.25 (m, 2H), 2.69–2.64 (m, 4H), 1.84 (m, 2H), 1.34 (br s, 1H); **^{13}C NMR (100 MHz, $CDCl_3$)** δ 142.3, 137.0, 128.5 (2C), 128.5 (2C), 125.9, 116.0, 52.6, 49.1, 33.8, 31.9; **IR (neat) ν_{max}:** 2926, 2853, 1675, 1643, 1452, 1153, 1118, 917, 749, 700; **HRMS (ESI+):** exact mass calculated for $[M+H]^+$ ($C_{12}H_{18}N$) requires *m/z* 176.1434, found *m/z* 176.1435.

***N*-Isobutyl-2-methyl-3-phenylpropan-1-amine (3t)**

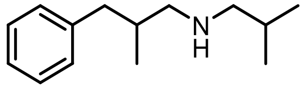

Prepared according to procedure A, method B with 4 equiv. *N,N,N′,N′*-tetramethyldiaminomethane from *cis*-β-methylstyrene–(62.5 mg, 61% yield), *trans*-β-methylstyrene–(61.5 mg, 60% yield); **^{1}H NMR (400 MHz, $CDCl_3$)** δ 7.30–7.24 (m, 2H), 7.21–7.14 (m, 3H), 2.73 (dd, *J* = 13.4, 6.0 Hz, 1H), 2.55 (dd, *J* = 11.7, 6.0 Hz, 1H), 2.47–2.35 (m, 4H), 2.00–1.88 (m, 1H), 1.78–1.66 (m, 1H), 1.08 (br s, 1H), 0.91–0.86 (m, 9H); **^{13}C NMR (100 MHz, $CDCl_3$)** δ 141.3, 129.3 (2C), 128.3 (2C), 125.9, 58.5, 56.4, 41.8, 35.4, 28.4, 20.8, 20.8, 18.1; **IR (neat) ν_{max}:** 3027, 2954, 2927, 1495, 1457, 1094, 739, 700; **HRMS (ESI+):** exact mass calculated for $[M+H]^+$ ($C_{14}H_{24}N$) requires *m/z* 206.1903, found *m/z* 206.1904.

N,2-Dimethyl-3-phenylpropan-1-amine (3u)

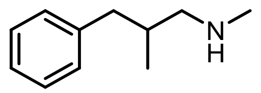

Prepared according to procedure A, method B with 4 equiv. *N,N,N',N'*-tetramethyldiaminomethane from *cis*-β-methylstyrene; (32 mg, 39% yield); **^{1}H NMR (400 MHz, CDCl$_3$)** δ 7.30–7.24 (m, 2H), 7.21–7.14 (m, 3H), 2.73 (dd, *J* = 13.4, 6.0 Hz, 1H), 2.53 (dd, *J* = 11.6, 6.0 Hz, 1H), 2.45–2.35 (m, 5H), 2.00–1.87 (m, 1H), 1.01 (br s, 1H), 0.89 (d, *J* = 6.7 Hz, 3H); **^{13}C NMR (100 MHz, CDCl$_3$)** δ 141.2, 129.3 (2C), 128.3 (2C), 125.9, 58.5, 41.7, 36.9, 35.4, 18.1; **IR (neat) ν_{max}:** 3174, 2960, 2924, 1668, 1630, 1545, 1454, 1384, 740, 701; **HRMS (ESI+):** exact mass calculated for $[M+H]^+$ ($C_{11}H_{18}N$) requires *m/z* 164.1434, found *m/z* 164.1434.

12-(Methylamino)dodecyl acetate (3v)

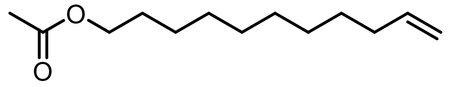

Prepared according to procedure A, method B with 4 equiv. *N,N,N',N'*-tetramethyldiaminomethane; (120 mg, 93% yield); **^{1}H NMR (600 MHz, CDCl$_3$)** δ 4.04 (t, *J* = 6.8 Hz, 2H), 2.55 (t, *J* = 7.2 Hz, 2H), 2.42 (s, 3H), 2.04 (s, 3H), 1.64–1.57 (m, 2H), 1.50–1.43 (m, 2H), 1.36–1.20 (m, 17H); **^{13}C NMR (150 MHz, CDCl$_3$)** δ 171.4, 64.8, 52.4, 36.7, 30.1, 29.7 (2C), 29.7, 29.7, 29.6, 29.4, 28.7, 27.5, 26.0, 21.2; **IR (neat) ν_{max}:** 2922, 2852, 1738, 1465, 1386, 1366, 1235, 1039; **HRMS (ESI+):** exact mass calculated for $[M+H]^+$ ($C_{15}H_{32}NO_2$) requires *m/z* 258.2428, found *m/z* 258.2423.

Methyl 7-(methylamino)heptanoate (3w)

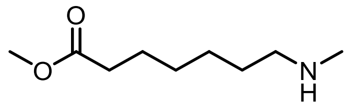

Prepared according to procedure A, method B with 4 equiv. *N,N,N',N'*-tetramethyldiaminomethane; (55 mg, 64% yield); **^{1}H NMR (400 MHz, CDCl$_3$)** δ 3.65 (s, 3H), 2.54 (t, *J* = 7.1 Hz, 2H), 2.41 (s, 3H), 2.29 (t, *J* = 7.6 Hz, 2H), 1.66–1.57 (m, 2H), 1.51–1.42 (m, 2H), 1.37–1.29 (m, 4H), 1.06 (br s, 1H); **^{13}C NMR (100 MHz, CDCl$_3$)** δ 174.3, 52.2, 51.5, 36.7, 34.1, 29.9, 29.2, 27.1, 25.0; **IR (neat) ν_{max}:** 3359, 3199, 1664, 1557, 1396, 636; **HRMS (ESI+):** exact mass calculated for $[M+H]^+$ ($C_9H_{20}NO_2$) requires *m/z* 174.1489, found *m/z* 174.1486.

Diethyl (9-(methylamino)nonyl)phosphonate (3x)

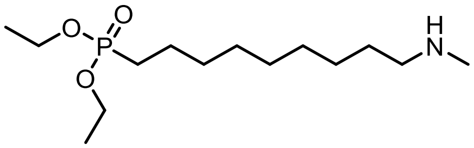

Prepared according to procedure A, method B with 4 equiv. *N,N,N',N'*-tetramethylldiaminomethane, (117 mg, 80% yield); **^{1}H NMR (600 MHz, $CDCl_3$)** δ 4.11–4.03 (m, 4H), 2.54 (t, *J* = 7.2 Hz, 2H), 2.42 (s, 3H), 1.73–1.66 (m, 2H), 1.61–1.55 (m, 2H), 1.52 (br s, 1H), 1.47–1.45 (m, 2H), 1.36–1.34 (m, 2H), 1.30 (m, 14H); **^{13}C NMR (150 MHz, $CDCl_3$)** δ 61.5 (d, *J* = 6.5 Hz, 2C), 52.3, 36.6, 30.7 (d, *J* = 17.0), 30.0, 29.6, 29.4, 29.2, 27.4, 25.8 (d, *J* = 140.3), 22.5 (d, *J* = 5.2 Hz), 16.6 (d, *J* = 6.0 Hz, 2C); **IR (neat) ν_{max}:** 3427, 2925, 2853, 1465, 1386, 1235, 1054, 1024, 958; **HRMS (ESI+):** exact mass calculated for $[M+H]^+$ ($C_{14}H_{33}NO_3P$) requires *m/z* 294.2193, found *m/z* 294.2192.

12-(Methylamino)-*N*-(pyridin-4-yl)dodecanamide (3y)

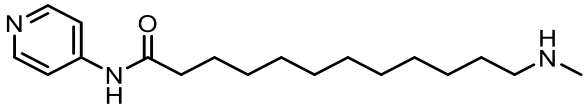

Prepared according to procedure A, method B with 4 equiv. *N,N,N',N'*-tetramethyldiaminomethane, (110 mg, 72% yield); **^{1}H NMR (400 MHz, $CDCl_3$)** δ 8.48 (dd, *J* = 4.9, 1.4 Hz, 2H), 7.71 (br s, 1H), 7.49–7.48 (m, 2H), 3.48 (s, 1H), 2.57 (t, *J* = 7.2 Hz, 2H), 2.44 (s, 3H), 2.41–2.32 (m, 2H), 1.75–1.68 (m, 4H), 1.50–1.47 (m, 2H), 1.34–1.27 (m, 12H); **^{13}C NMR (100 MHz, $CDCl_3$)** δ 172.3, 150.8 (2C), 145.3, 113.6 (2C), 52.3, 37.9, 36.5, 29.9, 29.6, 29.5 (2C), 29.4, 29.3, 29.3, 27.4, 25.4; **IR (neat) ν_{max}:** 2925, 2853, 1703, 1594, 1522, 1329, 1296, 1210, 832; **HRMS (ESI+):** exact mass calculated for $[M+H]^+$ ($C_{18}H_{32}N_3O$) requires *m/z* 306.2540, found *m/z* 306.2539.

13-(Methylamino)tridecanenitrile (3z)

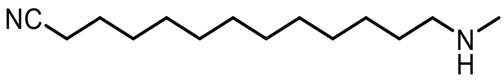

Prepared according to procedure A, method B with 4 equiv. *N,N,N′,N′*-tetramethyldiaminomethane; (94 mg, 84% yield); **^{1}H NMR (600 MHz, $CDCl_3$)** δ 2.55 (t, *J* = 7.1 Hz, 2H), 2.42 (s, 3H), 2.32 (t, *J* = 7.1 Hz, 2H), 1.64 (app quin, *J* = 7.5 Hz, 2H), 1.49–1.40 (m, 4H), 1.33–1.24 (m, 14H), 1.19 (br s, 1H); **^{13}C NMR (150 MHz, $CDCl_3$)** δ 120.0, 52.4, 36.7, 30.1, 29.7, 29.7, 29.6, 29.6, 29.4, 28.9, 28.8, 27.5, 25.5, 17.3; **IR (neat) ν_{max}:** 2922, 2852, 2243, 1465, 1426, 1382, 1308, 722; **HRMS (ESI+):** exact mass calculated for $[M+H]^+$ ($C_{14}H_{29}N_2$) requires *m/z* 225.2325, found *m/z* 225.2324.

***N*-Benzyl-9-bromononan-1-amine (3aa)**

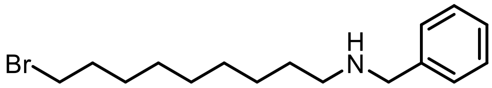

Prepared according to procedure A, method B with 1.5 equiv. *N,N,N′,N′*-tetrabenzyldiaminomethane, (62 mg, 40% yield); **^{1}H NMR (400 MHz, $CDCl_3$)** δ 7.33 (app d, *J* = 4.1 Hz, 4H), 7.27–7.24 (m, 1H), 3.80 (s, 2H), 3.40 (t, *J* = 6.9 Hz, 2H), 2.63 (t, *J* = 7.3 Hz, 2H), 1.88–1.81 (m, 3H), 1.54–1.51 (m, 2H), 1.43–1.40 (m, 2H), 1.29–1.26 (m, 8H); **^{13}C NMR (100 MHz, $CDCl_3$)** δ 138.7, 128.6 (2C), 128.4 (2C), 127.1, 54.1, 49.5, 34.1, 33.0, 30.0, 29.5, 29.5, 28.8, 28.3, 27.4; **IR (neat) ν_{max}:** 2920, 2851, 2795, 1457, 1435, 734, 696; **HRMS (ESI+):** exact mass calculated for $[M+H]^+$ ($C_{16}H_{27}NBr$) requires *m/z* 312.1321, found *m/z* 312.1319.

12-(Methylamino)dodecan-1-ol (3ab)

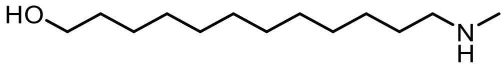

Prepared according to procedure A, method B with 4 equiv. *N,N,N′,N′*-tetramethyldiaminomethane; (88 mg, 82% yield; 20 mmol: 2.115 g, 71% yield); **^{1}H NMR (400 MHz, $CDCl_3$)** δ 3.63 (t, *J* = 6.6 Hz, 2H), 2.55 (t, *J* = 7.2 Hz, 2H), 2.42 (s, 3H), 1.60–1.51 (m, 2H), 1.51–1.42 (m, 2H), 1.38–1.24 (m, 18H); **^{13}C NMR (100 MHz, $CDCl_3$)** δ 63.2, 52.4, 36.7, 33.0, 30.1, 29.7 (3C), 29.7 (2C), 29.5, 27.5, 25.9; **IR (neat) ν_{max}:** 3304, 2918, 2850, 1680, 1468, 1387, 1202, 1180, 1134, 1059; **HRMS (ESI+):** exact mass calculated for $[M+H]^+$ ($C_{13}H_{30}NO$) requires *m/z* 216.2322, found *m/z* 216.2314.

***N*^{1}-Butyl-*N*^{12}-methyldodecane-1,12-diamine (3ac)**

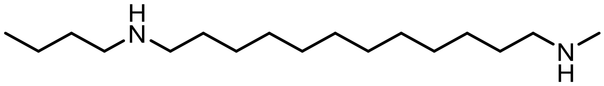

Prepared according to procedure A, method B with 4 equiv. *N,N,N′,N′*-tetramethyldiaminomethane; (81 mg, 60% yield); **^{1}H NMR (400 MHz, $CDCl_3$)** δ 2.61–2.52 (m, 6H), 2.42 (s, 3H), 1.51–1.41 (m, 6H), 1.37–1.24 (m, 18H), 1.00–0.85 (m, 5H); **^{13}C NMR (100 MHz, $CDCl_3$)** δ 52.4, 50.4, 50.0, 36.8, 32.5, 30.4, 30.1, 29.7

(7C), 27.6, 27.5, 20.7, 14.2; **IR (neat) ν_{max}:** 2921, 2850, 2811, 1466, 1377, 1128, 733; **HRMS (ESI+):** exact mass calculated for $[M+H]^+$ ($C_{17}H_{39}N_2$) requires *m/z* 271.3108, found *m/z* 271.3108.

***N*^{1},*N*^{1}-Diethyl-*N*^{12}-methyldodecane-1,12-diamine (3ad)**

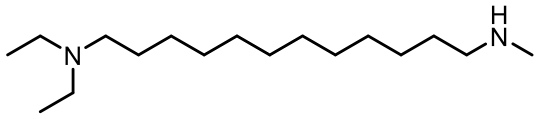

Prepared according to procedure A, method B with 4 equiv. *N,N,N',N'*-tetramethyldiaminomethane; (101 mg, 75% yield); **^{1}H NMR (400 MHz, $CDCl_3$)** δ 2.55 (m, 6H), 2.43 (s, 3H), 2.44–2.40 (m, 2H), 1.68 (br s, 1H), 1.47–1.44 (m, 4H), 1.26 (s, 16H), 1.03 (t, *J* = 7.2 Hz, 6H); **^{13}C NMR (150 MHz, $CDCl_3$)** δ 53.0, 52.3, 46.9 (2C), 36.6, 30.0, 29.8, 29.7 (2C), 29.7 (2C), 27.8 (2C), 27.5, 26.9, 11.7 (2C); **IR (neat) ν_{max}:** 2967, 2922, 2851, 1466, 1381, 1201, 1130, 721; **HRMS (ESI+):** exact mass calculated for $[M+H]^+$ ($C_{17}H_{39}N_2O$) requires *m/z* 271.3108, found *m/z* 271.3110.

(*E*)-*N*-Benzylundec-2-en-1-amine (6a)

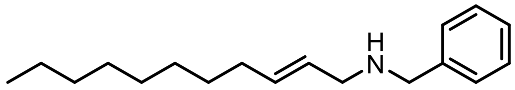

Prepared according to procedure B, method B with 1.5 equiv. *N,N,N',N'*-tetrabenzyldiaminomethane; (69 mg, 53% yield); **^{1}H NMR (600 MHz, $CDCl_3$)** δ 7.32 (d, *J* = 4.5 Hz, 3H), 7.26–7.24 (m, 2H), 5.62–5.57 (m, 1H), 5.53 (dt, *J* = 15.3, 6.0 Hz, 1H), 3.78 (s, 2H), 3.21 (d, *J* = 6.0 Hz, 2H), 2.02 (app q, *J* = 6.9 Hz, 2H), 1.53 (br s, 1H), 1.37-33 (m, 2H,), 1.30–1.26 (m, 10H), 0.87 (t, *J* = 7.0 Hz, 3H); **^{13}C NMR (150 MHz, $CDCl_3$)** δ 140.5 (C), 133.3, 128.5 (2C), 128.4 (2C), 128.1, 127.0, 53.4, 51.3, 32.6, 32.0, 29.6, 29.5, 29.4, 29.3, 22.8, 14.3; **IR (neat) ν_{max}:** 3027, 2955, 2852, 1454, 969, 731, 698; **HRMS (ESI+):** exact mass calculated for $[M+H]^+$ ($C_{18}H_{30}N$) requires *m/z* 260.2373, found *m/z* 260.2369.

(*E*)-3-Cyclohexyl-*N*-methylprop-2-en-1-amine (6b)

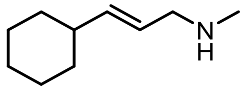

Prepared according to procedure A, method B with 4 equiv. *N,N,N',N'*-tetramethyldiaminomethane; (50 mg, 65% yield); **^{1}H NMR (400 MHz, $CDCl_3$)** δ 5.54 (dd, *J* = 15.5, 6.3 Hz, 1H), 5.49–5.42 (m, 1H), 3.14 (d, *J* = 5.9 Hz, 2H), 2.41 (s, 3H), 1.94 (ddd, *J* = 14.1, 11.2, 3.0 Hz, 1H), 1.72 (dd, *J* = 9.2, 6.7 Hz, 4H), 1.66–1.62 (m, 1H), 1.33–1.01 (m, 6H); **^{13}C NMR (100 MHz, $CDCl_3$)** δ 138.9, 125.5, 54.0, 40.6, 35.9, 33.2 (2C), 26.4 (2C), 26.2; **IR (neat) ν_{max}:** 2921, 2849, 2789, 1447, 1379, 1315, 1286, 1254, 1031, 970; **HRMS (ESI+):** exact mass calculated for $[M+H]^+$ ($C_{10}H_{20}N$) requires *m/z* 154.1590, found *m/z* 154.1590.

(*E*)-*N*-Benzyl-3-(cyclohex-1-en-1-yl)prop-2-en-1-amine (6c)

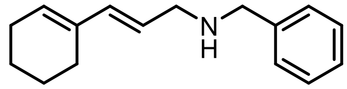

Prepared according to procedure C, method B with 1.5 equiv. *N,N,N′,N′*-tetrabenzyldiaminomethane; (77 mg, 68% yield); **^{1}H NMR (400 MHz, $CDCl_3$)** δ 7.35–7.30 (m, 4H), 7.28–7.23 (m, 1H), 6.17 (d, *J* = 15.7 Hz, 1H), 5.71 (br s, 1H), 5.69–5.59 (m, 1H), 3.80 (s, 2H), 3.32 (d, *J* = 6.3 Hz, 2H), 2.13-2.12 (m, 4H), 2.03 (s, 1H), 1.66 (ddd, *J* = 6.4, 4.6, 2.9 Hz, 2H), 1.63–1.54 (m, 2H); **^{13}C NMR (100 MHz, $CDCl_3$)** δ 140.2, 135.7, 135.5, 129.2, 128.6 (2C), 128.4 (2C), 127.1, 123.8, 53.3, 51.4, 26.0, 24.7, 22.7, 22.6; **IR (neat) ν_{max}:** 3025, 2922, 2855, 1493, 1450, 1072, 964, 731, 696; **HRMS (ESI+):** exact mass calculated for $[M+H]^+$ ($C_{16}H_{22}N$) requires *m/z* 228.1747, found *m/z* 228.1744.

(*E*)-*N*-Benzyl-3-cyclopropylprop-2-en-1-amine (6d)

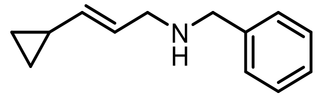

Prepared according to procedure C, method B with 1.5 equiv. *N,N,N′,N′*-tetrabenzyldiaminomethane; (80 mg, 85% yield); **^{1}H NMR (600 MHz, $CDCl_3$)** δ 7.32–7.31 (m, 4H), 7.26–7.24 (m, 1H), 5.64 (dt, *J* = 15.0, 6.4 Hz, 1H), 5.14 (dd, *J* = 15.3, 8.7 Hz, 1H), 3.78 (s, 2H), 3.21 (d, *J* = 6.4 Hz, 2H), 1.40–1.38 (m, 1H), 1.25 (bs, 1H), 0.70–0.67 (m, 2H), 0.36–0.34 (m, 2H); **^{13}C NMR (150 MHz, $CDCl_3$)** δ 140.5, 136.6, 128.5 (2C), 128.3 (2C), 127.0, 126.0, 53.4, 51.2, 13.6, 6.7 (2C); **IR (neat) ν_{max}:** 3083, 3064, 3025, 3005, 2958, 2924, 2853, 2807, 1495, 1453, 963, 735, 698; **HRMS (ESI+):** exact mass calculated for $[M+H]^+$ ($C_{13}H_{18}N$) requires *m/z* 188.1434, found *m/z* 188.1433.

(*E*)-*N*-Methyl-2-propylhex-2-en-1-amine (6e)

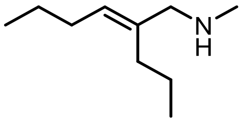

Prepared according to procedure A, method B with 4 equiv. *N,N,N′,N′*-tetramethyldiaminomethane; (32 mg, 41% yield); **^{1}H NMR (600 MHz, $CDCl_3$)** δ 5.32–5.29 (m, 1H), 3.10 (s, 2H), 2.38 (s, 3H), 2.04–1.99 (m, 4H), 1.67 (s, 1H), 1.38 (dt, *J* = 23.9, 7.5 Hz, 4H), 0.90 (t, *J* = 7.3 Hz, 6H); **^{13}C NMR (150 MHz, $CDCl_3$)** δ 137.3, 127.1, 57.6, 35.9, 31.1, 29.8, 23.2, 21.9, 14.3, 14.1; **IR (neat) ν_{max}:** 3446, 2965, 2918, 1455, 1396, 764, 703; **HRMS (ESI+):** exact mass calculated for $[M+H]^+$ ($C_{10}H_{22}N$) requires *m/z* 156.1747, found *m/z* 156.1743.

(*E*)-*N*-Methyl-3-phenylprop-2-en-1-amine (6f)

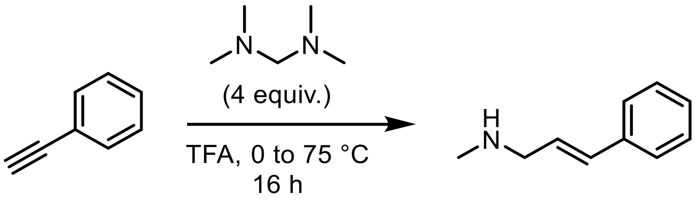

Prepared according to procedure A, method B with 4 equiv. *N,N,N′,N′*-tetramethyldiaminomethane; (42 mg, 57% yield). The crude reaction mixture was purified through preparative HPLC (column Waters, X select CSH prep C18, 5 µm, 30x150mm, Acetonitrile/ 1mM NH_4HCO_3 solution in water from 50% to 85%, in 20 minutes) to afford the pure product. **^{1}H NMR (400 MHz, $CDCl_3$)** δ 7.38 (dd, *J* = 8.3, 1.2 Hz, 2H), 7.32–7.29 (m, 2H), 7.24–7.20 (m, 1H), 6.54 (d, *J* = 15.9 Hz, 1H), 6.29 (dt, *J* = 15.9, 6.3 Hz, 1H), 3.39 (d, *J* = 5.8 Hz, 2H), 2.48 (s, 3H), 1.51 (s, 1H); **^{13}C NMR (100 MHz, $CDCl_3$)** δ 137.3, 131.6, 128.7 (2C), 128.4, 127.5, 126.4 (2C), 54.0, 36.0; **IR (neat) ν_{max}:** 3046,3325, 3082, 3059, 3026, 2938, 2848, 2790, 1598, 1552, 1493, 1449, 1380, 1356, 1287, 1258, 968, 744; **HRMS (ESI+):** exact mass calculated for $[M+H]^+$ ($C_{10}H_{14}N$) requires *m/z* 148.1121, found *m/z* 148.1121.

(*E*)-*N*-Methyl-3-(*p*-tolyl)prop-2-en-1-amine (6g)

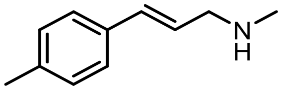

Prepared according to procedure A, method B with 4 equiv. *N,N,N′,N′*-tetramethyldiaminomethane; (41 mg, 51% yield); **^{1}H NMR (400 MHz, $CDCl_3$)** δ 7.27 (d, *J* = 7.9 Hz, 2H), 7.11 (d, *J* = 7.9 Hz, 2H), 6.50 (d, *J* = 15.9 Hz, 1H), 6.24 (dt, *J* = 15.9, 6.3 Hz, 1H), 3.37 (dd, *J* = 6.3, 1.4 Hz, 2H), 2.48 (s, 3H), 2.33 (s, 3H), 1.40 (br s, 1H); **^{13}C NMR (100 MHz, $CDCl_3$)** δ 137.3, 134.5, 131.4, 129.4 (2C), 127.4, 126.3 (2C), 54.0, 36.2, 21.3; **IR (neat) ν_{max}:** 3045, 2922, 2853, 1669, 1513, 1451, 1382, 970, 766, 749; **HRMS (ESI+):** exact mass calculated for $[M+H]^+$ ($C_{11}H_{16}N$) requires *m/z* 162.1277, found *m/z* 162.1274.

(*E*)-3-(4-Fluorophenyl)-*N*-methylprop-2-en-1-amine (6h)

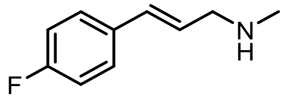

Prepared according to procedure A, method B with 4 equiv. *N,N,N′,N′*-tetramethyldiaminomethane; (26 mg, 31% yield); **^{1}H NMR (700 MHz, $CDCl_3$)** δ 7.34–7.32 (m, 2H), 6.99 (t, *J* = 8.7 Hz, 2H), 6.49 (d, *J* = 15.9 Hz, 1H), 6.20 (dt, *J* = 15.9, 6.3 Hz, 1H), 3.36 (dd, *J* = 6.3, 0.9 Hz, 2H), 2.48 (s, 3H), 1.25 (br s, 1H); **^{13}C NMR (175 MHz, $CDCl_3$)** δ 162.3 (d, *J* = 246 Hz), 133.5 (d, *J* = 3.3 Hz), 130.2, 128.3 (d, *J* = 2.1 Hz) , 127.8 (d, *J* = 7.9 Hz, 2C), 115.6 (*J* = 21.5 Hz, 2C), 53.9, 36.2; **IR (neat) ν_{max}:** 2360, 2177, 2045, 1509, 1227, 763, 749; **HRMS (ESI+):** exact mass calculated for $[M+H]^+$ ($C_{10}H_{13}N$) requires *m/z* 166.1027, found *m/z* 166.1021.

(*E*)-2-(4-(3-(Methylamino)prop-1-en-1-yl)phenyl)acetonitrile (6i)

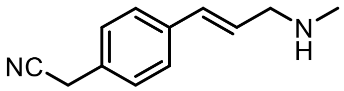

Prepared according to procedure B, method B with 4 equiv. *N,N,N′,N′*-tetramethyldiaminomethane; (34 mg, 37% yield; 20 mmol: 1.373 g, 35% yield); **^{1}H NMR (600 MHz, $CDCl_3$)** δ 7.38 (d, *J* = 8.2 Hz, 2H), 7.27–7.26 (m, 2H), 6.52 (d, *J* = 15.9 Hz, 1H), 6.31 (dt, *J* = 15.9, 6.2 Hz, 1H), 3.73 (s, 2H), 3.39 (dd, *J* = 6.3, 1.4 Hz, 2H), 2.48 (s, 3H), 1.63 (s, 1H); **^{13}C NMR (150 MHz, $CDCl_3$)** δ 137.0, 130.3, 129.2, 128.6, 128.1 (2C), 126.9 (2C), 117.8, 53.7, 36.0, 23.3; **IR (neat) ν_{max}:** 2981, 2898, 1670, 1512, 1361, 1275, 1261, 972, 764, 750; **HRMS (ESI+):** exact mass calculated for $[M+H]^+$ ($C_{12}H_{15}N$) requires *m/z* 187.1230, found *m/z* 187.1224.

(*E*)-*N*,2-Dimethyl-3-phenylprop-2-en-1-amine (6j)

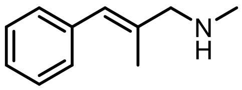

Prepared according to procedure A, method B with 4 equiv. *N,N,N′,N′*-tetramethyldiaminomethane; (39 mg, 48% yield); **^{1}H NMR (600 MHz, $CDCl_3$)** δ 7.33 (t, *J* = 7.6 Hz, 2H), 7.27 (d, *J* = 7.3 Hz, 2H), 7.20 (t, *J* = 7.3 Hz, 1H), 6.44 (s, 1H), 3.30 (s, 2H), 2.47 (s, 3H), 1.90 (d, *J* = 0.9 Hz, 3H), 1.57 (br s, 1H); **^{13}C NMR (150 MHz, $CDCl_3$)** δ 138.1, 136.8, 129.0 (2C), 128.2 (2C), 126.3, 126.0, 60.4, 35.9, 16.7; **IR (neat) ν_{max}:** 3024, 2925, 2853, 2794, 2770, 2714, 1680, 1491, 1448, 1387, 1357, 1037, 748, 699; **HRMS (ESI+):** exact mass calculated for $[M+H]^+$ ($C_{11}H_{16}N$) requires *m/z* 162.1277, found *m/z* 162.1277.

Methyl (*E*)-7-(benzylamino)hept-5-enoate (6k)

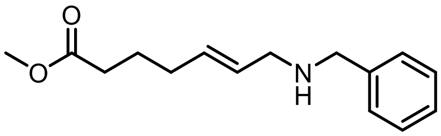

Prepared according to procedure A, method B with with 1.5 equiv. *N,N,N′,N′*-tetrabenzyldiaminomethane; (51 mg, 41% yield); **^{1}H NMR (400 MHz, CDCl3)** δ 7.26 (d, *J* = 4.5 Hz, 4H), 7.19–7.17 (m, 1H), 5.53–5.51 (m, 2H), 3.73 (s, 2H), 3.61 (s, 3H), 3.17 (dd, *J* = 3.7, 1.0 Hz, 2H), 2.26 (t, *J* = 7.5 Hz, 2H), 2.03–2.01 (m, 2H), 1.82 (br s, 1H), 1.67 (p, *J* = 7.5 Hz, 2H); **^{13}C NMR (100 MHz, $CDCl_3$)** δ 174.2, 140.3, 131.7, 129.4, 128.5 (2C), 128.4 (2C), 127.1, 53.4, 51.6, 51.1, 33.5, 31.8, 24.6; **IR (neat) ν_{max}:** 2926, 2797, 1734, 1602, 1451, 1436, 1154, 1121, 970, 734, 698; **HRMS (ESI+):** exact mass calculated for $[M+H]^+$ ($C_{15}H_{22}NO_2$) requires *m/z* 248.1645, found *m/z* 248.1644.

(*E*)-8-(Methylamino)oct-6-enenitrile (6l)

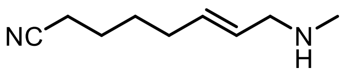

Prepared according to procedure A, method B with 4 equiv. *N,N,N',N'*-tetramethyldiaminomethane; (73 mg, 97% yield); **^{1}H NMR (400 MHz, CDCl$_3$)** δ 5.54 (td, *J* = 5.1, 3.0 Hz, 2H), 3.16–3.14 (m, 2H), 2.41 (s, 3H), 2.33 (t, *J* = 7.0 Hz, 2H), 2.07 (dd, *J* = 12.2, 7.2 Hz, 2H), 1.70–1.63 (m, 2H), 1.57–1.50 (m, 2H), 1.32 (br s, 1H); **^{13}C NMR (100 MHz, CDCl$_3$)** δ 131.4, 129.4, 119.8, 53.7, 36.0, 31.5, 28.3, 24.9, 17.2; **IR (neat) ν_{max}:** 2932, 2857, 2790, 1541, 1460, 1381, 1262, 1032, 974, 764, 750; **HRMS (ESI+):** exact mass calculated for $[M+H]^+$ ($C_9H_{17}N_2$) requires *m/z* 153.1386, found *m/z* 153.1386.

(*E*)-11-(Methylamino)undec-9-en-1-ol (6m)

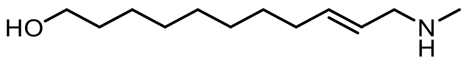

Prepared according to procedure B, method B with 4 equiv. *N,N,N',N'*-tetramethyldiaminomethane, stirred for 5 h at 75 °C instead of 16 h; (58 mg, 59% yield); **^{1}H NMR (600 MHz, CDCl$_3$)** δ 5.60–5.55 (m, 1H), 5.51–5.47 (m, 1H), 3.62 (t, *J* = 6.6 Hz, 2H), 3.14 (d, *J* = 6.1 Hz, 2H), 2.40 (s, 3H), 2.01 (q, *J* = 6.8 Hz, 2H), 1.63–1.53 (m, 4H), 1.35–1.24 (m, 10H); **^{13}C NMR (150 MHz, CDCl$_3$)** δ 133.2, 128.0, 63.1, 53.8, 35.9, 32.9, 32.4, 29.4, 29.3, 29.1, 25.8; **IR (neat) ν_{max}:** 3405, 3368, 2925, 2854, 1541, 1460, 1382, 1260, 1059, 971; **HRMS (ESI+):** exact mass calculated for $[M+H]^+$ ($C_{12}H_{26}NO$) requires *m/z* 200.2009, found *m/z* 200.2009.

(*E*)-*N*-Methyl-4-(triisopropylsilyl)but-2-en-1-amine (6n)

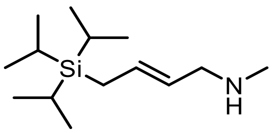

Prepared according to procedure A, method B with 4 equiv. *N,N,N',N'*-tetramethyldiaminomethane; ***stirred at room temperature instead of 75 °C***, (41 mg, 34% yield); **^{1}H NMR (400 MHz, CDCl$_3$)** δ 5.68–5.60 (m, 1H), 5.46–5.38 (m, 1H), 3.12 (dd, *J* = 6.5, 0.8 Hz, 2H), 2.40 (s, 3H), 1.59 (dd, *J* = 8.0, 1.1 Hz, 2H), 1.15 (br s, 1H), 1.05 (s, 9H); **^{13}C NMR (100 MHz, CDCl$_3$)** δ 130.1, 126.9, 54.4, 36.0, 18.8 (6C), 15.5, 11.2 (3C); **IR (neat) ν_{max}:** 2940, 2889, 2864, 1461, 1381, 1253, 1154, 966, 809, 749, 701, 658; **HRMS (ESI+):** exact mass calculated for $[M+H]^+$ ($C_{14}H_{32}N$) requires *m/z* 242.2294, found *m/z* 242.2295.

2.6 C–C Bond Formation on the Reaction Intermediate

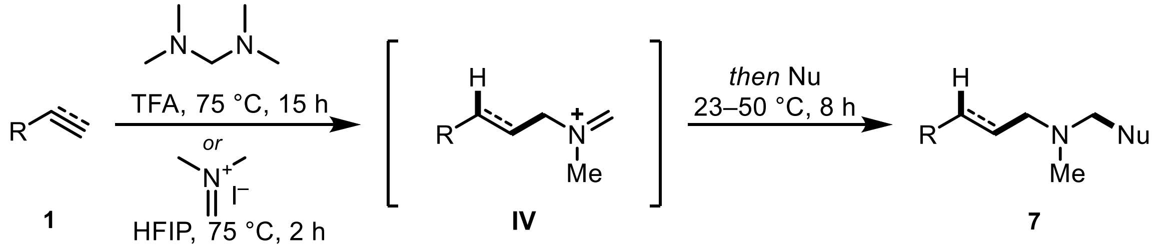

4-(Methyl(3-phenylpropyl)amino)butan-2-one (7a)

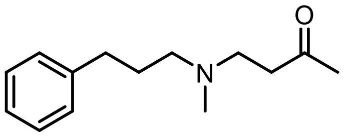

Following procedure A, (method B) styrene was subjected to hydroaminomethylation-conditions using *N,N,N′,N′*-tetramethyldiaminomethane. After the hydroaminomethylation reaction and cooling to 23 °C, acetone (55 equiv.) was added directly to the reaction mixture and the resulting solution was stirred at 23 °C for 8 h. After this time, volatile components were removed under reduced pressure and the residue was dissolved in chloroform. The resulting solution was treated with a 1 M aqueous solution of sodium hydroxide until basic and the aqueous phase was subsequently extracted with chloroform (3 ×). The combined organic phases were dried over anhydrous sodium sulfate, the dried solution was filtered and the filtrate was concentrated under reduced pressure. The crude residue was purified by flash column chromatography over silica gel (dichloromethane/MeOH/NH_4OH 18/1/0.15) to afford the title compound.

(78 mg, 71% yield); **^{1}H NMR (400 MHz, $CDCl_3$)** δ 7.30–7.24 (m, 2H), 7.20–7.14 (m, 3H), 2.68–2.54 (m, 6H), 2.39–2.33 (m, 2H), 2.20 (s, 3H), 2.15 (s, 3H), 1.83–1.74 (m, 2H); **^{13}C NMR (100 MHz, $CDCl_3$)** δ 208.2, 142.4, 128.5 (2C), 128.4 (2C), 125.9, 57.2, 52.2, 42.1, 41.8, 33.7, 30.3, 29.1; **IR (neat) ν_{max}:** 2942, 2847, 2793, 1711, 1495, 1454, 1356, 1161, 747, 700; **HRMS (ESI+):** exact mass calculated for $[M+H]^+$ ($C_{14}H_{22}NO$) requires *m/z* 220.1696, found *m/z* 220.1696.

(*E*)-4-((3-Cyclohexylallyl)(methyl)amino)butan-2-one (7b)

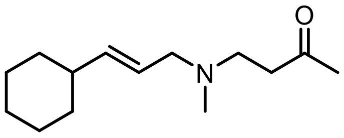

Following procedure A, (method B) cyclohexylacetylene was subjected to hydroaminomethylation-conditions using *N,N,N′,N′*-tetramethyldiaminomethane. After the hydroaminomethylation reaction and cooling to 23 °C, acetone (55 equiv.) was added directly to the reaction mixture and the resulting solution was stirred at 23 °C for 8 h. After this time, volatile components were removed under reduced pressure and the residue was dissolved in chloroform. The resulting solution was treated with a 1 M aqueous solution of sodium hydroxide until basic and the aqueous phase was subsequently extracted with

chloroform (3 ×). The combined organic phases were dried over anhydrous sodium sulfate, the dried solution was filtered and the filtrate was concentrated under reduced pressure. The crude residue was purified by flash column chromatography over silica gel (dichloromethane/MeOH/NH_4OH 18/1/0.15) to afford the title compound.

(61 mg, 55% yield); **^{1}H NMR (600 MHz, $CDCl_3$)** δ 5.53 (dd, *J* = 15.5, 6.6 Hz, 1H), 5.42–5.37 (m, 1H), 2.94 (d, *J* = 6.7 Hz, 2H), 2.67–2.59 (m, 4H), 2.18 (s, 3H), 2.16 (s, 3H), 1.95–1.92 (m, 1H), 1.70 (d, *J* = 10.6 Hz, 4H), 1.64–1.62 (m, 1H), 1.28–1.24 (m, 2H), 1.17–1.12 (m, 1H), 1.09–1.03 (m, 2H); **^{13}C NMR (150 MHz, $CDCl_3$)** δ 208.2, 140.8, 123.8, 60.2, 51.4, 41.9, 41.9, 40.6, 33.1 (2C), 30.2, 26.3, 26.2 (2C); **IR (neat) ν_{max}:** 2923, 2850, 2790, 1715, 1449, 1356, 1228, 1164, 1030, 972; **HRMS (ESI+):** exact mass calculated for $[M+H]^+$ ($C_{14}H_{26}NO$) requires *m/z* 224.2009, found *m/z* 224.2011.

4,4-Dimethyl-1-(methyl(3-phenylpropyl)amino)pentan-3-one (7c)

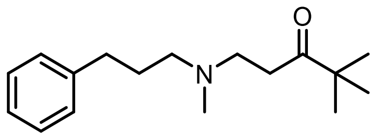

Following procedure A, (method B) styrene was subjected to hydroaminomethylation-conditions using 4 equivalents of *N,N,N′,N′*-tetramethyldiaminomethane. After the hydroaminomethylation reaction and cooling to 23 °C, 3,3-dimethyl-2-butanone (15 equiv.) was added directly to the reaction mixture and the resulting solution was stirred at 23 °C for 8 h. After this time, volatile components were removed under reduced pressure and the residue was dissolved in chloroform. The resulting solution was treated with a 1 M aqueous solution of sodium hydroxide until basic and the aqueous phase was subsequently extracted with chloroform (3 ×). The combined organic phases were dried over anhydrous sodium sulfate, the dried solution was filtered and the filtrate was concentrated under reduced pressure. The crude residue was purified by flash column chromatography over silica gel (dichloromethane/MeOH/NH_4OH 18/1/0.15) to afford the title compound.

(68 mg, 52%yield); **^{1}H NMR (700 MHz, $CDCl_3$)** δ 7.29–7.26 (m, 2H), 7.19–7.17 (m, 3H), 2.63 (m, 6H), 2.39–2.37 (m, 2H), 2.23 (s, 3H), 1.80 (m, 2H), 1.13 (s, 9H); **^{13}C NMR (175 MHz, $CDCl_3$)** δ 215.1, 142.3, 128.5 (2C), 128.5 (2C), 125.9, 57.3, 52.5, 44.4, 42.3, 34.6, 33.7, 29.0, 26.5 (3C); **IR (neat) ν_{max}:** 2928, 2853, 2793, 1703, 1495, 1455, 1365, 1306, 1123, 1081, 1030, 985, 742, 698; **HRMS (ESI+):** exact mass calculated for $[M+H]^+$ ($C_{17}H_{28}NO$) requires *m/z* 262.2165, found *m/z* 262.2162.

3-(Methyl(3-phenylpropyl)amino)-1-phenylpropan-1-one (7d)

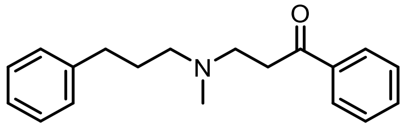

Following procedure A, (method B) styrene was subjected to hydroaminomethylation-conditions using 4 equivalents of *N,N,N′,N′*-tetramethyldiaminomethane. After the hydroaminomethylation reaction and cooling to 23 °C, acetophenone (10 equiv.) was added directly to the reaction mixture and the resulting solution was stirred at 23 °C for 8 h. After this time, volatile components were removed under reduced pressure and the residue was dissolved in chloroform. The resulting solution was treated with a 1 M aqueous solution of sodium hydroxide until basic and the aqueous phase was subsequently extracted with chloroform (3 ×). The combined organic phases were dried over anhydrous sodium sulfate, the dried solution was filtered and the filtrate was concentrated under reduced pressure. The crude residue was purified by flash column chromatography over silica gel (dichloromethane/MeOH/NH_4OH 18/1/0.15) to afford the title compound.

(83 mg, 59% yield); **^{1}H NMR (600 MHz, $CDCl_3$)** δ 7.96–7.95 (m, 2H), 7.58–7.55 (m, 1H), 7.48–7.45 (m, 2H), 7.28–7.26 (m, 2H), 7.19–7.16 (m, 3H), 3.15–3.12 (m, 2H), 2.85–2.83 (m, 2H), 2.63–2.61 (m, 2H), 2.44–2.42 (m, 2H), 2.29 (s, 3H), 1.81 (dt, *J* = 15.0, 7.6 Hz, 2H); **^{13}C NMR (150 MHz, $CDCl_3$)** δ 199.6, 142.3, 137.1, 133.2, 128.7 (2C), 128.6 (2C), 128.4 (2C), 128.2 (2C), 125.9, 57.2, 52.7, 42.4, 36.7, 33.7, 29.1; **IR (neat) ν_{max}:** 2939, 2849, 2794, 1682, 1598, 1495, 1450, 1206, 1180, 745, 696; **HRMS (ESI+):** exact mass calculated for $[M+H]^+$ ($C_{19}H_{24}NO_3$) requires *m/z* 282.1852, found *m/z* 282.1854.

1-(4-Methoxyphenyl)-3-(methyl(3-phenylpropyl)amino)propan-1-one (7e)

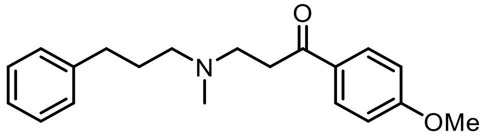

Following procedure A, (method B) styrene was subjected to hydroaminomethylation-conditions using 4 equivalents of *N,N,N′,N′*-tetramethyldiaminomethane. After the hydroaminomethylation reaction and cooling to 23 °C, 4′-methoxyacetophenone (20 equiv.) was added directly to the reaction mixture and the resulting solution was stirred at 23 °C for 8 h. After this time, volatile components were removed under reduced pressure and the residue was dissolved in chloroform. The resulting solution was treated with a 1 M aqueous solution of sodium hydroxide until basic and the aqueous phase was subsequently extracted with chloroform (3 ×). The combined organic phases were dried over anhydrous sodium sulfate, the dried solution was filtered and the filtrate was concentrated under reduced pressure. The crude residue was purified by flash column chromatography over silica gel (dichloromethane/MeOH/NH_4OH 18/1/0.15) to afford the title compound.

(109 mg, 70% yield); **^{1}H NMR (400 MHz, $CDCl_3$)** δ 7.95–7.92 (m, 2H), 7.29–7.25 (m, 2H), 7.19–7.15 (m, 3H), 6.95–6.92 (m, 2H), 3.87 (s, 3H), 3.10 (t, *J* = 7.4 Hz, 2H), 2.85 (t, *J* = 7.3 Hz, 2H), 2.64–2.60 (m, 2H), 2.47–

2.43 (m, 2H), 2.30 (s, 3H), 1.82 (dt, J = 15.0, 7.6 Hz, 2H); **^{13}C NMR (100 MHz, CDCl$_3$)** δ 197.0, 163.5, 142.1, 130.4 (2C), 130.1, 128.4, 128.3 (2C), 125.8, 113.8 (2C), 57.1, 55.5, 52.7, 42.2, 36.1, 33.5, 28.8; **IR (neat) ν_{max}:** 2926, 2850, 1673, 1599, 1576, 1510, 1456, 1419, 1310, 1240, 1169, 1030, 982, 839, 700; **HRMS (ESI+):** exact mass calculated for $[M+H]^+$ ($C_{20}H_{26}NO_2$) requires *m/z* 312.1958, found *m/z* 312.1958.

3-(Methyl(3-phenylpropyl)amino)-1-(p-tolyl)propan-1-one (7f)

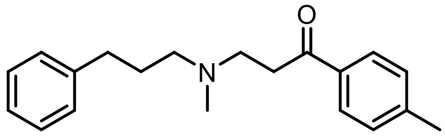

Following procedure A, (method B) styrene was subjected to hydroaminomethylation-conditions using 4 equivalents of *N,N,N′,N′*-tetramethyldiaminomethane. After the hydroaminomethylation reaction and cooling to 23 °C, 4′-methylacetophenone (20 equiv.) was added directly to the reaction mixture and the resulting solution was stirred at 23 °C for 8 h. After this time, volatile components were removed under reduced pressure and the residue was dissolved in chloroform. The resulting solution was treated with a 1 M aqueous solution of sodium hydroxide until basic and the aqueous phase was subsequently extracted with chloroform (3 ×). The combined organic phases were dried over anhydrous sodium sulfate, the dried solution was filtered and the filtrate was concentrated under reduced pressure. The crude residue was purified by flash column chromatography over silica gel (dichloromethane/MeOH/NH_4OH 18/1/0.15) to afford the title compound.

(86 mg, 58% yield); **^{1}H NMR (400 MHz, CDCl$_3$)** δ 7.86–7.84 (m, 2H), 7.29–7.25 (m, 4H), 7.19–7.16 (m, 3H), 3.12 (t, J = 7.3 Hz, 2H), 2.85 (t, J = 7.3 Hz, 2H), 2.64–2.60 (m, 2H), 2.47–2.43 (m, 2H), 2.41 (s, 3H), 2.30 (s, 3H), 1.82 (m, 2H); **^{13}C NMR (100 MHz, CDCl$_3$)** δ 199.0, 143.8, 134.5, 129.7, 129.3 (2C), 128.4 (2C), 128.3 (2C), 128.2 (2C), 125.8, 57.0, 52.6, 42.1, 36.4, 33.5, 28.8, 21.6; **IR (neat) ν_{max}:** 3026, 2941, 2853, 2794, 1678, 1605, 1453, 1407, 1376, 1326, 1287, 1230, 1205, 1180; **HRMS (ESI+):** exact mass calculated for $[M+H]^+$ ($C_{20}H_{26}NO$) requires *m/z* 296.2009, found *m/z* 296.2010.

1-(4-Fluorophenyl)-3-(methyl(3-phenylpropyl)amino)propan-1-one (7g)

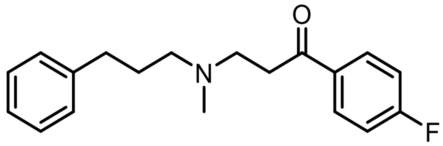

Following procedure A, (method B) styrene was subjected to hydroaminomethylation-conditions using 4 equivalents of *N,N,N′,N′*-tetramethyldiaminomethane. After the hydroaminomethylation reaction and cooling to 23 °C, 4′-fluoroacetophenone (20 equiv.) was added directly to the reaction mixture and the resulting solution was stirred at 23 °C for 8 h. After this time, volatile components were removed under reduced pressure and the residue was dissolved in chloroform. The resulting solution was treated with a 1 M aqueous solution of sodium hydroxide until basic and the aqueous phase was subsequently extracted

with chloroform (3 ×). The combined organic phases were dried over anhydrous sodium sulfate, the dried solution was filtered and the filtrate was concentrated under reduced pressure. The crude residue was purified by flash column chromatography over silica gel (dichloromethane/MeOH/NH_4OH 18/1/0.15) to afford the title compound.

(91 mg, 61% yield); **^{1}H NMR (400 MHz, CDCl$_3$)** δ 7.99–7.96 (m, 2H), 7.29 –7.25 (m, 2H), 7.19–7.11 (m, 5H), 3.10 (t, *J* = 7.3 Hz, 2H), 2.84 (t, *J* = 7.3 Hz, 2H), 2.64–2.60 (m, 2H), 2.45–2.41 (m, 2), 2.29 (s, 3H), 1.81 (dt, *J* = 15.0, 7.5 Hz, 2H); . **^{13}C NMR (100 MHz, CDCl$_3$)** δ 197.8, 165.7 (d, *J* = 255 Hz), 142.1, 133.4, 130.7 (d, *J* = 9.3 Hz, 2C), 128.4 (2C), 128.3 (2C), 125.8, 115.7 (d, *J* = 21.9 Hz, 2C), 57.1, 52.5, 42.2, 36.5, 33.5, 28.8; **IR (neat) ν_{max}:** 2943, 2796, 1684, 1598, 1505, 1454, 1410, 1369, 1299, 1229, 1156, 844, 700; **HRMS (ESI+):** exact mass calculated for $[M+H]^+$ ($C_{19}H_{23}NOF$) requires *m/z* 300.1758, found *m/z* 300.1759.

_N_-Methyl-3-phenyl-_N_-(2,4,6-trimethoxybenzyl)propan-1-amine (7h)

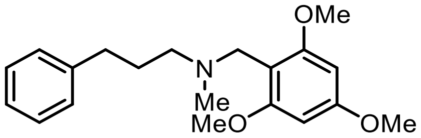

Following procedure A, (method B) styrene was subjected to hydroaminomethylation-conditions using 4 equivalents of *N,N,N′,N′*-tetramethyldiaminomethane. After the hydroaminomethylation reaction and cooling to 23 °C, 1,3,5-trimethoxybenzene (15 equiv.) was added directly to the reaction mixture and the resulting solution was warmed up to 50 °C and stirred for 8 h. After this time, volatile components were removed under reduced pressure and the residue was dissolved in chloroform. The resulting solution was treated with a 1 M aqueous solution of sodium hydroxide until basic and the aqueous phase was subsequently extracted with chloroform (3 ×). The combined organic phases were dried over anhydrous sodium sulfate, the dried solution was filtered and the filtrate was concentrated under reduced pressure. The crude residue was purified by flash column chromatography over silica gel (dichloromethane/MeOH/NH_4OH 18/1/0.15) to afford the title compound.

(89 mg, 54% yield); **^{1}H NMR (600 MHz, CDCl$_3$)** δ 7.29–7.26 (m, 2H), 7.20 (t, *J* = 7.4 Hz, 1H), 7.16 (d, *J* = 7.0 Hz, 2H), 6.11 (s, 2H), 4.24 (m, 1H), 4.12 (m, 1H), 3.82 (s, 3H), 3.79 (s, 6H), 3.09 (br s, 1H), 2.80 (br s, 1H), 2.68 (t, *J* = 6.2 Hz, 2H), 2.61 (s, 3H), 2.28 (br s, 1H), 2.17 (br s, 1H); **^{13}C NMR (150 MHz, CDCl$_3$)** δ 163.3 (2C), 161.1, 160.6, 140.1, 128.7 (2C), 128.5 (2C), 126.5, 90.6 (2C), 55.9 (2C), 55.6, 55.1, 47.5, 39.6, 33.2, 25.6; **IR (neat) ν_{max}:** 3397, 2941, 2845, 2613, 1609, 1594, 1458, 1421, 1232, 1204, 1152, 1055, 1033, 951, 819; **HRMS (ESI+):** exact mass calculated for $[M+H]^+$ ($C_{20}H_{28}NO_3$) requires *m/z* 330.2064, found *m/z* 330.2055.

N,N-Dimethyl-3-phenylpropan-1-amine (7i)

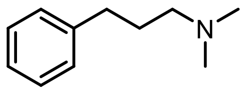

Following method A, a round-bottom flask covered from light was charged with *N,N*-dimethylmethyleneiminium iodide (Eschenmoser´s iodide, 4 equiv.) and a magnetic stir-bar under argon-atmosphere was cooled to 0 °C. After this, styrene (1 equivalent) and 1,1,1,3,3,3-hexafluoroisopropanol (HFIP, 0.6 M with respect to the alkene) were added. After completed addition of the solvent, the flask was sealed and placed in an oil bath at 75 °C. The reaction was vigorously stirred at this temperature for 2 h, after which it was allowed to cool to room temperature. Subsequently, $NaBH(OAc)_3$ was added (15 equiv. with respect to the alkene) followed by methanol (10 ml/mmol). The reaction was stirred at room temperature for 6 hours; then aqueous sodium hydroxide (0.1 M) was added until the reaction mixture reached pH 12. The resulting biphasic mixture was separated, and the aqueous phase was extracted with dichloromethane (3 x 200 mL/mmol). The combined organic phases were then dried over anhydrous potassium carbonate and filtered. The filtrate was concentrated under reduced pressure to afford the crude product, which was purified by flash column chromatography on silica gel (dichloromethane/MeOH/NH_4OH 19:1:0.15) to afford the analytically pure desired product.

(60 mg, 74% yield); **^{1}H NMR (400 MHz, $CDCl_3$)** δ 7.30–7.25 (m, 2H), 7.20–7.16 (m, 3H), 2.67–2.63 (m, 2H), 2.36–2.27 (m, 2H), 2.27 (s, 6H), 1.82 (dt, *J* = 15.2, 7.6 Hz, 2H); **^{13}C NMR (100 MHz, $CDCl_3$)** δ 142.2, 128.5 (2C), 128.5 (2C), 125.9, 59.3, 45.5 (2C), 33.7, 29.3; **IR (neat) ν_{max}:** 3403, 3061, 3026, 2922, 2852, 2781, 1667, 1602, 1495, 1454, 1377, 1030; **HRMS (ESI+):** exact mass calculated for $[M+H]^+$ ($C_{11}H_{18}N$) requires *m/z* 164.1434, found *m/z* 164.1438.

N-Ethyl-N-methyl-3-phenylpropan-1-amine (7j)

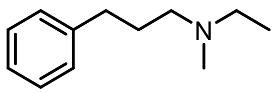

Following method A, a round-bottom flask covered from light was charged with *N,N* dimethylmethyleneiminium iodide (Eschenmoser´s iodide, 4 equiv.) and a magnetic stir-bar under argon-atmosphere was cooled to 0 °C. After this, styrene (1 equivalent) and 1,1,1,3,3,3-hexafluoroisopropanol (HFIP, 0.6 M with respect to the alkene) were added. After completed addition of the solvent, the flask was sealed and placed in an oil bath at 75 °C. The reaction was vigorously stirred at this temperature for 2 h, after which it was allowed to cool to room temperature. Subsequently, dimethylzinc was added (15 equiv. with respect to the alkene, 1 mol/L solution in heptane). The reaction was stirred at room temperature for 5 hours. Water was slowly added to quench the reaction, then aqueous sodium hydroxide (0.1 M) was added until the reaction mixture reached pH 12. The resulting biphasic mixture was separated, and the aqueous phase was extracted with dichloromethane (3 x mL/mmol). The combined organic phases were then dried over anhydrous potassium carbonate and

filtered. The filtrate was concentrated under reduced pressure to afford the crude product, which was purified by flash column chromatography on silica gel (dichloromethane/MeOH/NH_4OH 19:1:0.15) to afford the analytically pure desired product.

(58 mg, 66% yield); **^{1}H NMR (400 MHz, $CDCl_3$)** δ 7.30–7.26 (m, 2H), 7.19 (d, *J* = 7.4 Hz, 3H), 2.64 (t, *J* = 7.8 Hz, 2H), 2.50–2.47 (m, 4H), 2.26 (s, 3H), 1.86 (dd, *J* = 15.3, 7.8 Hz, 2H), 1.08 (t, *J* = 7.2 Hz, 3H); **^{13}C NMR (100 MHz, $CDCl_3$)** δ 142.2, 128.5 (2C), 128.5 (2C), 125.9, 56.8, 51.5, 41.6, 33.9, 28.9, 12.1; **IR (neat) ν_{max}:** 3433, 3351, 2943, 2932, 2361, 1495, 1455, 750, 701; **HRMS (ESI+):** exact mass calculated for $[M+H]^+$ ($C_{12}H_{20}N$) requires *m/z* 178.1590, found *m/z* 178.1592.

***N*-Methyl-3-phenyl-*N*-propylpropan-1-amine (7k)**

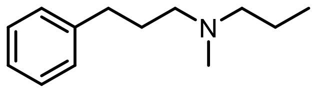

Following method A, a round-bottom flask covered from light was charged with *N,N*-dimethylmethyleneiminium iodide (Eschenmoser´s iodide, 4 equiv.) and a magnetic stir-bar under argon-atmosphere was cooled to 0 °C. After this, styrene (1 equivalent) and 1,1,1,3,3,3-hexafluoroisopropanol (HFIP, 0.6 M with respect to the alkene) were added. After completed addition of the solvent, the flask was sealed and placed in an oil bath at 75 °C. The reaction was vigorously stirred at this temperature for 2 h, after which it was allowed to cool to room temperature. Subsequently, diethylzinc was added (15 equiv. with respect to the alkene, 1 mol/L solution in hexanes). The reaction was stirred at room temperature for 6 hours. Water was slowly added to quench the reaction, then aqueous sodium hydroxide (0.1 M) was added until the reaction mixture reached pH 12. The resulting biphasic mixture was separated and the aqueous phase was extracted with c (3 x 200 mL/mmol). The combined organic phases were then dried over anhydrous potassium carbonate and filtered. The filtrate was concentrated under reduced pressure to afford the crude product, which was purified by flash column chromatography on silica gel (dichloromethane/MeOH/NH_4OH 19:1:0.15) to afford the analytically pure desired product.

(62 mg, 63% yield); **^{1}H NMR (400 MHz, $CDCl_3$)** δ 7.30–7.26 (m, 2H), 7.20–7.16 (m, 3H), 2.65–2.61 (m, 2H), 2.39–2.35 (m, 2H), 2.29 (dd, *J* = 8.6, 6.7 Hz, 2H), 2.22 (s, 3H), 1.84–1.76 (m, 2H), 1.52–1.43 (m, 2H), 0.89 (t, *J* = 7.4 Hz, 3H); **^{13}C NMR (100 MHz, $CDCl_3$)** δ 142.6, 128.5 (2C), 128.4 (2C), 125.8, 60.0, 57.5, 42.4, 33.9, 29.2, 20.6, 12.1; **IR (neat) ν_{max}:** 3433, 3352, 3207, 2954, 2922, 2852,1658, 1633, 1467, 1422; **HRMS (ESI+):** exact mass calculated for $[M+H]^+$ ($C_{13}H_{22}N$) requires *m/z* 192.1747, found *m/z* 192.1749.

2.7 Synthesis of Naftifine

(*E*)-*N*-Methyl-3-phenylprop-2-en-1-amine (6f)

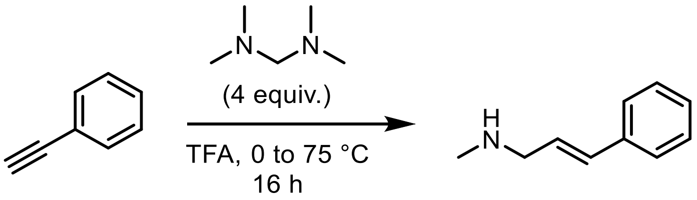

Prepared according to procedure A, method B with 4 equiv. *N,N,N',N'*-tetramethyldiaminomethane; (42 mg, 57% yield), see above.

Naftifine (8)

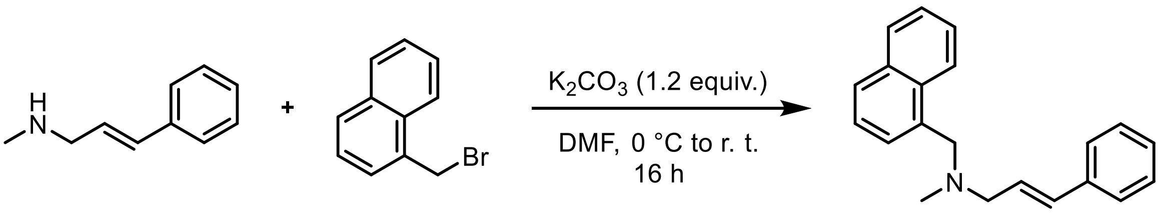

(*E*)-N-methyl-3-phenylprop-2-en-1-amine (9.5 mg, 0.0645 mmol, 1 equiv.) and potassium carbonate (10.7 mg, 0.0774 mmol, 1.2 equiv.) were suspended in 1 mL of anhydrous *N,N*-dimethylformamide (DMF) and cooled to 0 °C. 1-(Bromomethyl)naphthalene (14 mg, 0.0645 mmol, 1 equiv.) was dissolved in 2 mL of anhydrous DMF and added dropwise over 15 minutes. The mixture was then allowed to warm to room temperature and stirred for further 16 hours. Diethyl ether and NaOH (1 M) were then added; the aqueous phase was washed with ether (3 × 10 mL) and the organic phases were combined, dried over potassium carbonate and filtered. The filtrate was concentrated under reduced pressure to afford the crude product, which was purified by flash column chromatography on silica gel (dichloromethane/MeOH/NH_4OH 19:1:0.15) to afford the analytically pure desired product in 70% yield (12.9 mg).

^{1}H NMR (400 MHz, $CDCl_3$) δ 8.32 (d, *J* = 8.3 Hz, 1H), 7.87–7.84 (m, 1H), 7.78 (d, *J* = 8 Hz, 1H), 7.55–7.39 (m, 6H), 7.34–7.30 (m, 2H), 7.25–7.21 (m, 1H), 6.59 (d, *J* = 15.9 Hz, 1H), 6.38 (dt, *J* = 15.9, 6.6 Hz, 1H), 3.96 (s, 2H), 3.29 (dd, *J* = 6.6, 1.1 Hz, 2H), 2.29 (s, 3H); **^{13}C NMR (100 MHz, $CDCl_3$)** δ 137.4, 135.1, 134.1, 132.9, 132.7, 128.7 (2C), 128.6, 128.1, 127.8, 127.6, 127.6, 126.5 (2C), 126.1, 125.8, 125.3, 124.8, 60.6, 60.3, 42.7; **IR (neat) ν_{max}:** 3028, 2927, 2836, 2785, 1597, 1494, 1450, 1363, 1015, 968, 793, 775; **HRMS (ESI+):** exact mass calculated for $[M+H]^+$ ($C_{31}H_{32}N$) requires *m/z* 228.1747, found *m/z* 288.1746.

2.8 Mechanistic Experiments

2.8.1 Deuteration Experiments

N-(Methyl-*d*$_3$)-3-phenylpropan-3-*d*-1-amine (9a)

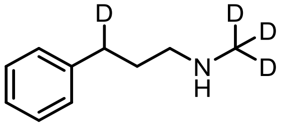

Prepared with 4 equiv. *N,N,N',N'*-tetra(methyl-*d*$_3$)methanediamine; (55 mg, 72% yield; **^{1}H NMR (600 MHz, CDCl$_3$)** δ 7.30–7.25 (m, 2H), 7.21–7.16 (m, 3H), 2.68–2.63 (m, 1H), 2.61 (t, *J* = 7.2 Hz, 2H), 1.81 (app q, *J* = 7.2 Hz, 2H), 1.16 (br s, 1H); **^{13}C NMR (150 MHz, CDCl$_3$)** δ 142.3, 128.5 (2C), 128.4 (2C), 125.9, 51.7, 35.8 (sept, *J* = 20.3 Hz), 33.4 (t, *J* = 19.4 Hz), 31.7; **IR (neat) ν_{max}:** 3025, 2927, 1541, 1495, 1450, 1408, 1366, 1306, 742, 700; **HRMS (ESI+):** exact mass calculated for $[M+H]^+$ ($C_{10}H_{12}D_4N$) requires *m/z* 154.1528, found *m/z* 154.1527.

2.8.2 Kinetic Isotope Effect Measurements

Following the general procedure, alkenes **1m** and **1a** were submitted to hydroaminomethylation using *N,N'*-dibutyl-*N,N'*-bis(butyl-d$_9$)methanediamine aminal. K_H/K_D values were obtained through mass spectrometry and, for **9c**, NMR-analysis.[C]

The obtained HRMS-data are shown below.

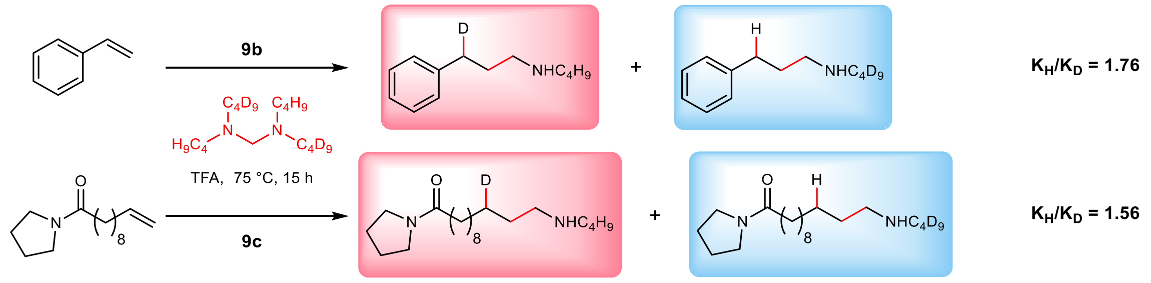

Following the general procedure, phenylacetilene **5i** was submitted to hydroaminomethylation using a 50/50 mixture of *N,N,N',N'*-Tetramethyldiaminomethane and *N,N,N',N'*-Tetra(methyl-d$_3$)diaminomethane aminal. The ratios were obtained through mass spectrometry.[c]

[C] **9b** and **9d** were not amenable to K_H/K_D-determination through NMR-analysis due to signal overlap and ambiguity. The crude spectra are provided in the following section.

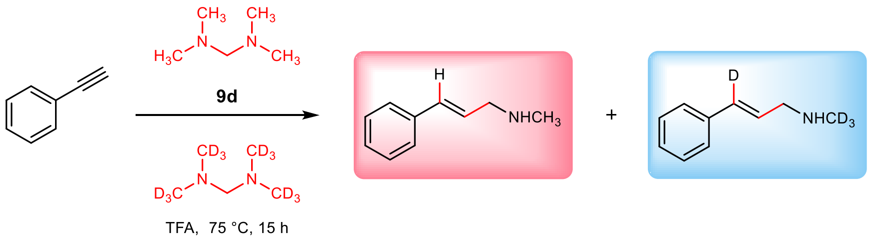

H/D = 1.08

9b-crude								Measurement 1	Measurement 2	Measurement 3	
Measurement 1		Measurement 2		Measurement 3							
m/z	Intensity	m/z	Intensity	m/z	Intensity						
193.1813	278363	193.1814	269583	193.1813	272728		Σ Intensity	322318	312195	315408	
194.1846	41133	194.1846	39908	194.1846	39865		Σ Intensity	569634	552285	553470	
195.1878	2822	195.1878	2704	195.1878	2815						Avg.
200.2252	13359	200.2252	13041	200.2252	13036		K_H/K_D	1.76730	1.76904	1.75477	**1.764**
201.2316	486079	201.2316	471207	201.2316	471942						
202.2348	65540	202.2348	63587	202.2347	63891						
203.2381	4656	203.2381	4450	203.2380	4601						

9c-crude								Measurement 1	Measurement 2	Measurement 3	
Measurement 1		Measurement 2		Measurement 3							
m/z	Intensity	m/z	Intensity	m/z	Intensity						
326.3277	182281	326.3277	197583	326.3277	200246						
327.3309	40099	327.3309	43957	327.3309	44657		Σ Intensity	227494	247057	250501	
328.3343	5114	328.3344	5517	328.3345	5598		Σ Intensity	355245	385362	392931	
333.3714	8652	333.3714	9194	333.3714	9519						Avg.
334.3778	276631	334.3778	300216	334.3779	306563		K_H/K_D	1.56156	1.55981	1.56858	**1.563**
335.3810	62783	335.3811	68185	335.3811	68911						
336.3842	7179	336.3842	7767	336.3841	7938						

9d-crude								Measurement 1	Measurement 2	Measurement 3	
Measurement 1		Measurement 2		Measurement 3							
m/z	Intensity	m/z	Intensity	m/z	Intensity						
152.1373	10611	152.1372	10931	152.1373	11116						
153.141	1145	153.1409	1141	153.141	1200		Σ Intensity	13112	13429	13670	
154.1514	1356	154.1513	1357	154.1513	1354		Σ Intensity	13857	14585	15061	
											Avg.
148.1123	12344	148.1123	12987	148.1123	13451		K_H/K_D	1.05682	1.08608	1.10176	**1.082**
149.0918	81	149.0918	76	149.092	73						
149.1158	1432	149.1157	1522	149.1158	1537						

2.8.3 Reactions with Bis(piperidinyl)methane: Domino Hydroaminomethylation/C–H Functionalization

The use of bis(piperidinyl)methane (**2g**) led to a scenario where *in situ* hydrogenation allowed the synthesis of a functionalized, 3-substituted piperidine (**10a–c**, Fig. S1). Likely, iminium/enamine isomerization followed by interception with a formaldehyde equivalent resulting from aminal activation funnels down to an intermediate which, upon hydrogenation, accounts for the formation of **10a.** This transformation formally represents a domino hydroaminomethylation/functionalization. The same reactivity was observed using styrene, offering a slightly more diverse picture: reduction of the putative cationic intermediate with $NaBH(OAc)_3$ afforded a mixture of the fully reduced product and the corresponding product of 1,2-reduction (**10b** and **10c,** 87%).

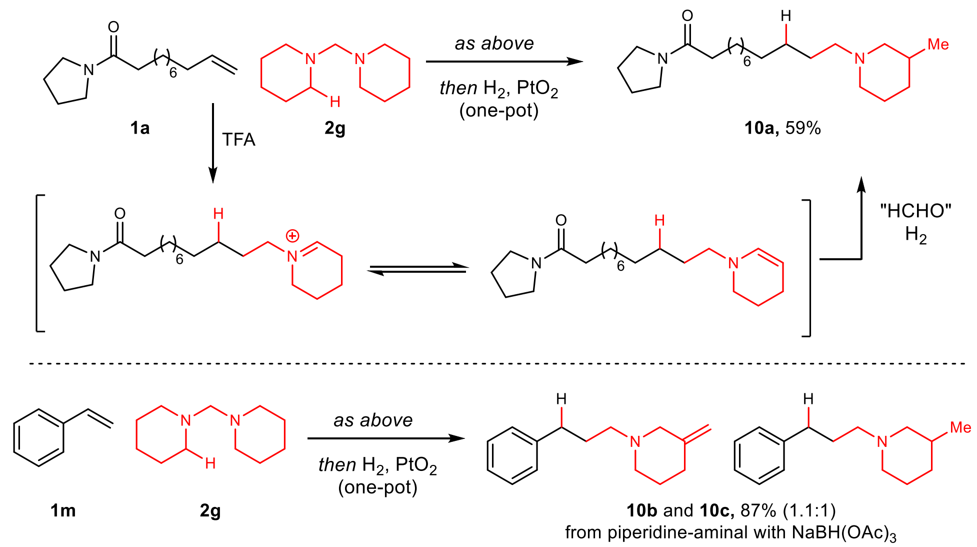

Figure S1. Reactions with bis(piperidinyl)methane: Domino hydroaminomethylation/C–H functionalization.

12-(3-Methylpiperidin-1-yl)-1-(pyrrolidin-1-yl)dodecan-1-one (10a)

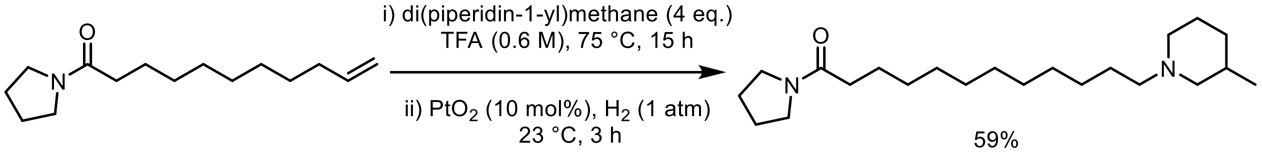

Following procedure A, the alkene was subjected to hydroaminomethylation-conditions using di(piperidin-1-yl)methane (**2g**). After removal of volatile components under reduced pressure, platinum(IV) oxide (Adams's catalyst, 10 mol%) was added and the reaction mixture was placed under an atmosphere of dihydrogen by repeated (3 ×) evacuation and back-filling with H_2. The heterogeneous mixture was stirred at room temperature (23 °C) for 3 h, after which hydrochloric acid (1 M) and chloroform were added. The biphasic mixture was vigorously stirred for 1 h (in order to hydrolyse excess aminal) and subsequently treated with aqueous sodium hydroxide (5 M) until it reached pH 12. The phases were separated and the aqueous phase was extracted with chloroform (2 ×) and the combined organic phases were dried over anhydrous sodium sulfate. In the case of the presence of remaining PtO_2, filtration over Celite® afforded a colourless solution. The solution was concentrated under reduced pressure and the crude residue was purified by flash column chromatography over silica gel (dichloromethane/MeOH/NH_4OH 18/1/0.15) to afford the desired products.

(103 mg, 59% yield; **^{1}H NMR (600 MHz, CDCl$_3$)** δ 3.45 (t, *J* = 6.8 Hz, 2H), 3.40 (t, *J* = 6.9 Hz, 2H), 2.86 (d, *J* = 10.5 Hz, 1H), 2.81 (d, *J* = 10.5 Hz, 1H), 2.29–2.20 (m, 4H), 1.97–1.91 (m, 2H), 1.87–1.80 (m, 2H), 1.75 (t, *J* = 11.4 Hz, 1H), 1.70–1.53 (m, 6H), 1.47–1.44 (m, 3H), 1.34–1.21 (m, 14H), 0.88–0.78 (m, 4H); **^{13}C NMR (150 MHz, CDCl$_3$)** δ 172.0, 62.4, 59.6, 54.3, 46.7, 45.7, 35.0, 33.3, 31.3, 29.8, 29.7 (2C), 29.7, 29.6, 29.6, 27.9, 27.1, 26.3, 25.8, 25.1, 24.6, 20.0; **IR (neat) ν_{max}:** 2924, 2852, 2800, 2759, 1645, 1430, 1345; **HRMS (ESI+):** exact mass calculated for $[M+H]^+$ ($C_{22}H_{43}N_2O$) requires *m/z* 351.3370, found *m/z* 351.3372.

3-Methylene-1-(3-phenylpropyl)piperidine (10b) and 3-Methyl-1-(3-phenylpropyl)piperidine (10c)

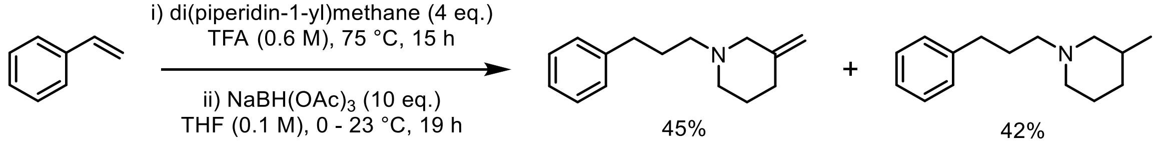

Following procedure A, styrene was subjected to hydroaminomethylation-conditions using di(piperidin-1-yl)methane. After removal of volatile components under reduced pressure, tetrahydrofuran (THF, 0.1 M) was added and the reaction mixture was cooled to 0 °C. After cooling, $NaBH(OAc)_3$ (10 equiv.) was added and the reaction mixture was allowed to warm to room temperature (23 °C) over the course of 19 h. After this time, aqueous sodium hydroxide (5 M) was added and the biphasic mixture was extracted with chloroform (3 ×). The combined organic phases were dried over anhydrous sodium sulfate, filtered and the filtrate was concentrated under reduced pressure. The crude residue was purified by flash column chromatography over silica gel (dichloromethane/MeOH/NH_4OH 54/1/0.15) to afford the desired products.

3-Methylene-1-(3-phenylpropyl)piperidine (10b)

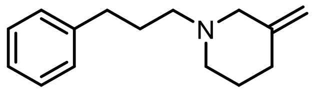

(48 mg, 45% yield; **^{1}H NMR (600 MHz, $CDCl_3$)** δ 7.30–7.25 (m, 2H), 7.21–7.14 (m, 3H), 4.75 (d, *J* = 13.6 Hz, 2H), 2.90 (s, 2H), 2.63 (t, *J* = 7.7 Hz, 2H), 2.49–2.45 (m, 2H), 2.40–2.36 (m, 2H), 2.17–2.12 (m, 2H), 1.88–1.81 (m, 2H), 1.69–1.63 (m, 2H); **^{13}C NMR (150 MHz, $CDCl_3$)** δ 144.9, 142.4, 128.5 (2C), 128.4 (2C), 125.9, 109.2, 60.7, 58.3, 53.9, 34.0, 33.0, 28.9, 26.6; **IR (neat) ν_{max}:** 2939, 2855, 2797, 2755, 2727, 1658, 1495, 1452, 1128, 893, 747, 699; **HRMS (ESI+):** exact mass calculated for $[M+H]^+$ ($C_{15}H_{22}N$) requires *m/z* 216.1747, found *m/z* 216.1747.

3-Methyl-1-(3-phenylpropyl)piperidine (10c)

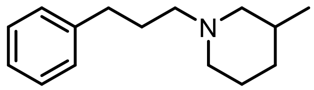

(45 mg, 42% yield; **^{1}H NMR (600 MHz, $CDCl_3$)** δ 7.27–7.23 (m, 2H), 7.18–7.13 (m, 3H), 2.81 (dd, *J* = 25.0, 10.9 Hz, 2H), 2.59 (t, *J* = 8.0 Hz, 2H), 2.33–2.28 (m, 2H), 1.84–1.73 (m, 3H), 1.70–1.58 (m, 3H,), 1.57–1.52 (m, 1H), 1.47 (t, *J* = 10.5 Hz, 1H), 0.84–0.77 (m, 4H); **^{13}C NMR (150 MHz, $CDCl_3$)** δ 142.5, 128.5 (2C), 128.4 (2C), 125.8, 62.3, 58.8, 54.2, 34.1, 33.3, 31.3, 28.9, 25.8, 20.0; **IR (neat) ν_{max}:** 2926, 2869, 2852, 2800, 2759, 1454, 1126, 746, 698; **HRMS (ESI+):** exact mass calculated for $[M+H]^+$ ($C_{15}H_{24}N$) requires *m/z* 218.1903, found *m/z* 218.1904.

3 NMR Spectra

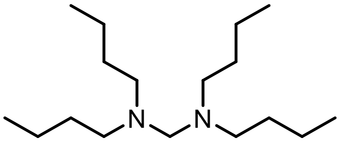

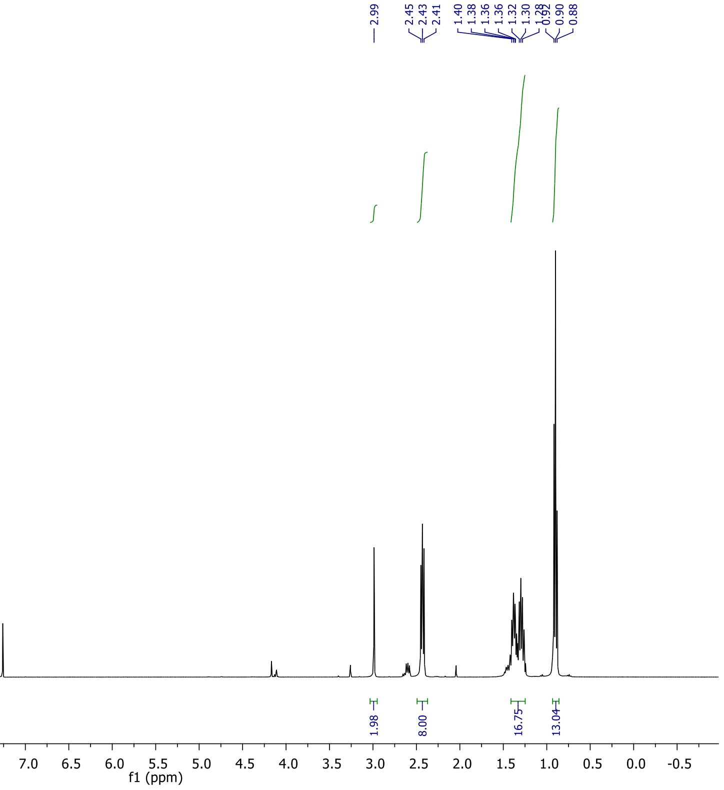

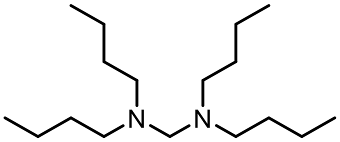

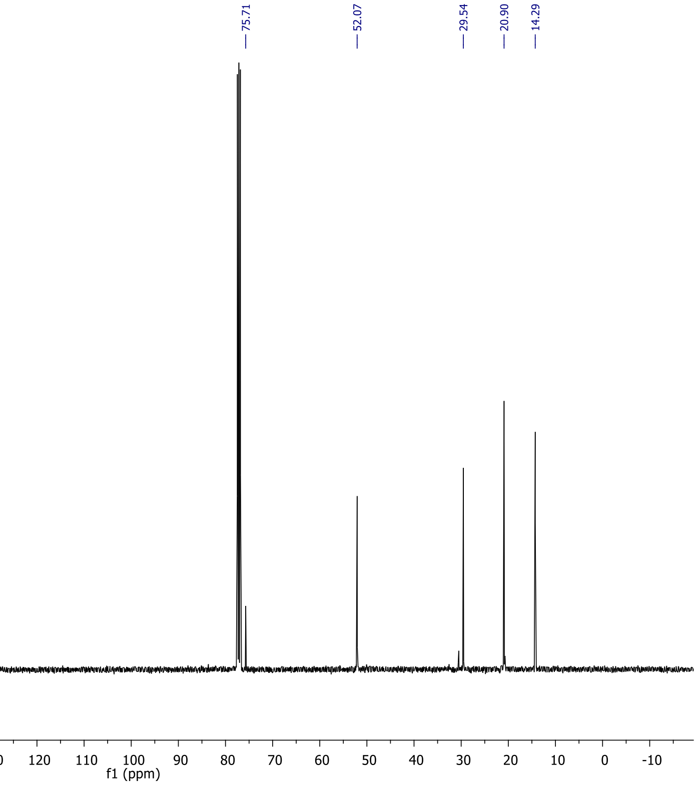

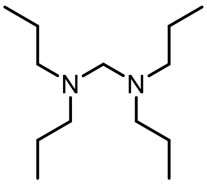
N
N

crude

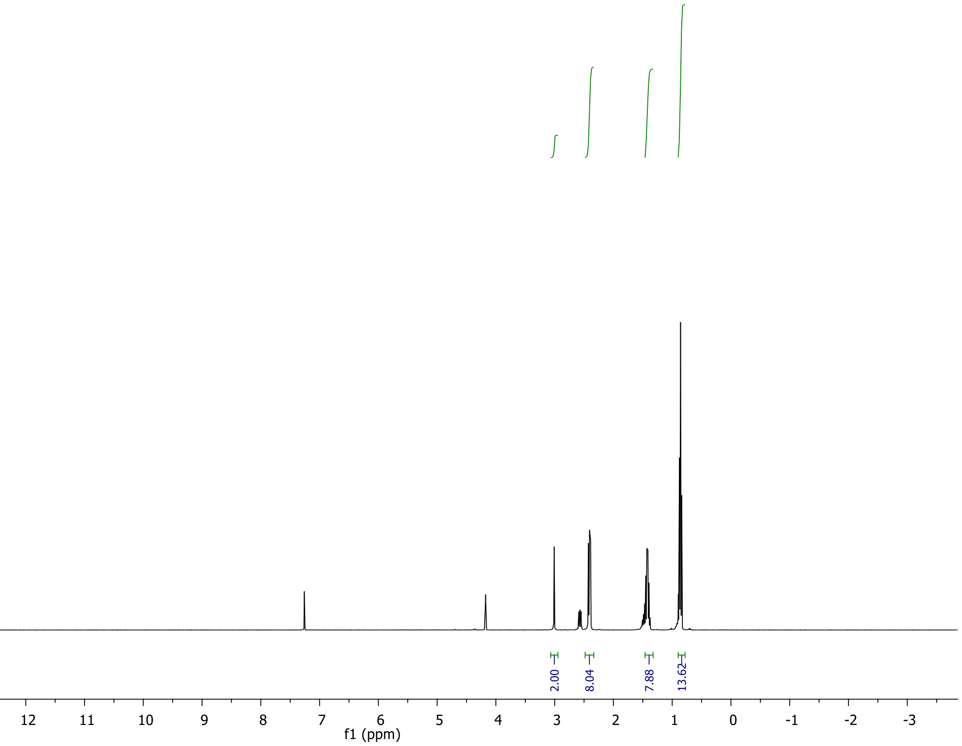
2.00
8.04
7.88
13.62
f1 (ppm)

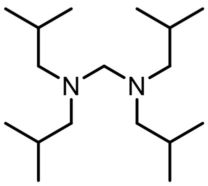
N
N

crude

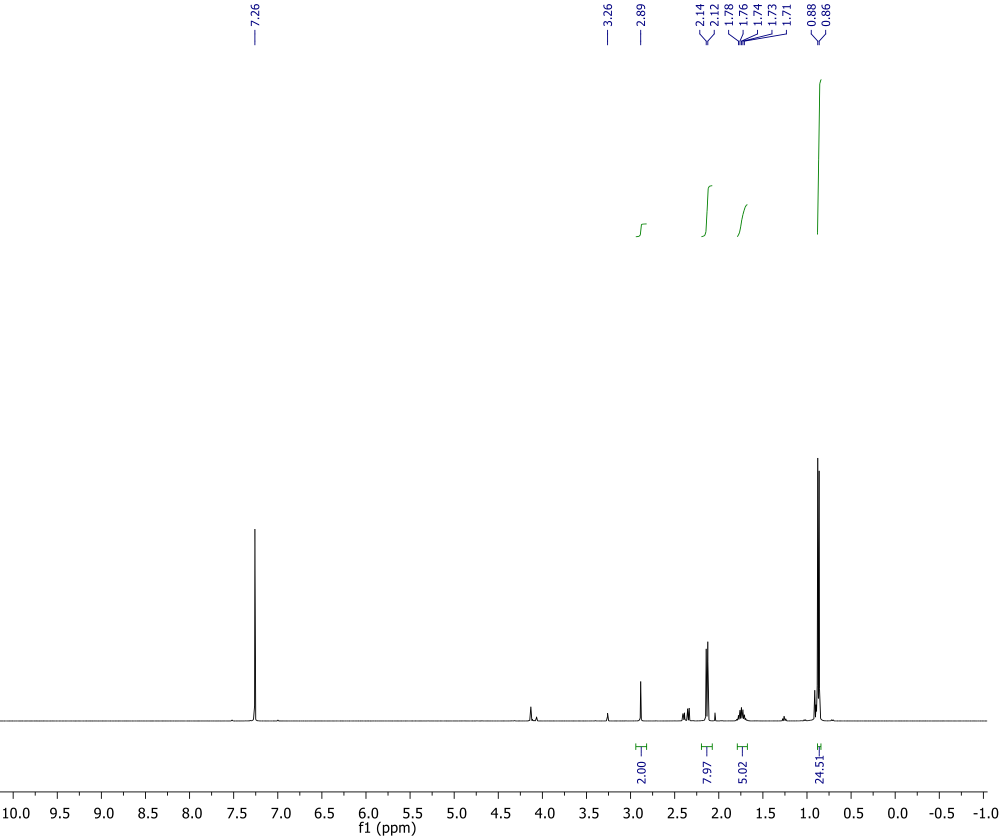
7.26
3.26
2.89
2.14
2.12
1.78
1.76
1.74
1.73
1.71
0.88
0.86
2.00
7.97
5.02
24.51
f1 (ppm)

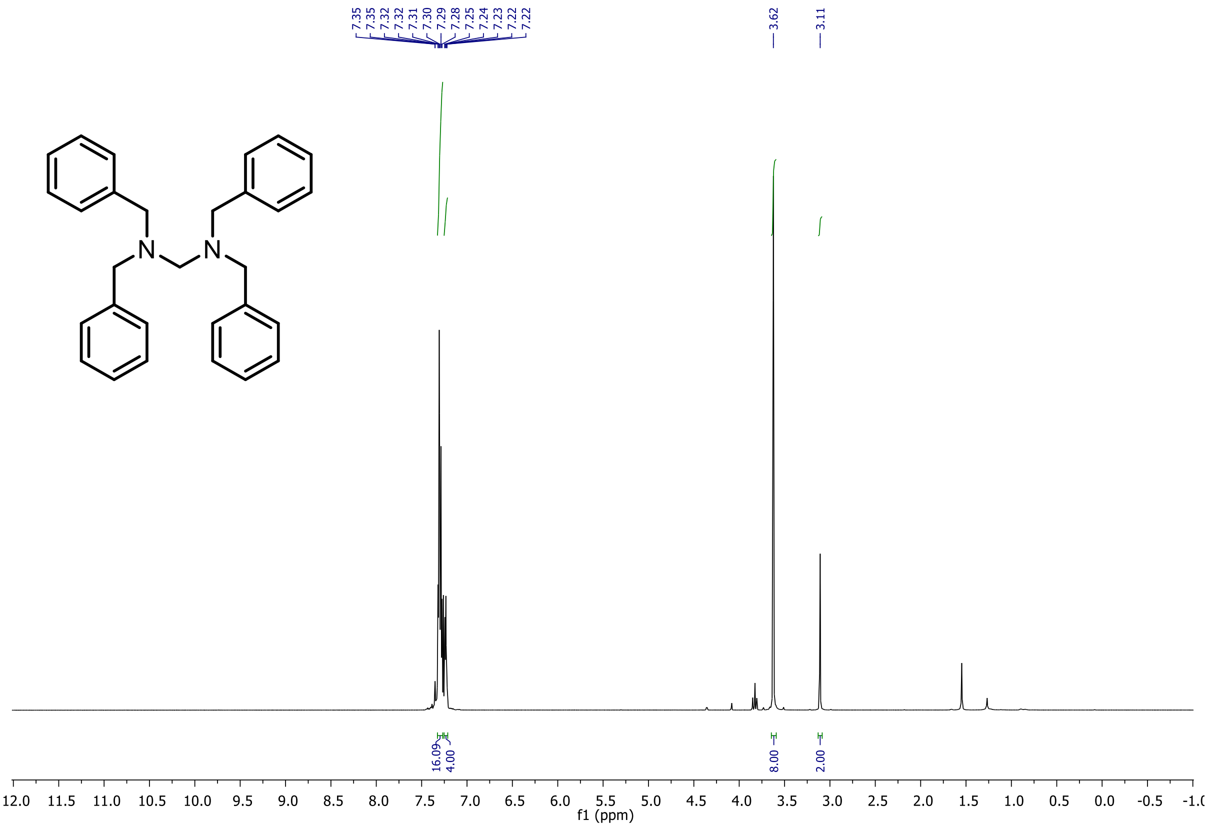
7.35
7.35
7.32
7.32
7.31
7.30
7.29
7.28
7.25
7.24
7.23
7.22
7.22
3.62
3.11
N
N
16.09
4.00
8.00
2.00
12.0 11.5 11.0 10.5 10.0 9.5 9.0 8.5 8.0 7.5 7.0 6.5 6.0 5.5 5.0 4.5 4.0 3.5 3.0 2.5 2.0 1.5 1.0 0.5 0.0 -0.5
f1 (ppm)

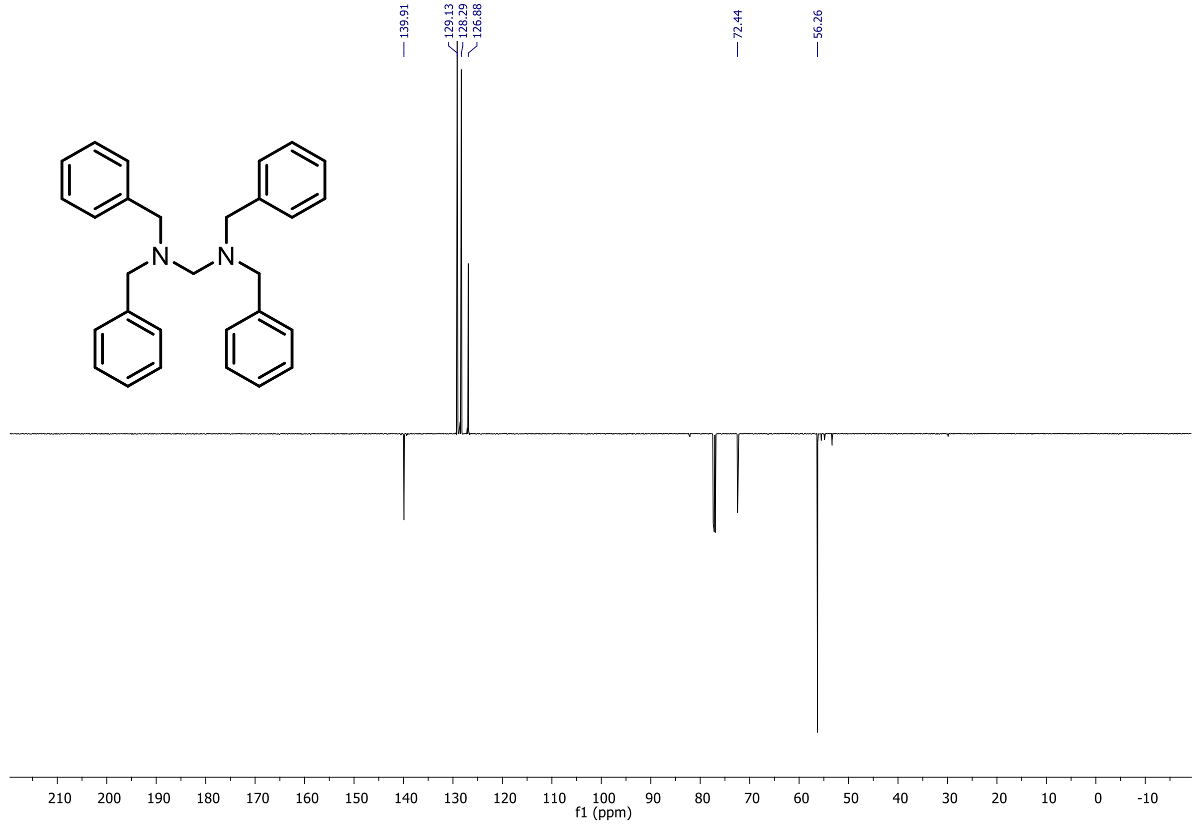
139.91
129.13
128.29
126.88
72.44
56.26
N
N
210 200 190 180 170 160 150 140 130 120 110 100 90 80 70 60 50 40 30 20 10 0 -10
f1 (ppm)

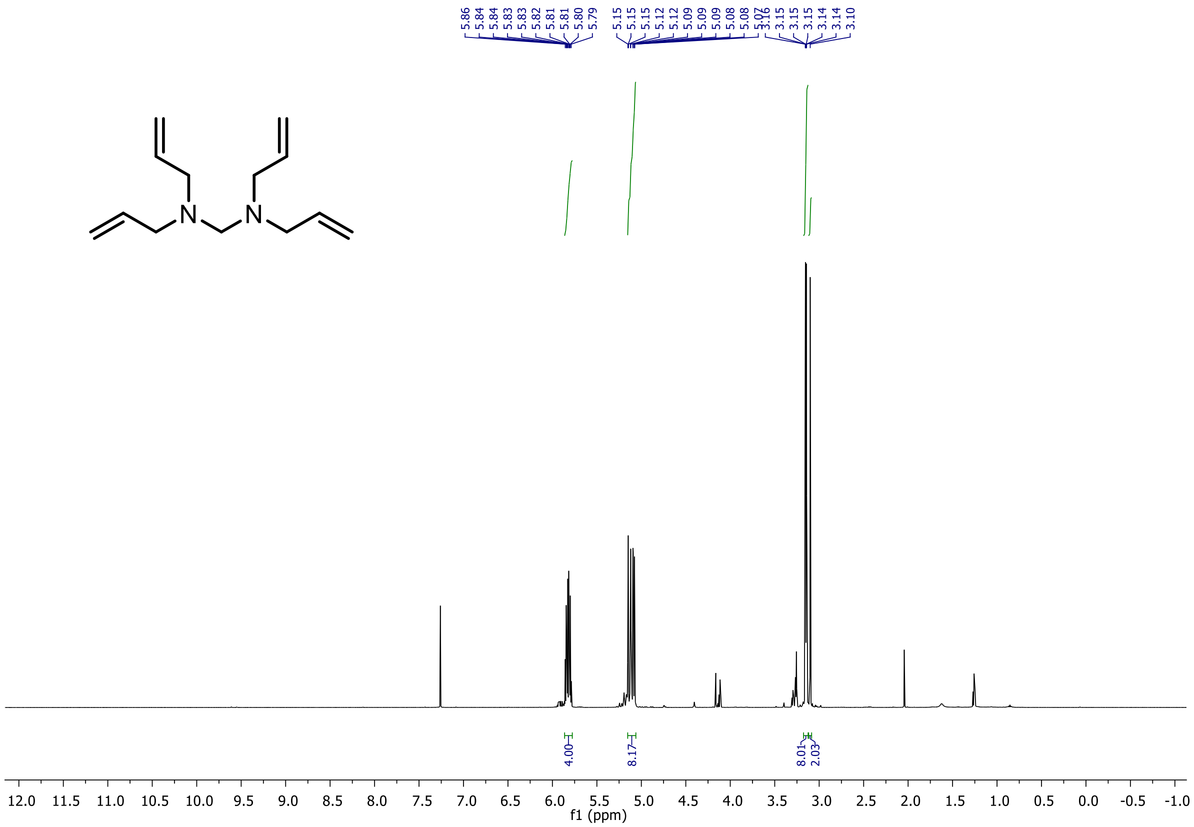

5.86 5.84 5.84 5.83 5.83 5.82 5.81 5.81 5.80 5.79
5.15 5.15 5.15 5.12 5.12 5.09 5.09 5.09 5.08 5.08 5.07
3.16 3.15 3.15 3.15 3.15 3.14 3.14 3.10
4.00
8.17
8.01
2.03
12.0 11.5 11.0 10.5 10.0 9.5 9.0 8.5 8.0 7.5 7.0 6.5 6.0 5.5 5.0 4.5 4.0 3.5 3.0 2.5 2.0 1.5 1.0 0.5 0.0 -0.5 -1.0
f1 (ppm)

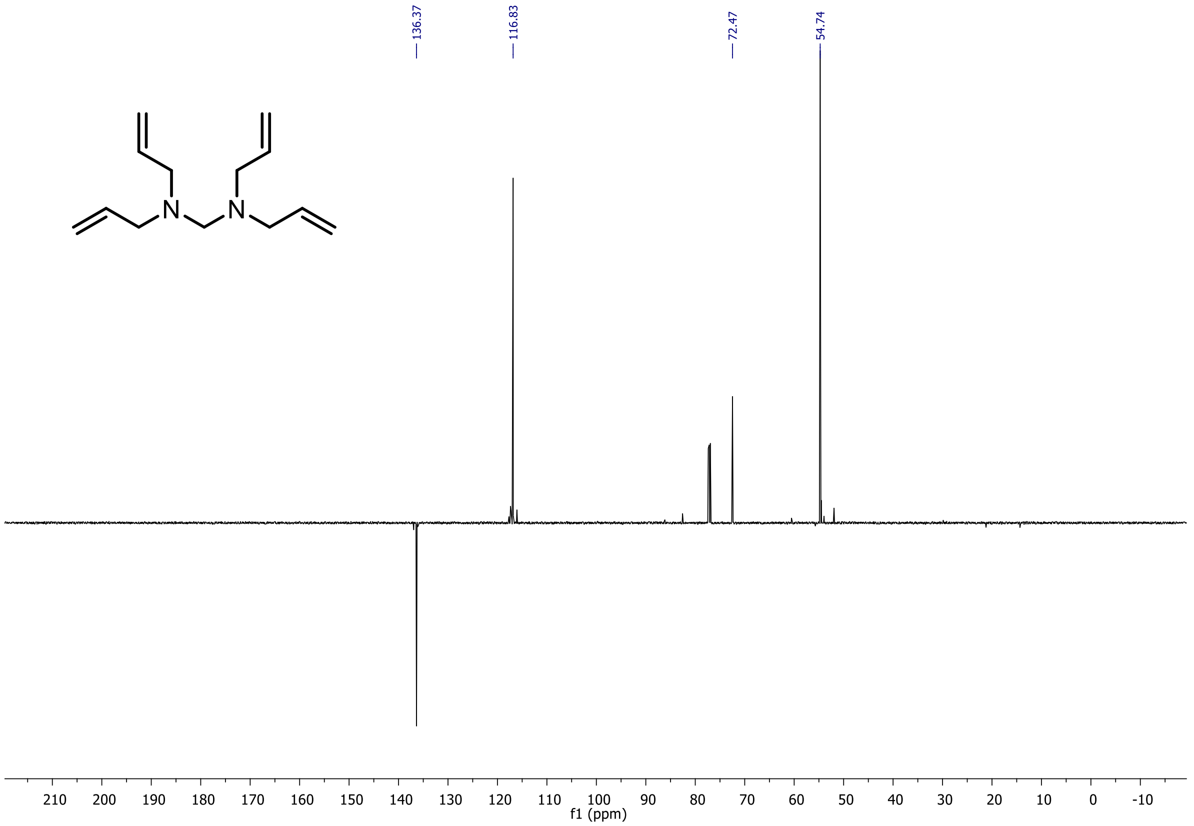

136.37
116.83
72.47
54.74
210 200 190 180 170 160 150 140 130 120 110 100 90 80 70 60 50 40 30 20 10 0 -10
f1 (ppm)

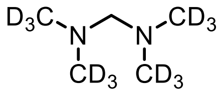

crude

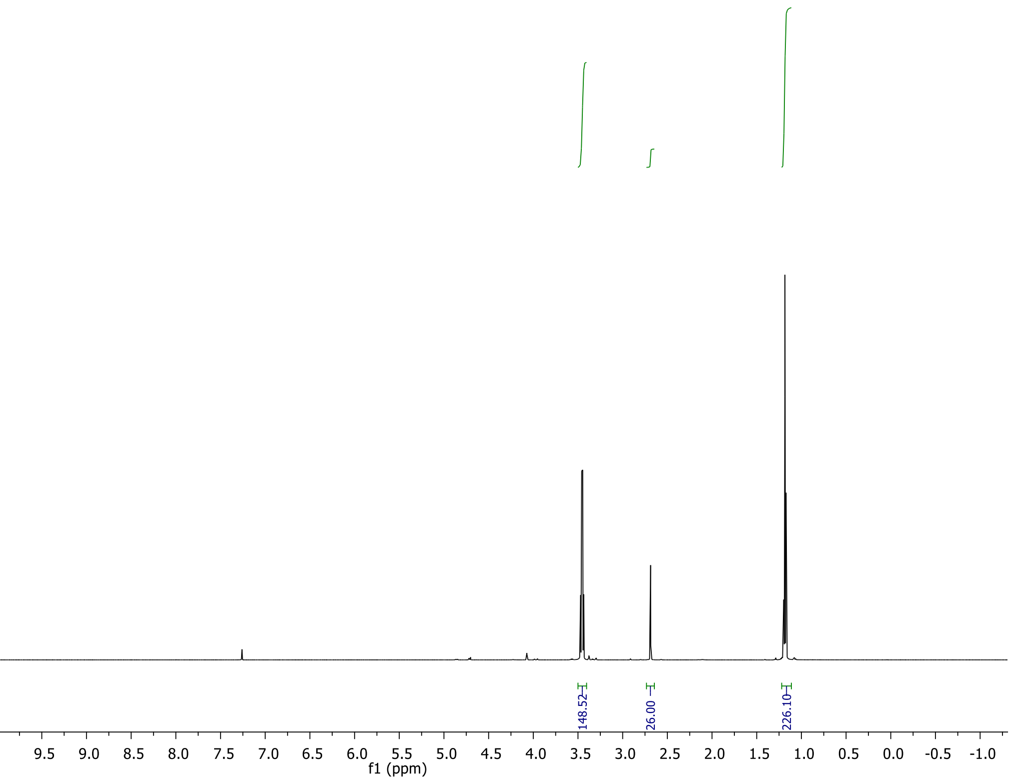

148.52
26.00
226.10
11.5
10.5
9.5
9.0
8.5
8.0
7.5
7.0
6.5
6.0
5.5
5.0
4.5
4.0
3.5
3.0
2.5
2.0
1.5
1.0
0.5
0.0
-0.5
-1.0
f1 (ppm)

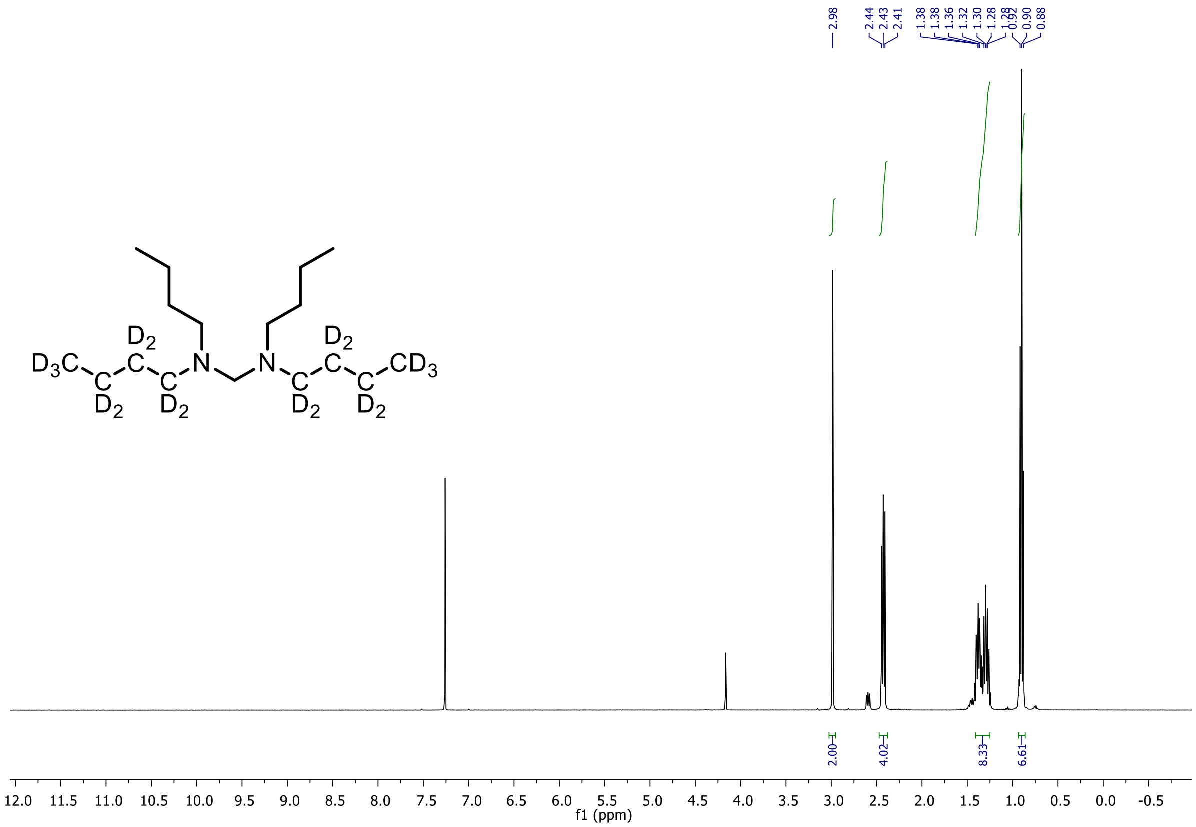
2.98
2.44
2.43
2.41
1.38
1.38
1.36
1.32
1.30
1.28
0.92
0.90
0.88
2.00
4.02
8.33
6.61
f1 (ppm)

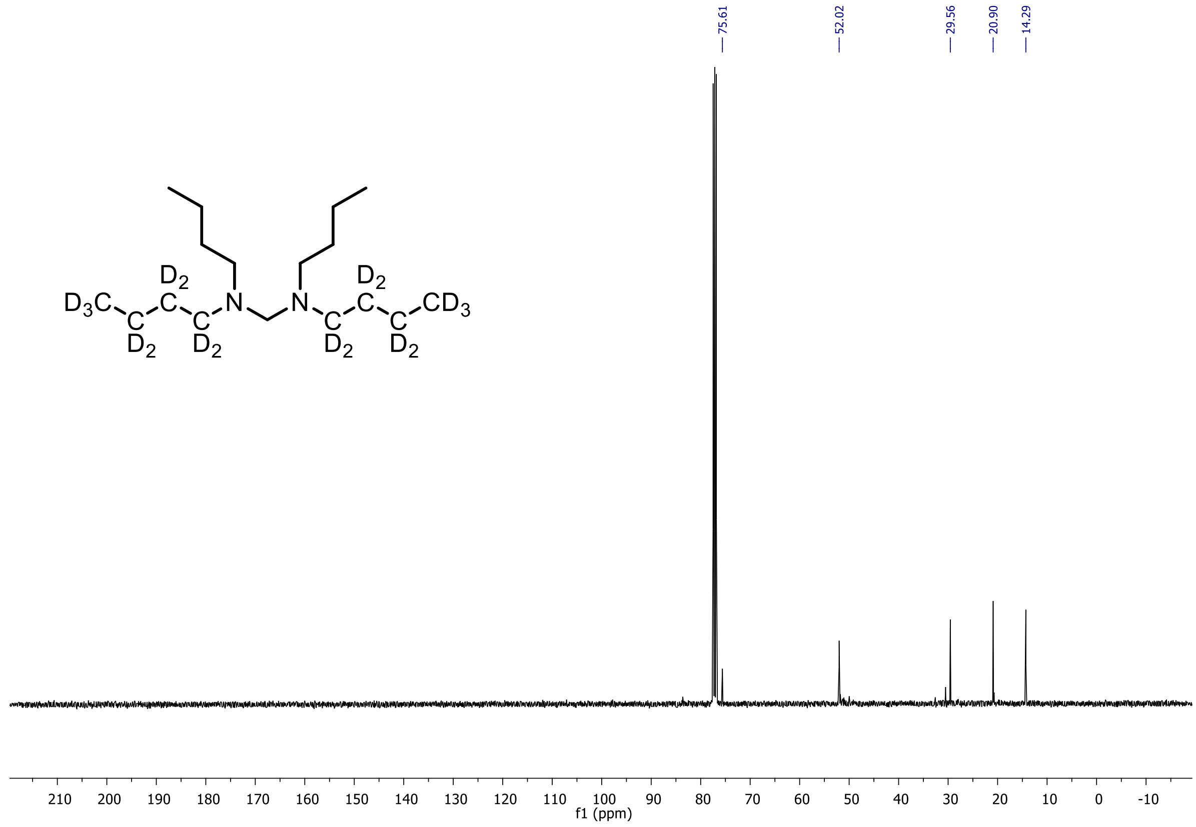
75.61
52.02
29.56
20.90
14.29
f1 (ppm)

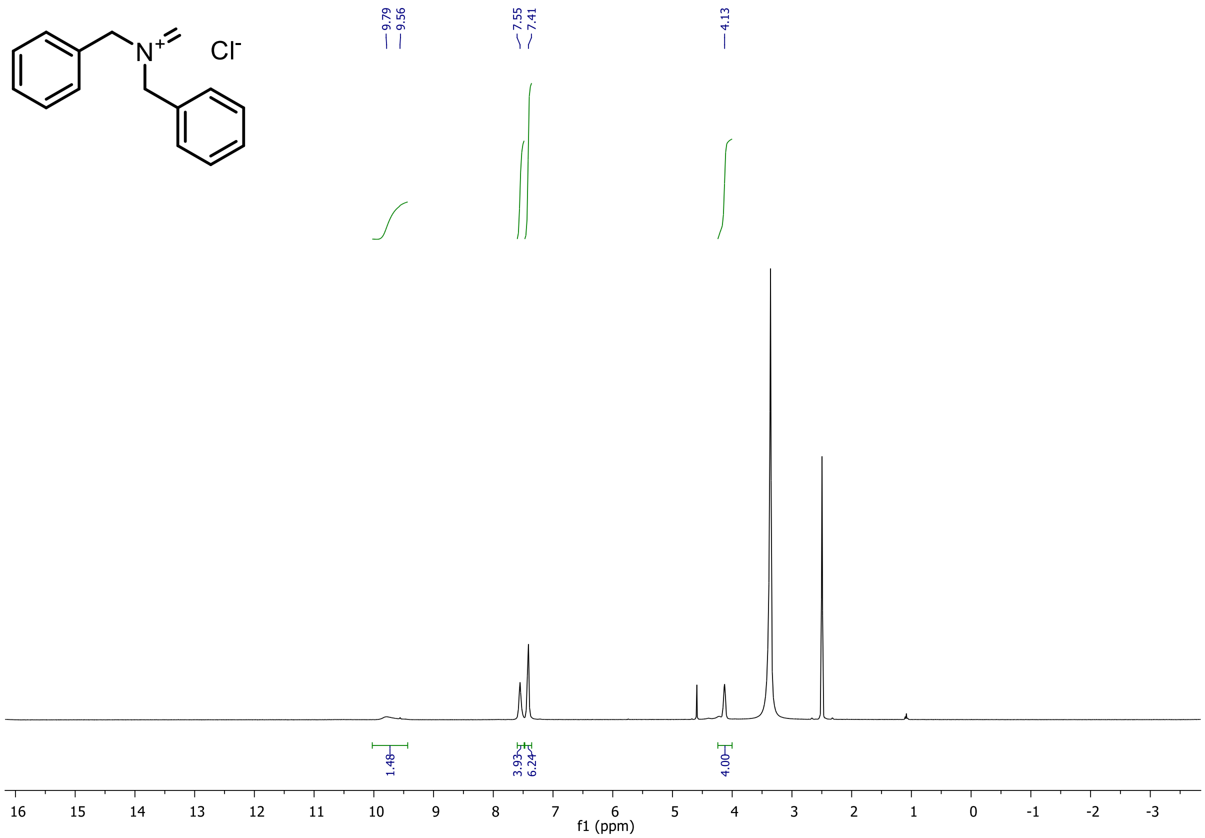
Cl-
9.79
9.56
7.55
7.41
4.13
1.48
3.93
6.24
4.00
16 15 14 13 12 11 10 9 8 7 6 5 4 3 2 1 0 -1 -2 -3
f1 (ppm)

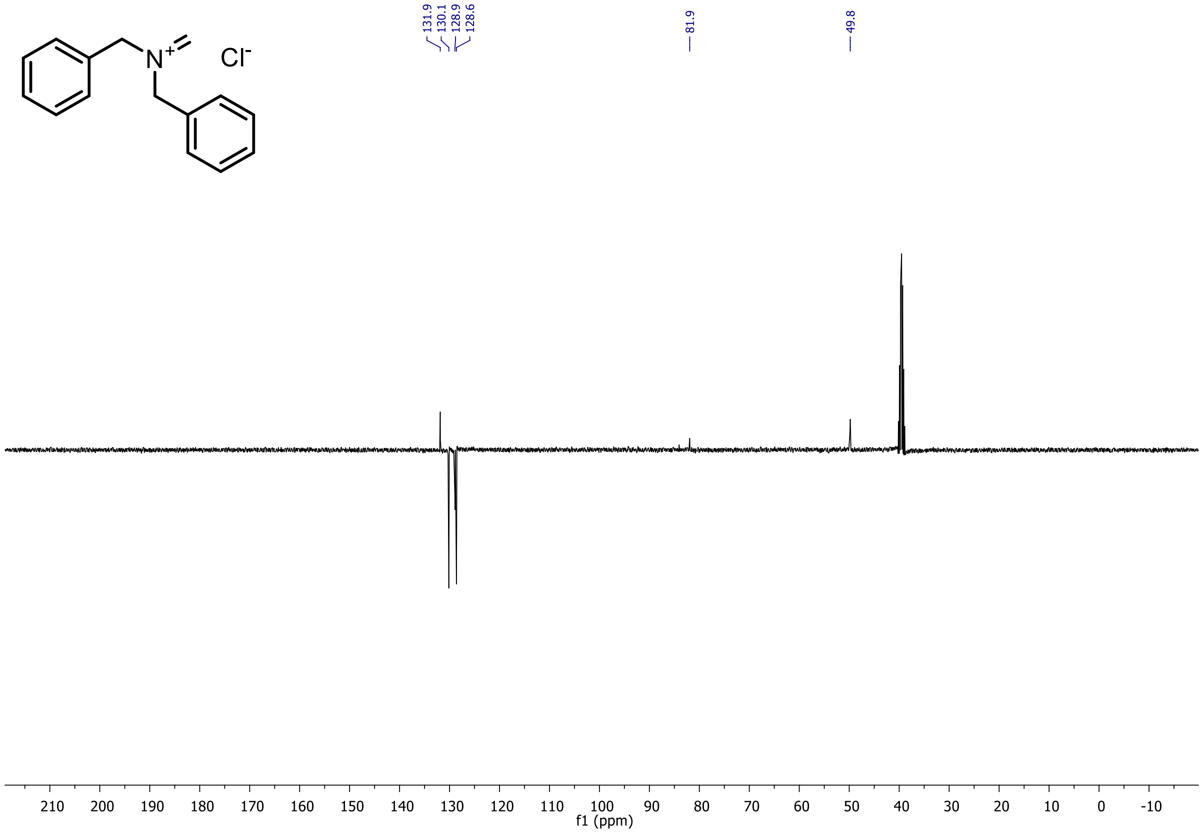
Cl-
131.9
130.1
128.9
128.6
81.9
49.8
210 200 190 180 170 160 150 140 130 120 110 100 90 80 70 60 50 40 30 20 10 0 -10
f1 (ppm)

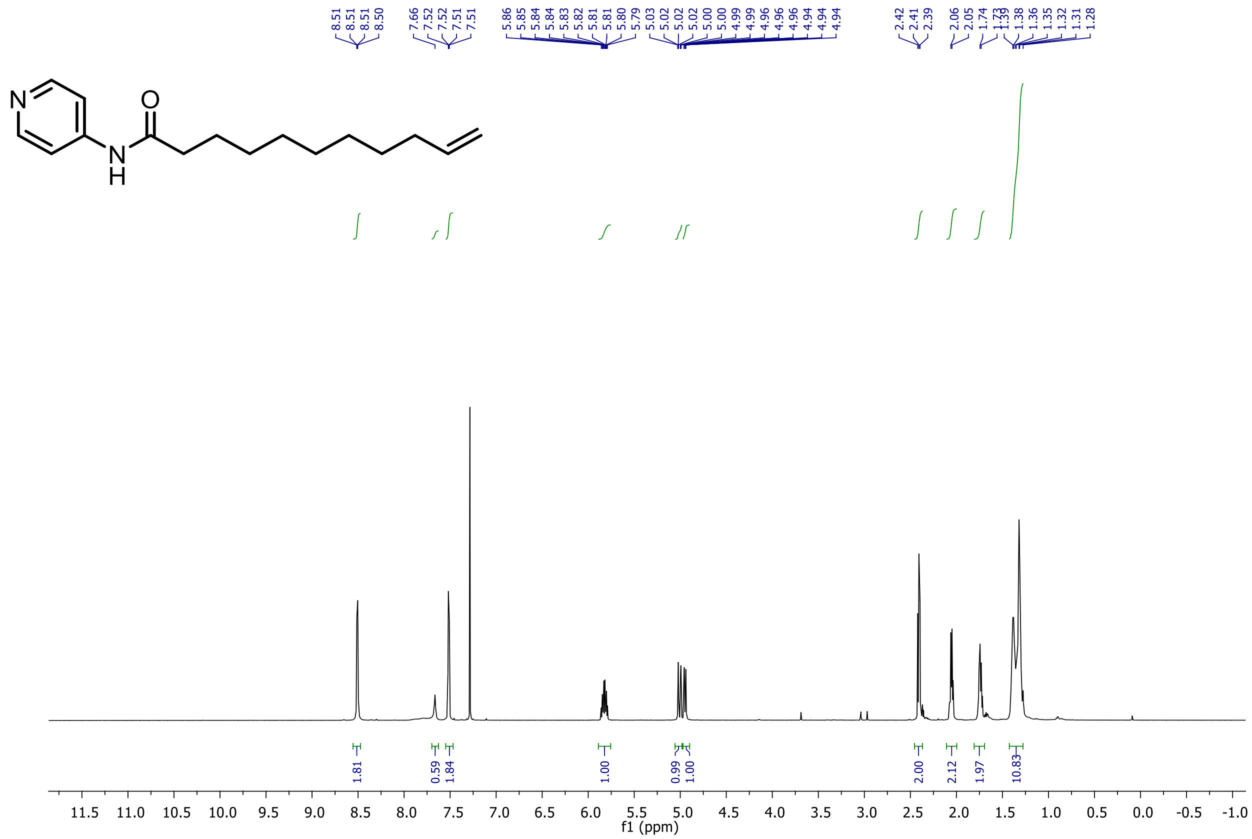

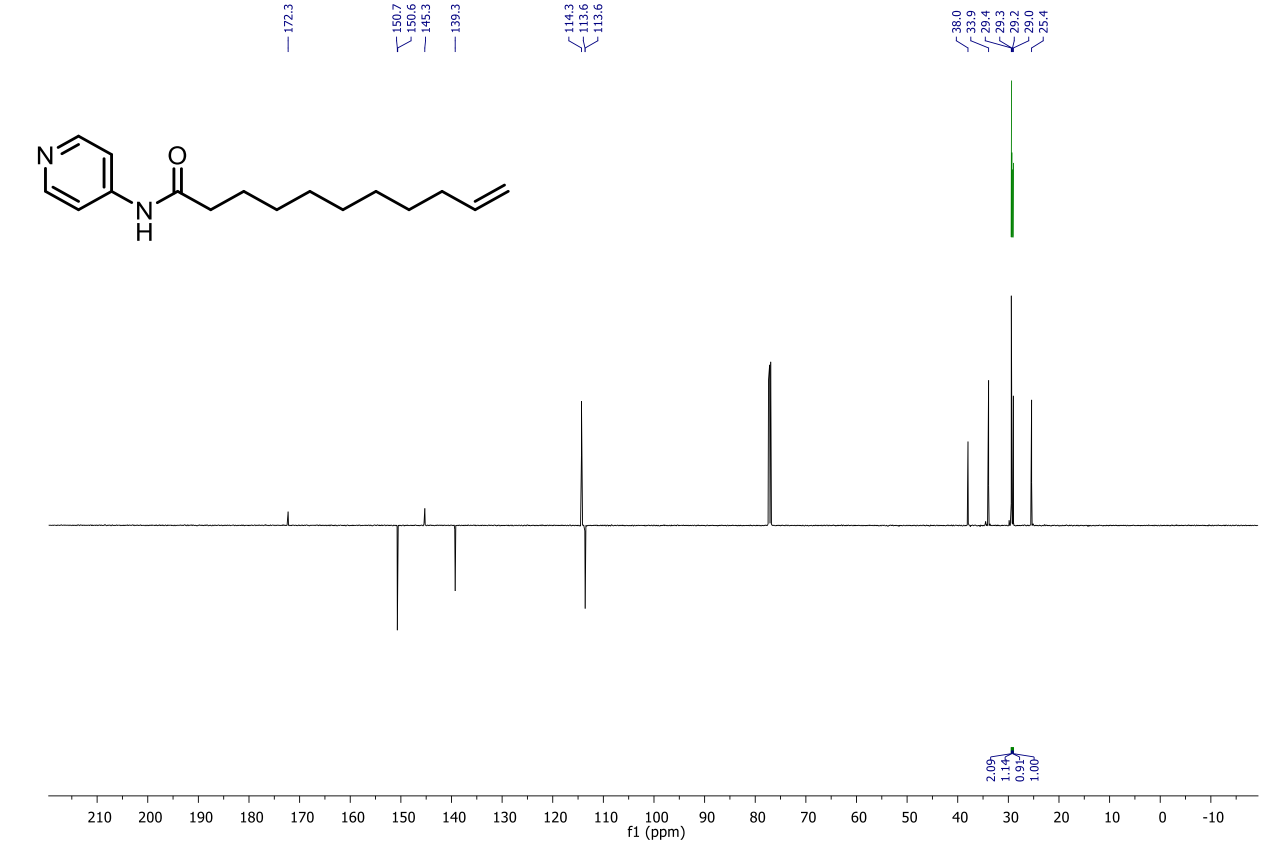

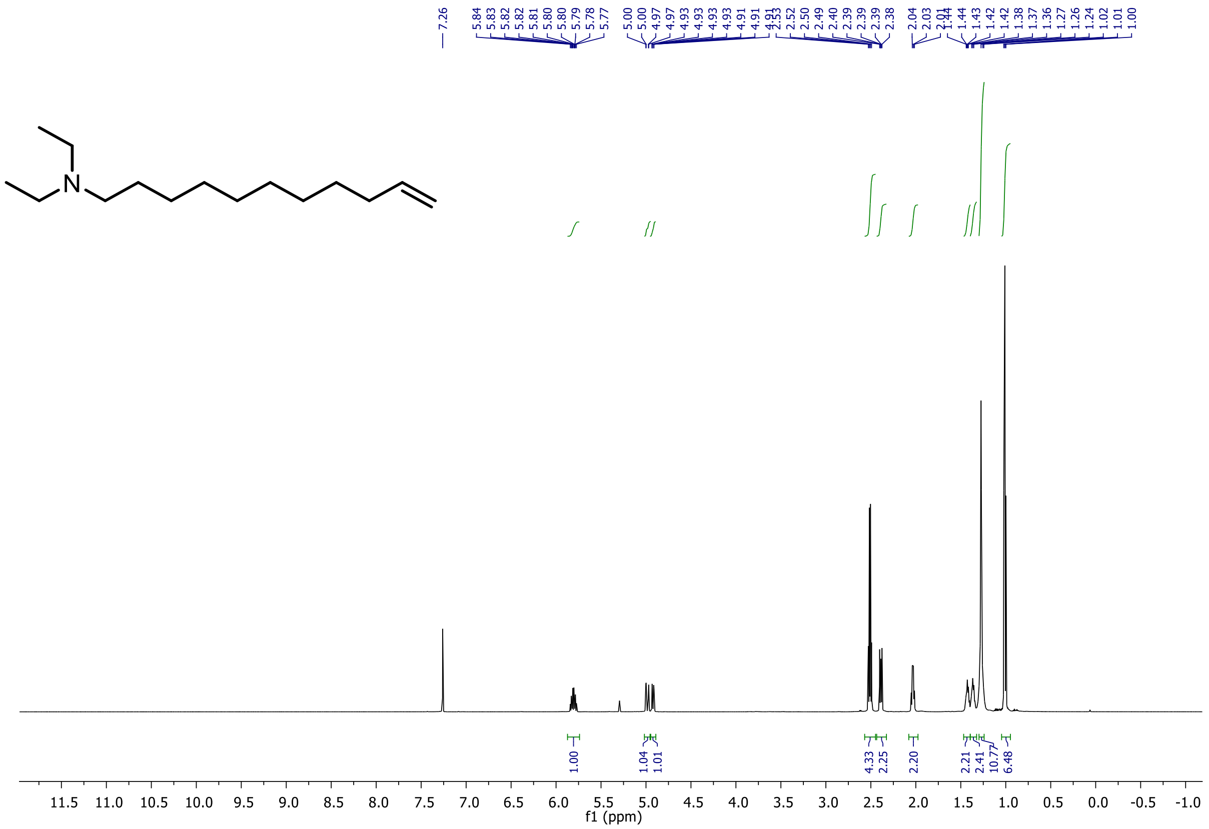

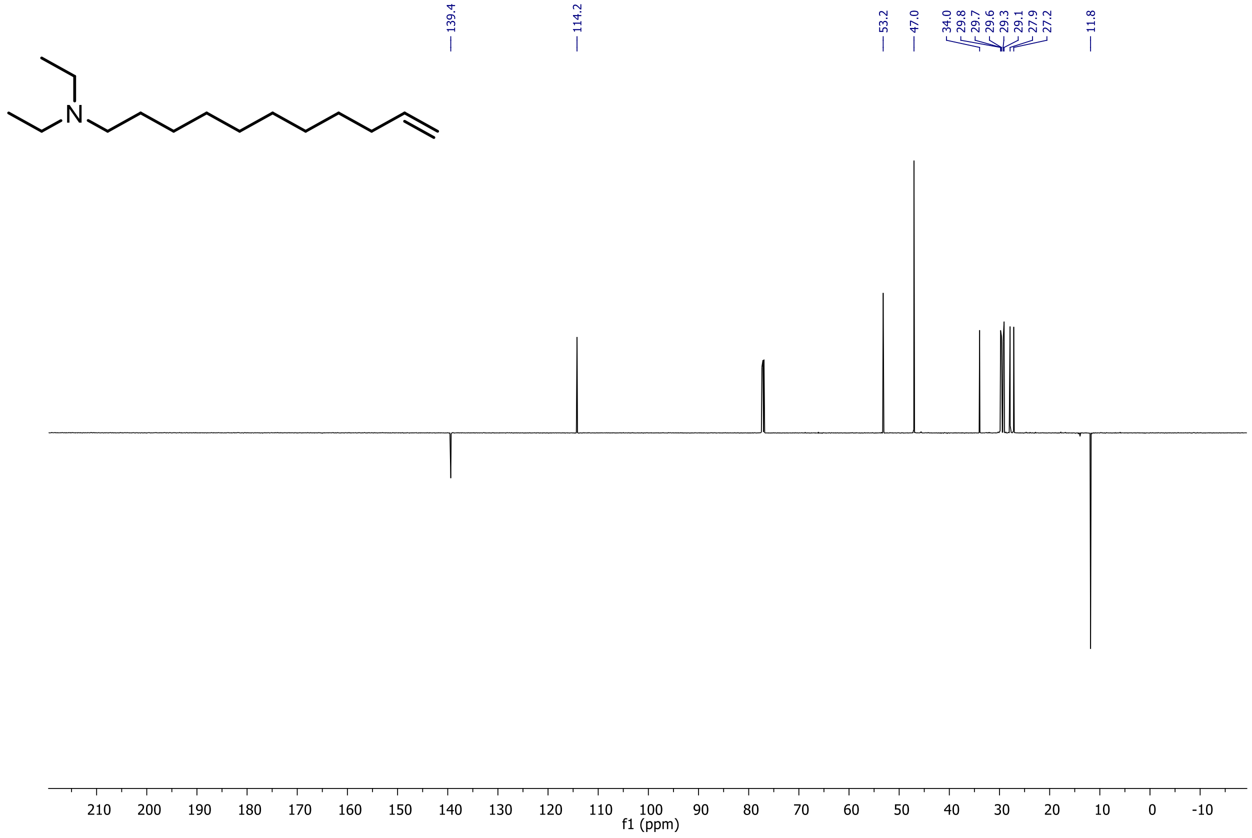

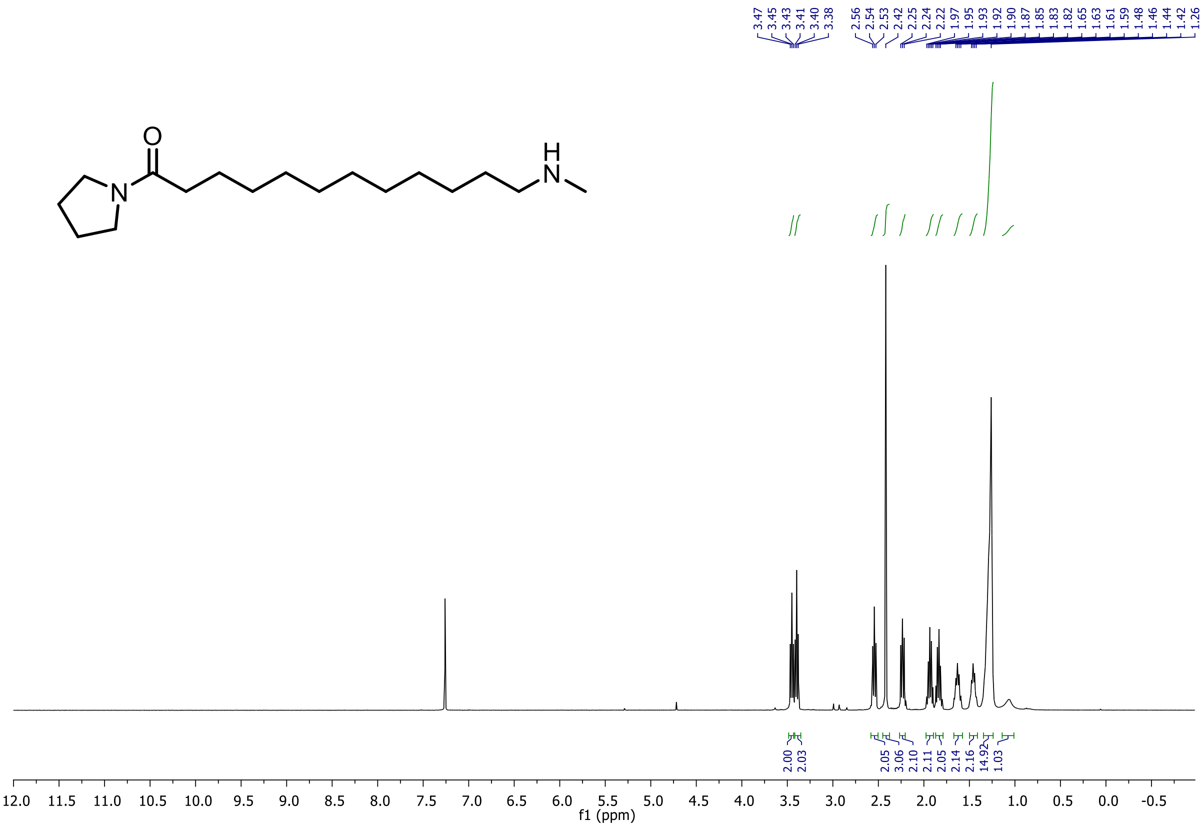

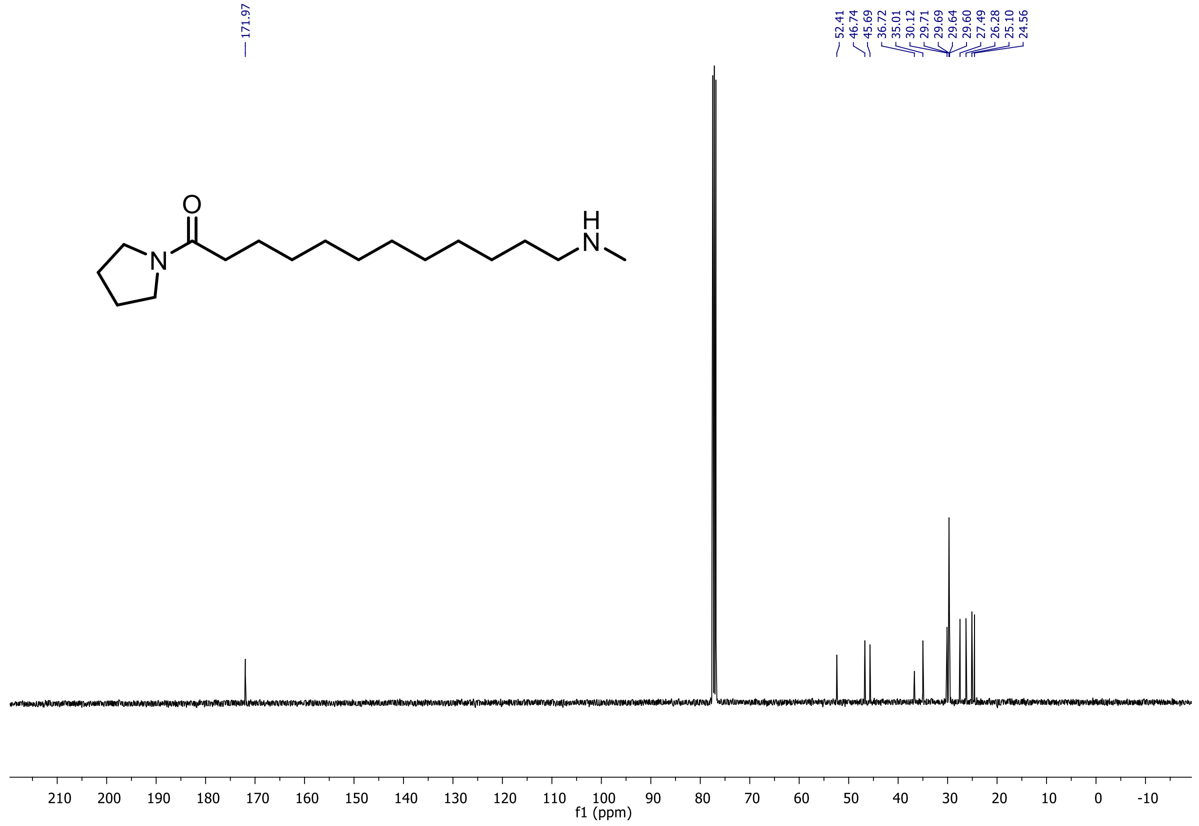

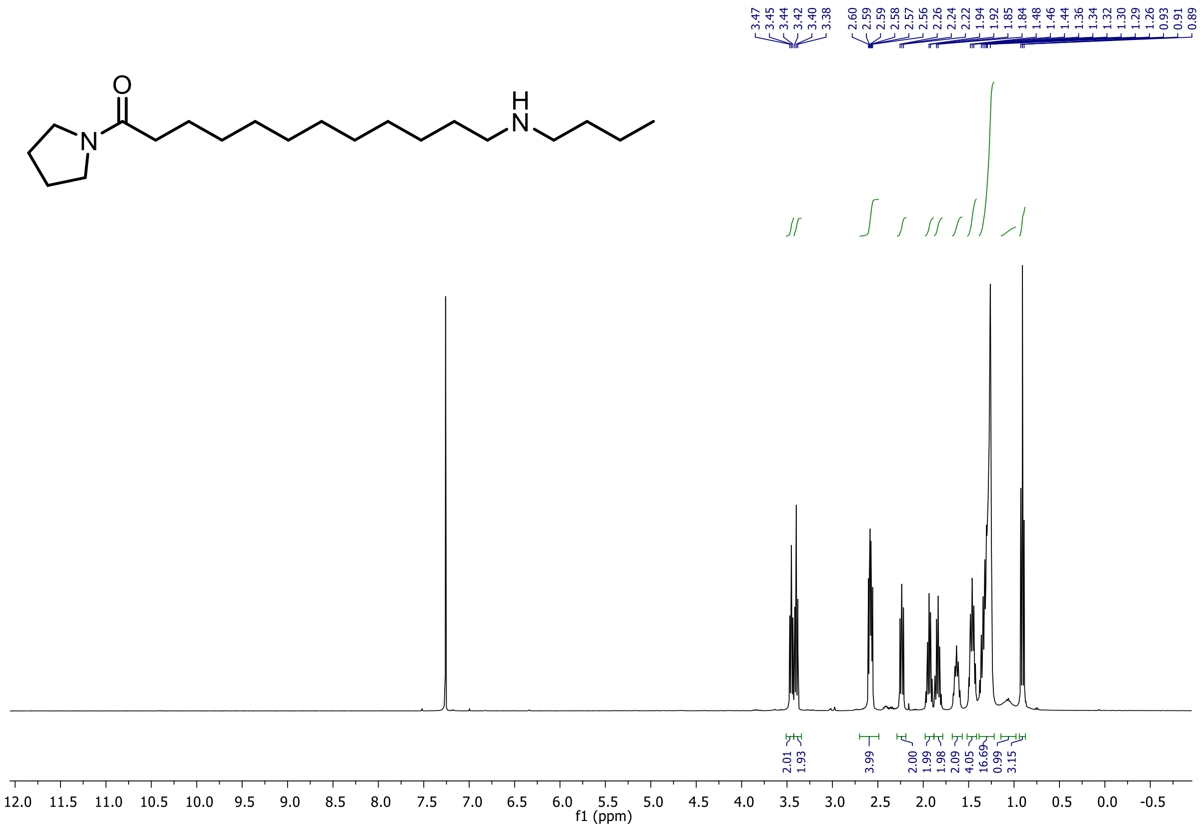

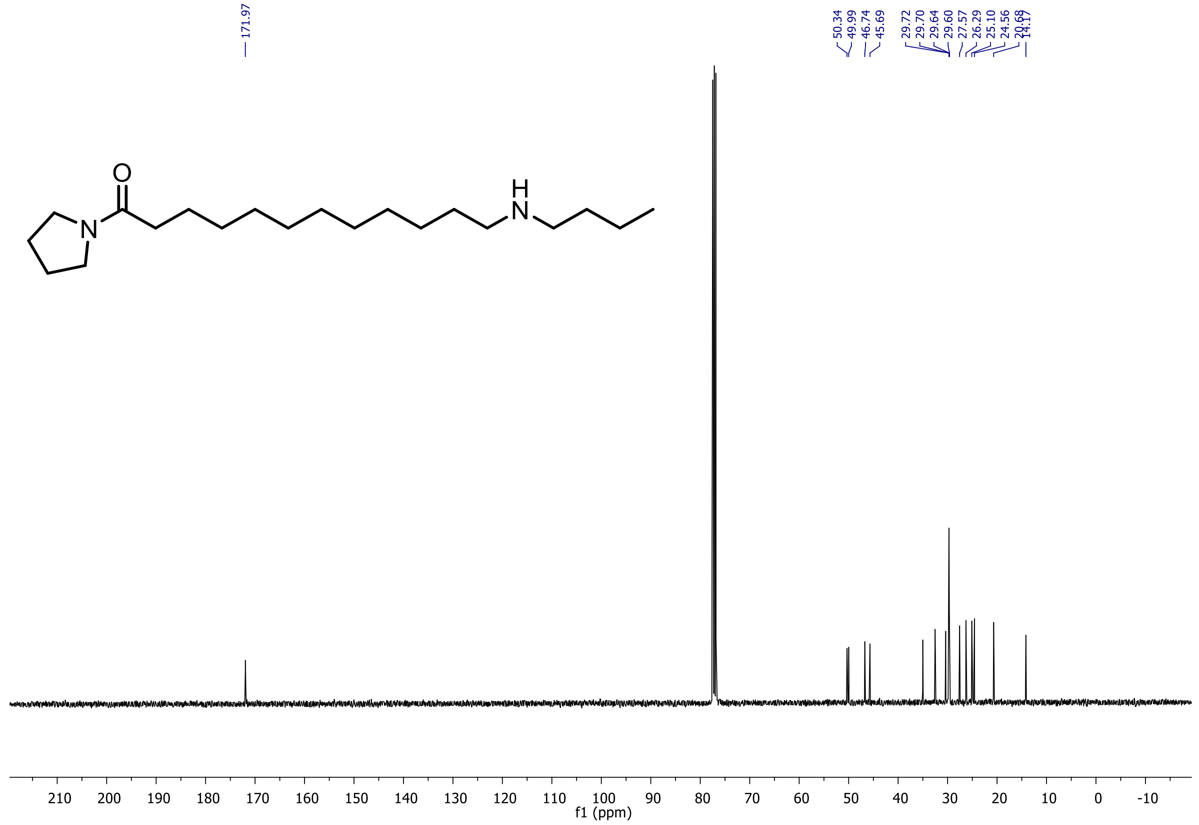

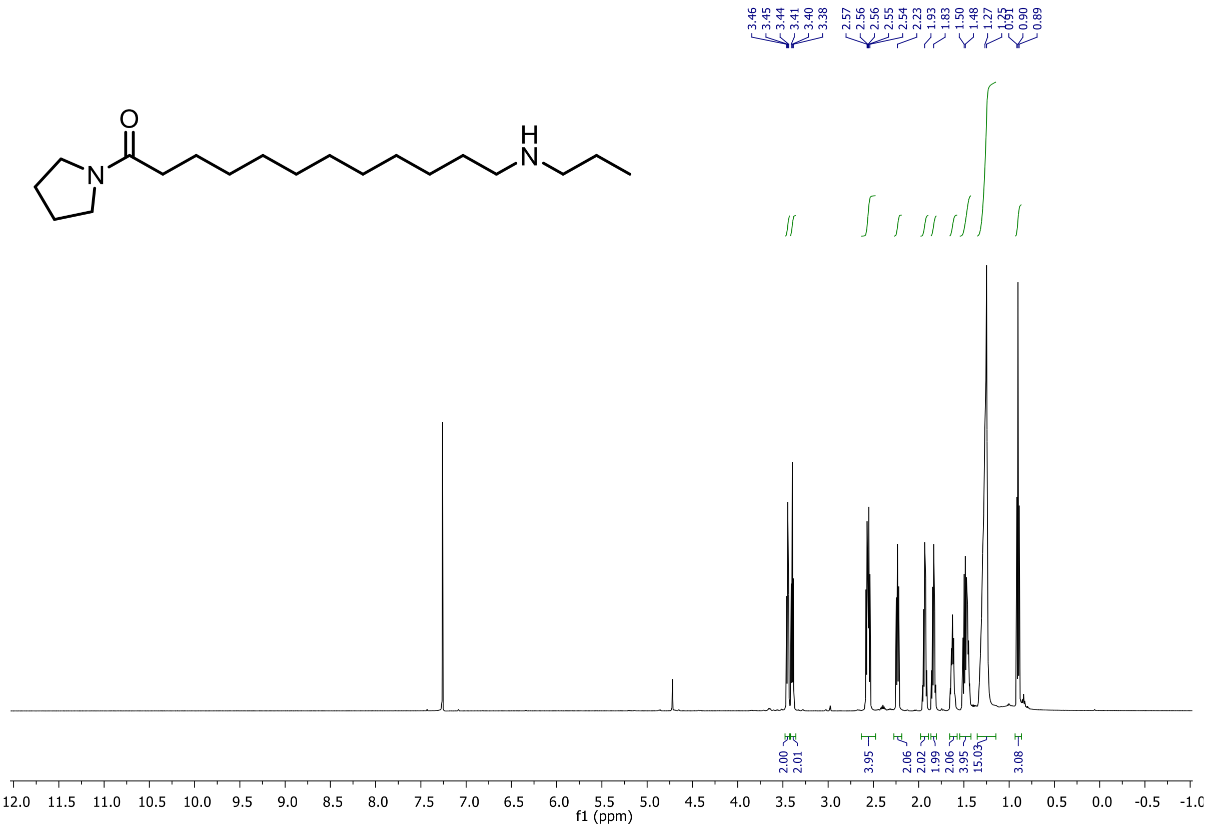
3.46
3.45
3.44
3.41
3.40
3.38
2.57
2.56
2.56
2.55
2.54
2.23
1.93
1.83
1.50
1.48
1.27
0.90
0.89
2.00
2.01
3.95
2.06
2.02
1.99
2.06
3.95
15.03
3.08
O
N
H
N
12.0 11.5 11.0 10.5 10.0 9.5 9.0 8.5 8.0 7.5 7.0 6.5 6.0 5.5 5.0 4.5 4.0 3.5 3.0 2.5 2.0 1.5 1.0 0.5 0.0 -0.5
f1 (ppm)

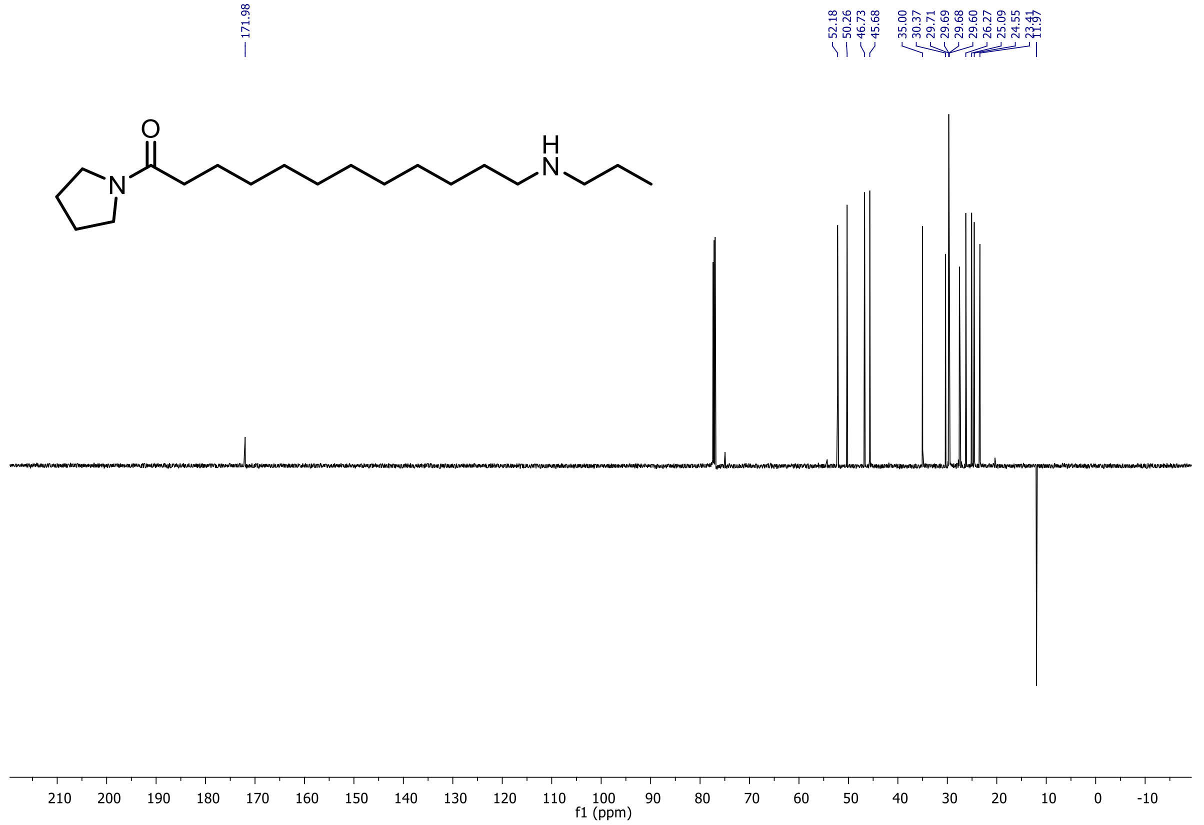
171.98
52.18
50.26
46.73
45.68
35.00
30.37
29.71
29.69
29.68
29.60
26.27
25.09
24.55
O
N
H
N
210 200 190 180 170 160 150 140 130 120 110 100 90 80 70 60 50 40 30 20 10 0 -10
f1 (ppm)

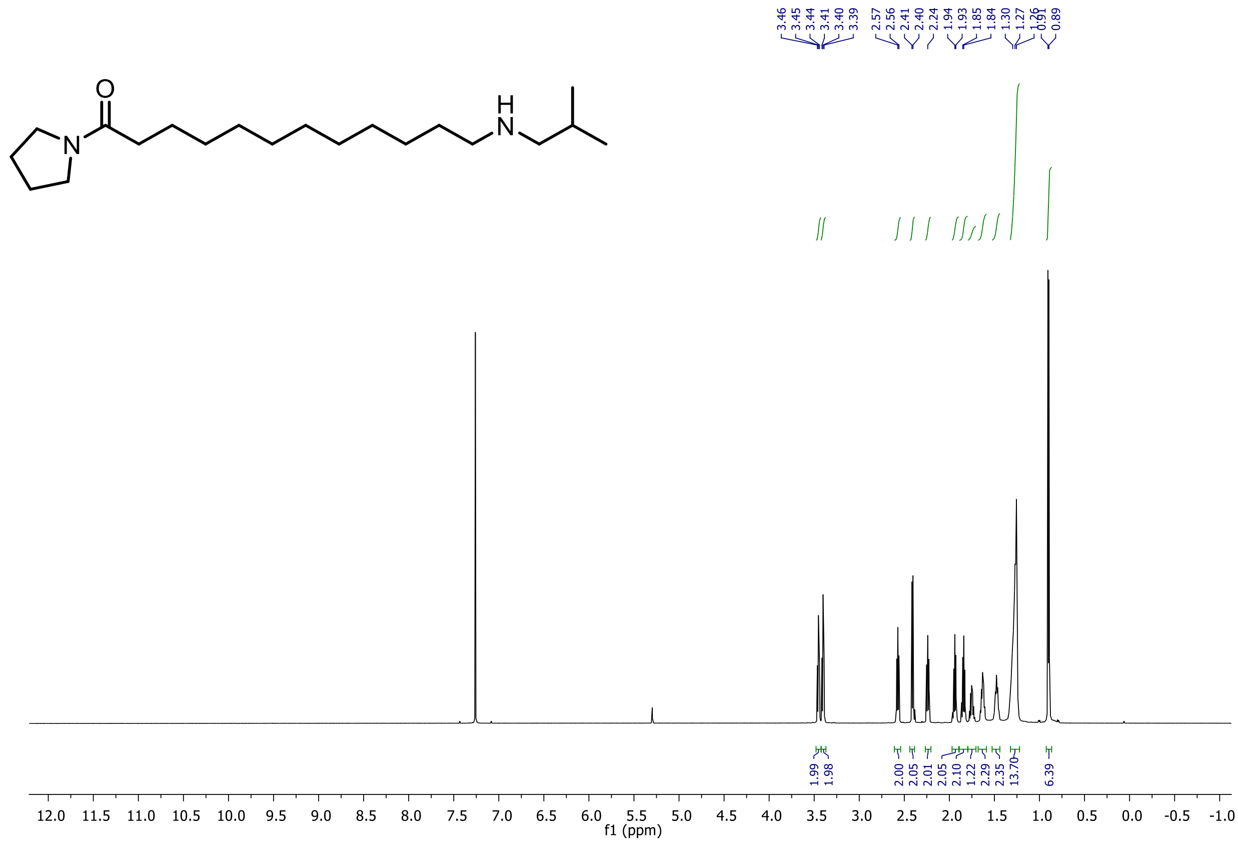

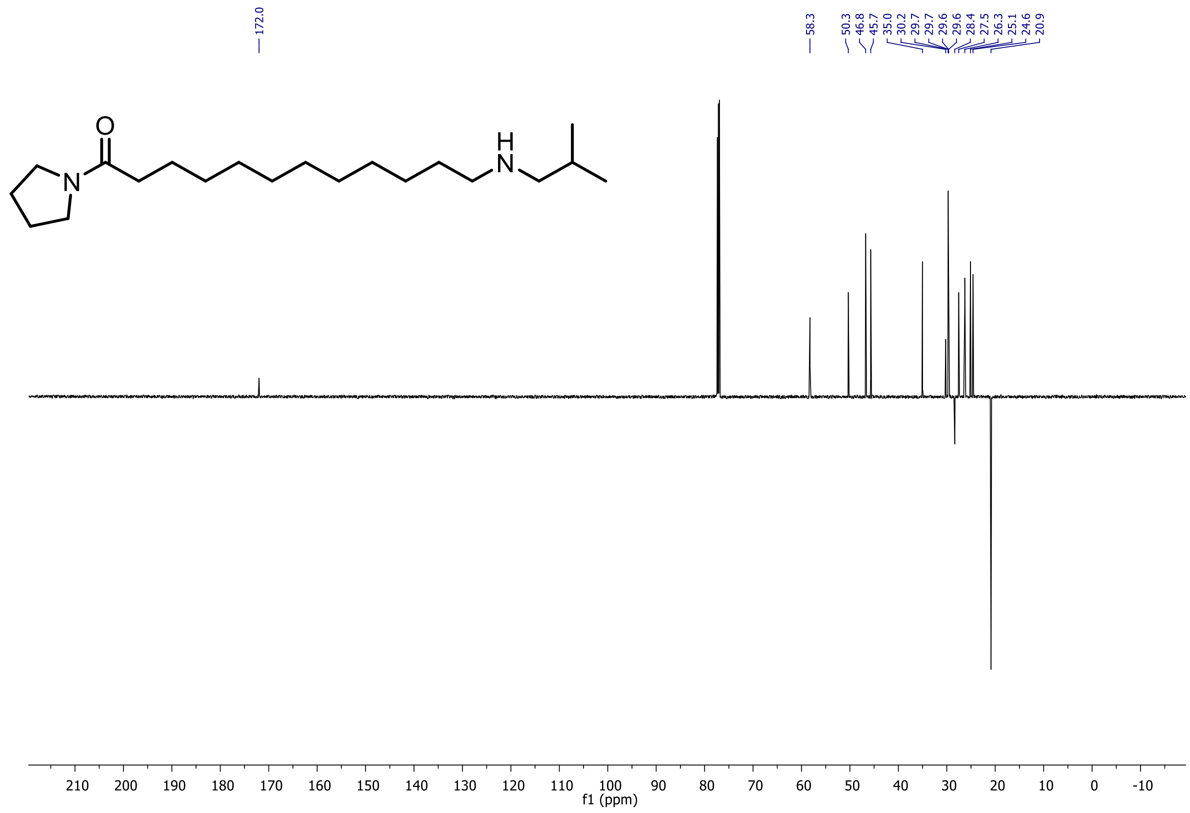

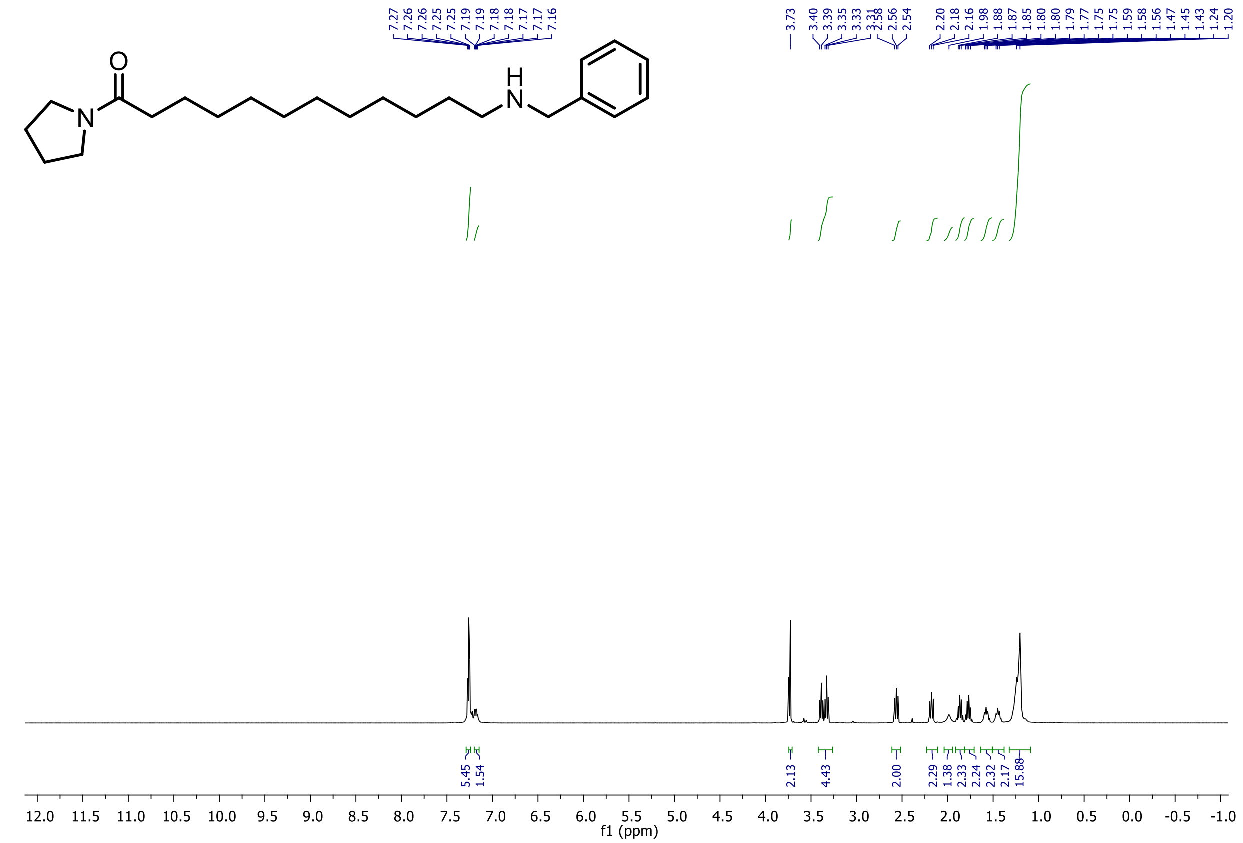

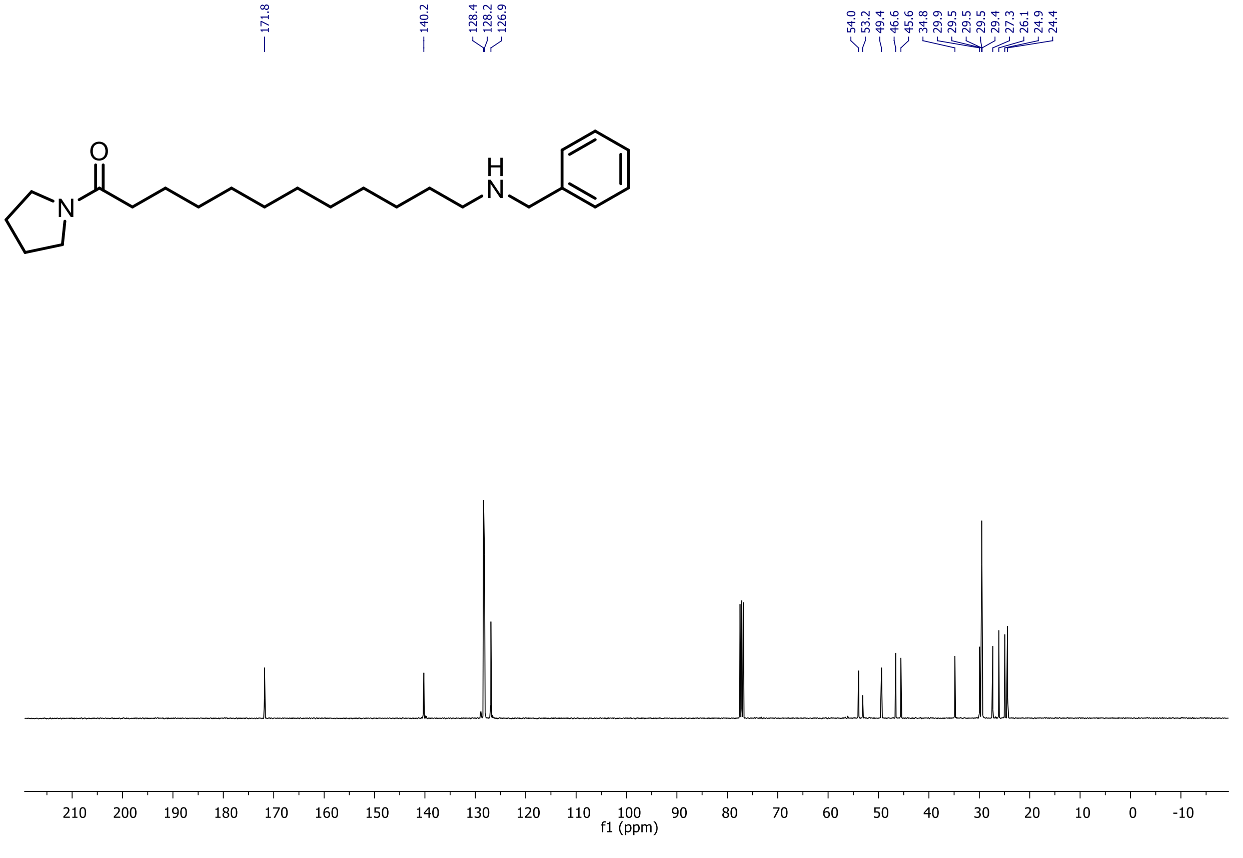

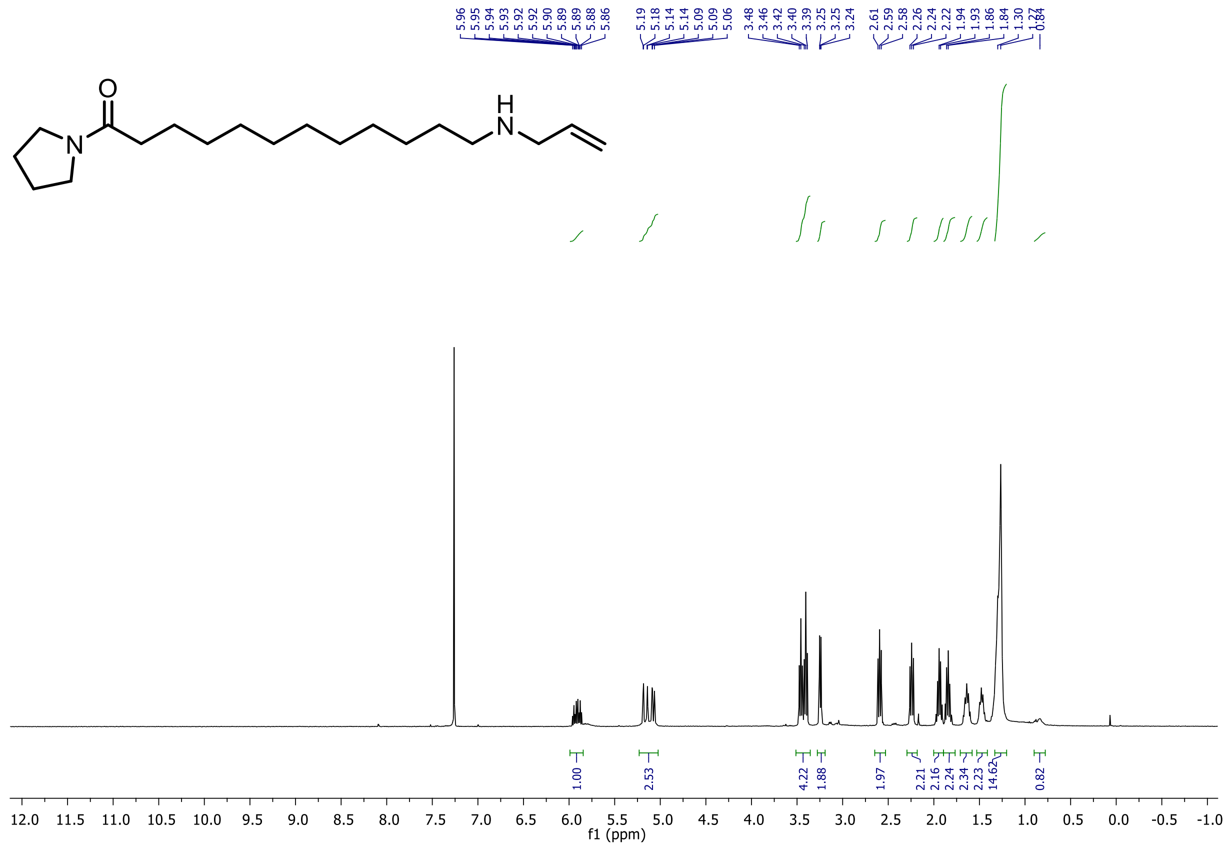
5.96
5.95
5.94
5.93
5.92
5.92
5.90
5.89
5.89
5.88
5.86
5.19
5.18
5.14
5.14
5.09
5.09
5.06
3.48
3.46
3.42
3.40
3.39
3.25
3.25
3.24
2.61
2.59
2.58
2.26
2.24
2.22
1.94
1.93
1.86
1.84
1.30
1.27
0.84
O
H
N
N
1.00
2.53
4.22
1.88
1.97
2.21
2.16
2.24
2.34
2.23
14.62
0.82
f1 (ppm)

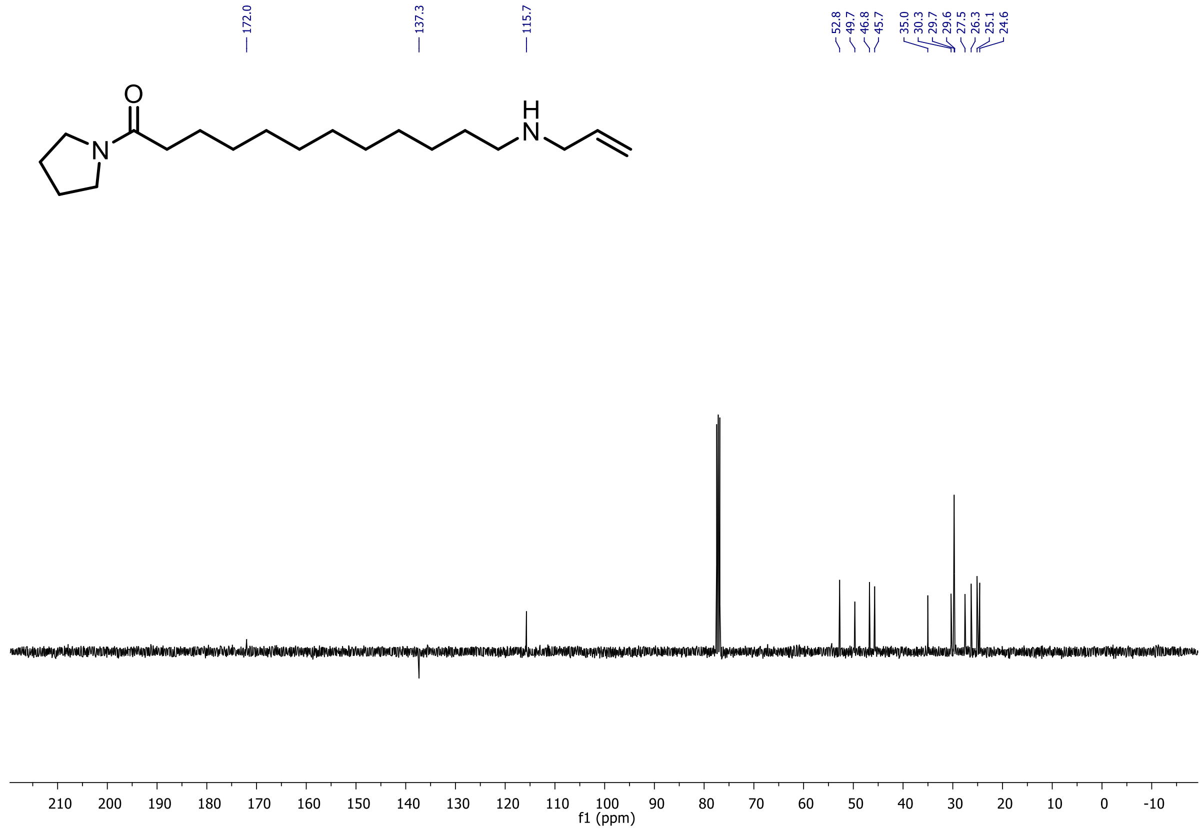
172.0
137.3
115.7
52.8
49.7
46.8
45.7
35.0
30.3
29.7
29.6
27.5
26.3
25.1
24.6
O
H
N
N
f1 (ppm)

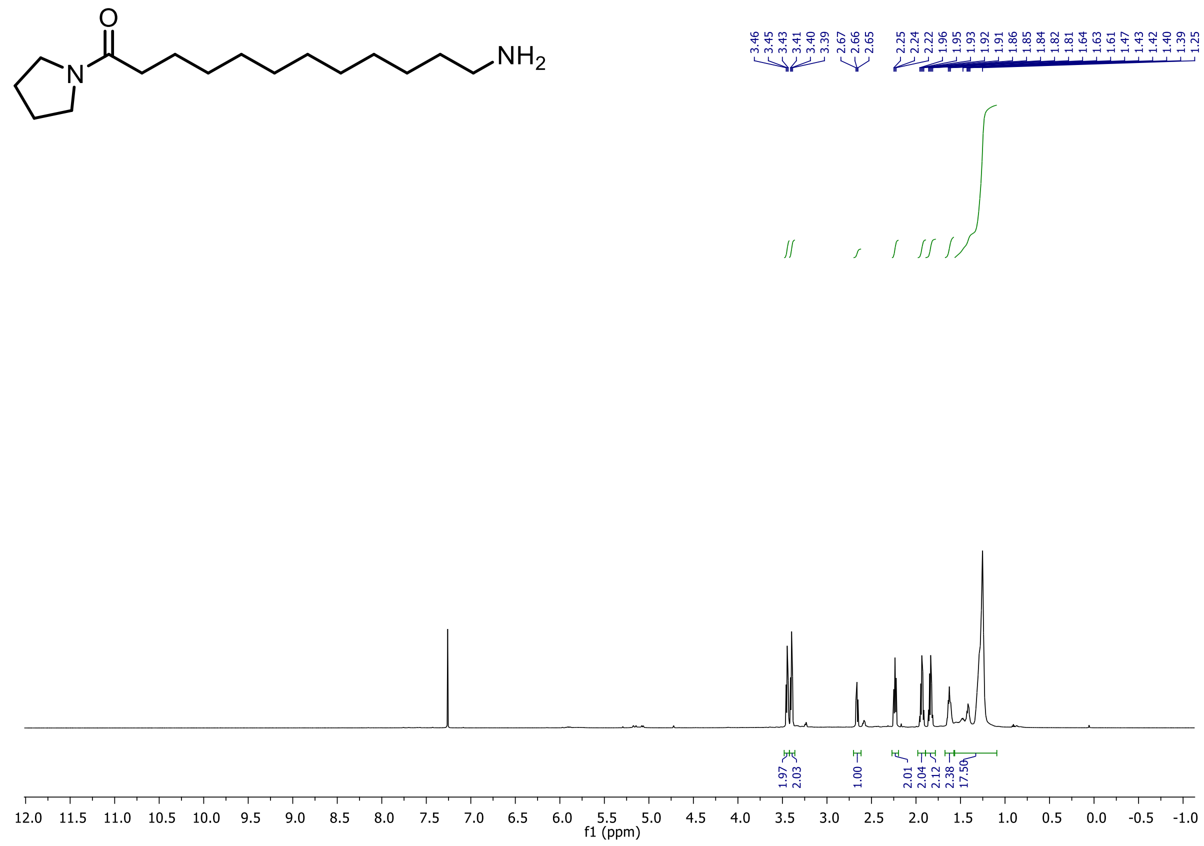

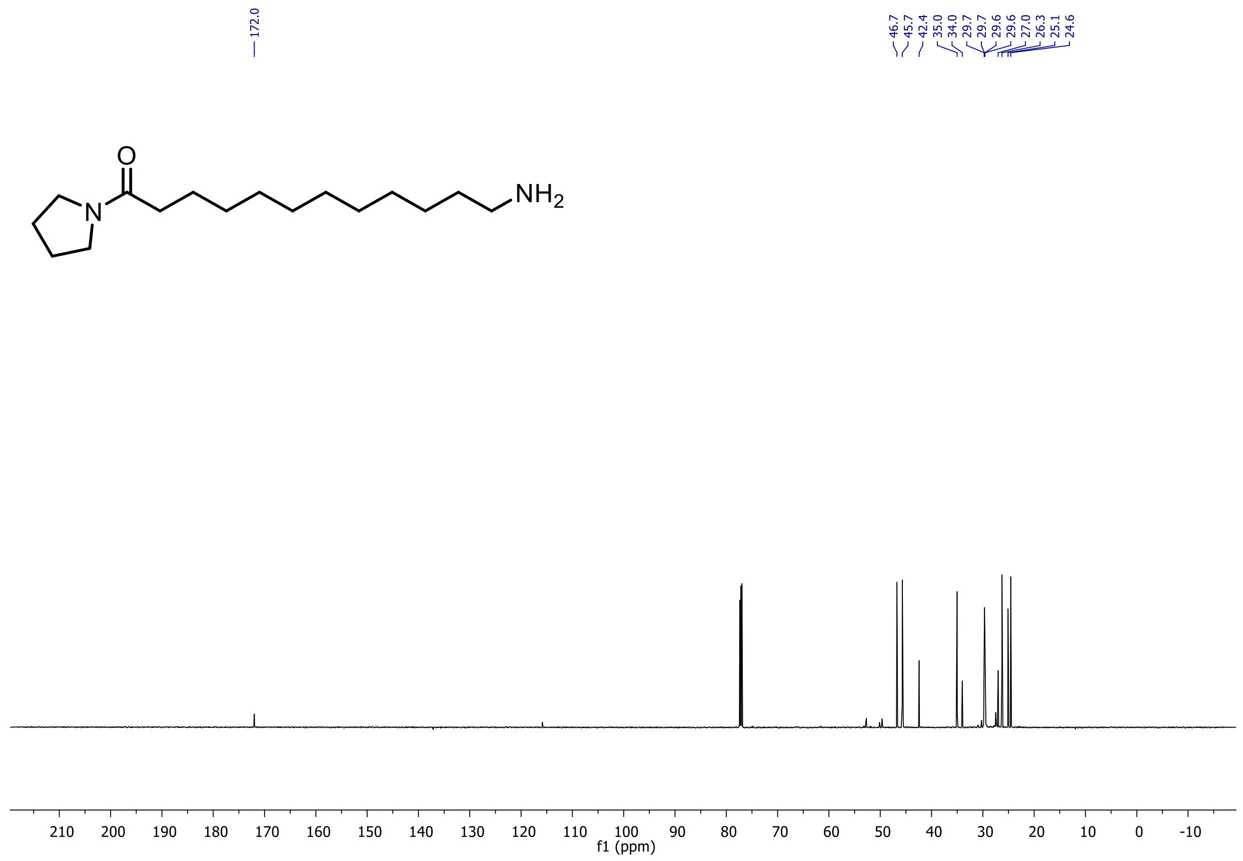

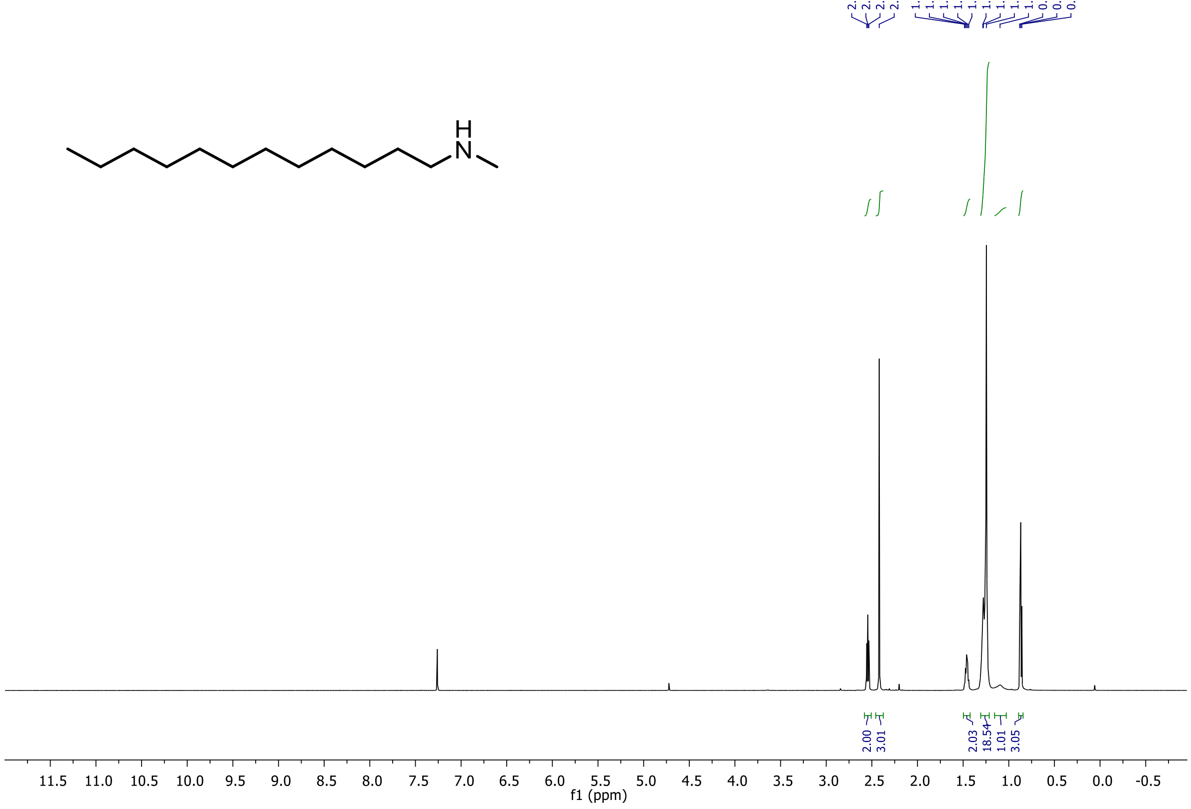
2.56
2.54
2.53
2.42
1.49
1.47
1.46
1.45
1.44
1.28
1.28
1.25
1.10
0.88
0.87
0.86
H
N
2.00
3.01
2.03
18.54
1.01
3.05
11.5 11.0 10.5 10.0 9.5 9.0 8.5 8.0 7.5 7.0 6.5 6.0 5.5 5.0 4.5 4.0 3.5 3.0 2.5 2.0 1.5 1.0 0.5 0.0 -0.5
f1 (ppm)

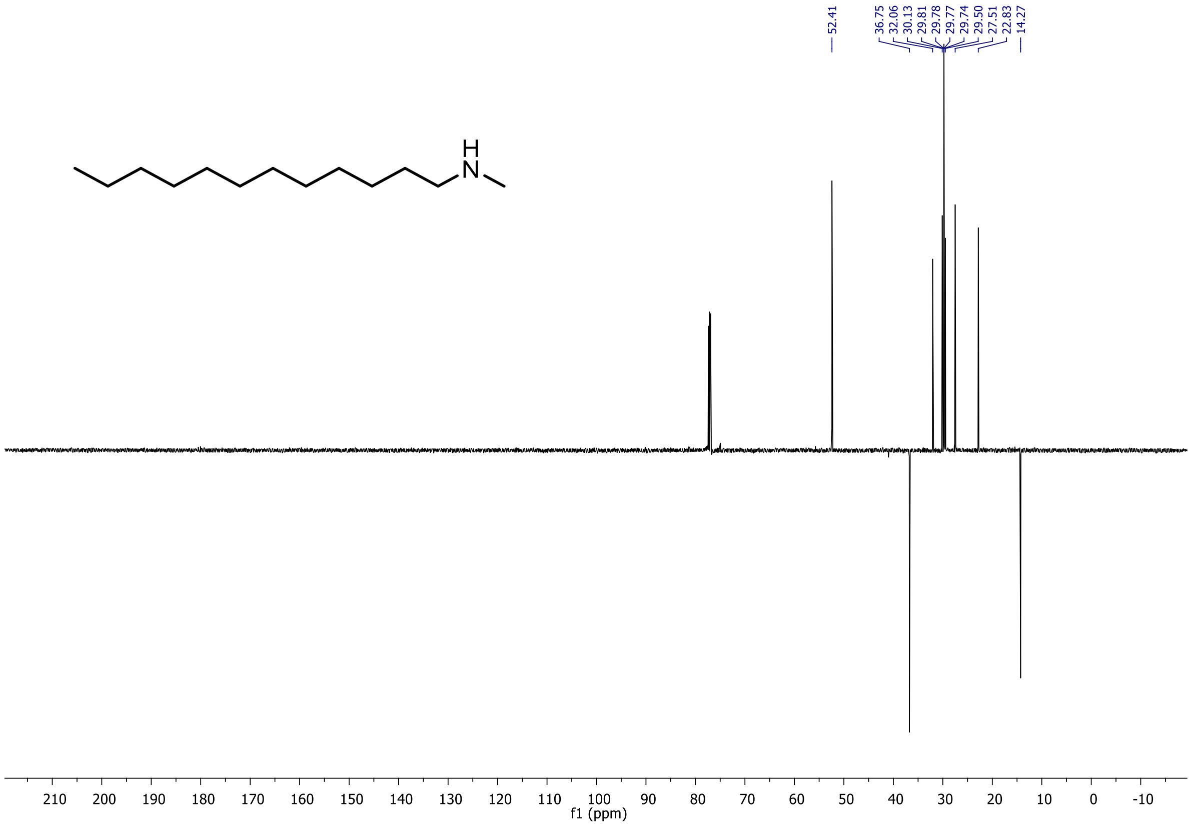
52.41
36.75
32.06
30.13
29.81
29.78
29.77
29.74
29.50
27.51
22.83
14.27
H
N
210 200 190 180 170 160 150 140 130 120 110 100 90 80 70 60 50 40 30 20 10 0 -10
f1 (ppm)

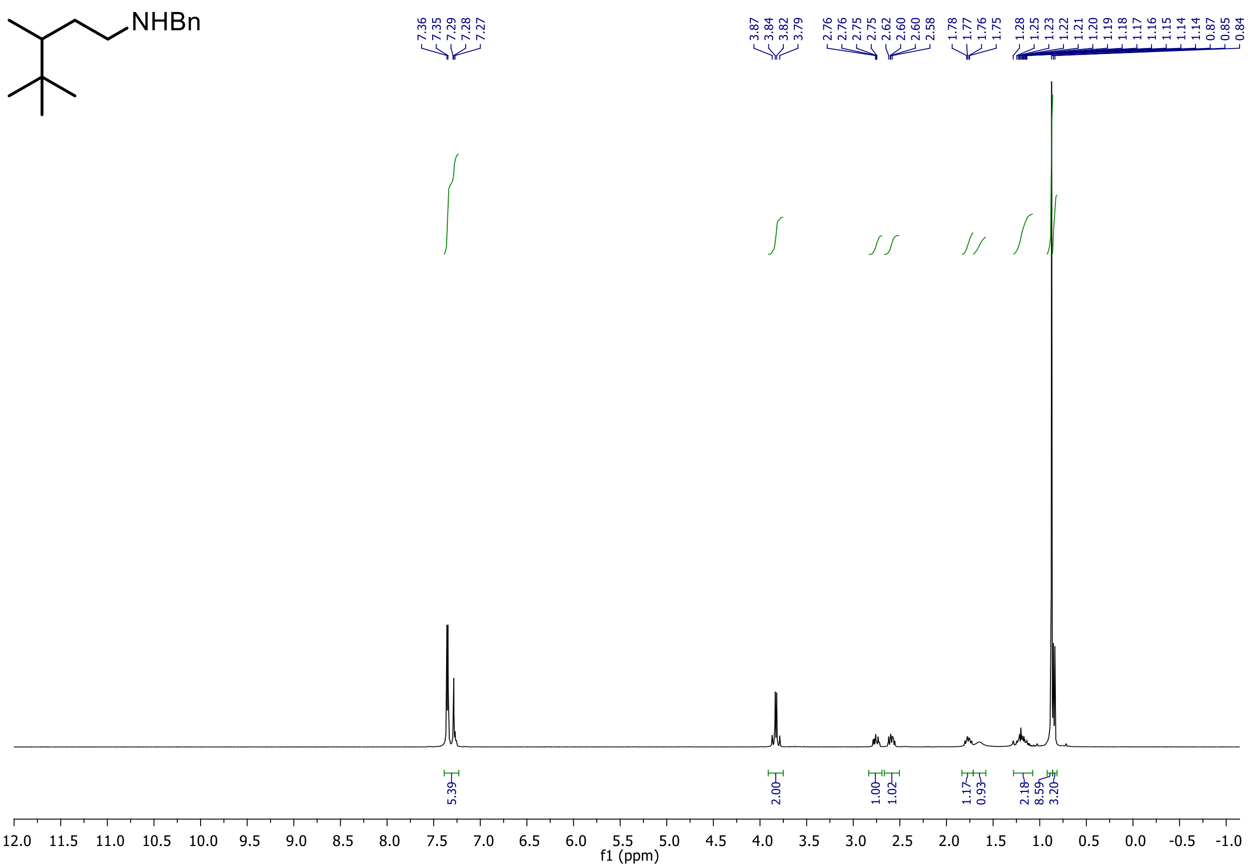
NHBn
7.36
7.35
7.29
7.28
7.27
3.87
3.84
3.82
3.79
2.76
2.76
2.75
2.75
2.62
2.60
2.60
2.58
1.78
1.77
1.76
1.75
1.28
1.25
1.23
1.22
1.21
1.20
1.19
1.18
1.17
1.16
1.15
1.14
1.14
0.87
0.85
0.84
5.39
2.00
1.00
1.02
1.17
0.93
2.18
8.59
3.20
12.0 11.5 11.0 10.5 10.0 9.5 9.0 8.5 8.0 7.5 7.0 6.5 6.0 5.5 5.0 4.5 4.0 3.5 3.0 2.5 2.0 1.5 1.0 0.5 0.0 -0.5 -1.0
f1 (ppm)

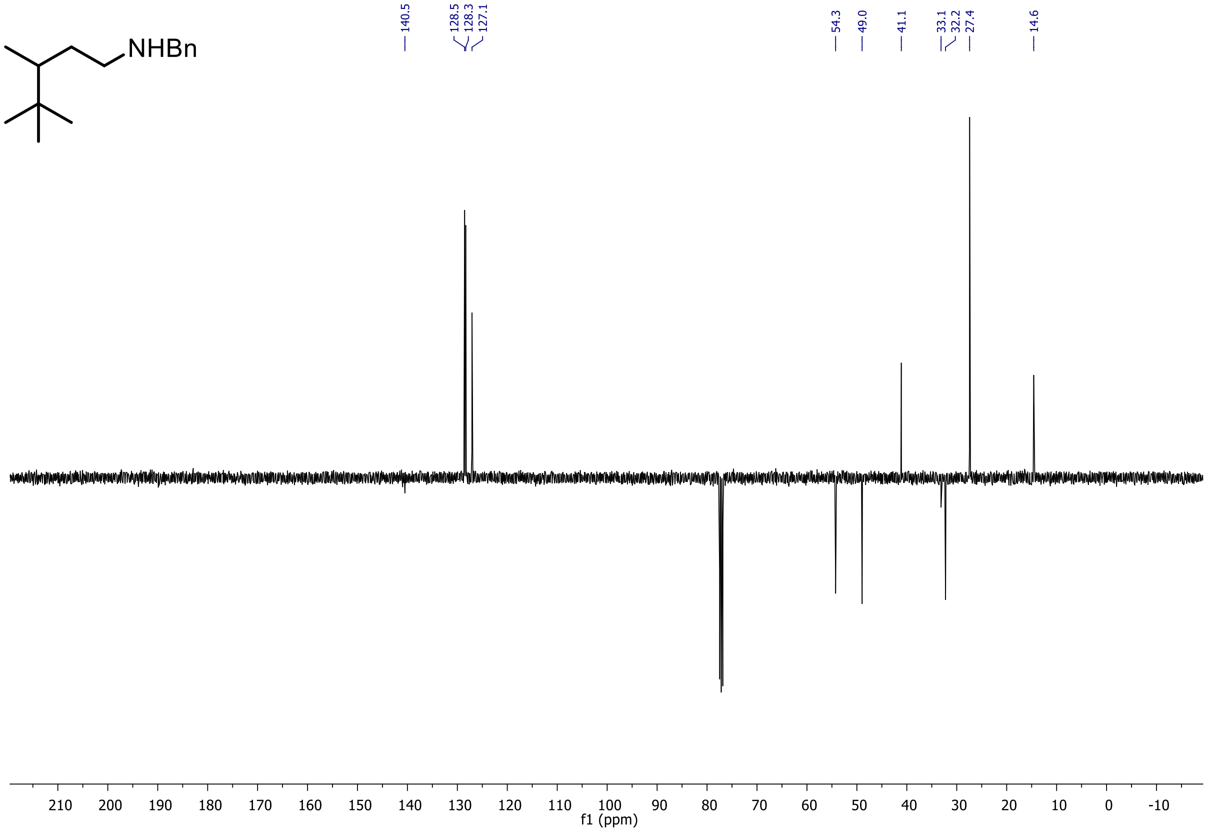
NHBn
140.5
128.5
128.3
127.1
54.3
49.0
41.1
33.1
32.2
27.4
14.6
210 200 190 180 170 160 150 140 130 120 110 100 90 80 70 60 50 40 30 20 10 0 -10
f1 (ppm)

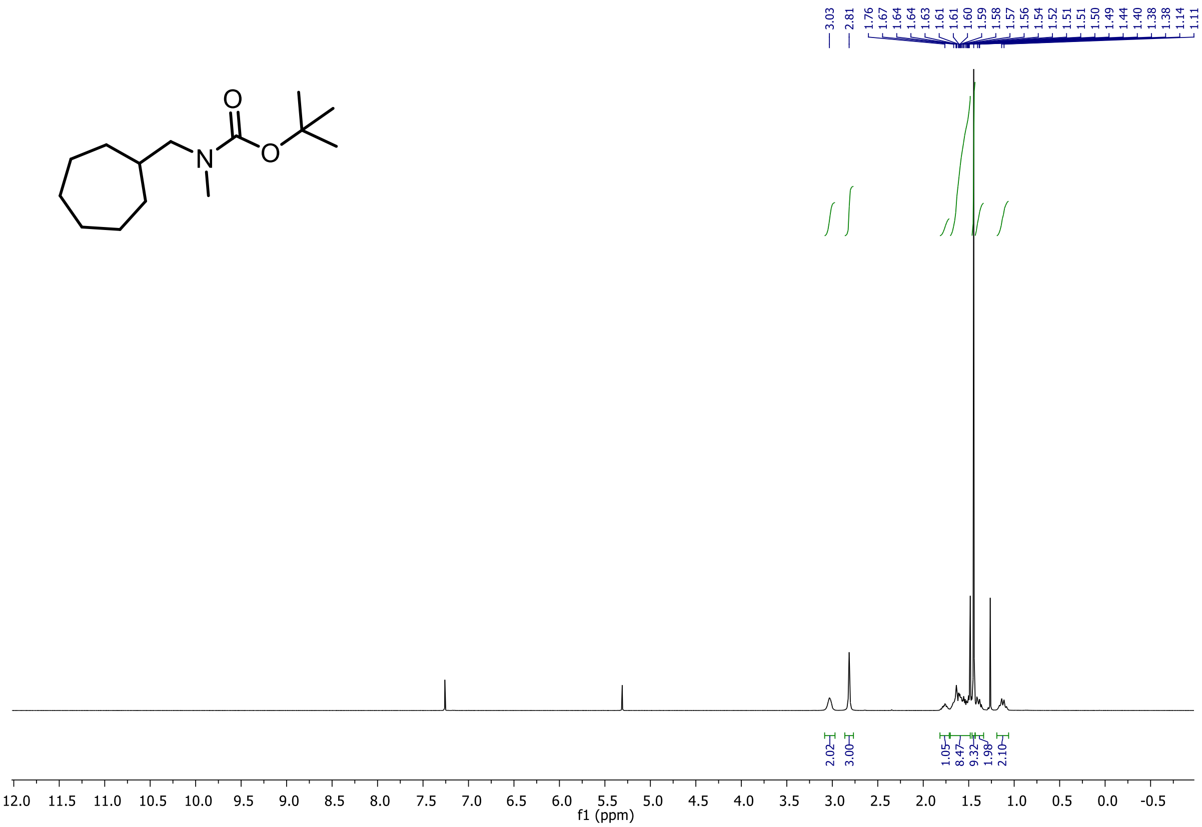
3.03
2.81
1.76 1.67 1.64 1.64 1.63 1.61 1.61 1.60 1.59 1.58 1.57 1.56 1.54 1.52 1.51 1.51 1.50 1.49 1.44 1.40 1.38 1.38 1.14 1.11
2.02 3.00 1.05 8.47 9.32 1.98 2.10
O
N
O
12.0 11.5 11.0 10.5 10.0 9.5 9.0 8.5 8.0 7.5 7.0 6.5 6.0 5.5 5.0 4.5 4.0 3.5 3.0 2.5 2.0 1.5 1.0 0.5 0.0 -0.5
f1 (ppm)

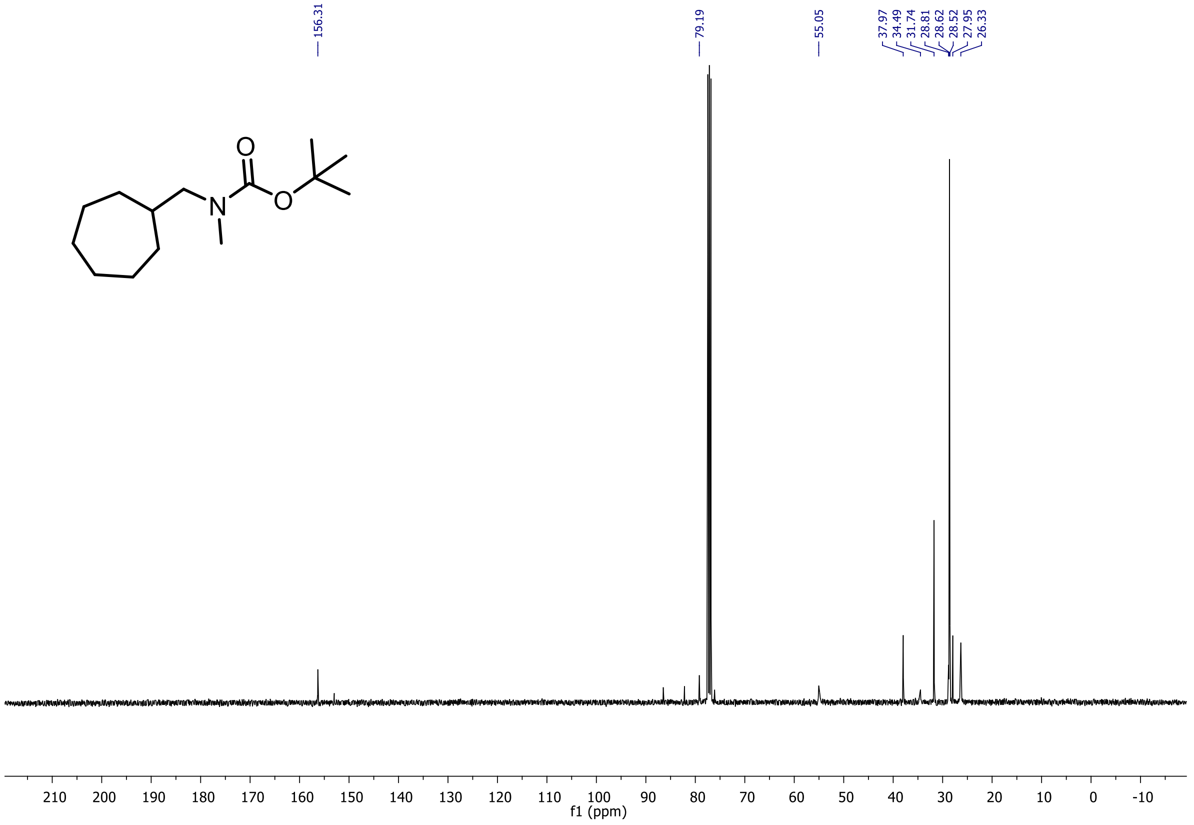
156.31
79.19
55.05
37.97 34.49 31.74 28.81 28.62 28.52 27.95 26.33
O
N
O
210 200 190 180 170 160 150 140 130 120 110 100 90 80 70 60 50 40 30 20 10 0 -10
f1 (ppm)

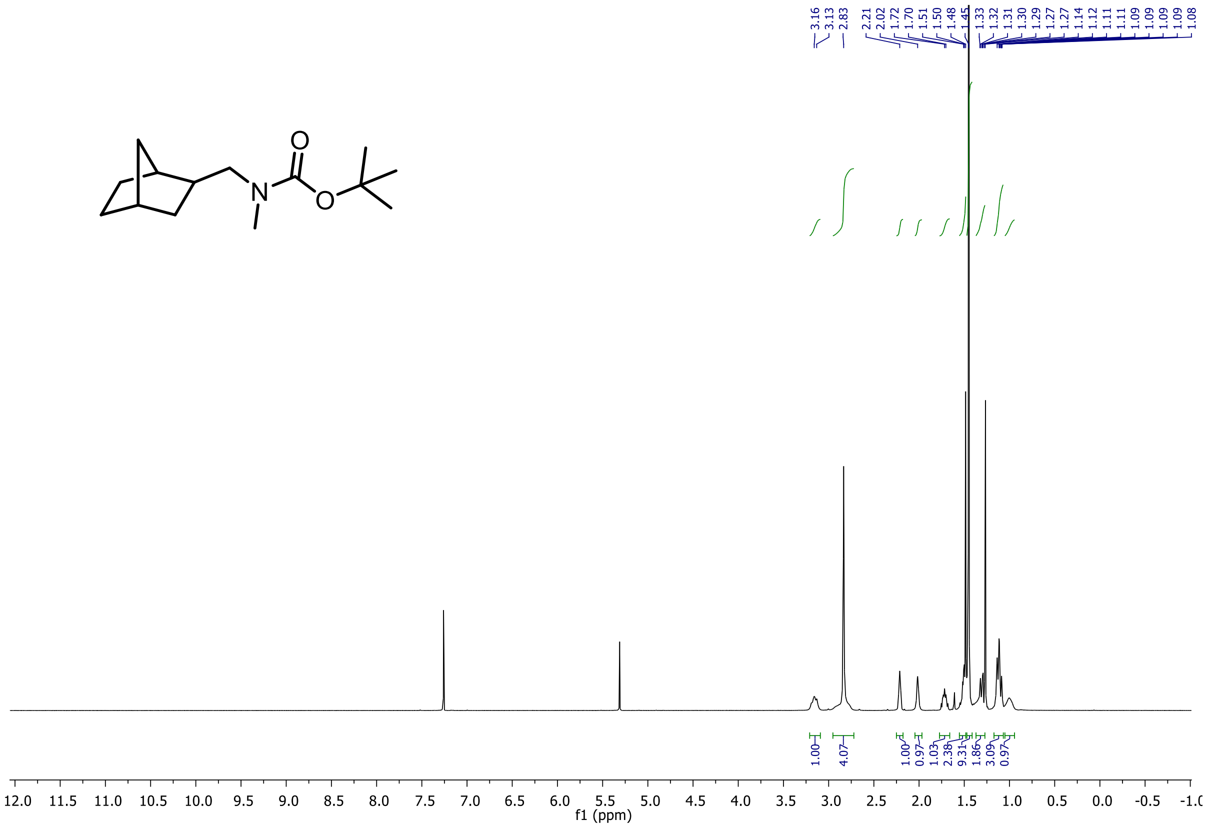
3.16
3.13
2.83
2.21
2.02
1.72
1.70
1.51
1.50
1.48
1.45
1.33
1.32
1.31
1.30
1.29
1.27
1.27
1.14
1.12
1.11
1.11
1.09
1.09
1.09
1.09
1.08
O
N
O
1.00
4.07
1.00
0.97
1.03
2.38
9.31
1.86
3.09
0.97
12.0 11.5 11.0 10.5 10.0 9.5 9.0 8.5 8.0 7.5 7.0 6.5 6.0 5.5 5.0 4.5 4.0 3.5 3.0 2.5 2.0 1.5 1.0 0.5 0.0 -0.5
f1 (ppm)

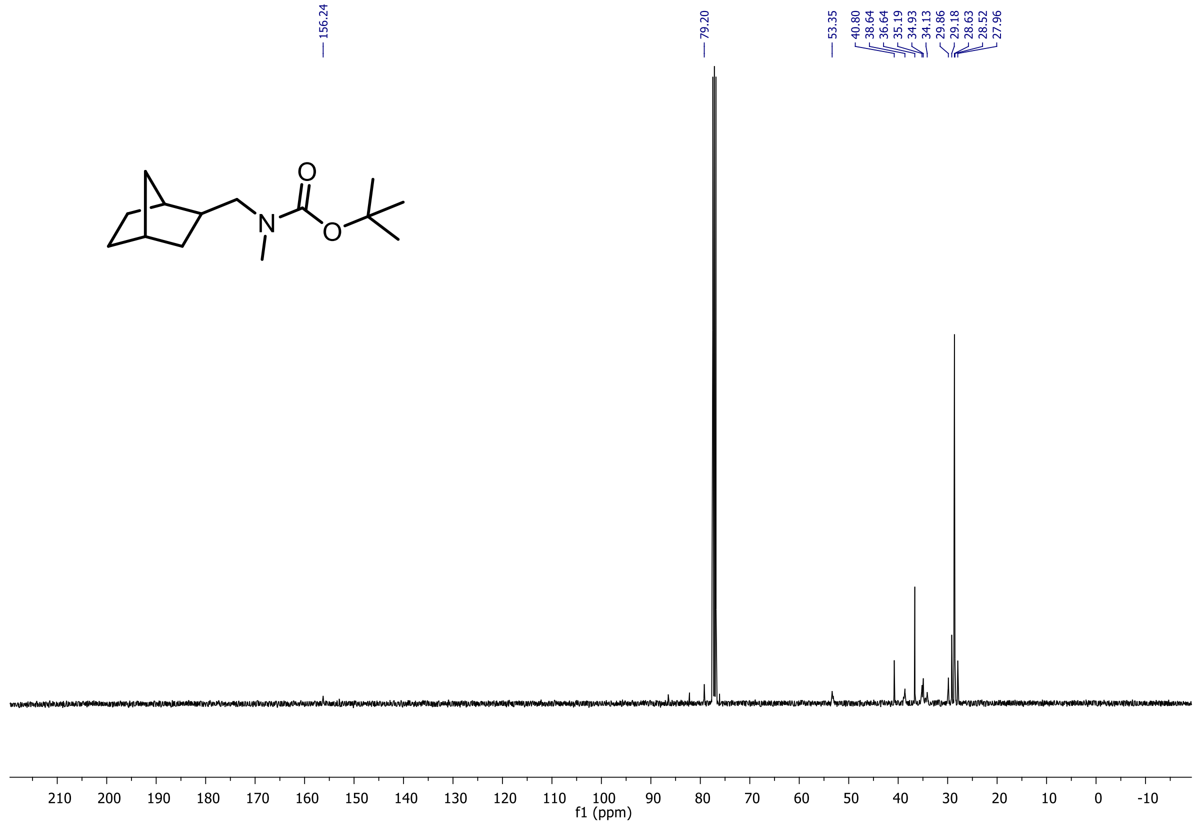
156.24
79.20
53.35
40.80
38.64
36.64
35.19
34.93
34.13
29.86
29.18
28.63
28.52
27.96
O
N
O
210 200 190 180 170 160 150 140 130 120 110 100 90 80 70 60 50 40 30 20 10 0 -10
f1 (ppm)

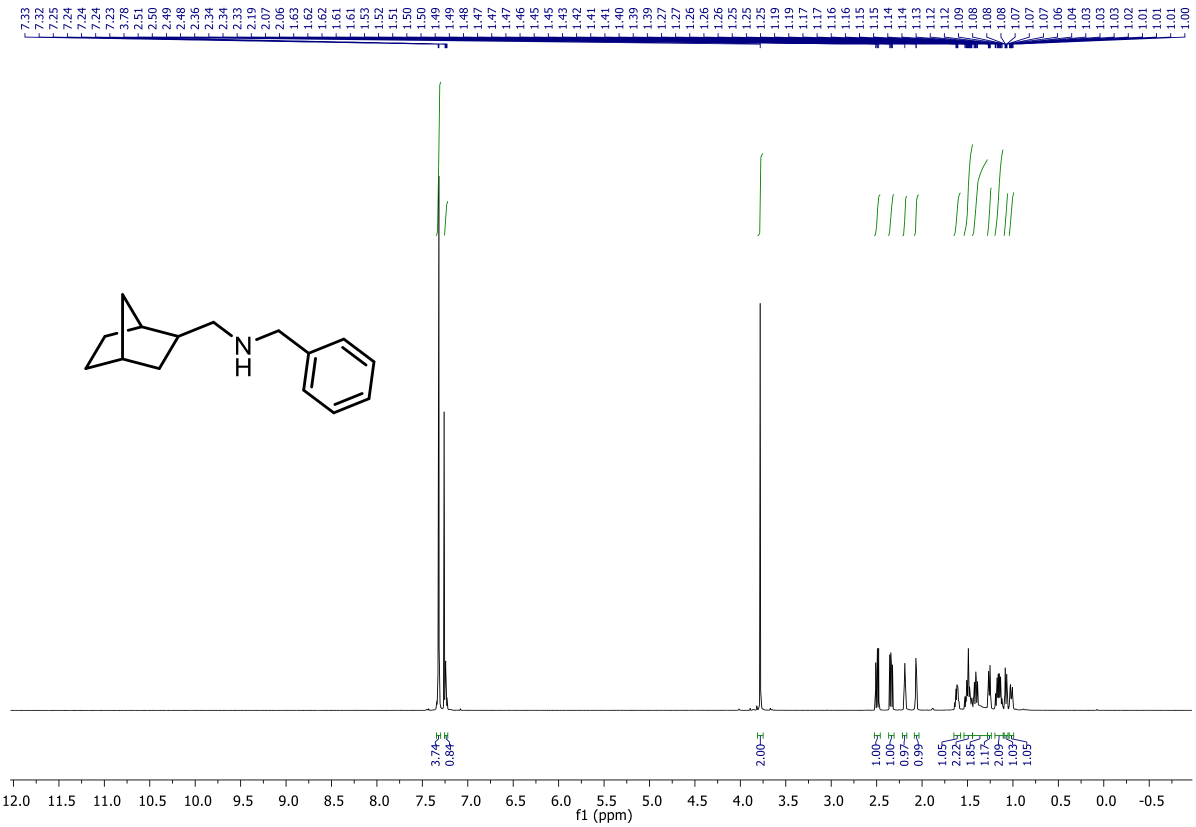
7.33 7.32 7.25 7.24 7.24 7.24 7.23 3.78 2.51 2.50 2.49 2.48 2.36 2.34 2.34 2.33 2.19 2.07 2.06 1.63 1.62 1.62 1.61 1.61 1.53 1.52 1.51 1.50 1.50 1.49 1.49 1.48 1.47 1.47 1.46 1.45 1.43 1.42 1.41 1.41 1.40 1.39 1.39 1.27 1.27 1.26 1.26 1.26 1.25 1.25 1.25 1.19 1.19 1.17 1.17 1.16 1.15 1.15 1.14 1.14 1.13 1.13 1.12 1.12 1.09 1.08 1.08 1.08 1.07 1.07 1.07 1.06 1.04 1.03 1.03 1.02 1.01 1.01 1.00
N
H
3.74
0.84
2.00
1.00
1.00
0.97
0.99
1.05
2.22
1.85
1.17
2.09
1.03
1.05
12.0 11.5 11.0 10.5 10.0 9.5 9.0 8.5 8.0 7.5 7.0 6.5 6.0 5.5 5.0 4.5 4.0 3.5 3.0 2.5 2.0 1.5 1.0 0.5 0.0 -0.5
f1 (ppm)

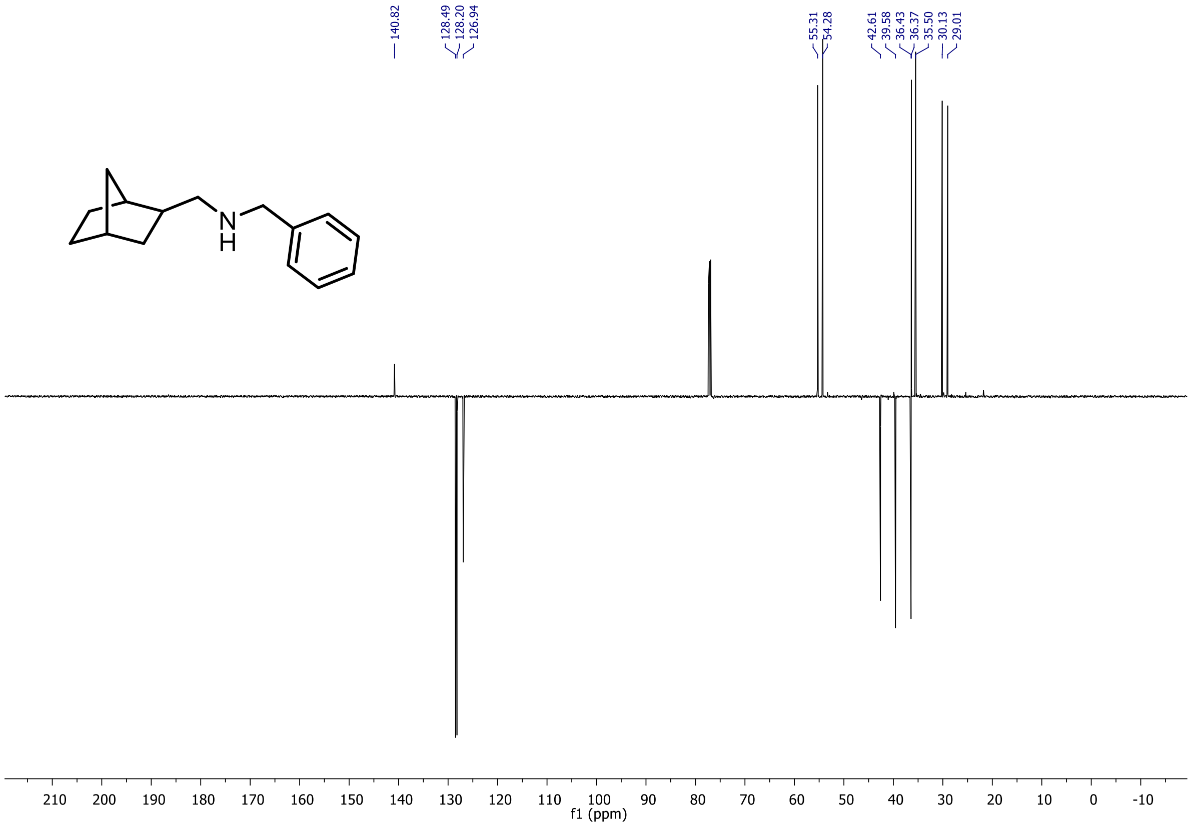
140.82
128.49 128.20 126.94
55.31 54.28
42.61 39.58 36.43 36.37 35.50 30.13 29.01
N
H
210 200 190 180 170 160 150 140 130 120 110 100 90 80 70 60 50 40 30 20 10 0 -10
f1 (ppm)

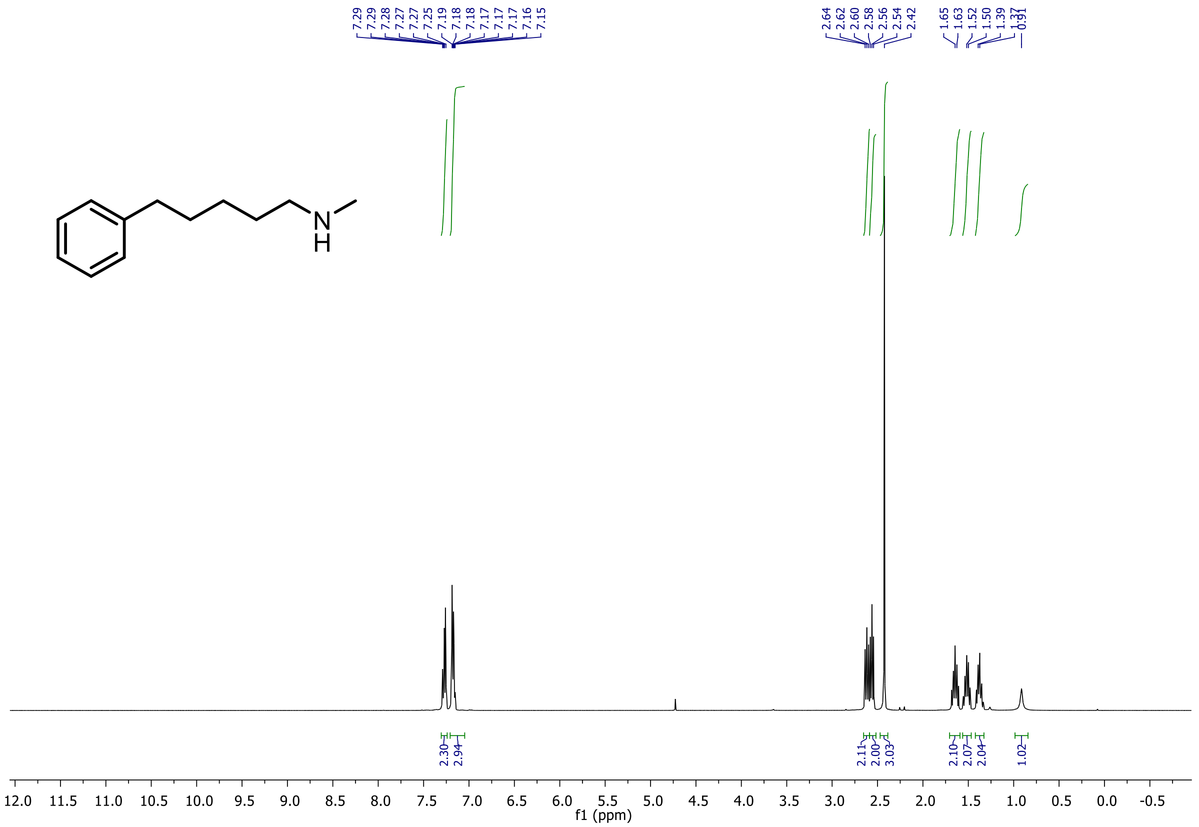

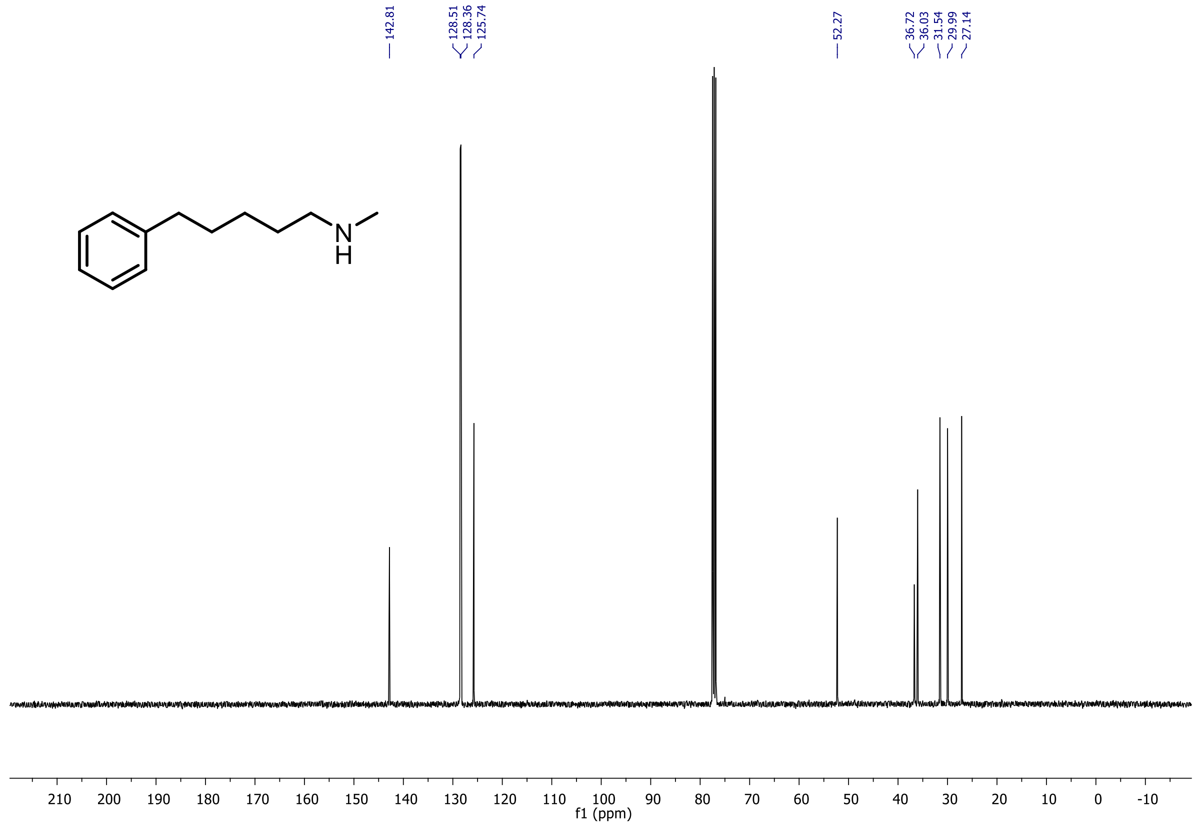

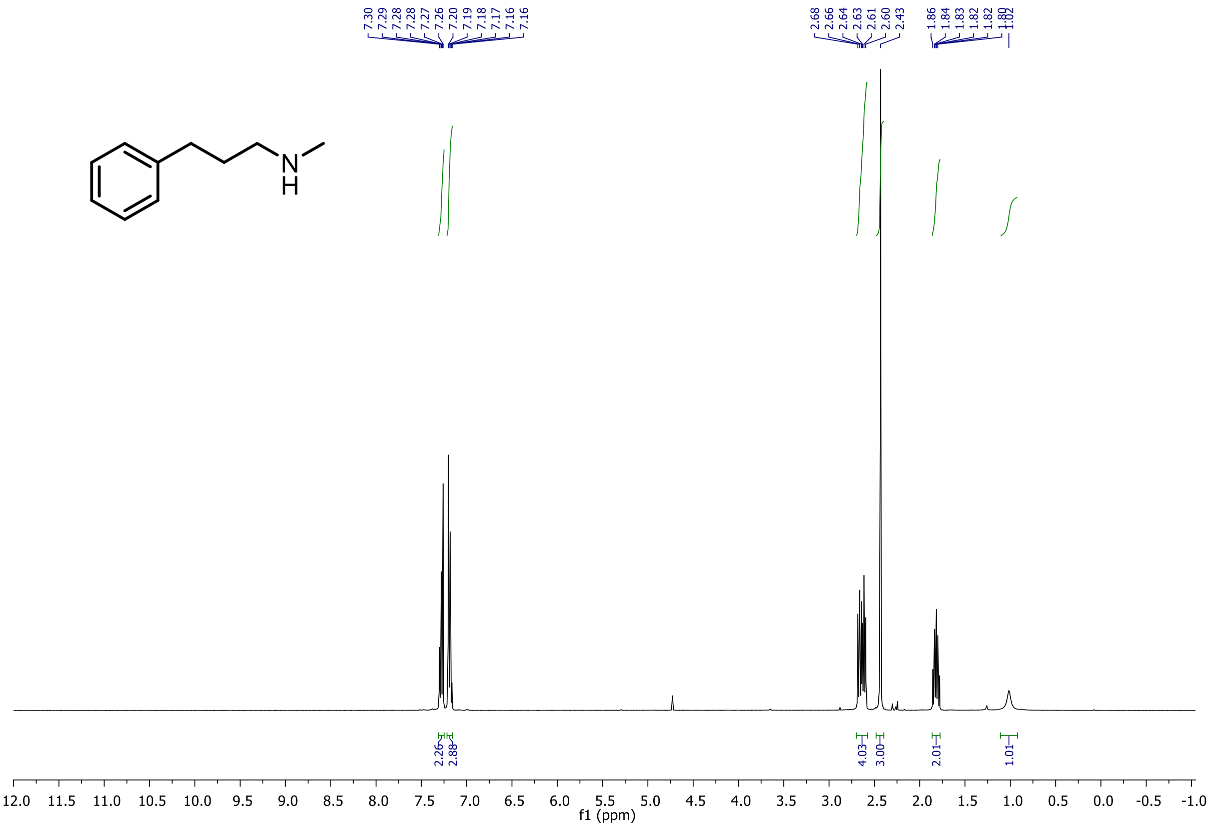
7.30
7.29
7.28
7.28
7.27
7.26
7.20
7.19
7.18
7.17
7.16
7.16
2.68
2.66
2.64
2.63
2.61
2.60
2.43
1.86
1.84
1.83
1.82
1.82
1.80
1.02
N
H
2.26
2.88
4.03
3.00
2.01
1.01
12.0 11.5 11.0 10.5 10.0 9.5 9.0 8.5 8.0 7.5 7.0 6.5 6.0 5.5 5.0 4.5 4.0 3.5 3.0 2.5 2.0 1.5 1.0 0.5 0.0 -0.5 -1.0
f1 (ppm)

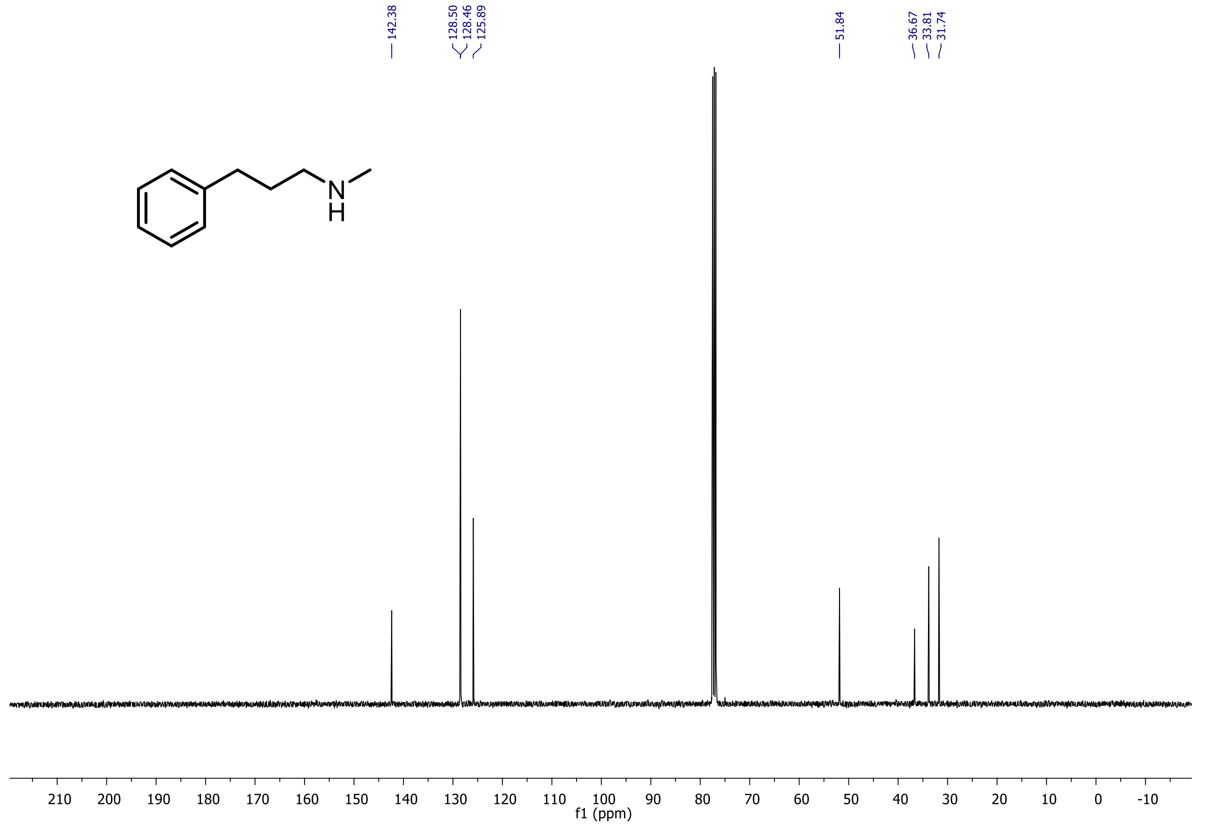
142.38
128.50
128.46
125.89
51.84
36.67
33.81
31.74
N
H
210 200 190 180 170 160 150 140 130 120 110 100 90 80 70 60 50 40 30 20 10 0 -10
f1 (ppm)

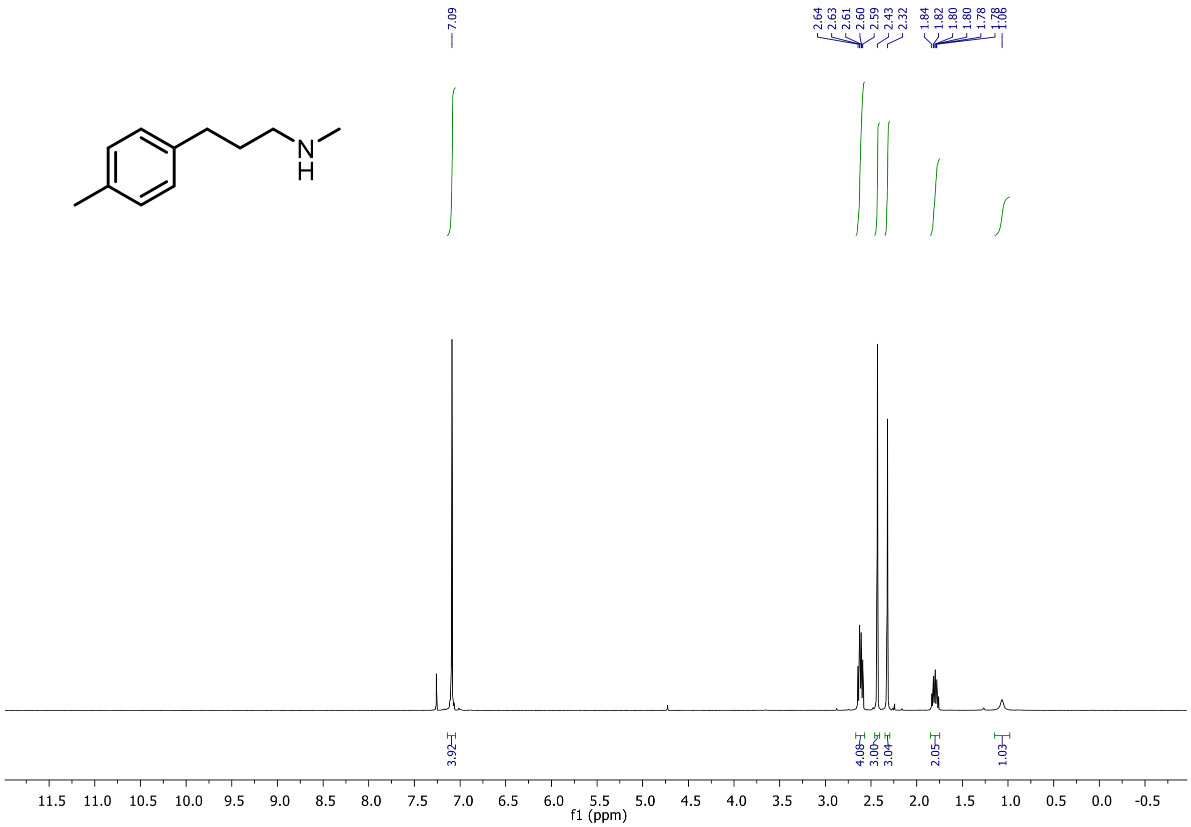
7.09
2.64
2.63
2.61
2.60
2.59
2.43
2.32
1.84
1.82
1.80
1.80
1.78
1.08
N
H
3.92
4.08
3.00
3.04
2.05
1.03
11.5 11.0 10.5 10.0 9.5 9.0 8.5 8.0 7.5 7.0 6.5 6.0 5.5 5.0 4.5 4.0 3.5 3.0 2.5 2.0 1.5 1.0 0.5 0.0 -0.5
f1 (ppm)

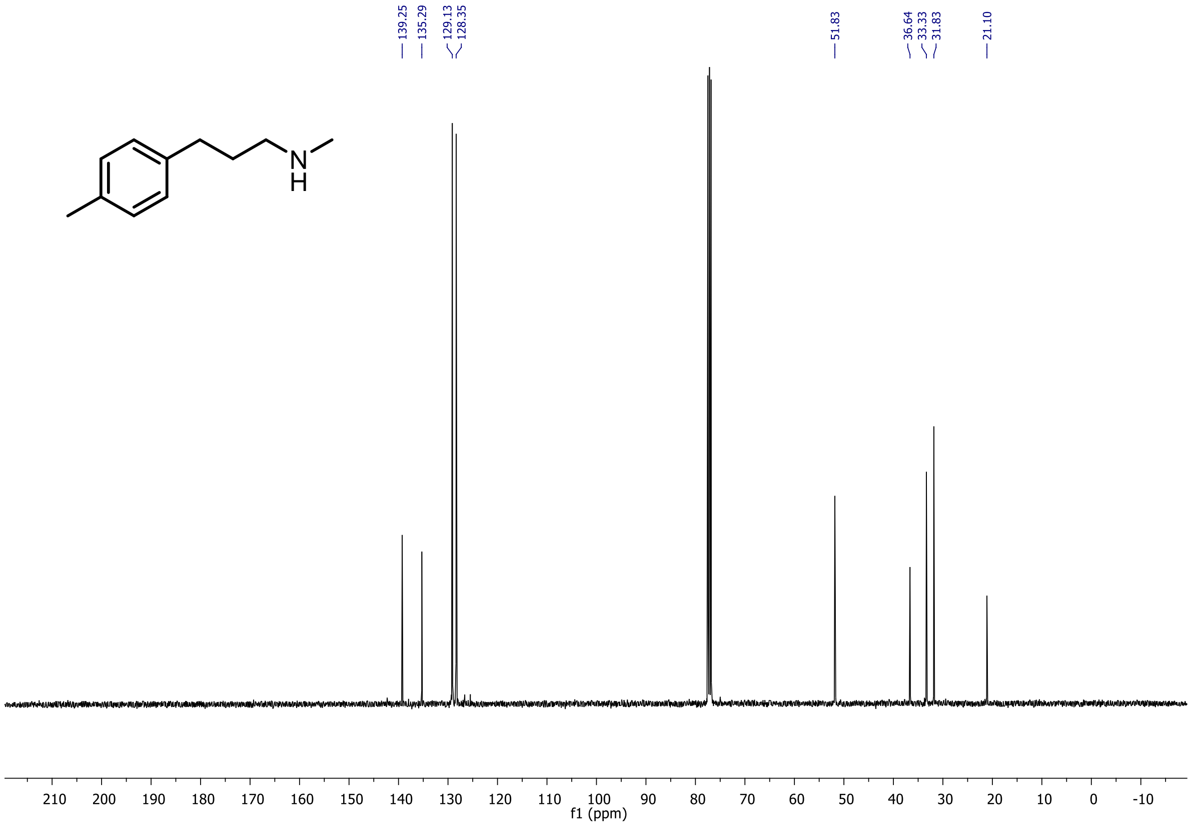
139.25
135.29
129.13
128.35
51.83
36.64
33.33
31.83
21.10
N
H
210 200 190 180 170 160 150 140 130 120 110 100 90 80 70 60 50 40 30 20 10 0 -10
f1 (ppm)

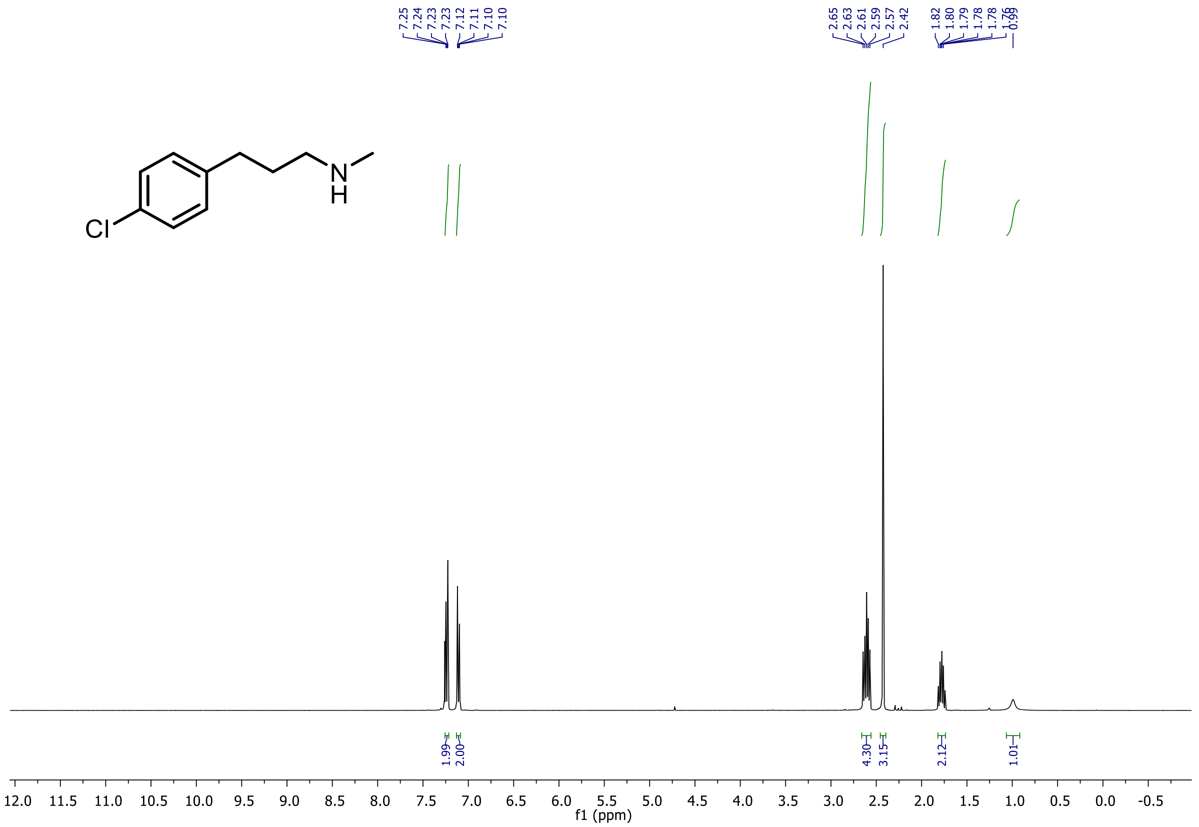

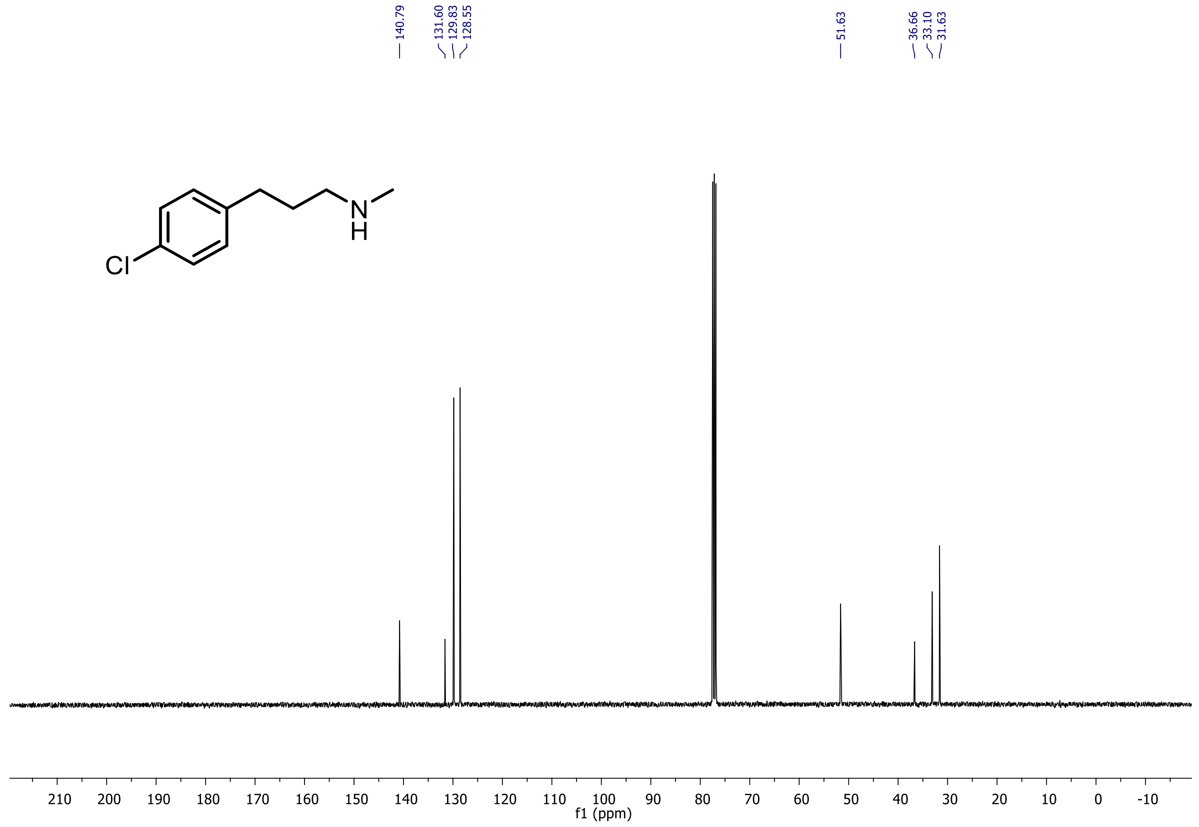

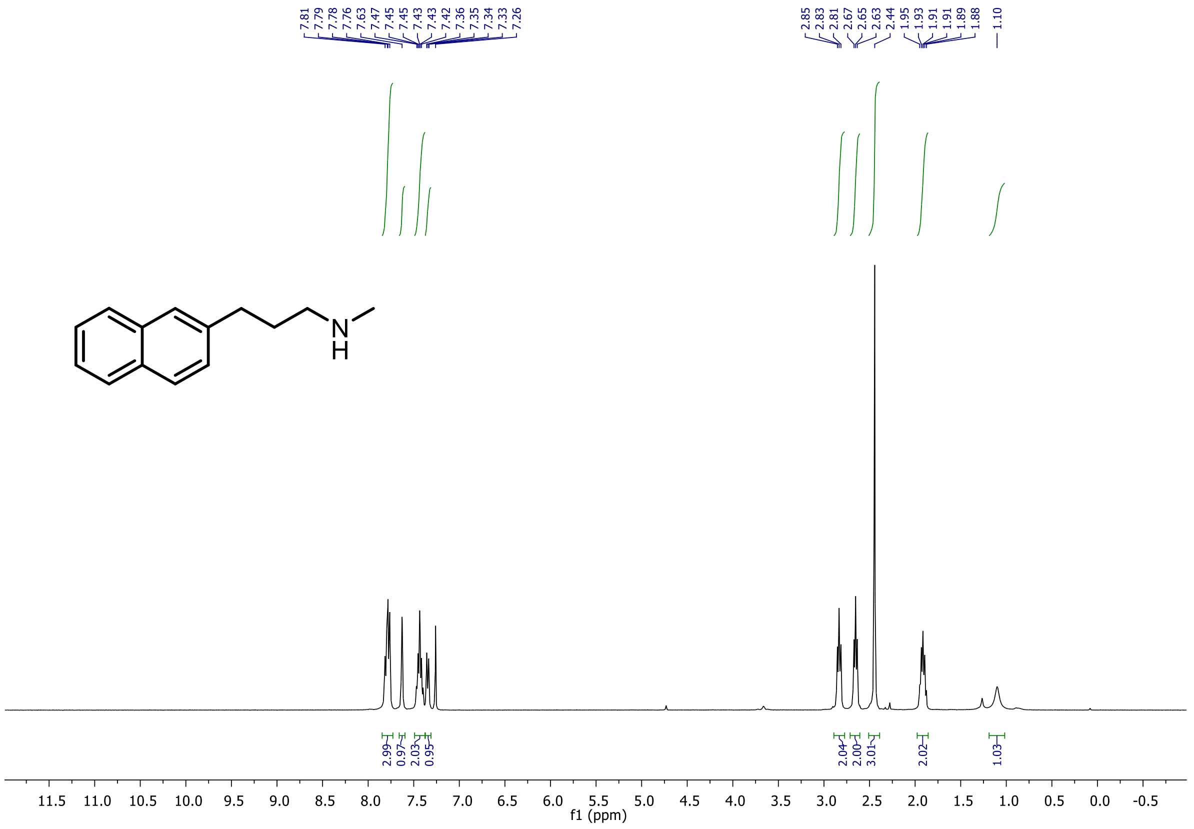

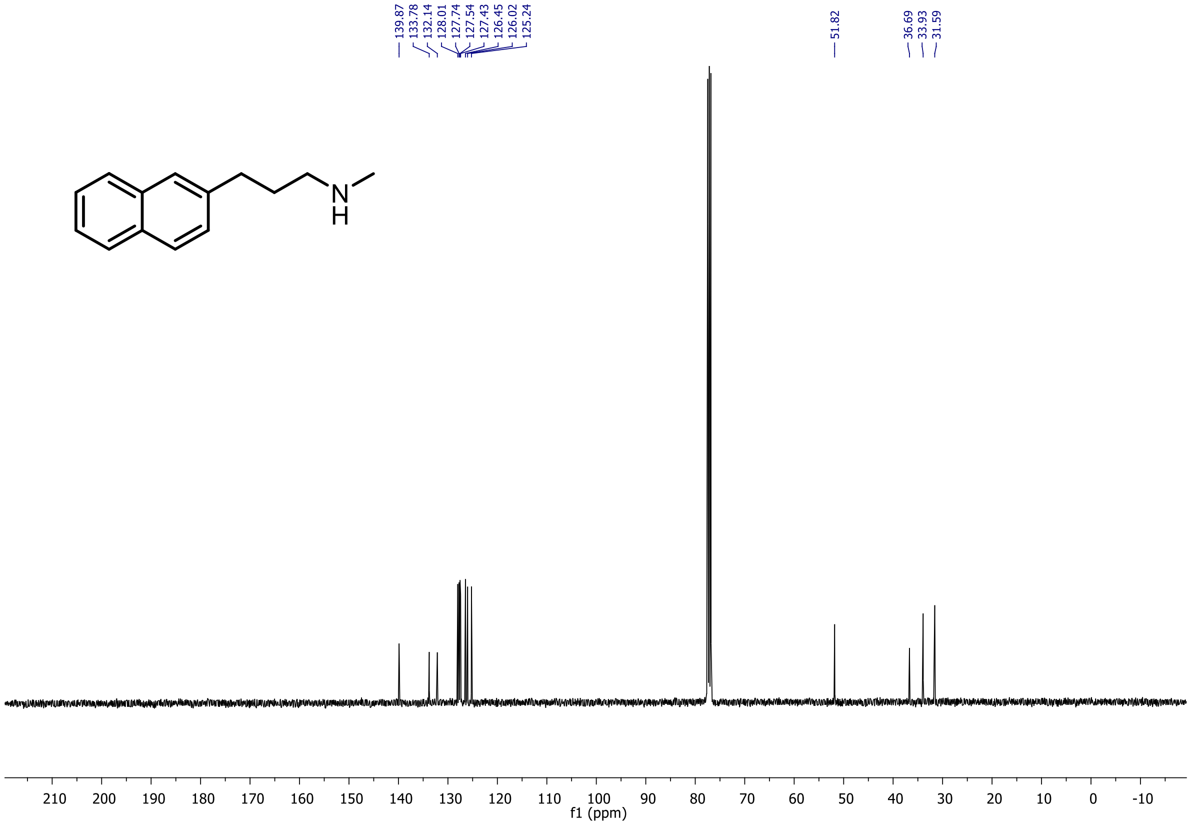

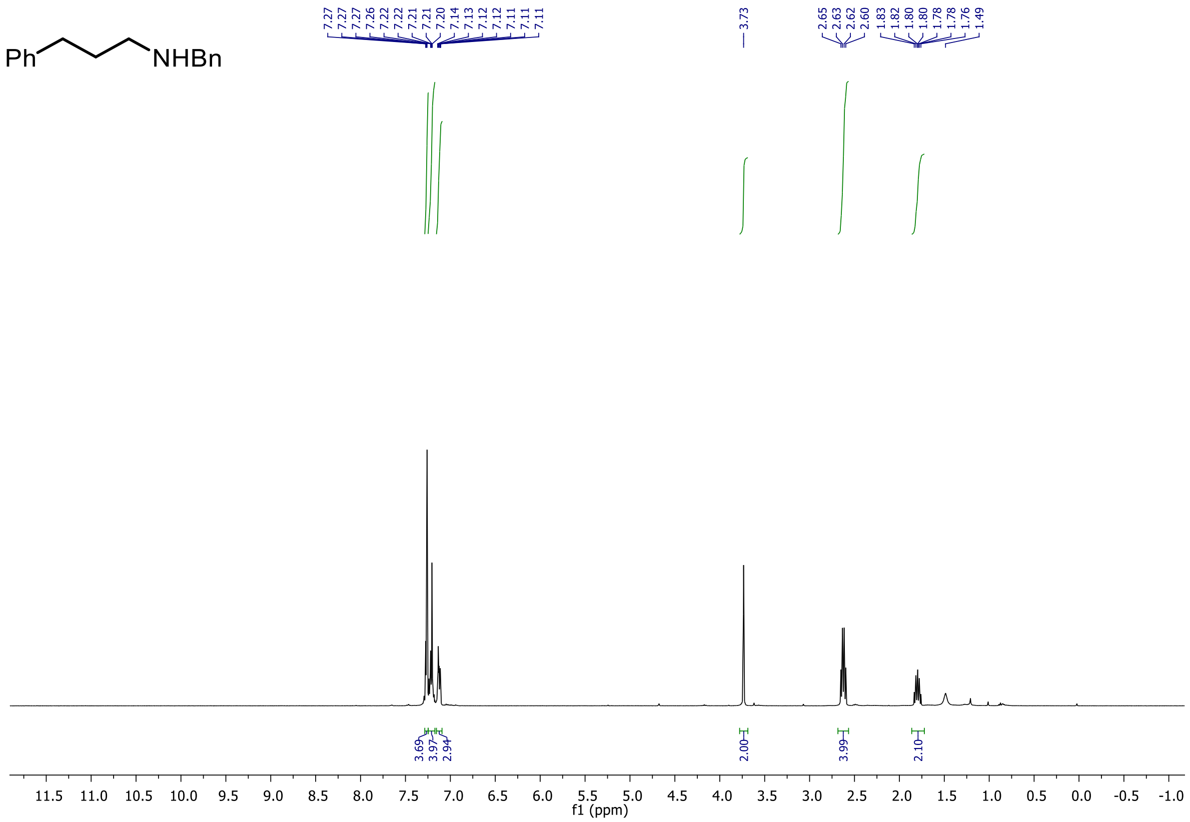
Ph NHBn
7.27 7.27 7.27 7.26 7.22 7.22 7.21 7.21 7.20 7.14 7.13 7.12 7.12 7.11 7.11 7.11
3.73
2.65 2.63 2.62 2.60 1.83 1.82 1.80 1.80 1.78 1.78 1.76 1.49
3.69 3.97 2.94
2.00
3.99
2.10
11.5 11.0 10.5 10.0 9.5 9.0 8.5 8.0 7.5 7.0 6.5 6.0 5.5 5.0 4.5 4.0 3.5 3.0 2.5 2.0 1.5 1.0 0.5 0.0 -0.5 -1.0
f1 (ppm)

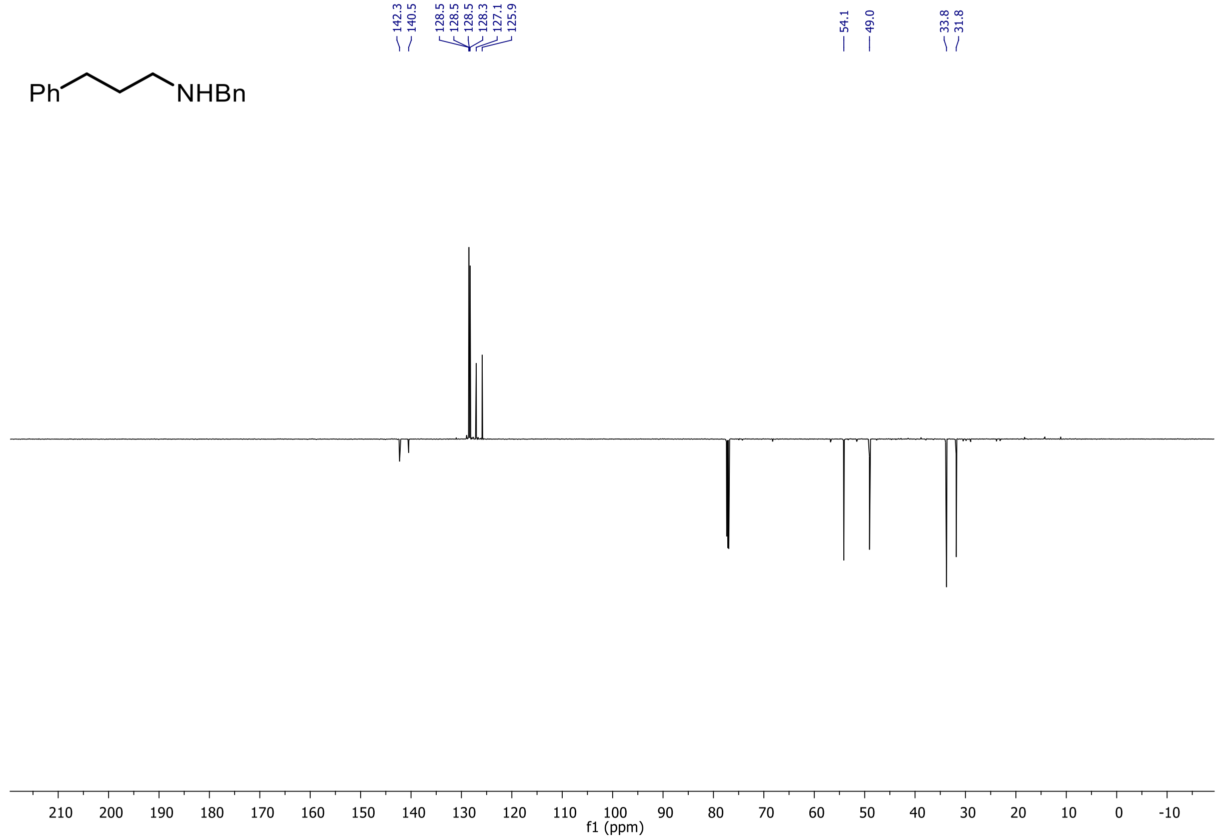
142.3 140.5 128.5 128.5 128.5 128.3 127.1 125.9
54.1 49.0
33.8 31.8
Ph NHBn
210 200 190 180 170 160 150 140 130 120 110 100 90 80 70 60 50 40 30 20 10 0 -10
f1 (ppm)

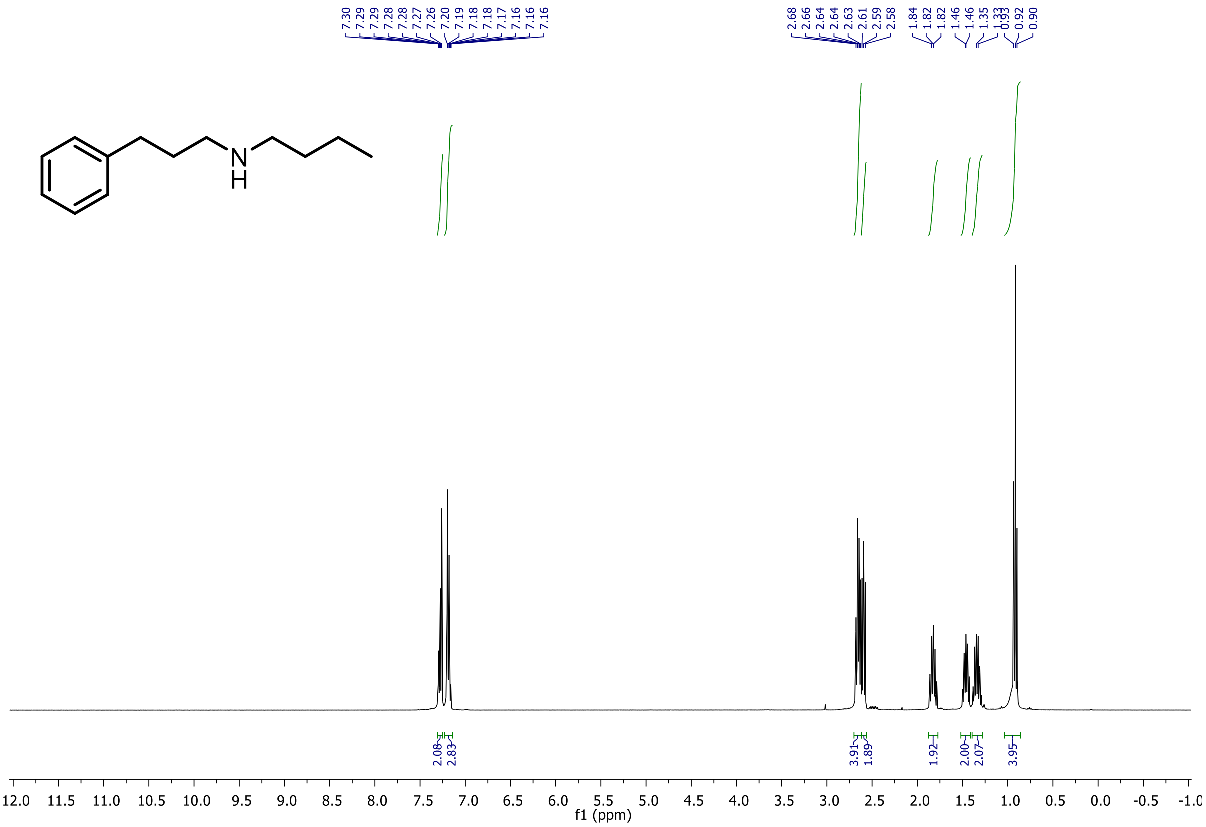
7.30 7.29 7.29 7.28 7.28 7.27 7.26 7.20 7.19 7.18 7.18 7.17 7.16 7.16
2.68 2.66 2.64 2.64 2.63 2.61 2.59 2.58 1.84 1.82 1.82 1.46 1.46 1.35 1.33 0.93 0.92 0.90
N
H
2.08 2.83 3.91 1.89 1.92 2.00 2.07 3.95
f1 (ppm)

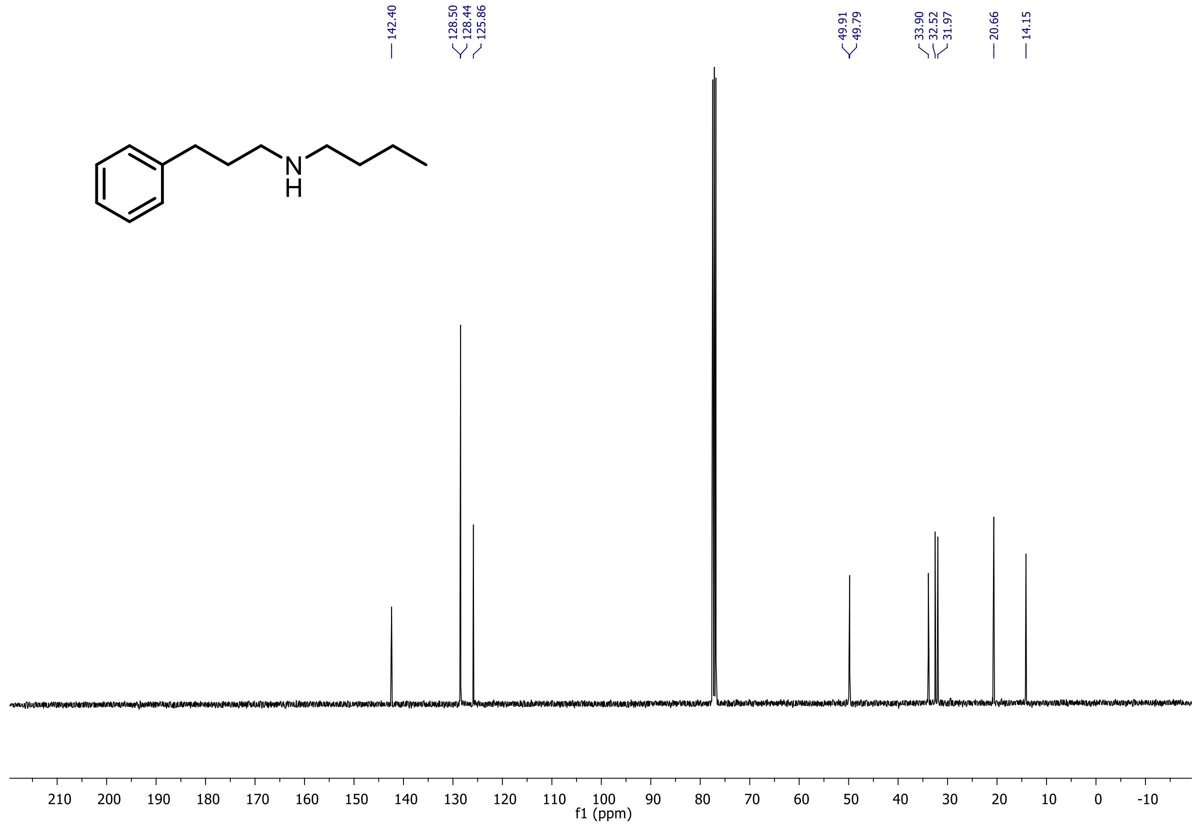
142.40 128.50 128.44 125.86 49.91 49.79 33.90 32.52 31.97 20.66 14.15
N
H
f1 (ppm)

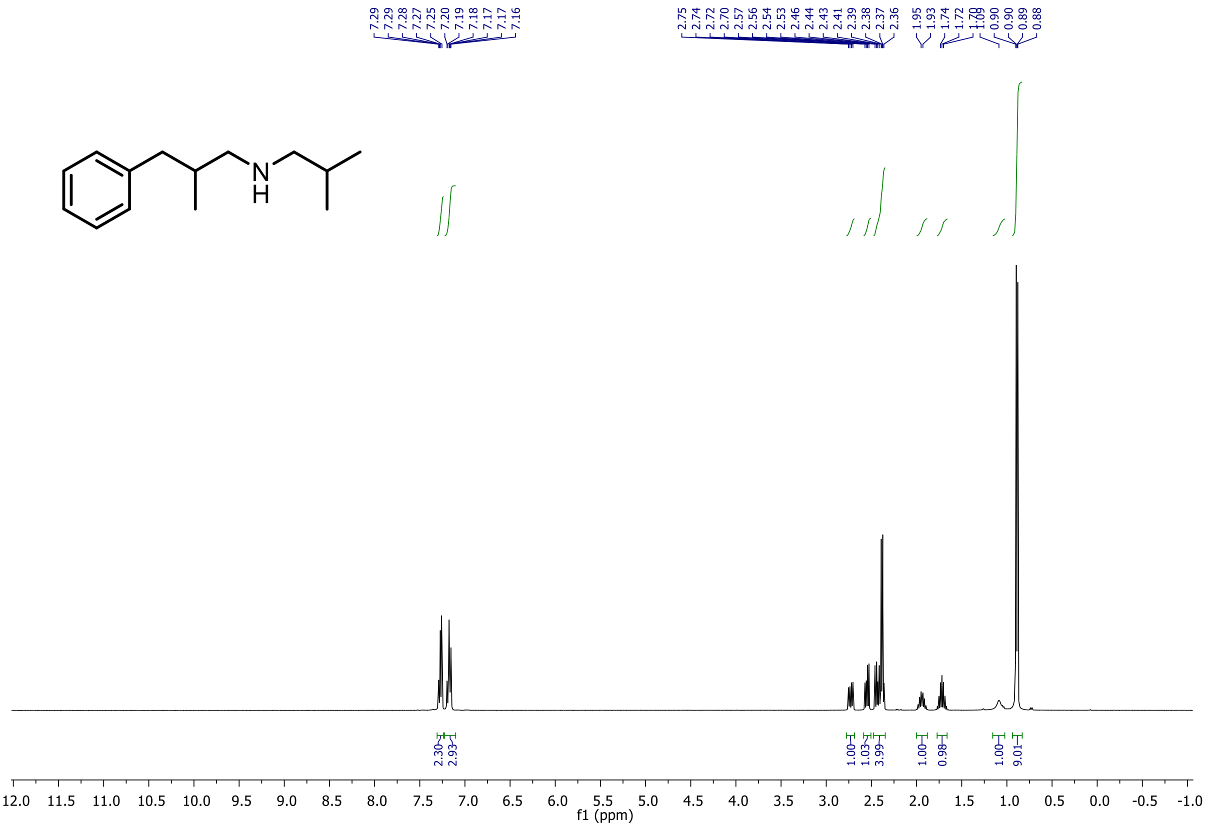

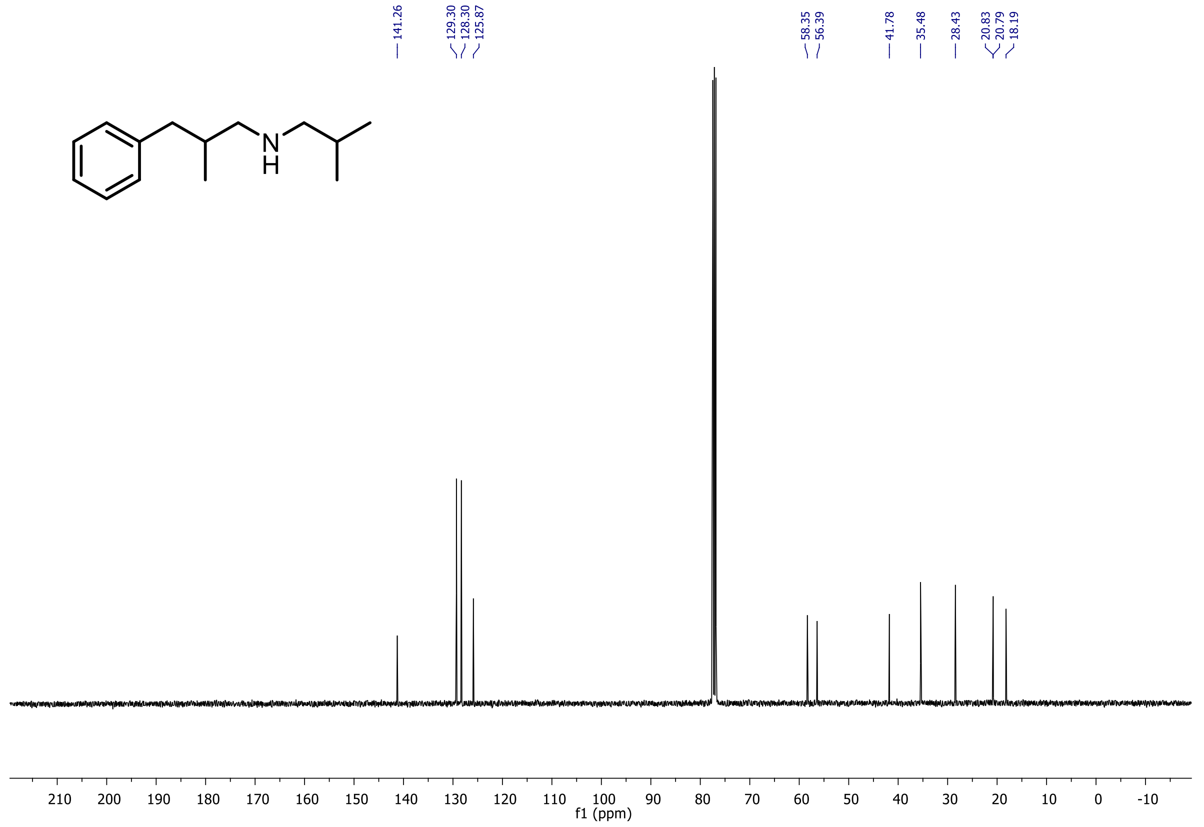

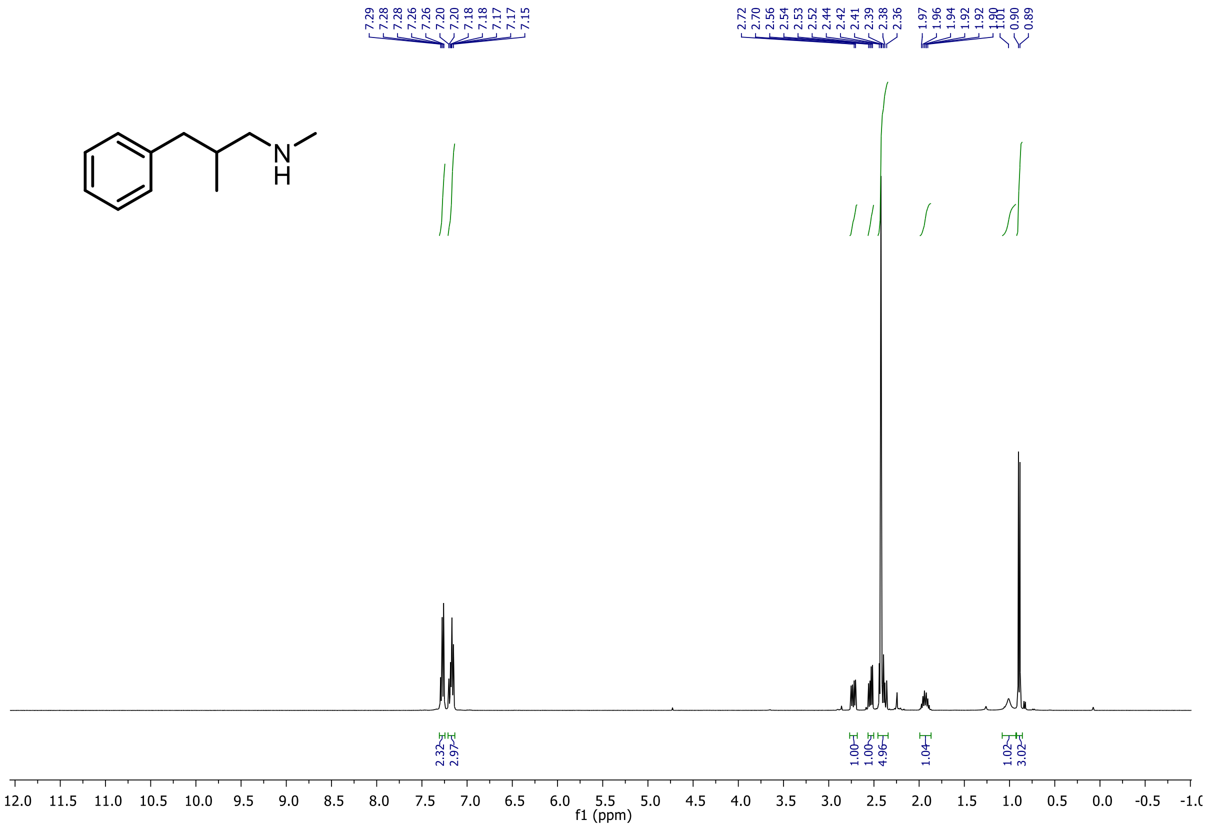

7.29 7.28 7.28 7.26 7.26 7.20 7.20 7.18 7.17 7.17 7.15
2.72 2.70 2.56 2.54 2.53 2.52 2.44 2.42 2.41 2.39 2.38 2.36
1.97 1.96 1.94 1.92 1.92 1.90 1.01 0.90 0.89
2.32 2.97
1.00 1.00 4.96
1.04
1.02 3.02
f1 (ppm)

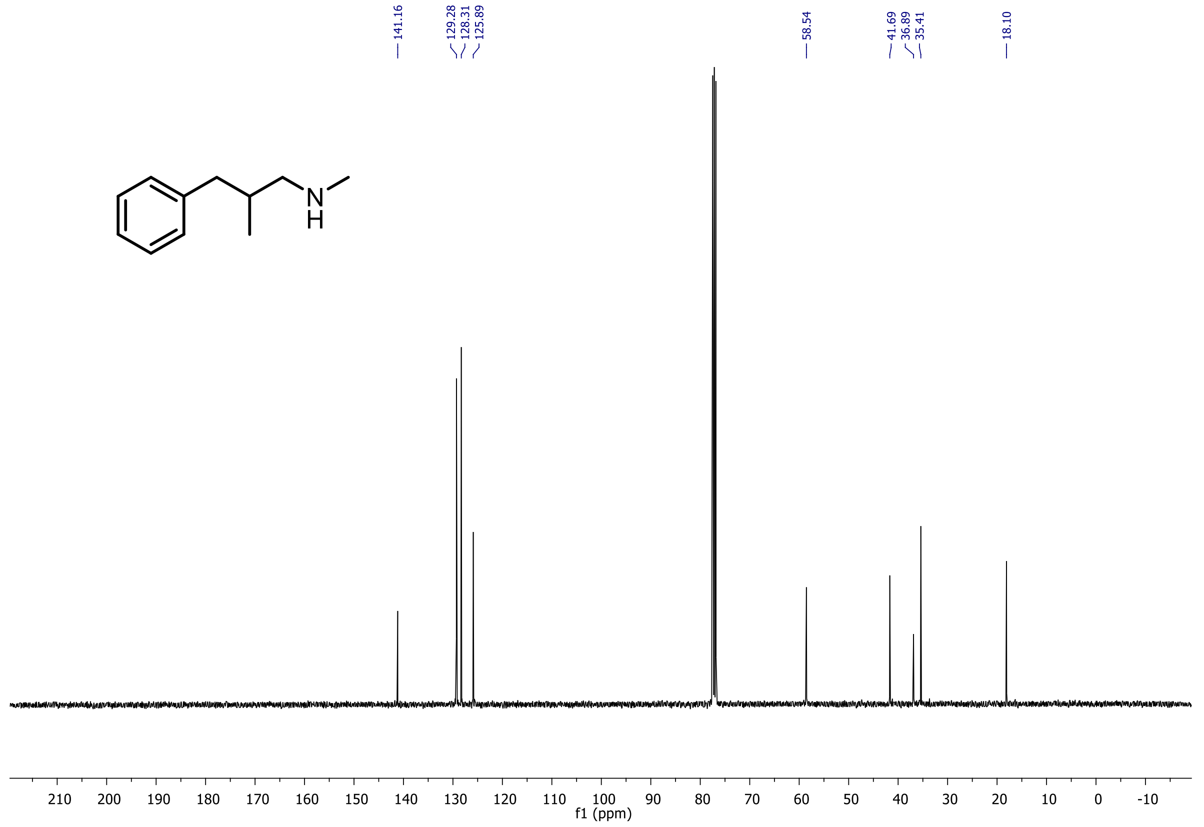

141.16
129.28 128.31 125.89
58.54
41.69 36.89 35.41
18.10
f1 (ppm)

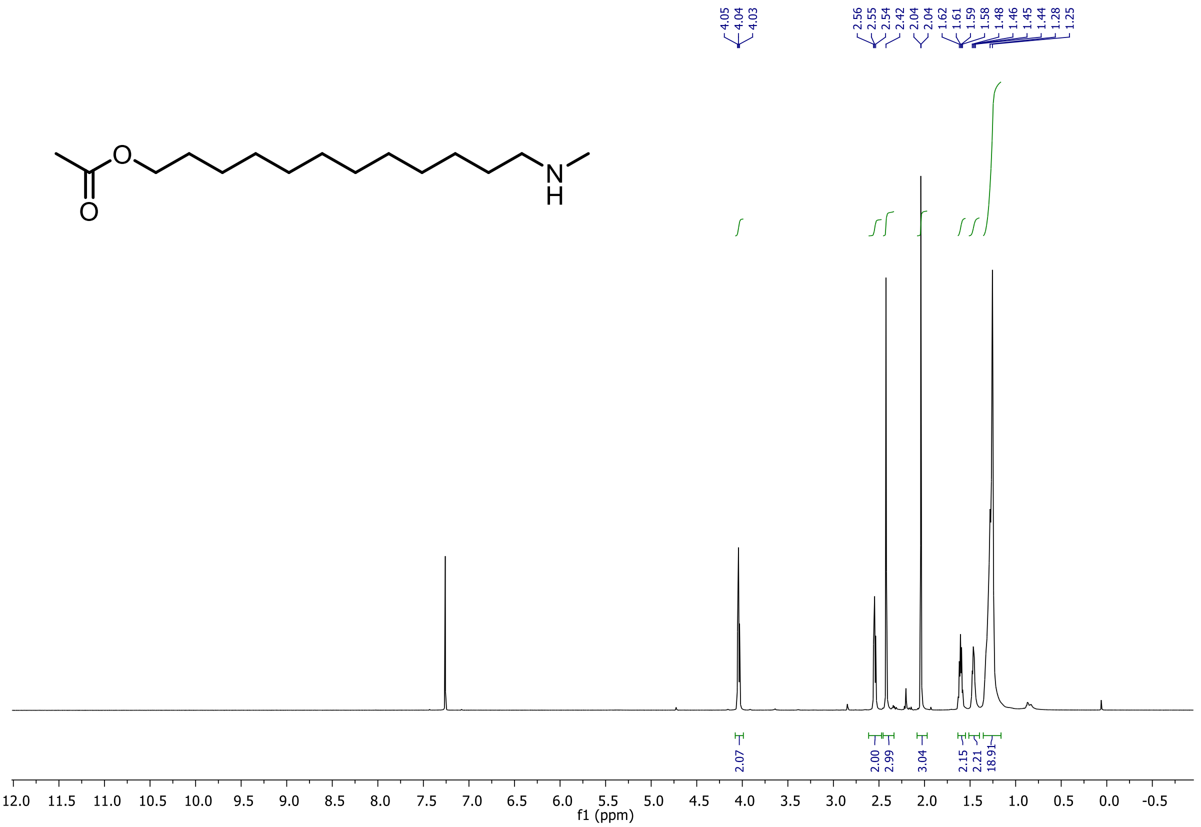
4.05
4.04
4.03
2.56
2.55
2.54
2.42
2.04
2.04
1.62
1.61
1.59
1.58
1.48
1.46
1.45
1.44
1.28
1.25
O
N
H
2.07
2.00
2.99
3.04
2.15
2.21
18.91
f1 (ppm)

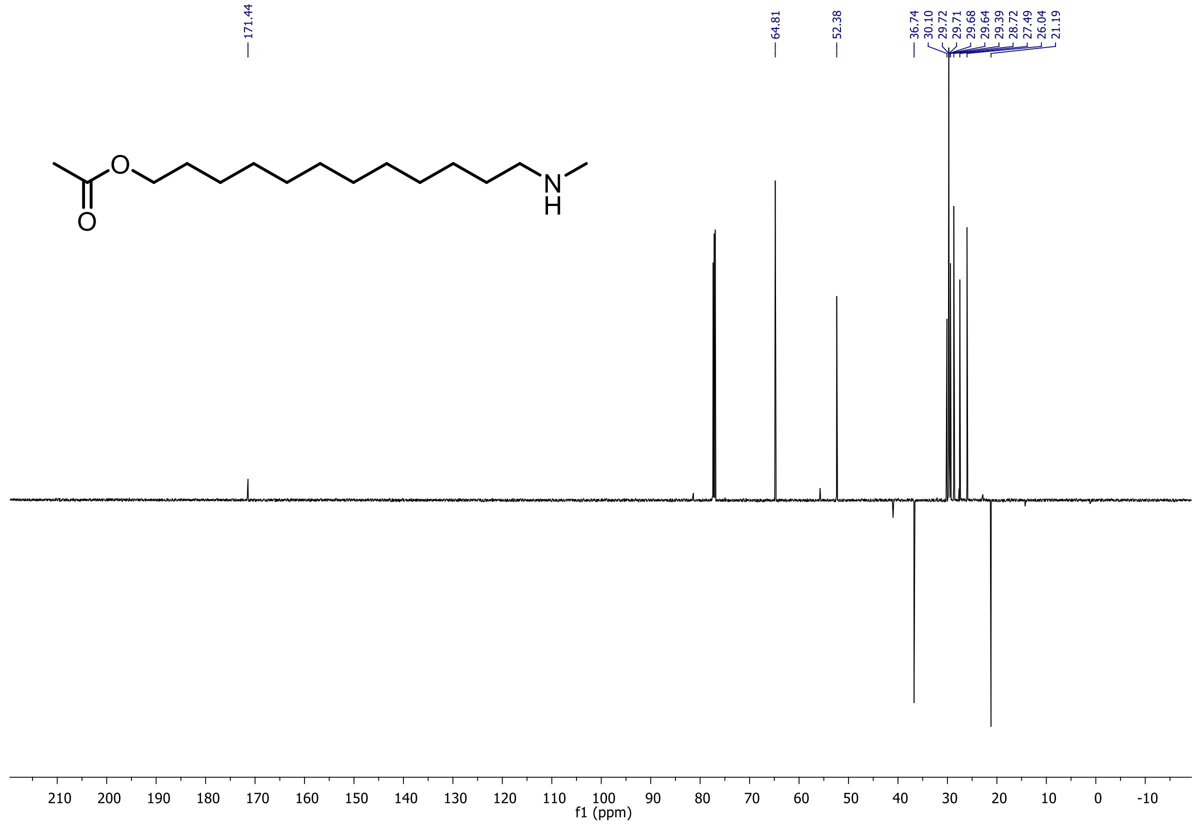
171.44
64.81
52.38
36.74
30.10
29.72
29.71
29.68
29.64
29.39
28.72
27.49
26.04
21.19
O
N
H
f1 (ppm)

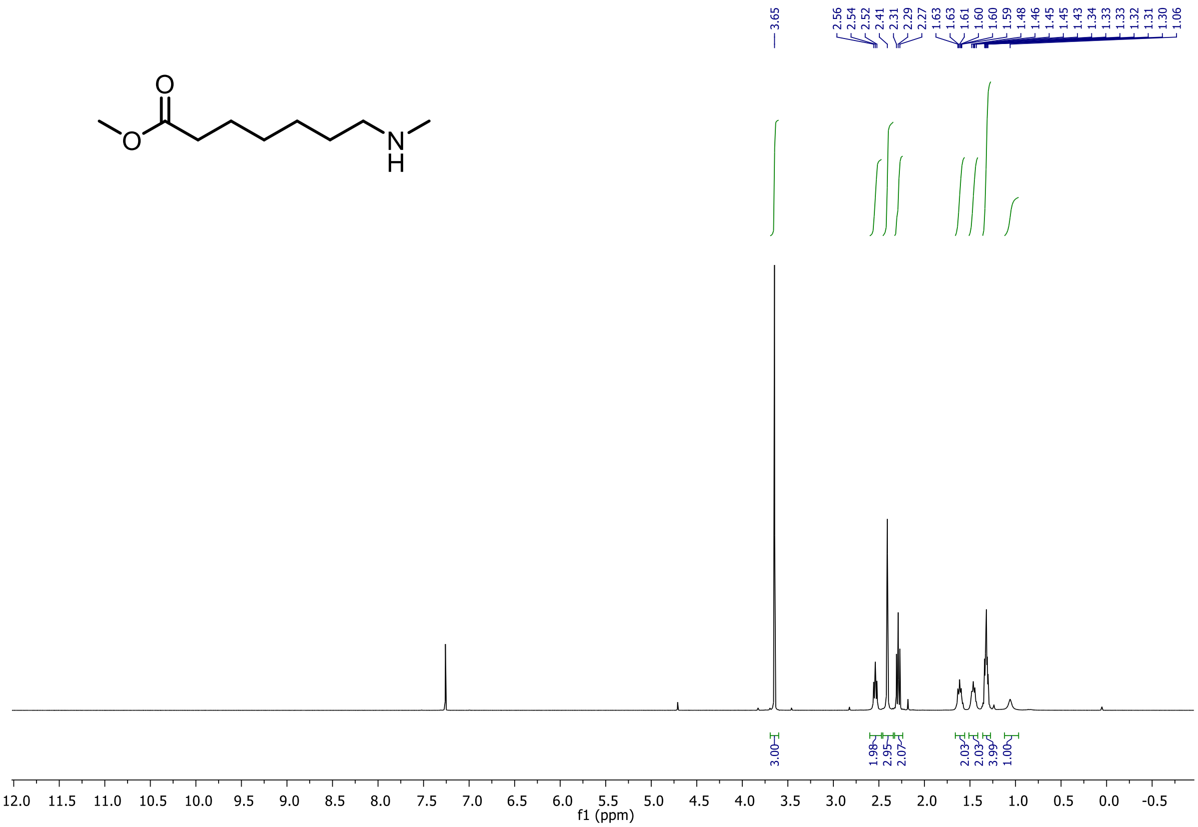

3.65
2.56 2.54 2.52 2.41 2.31 2.29 2.27 1.63 1.63 1.61 1.60 1.60 1.59 1.48 1.46 1.45 1.45 1.43 1.34 1.33 1.33 1.32 1.31 1.30 1.06
O
O
N
H
3.00
1.98 2.95 2.07
2.03 2.03 3.99 1.00
12.0 11.5 11.0 10.5 10.0 9.5 9.0 8.5 8.0 7.5 7.0 6.5 6.0 5.5 5.0 4.5 4.0 3.5 3.0 2.5 2.0 1.5 1.0 0.5 0.0 -0.5
f1 (ppm)

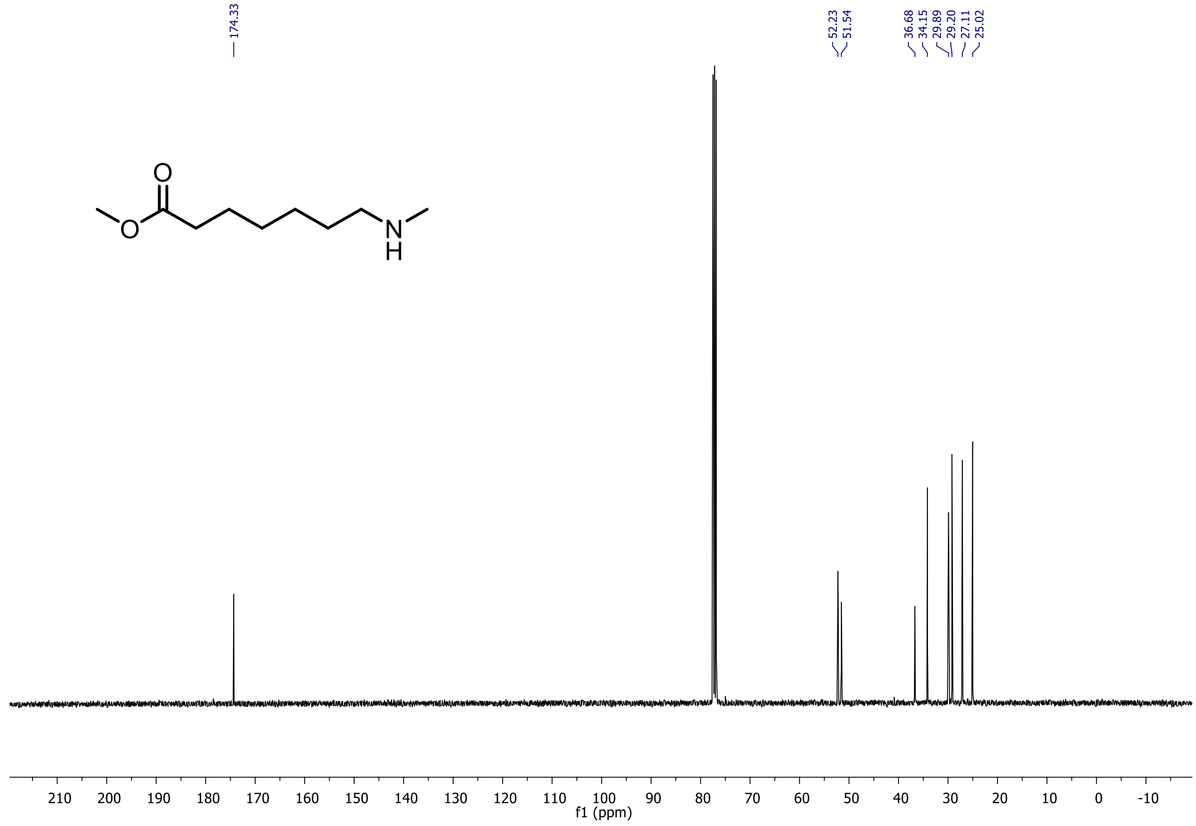

174.33
52.23 51.54
36.68 34.15 29.89 29.20 27.11 25.02
O
O
N
H
210 200 190 180 170 160 150 140 130 120 110 100 90 80 70 60 50 40 30 20 10 0 -10
f1 (ppm)

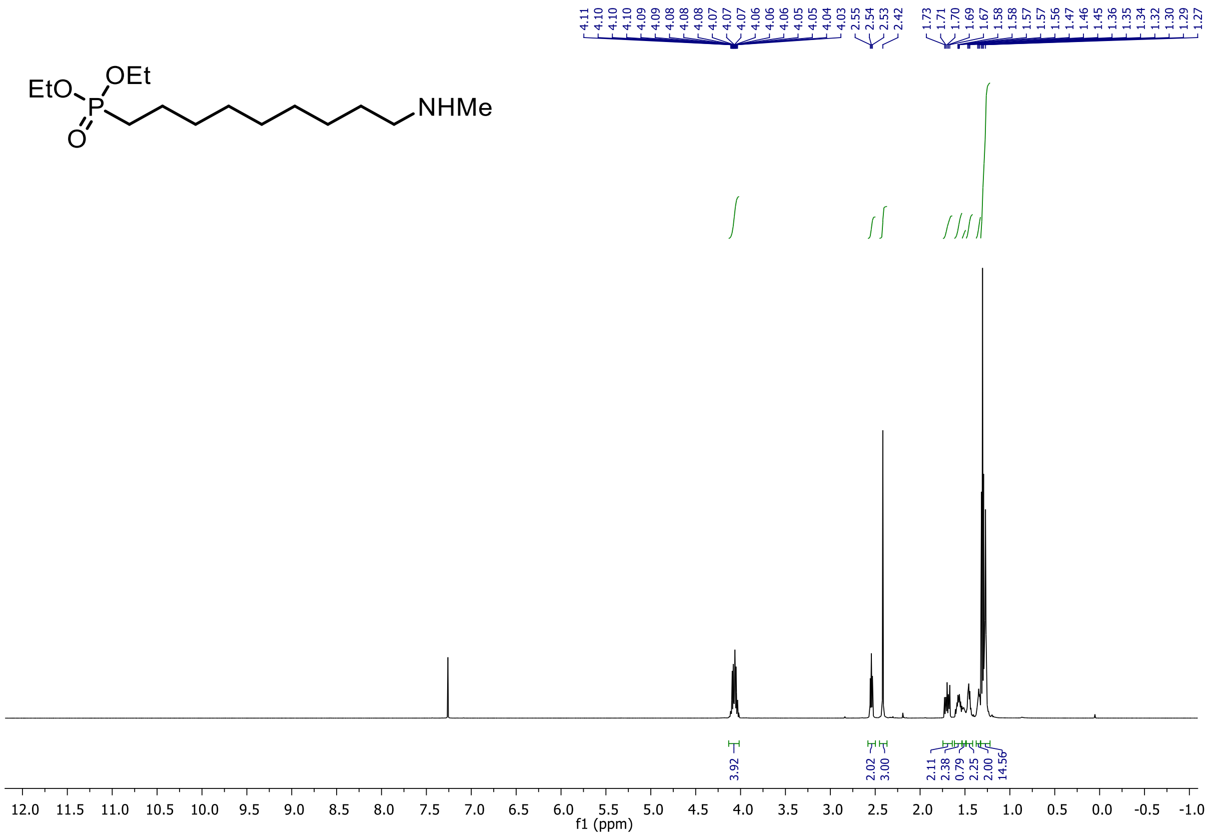
EtO
OEt
P
O
NHMe
f1 (ppm)

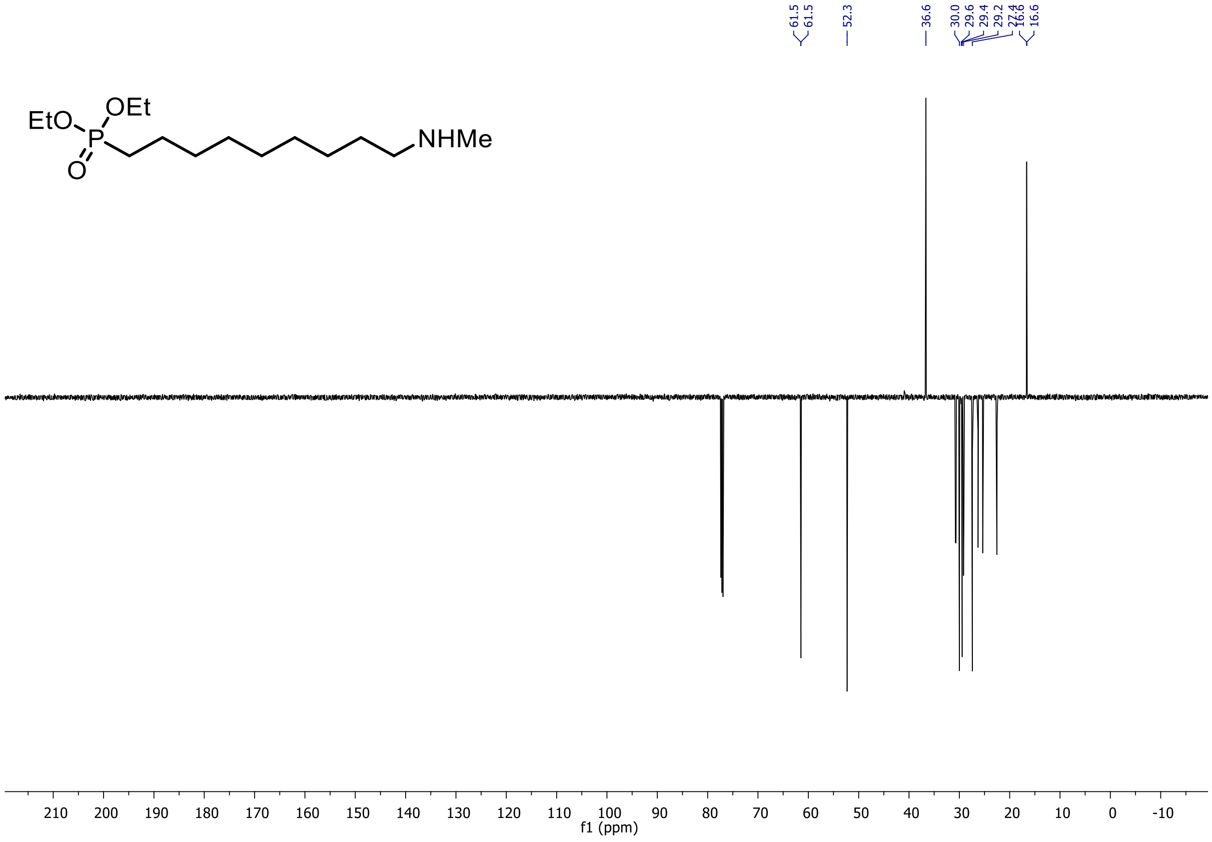
EtO
OEt
P
O
NHMe
f1 (ppm)

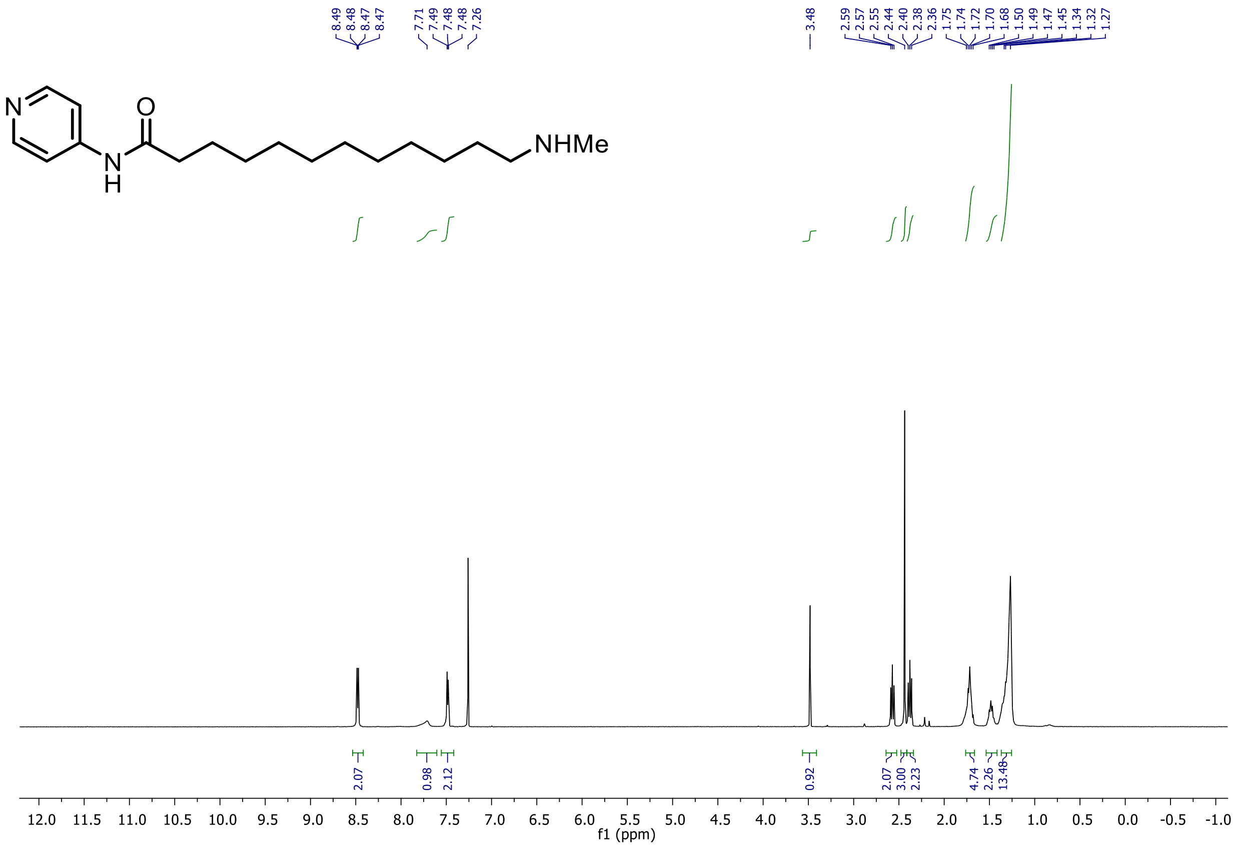

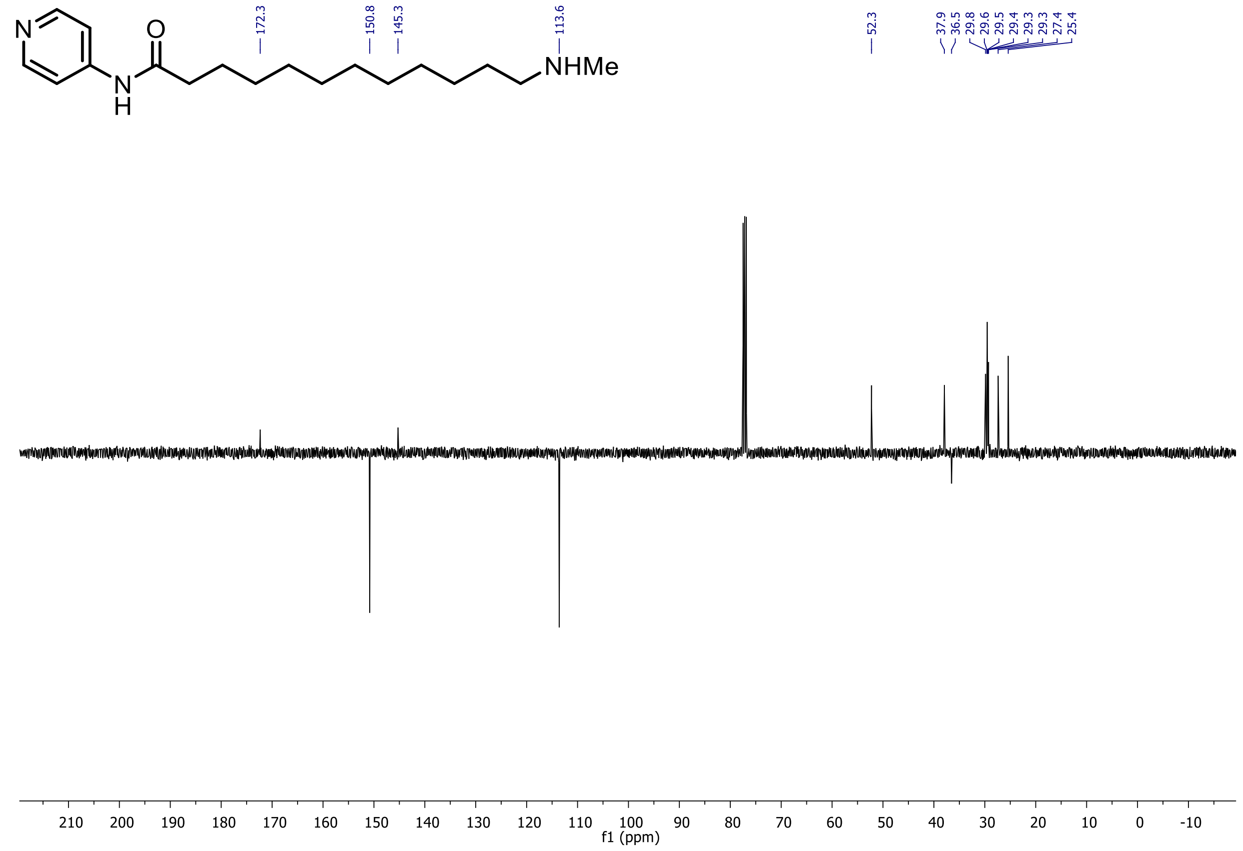

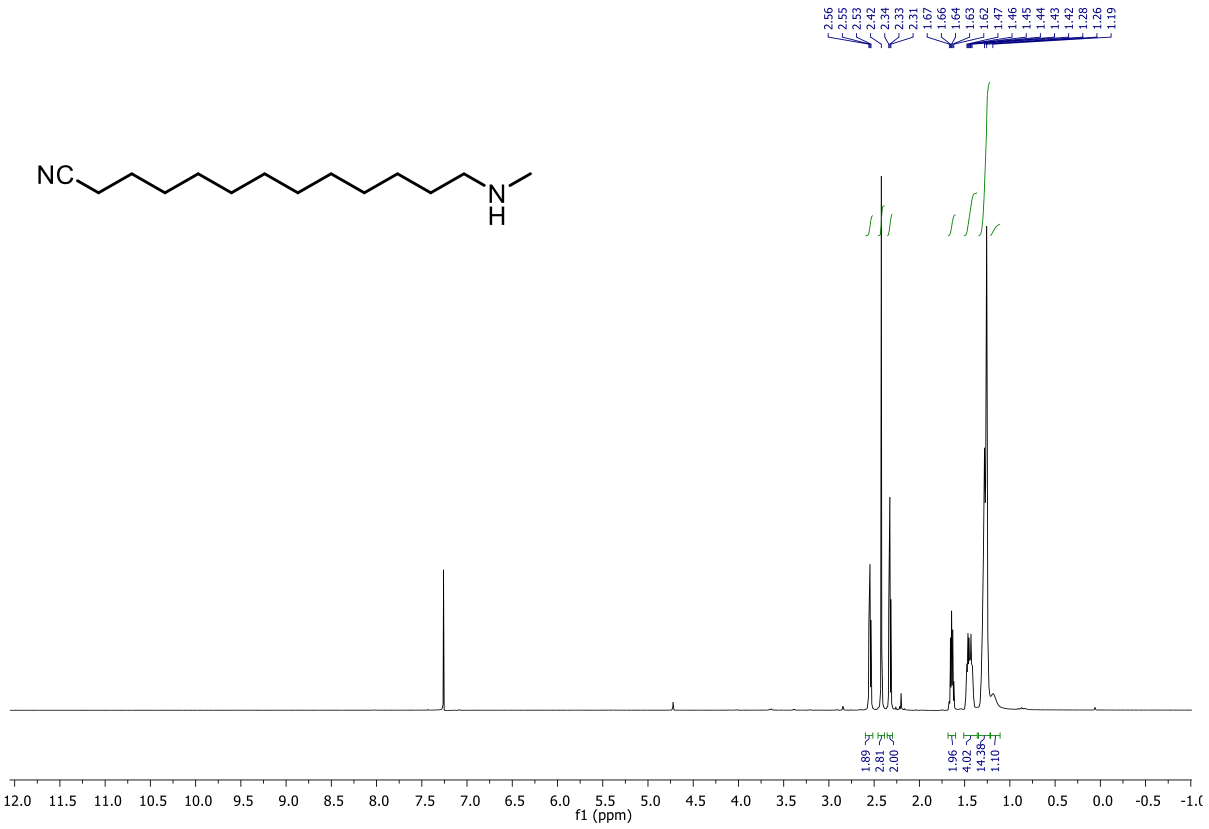

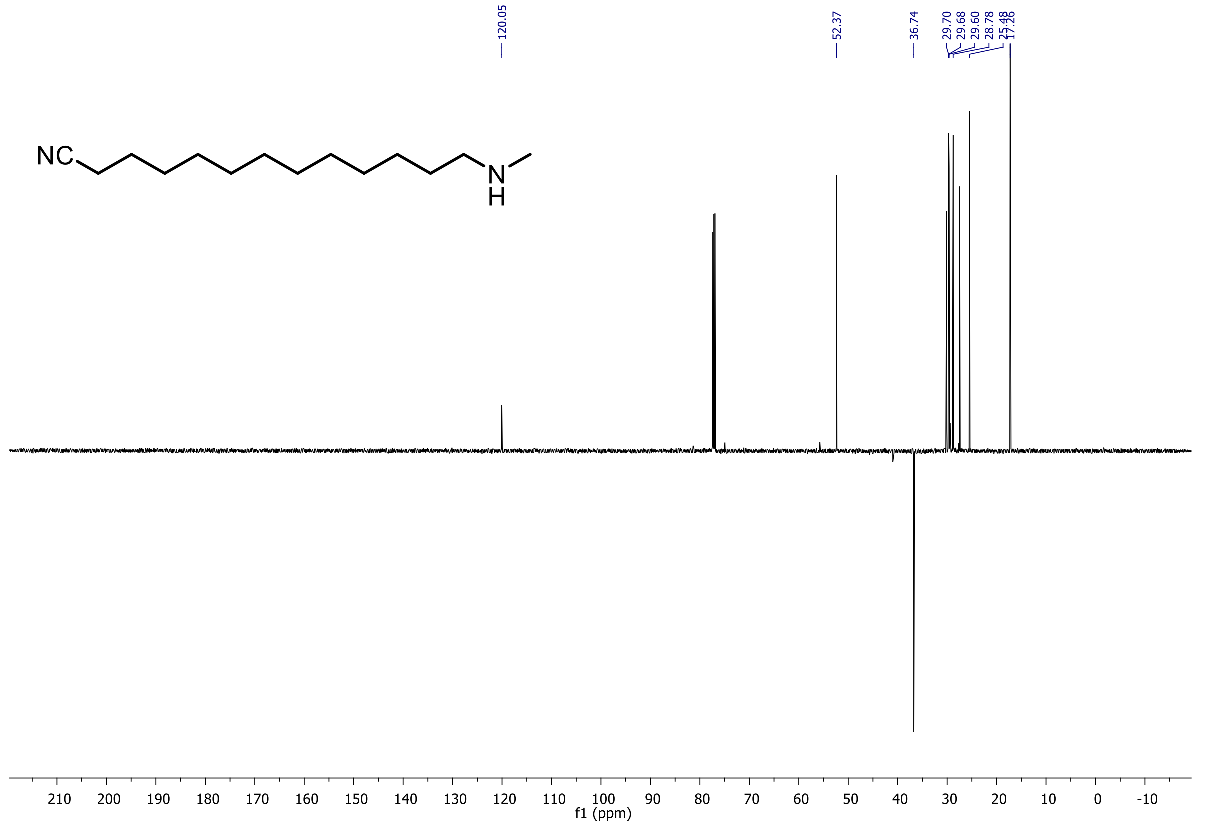

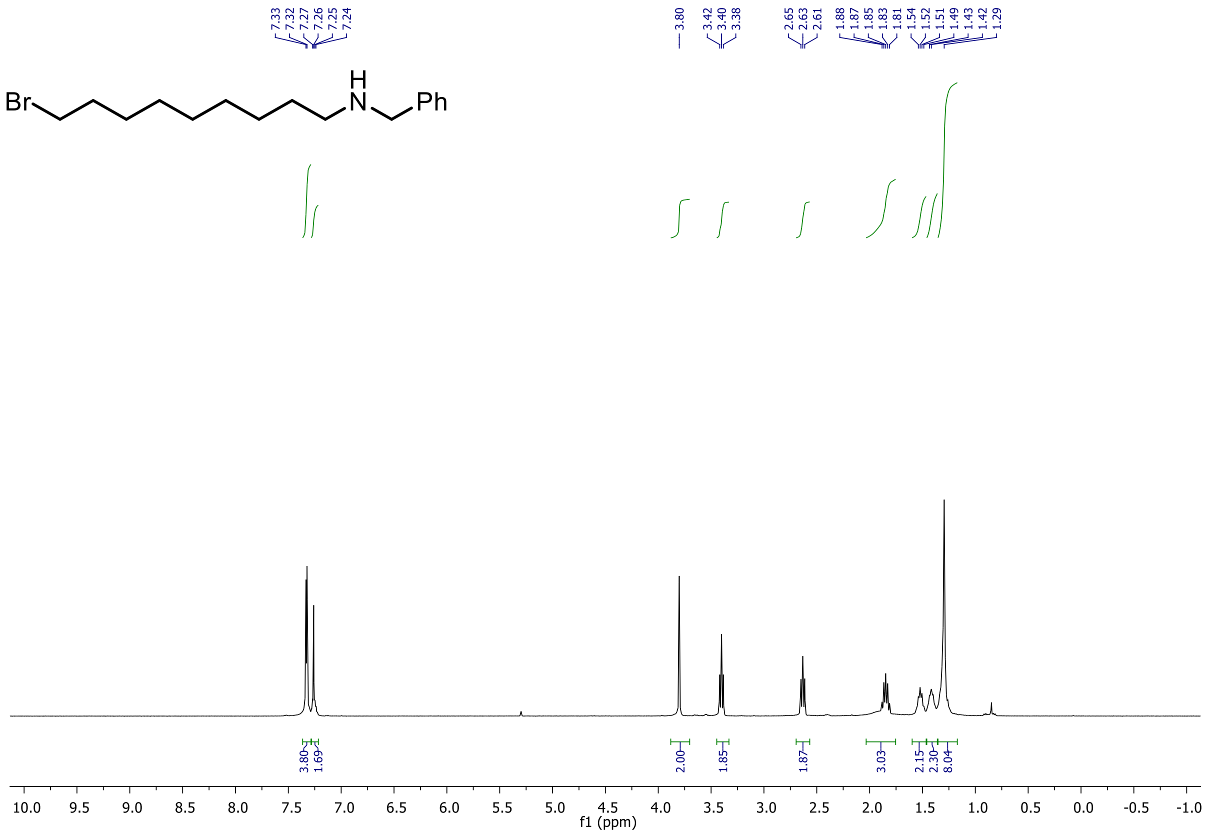
7.33
7.32
7.27
7.26
7.25
7.24
3.80
3.42
3.40
3.38
2.65
2.63
2.61
1.88
1.87
1.85
1.83
1.81
1.54
1.52
1.51
1.49
1.43
1.42
1.29
Br
H
N
Ph
3.80
1.69
2.00
1.85
1.87
3.03
2.15
2.30
8.04
f1 (ppm)

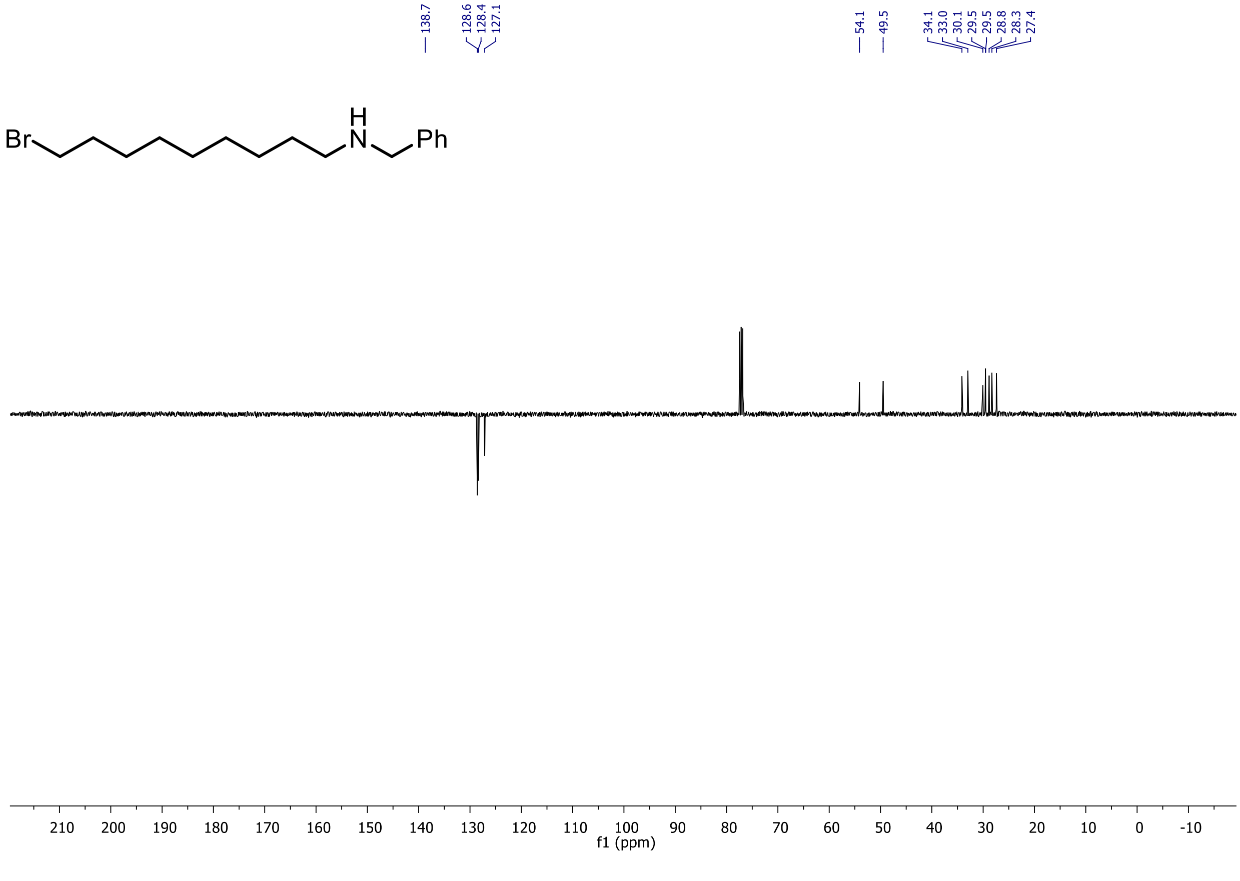
138.7
128.6
128.4
127.1
54.1
49.5
34.1
33.0
30.1
29.5
29.5
28.8
28.3
27.4
Br
H
N
Ph
f1 (ppm)

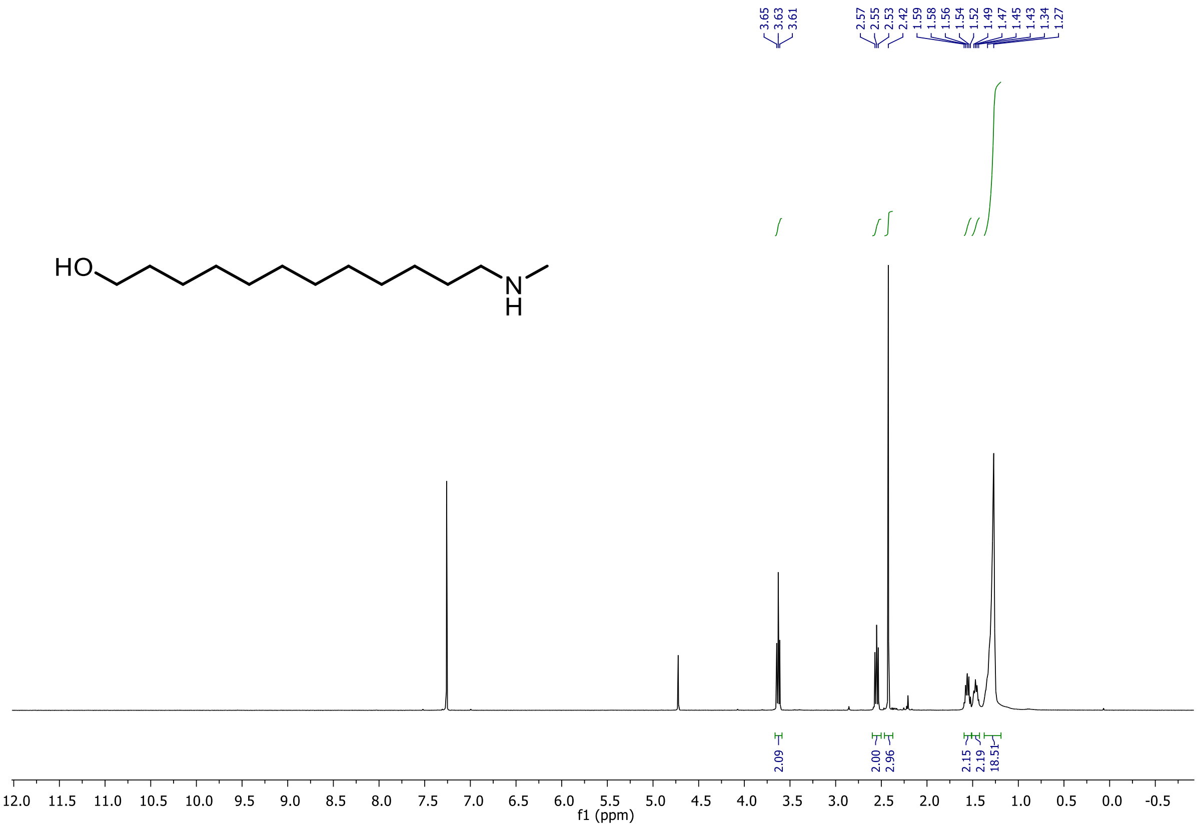
3.65
3.63
3.61
2.57
2.55
2.53
2.42
1.59
1.58
1.56
1.54
1.52
1.49
1.47
1.45
1.43
1.34
1.27
HO
N
H
2.09
2.00
2.96
2.15
2.19
18.51
12.0 11.5 11.0 10.5 10.0 9.5 9.0 8.5 8.0 7.5 7.0 6.5 6.0 5.5 5.0 4.5 4.0 3.5 3.0 2.5 2.0 1.5 1.0 0.5 0.0 -0.5
f1 (ppm)

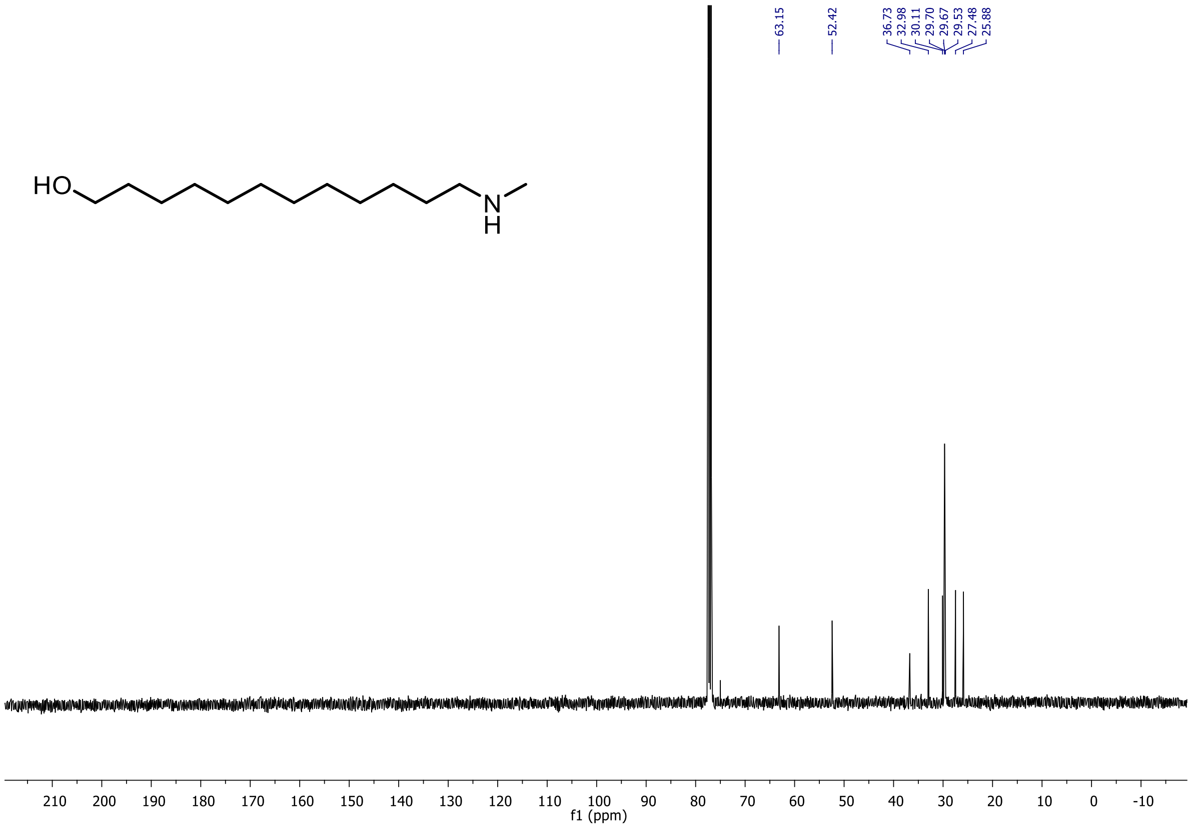
63.15
52.42
36.73
32.98
30.11
29.70
29.67
29.53
27.48
25.88
HO
N
H
210 200 190 180 170 160 150 140 130 120 110 100 90 80 70 60 50 40 30 20 10 0 -10
f1 (ppm)

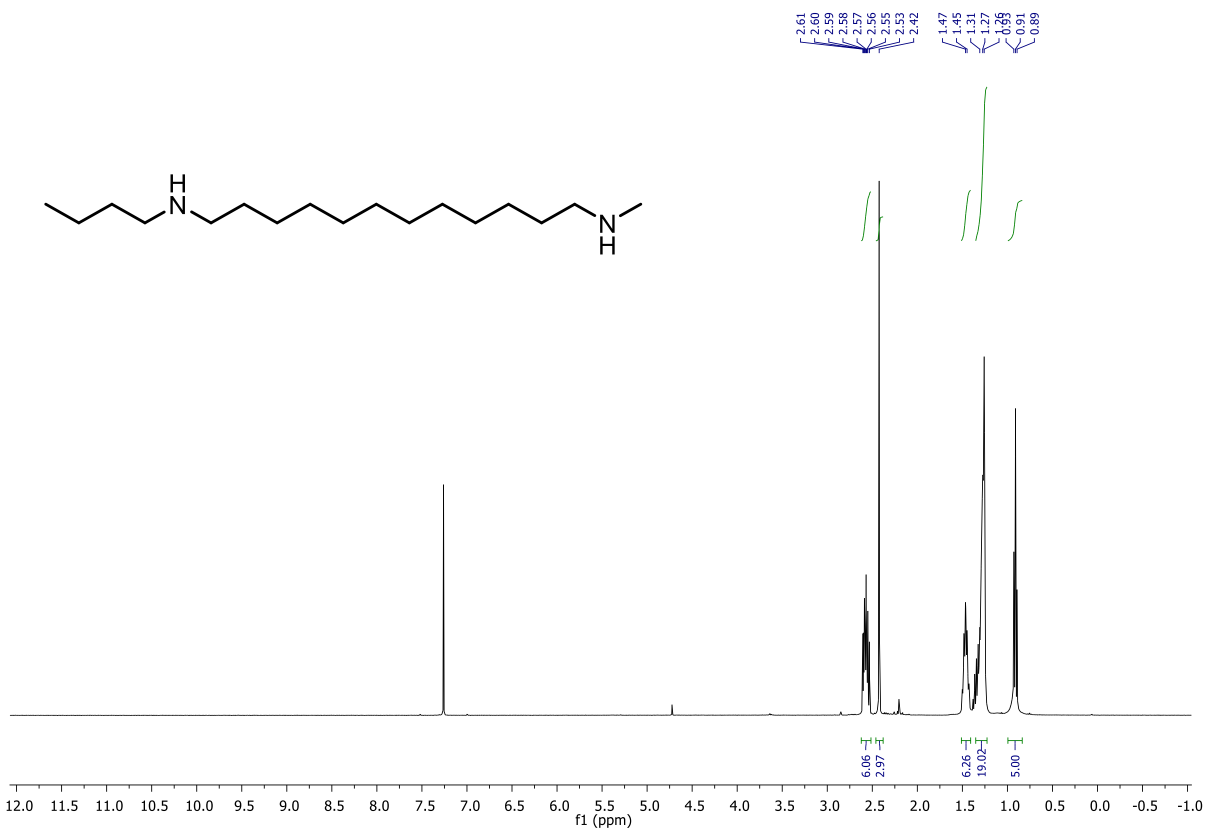
2.61
2.60
2.59
2.58
2.57
2.56
2.55
2.53
2.42
1.47
1.45
1.31
1.27
1.26
0.93
0.91
0.89
6.06
2.97
6.26
19.02
5.00
12.0 11.5 11.0 10.5 10.0 9.5 9.0 8.5 8.0 7.5 7.0 6.5 6.0 5.5 5.0 4.5 4.0 3.5 3.0 2.5 2.0 1.5 1.0 0.5 0.0 -0.5 -1.0
f1 (ppm)

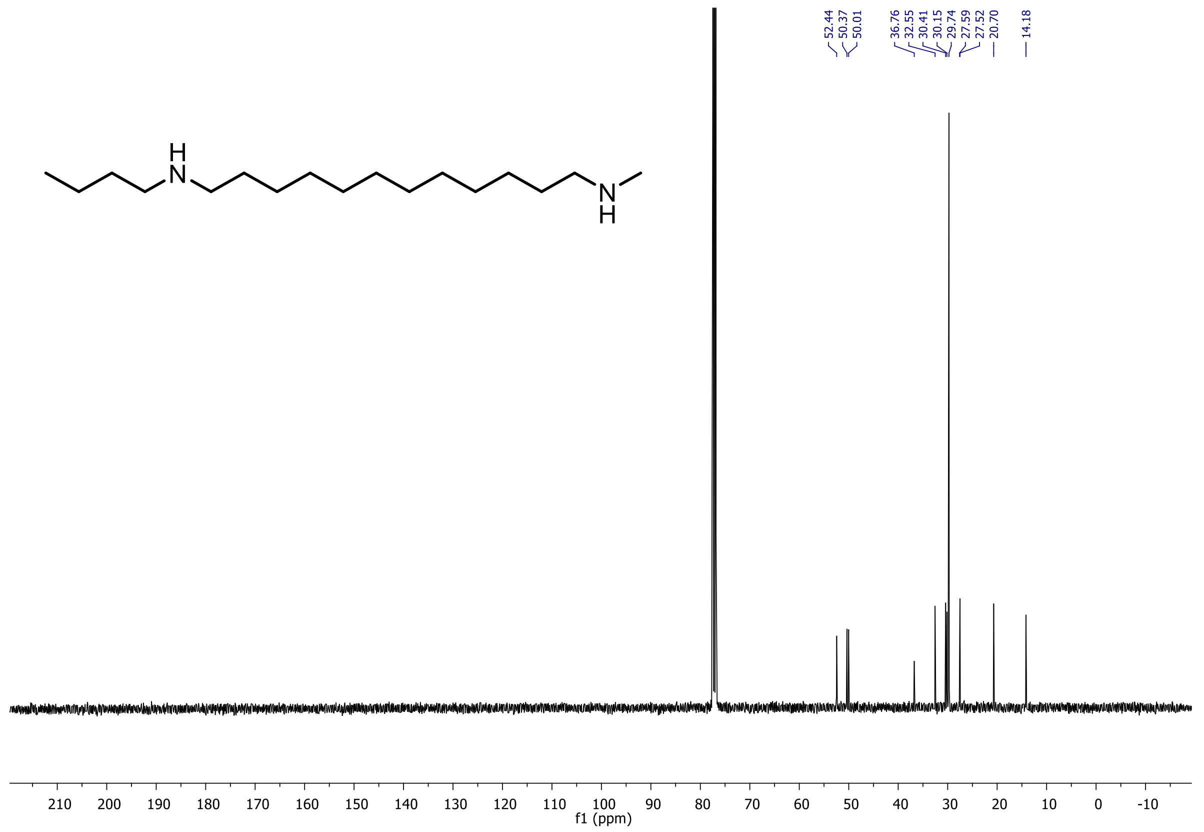
52.44
50.37
50.01
36.76
32.55
30.41
30.15
29.74
27.59
27.52
20.70
14.18
210 200 190 180 170 160 150 140 130 120 110 100 90 80 70 60 50 40 30 20 10 0 -10
f1 (ppm)

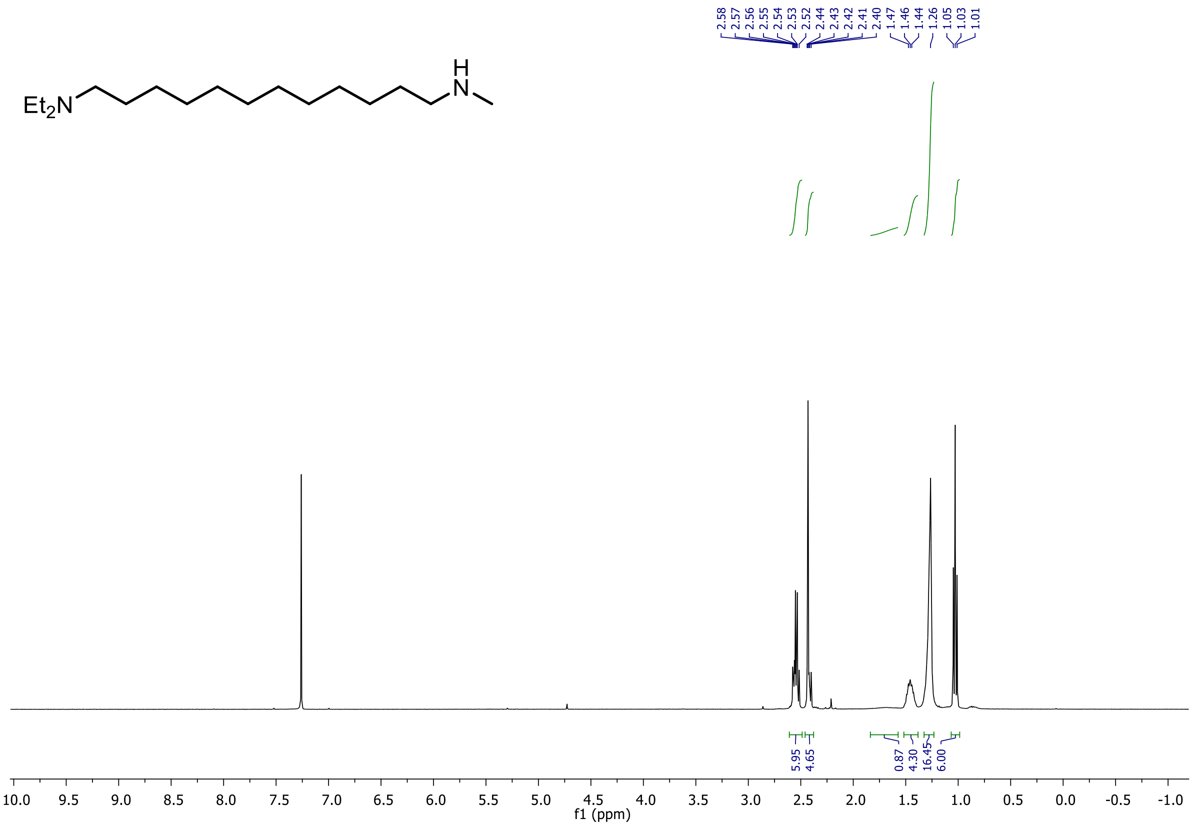
Et2N
H
N
2.58 2.57 2.56 2.55 2.54 2.53 2.52 2.44 2.43 2.42 2.41 2.40 1.47 1.46 1.44 1.26 1.05 1.03 1.01
5.95 4.65 0.87 4.30 16.45 6.00
f1 (ppm)

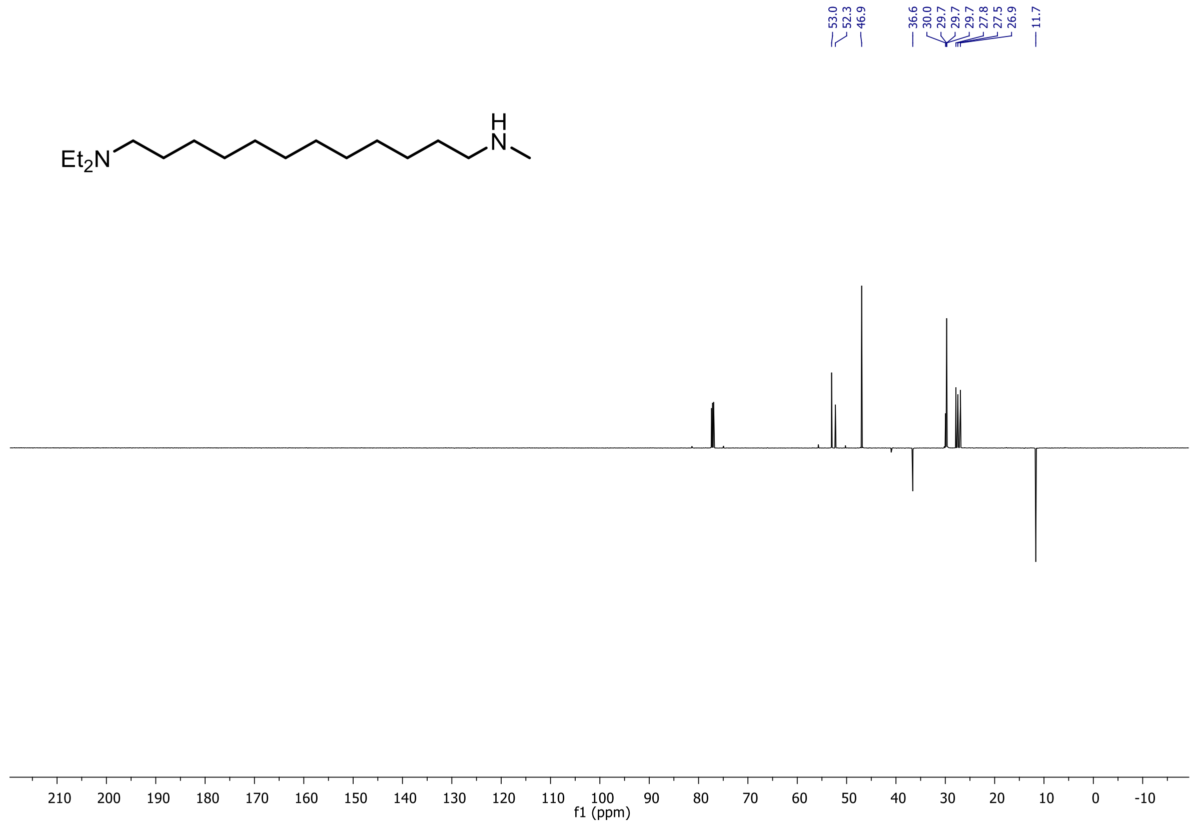
Et2N
H
N
53.0 52.3 46.9 36.6 30.0 29.7 29.7 27.8 27.5 26.9 11.7
f1 (ppm)

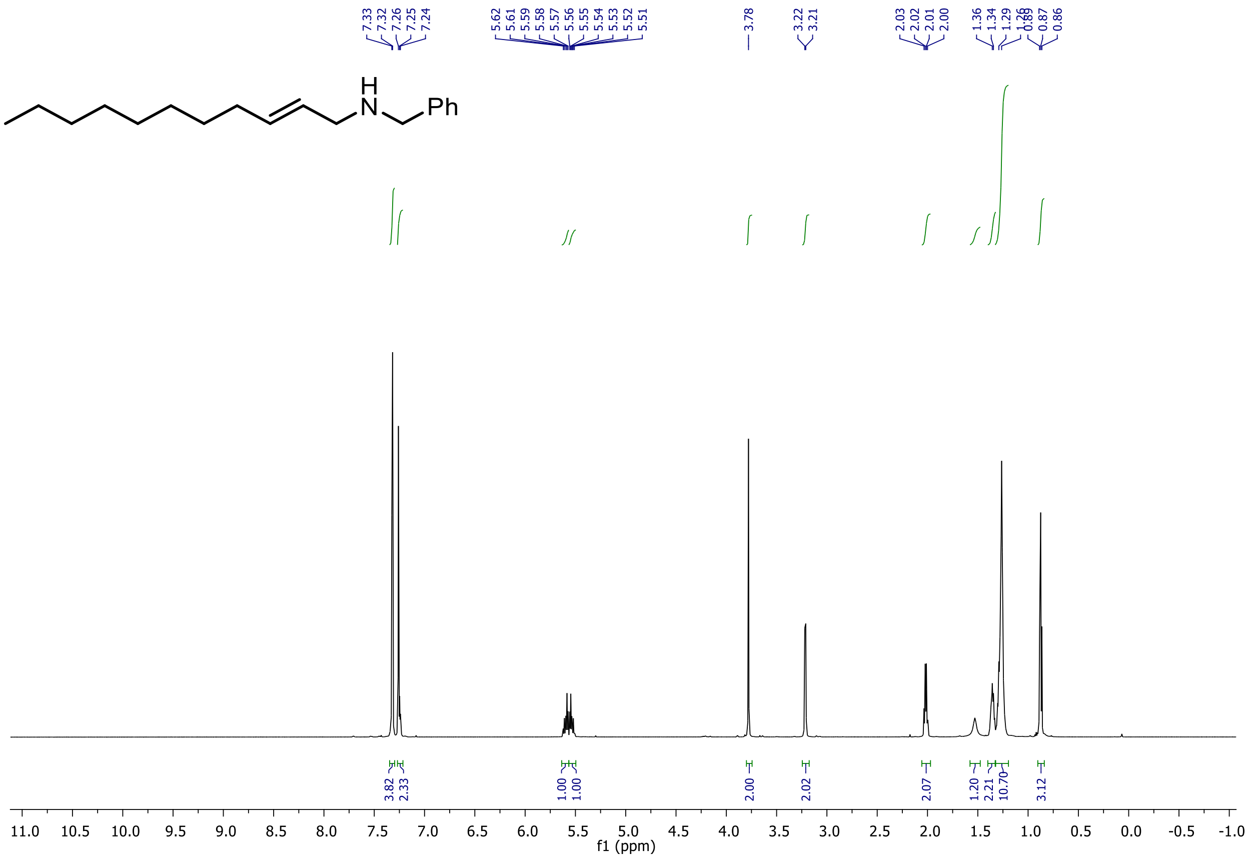

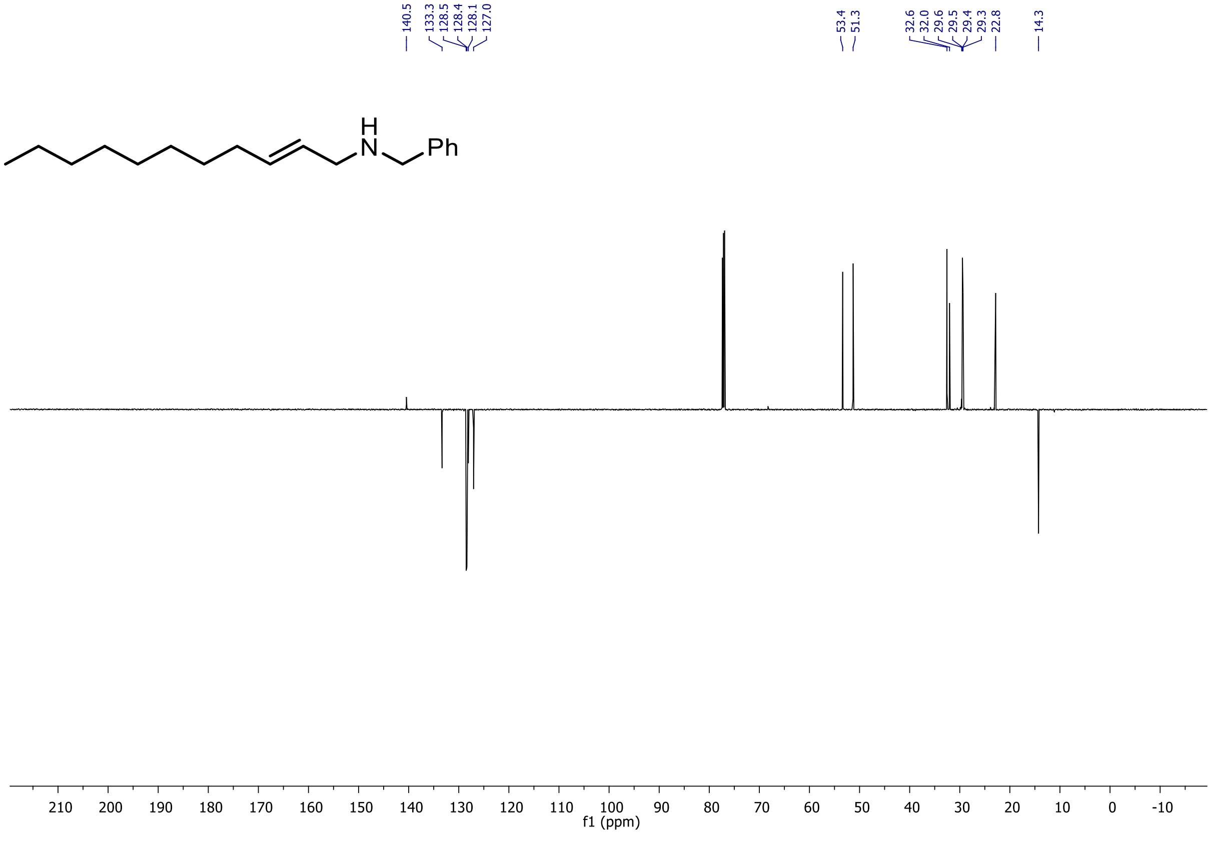

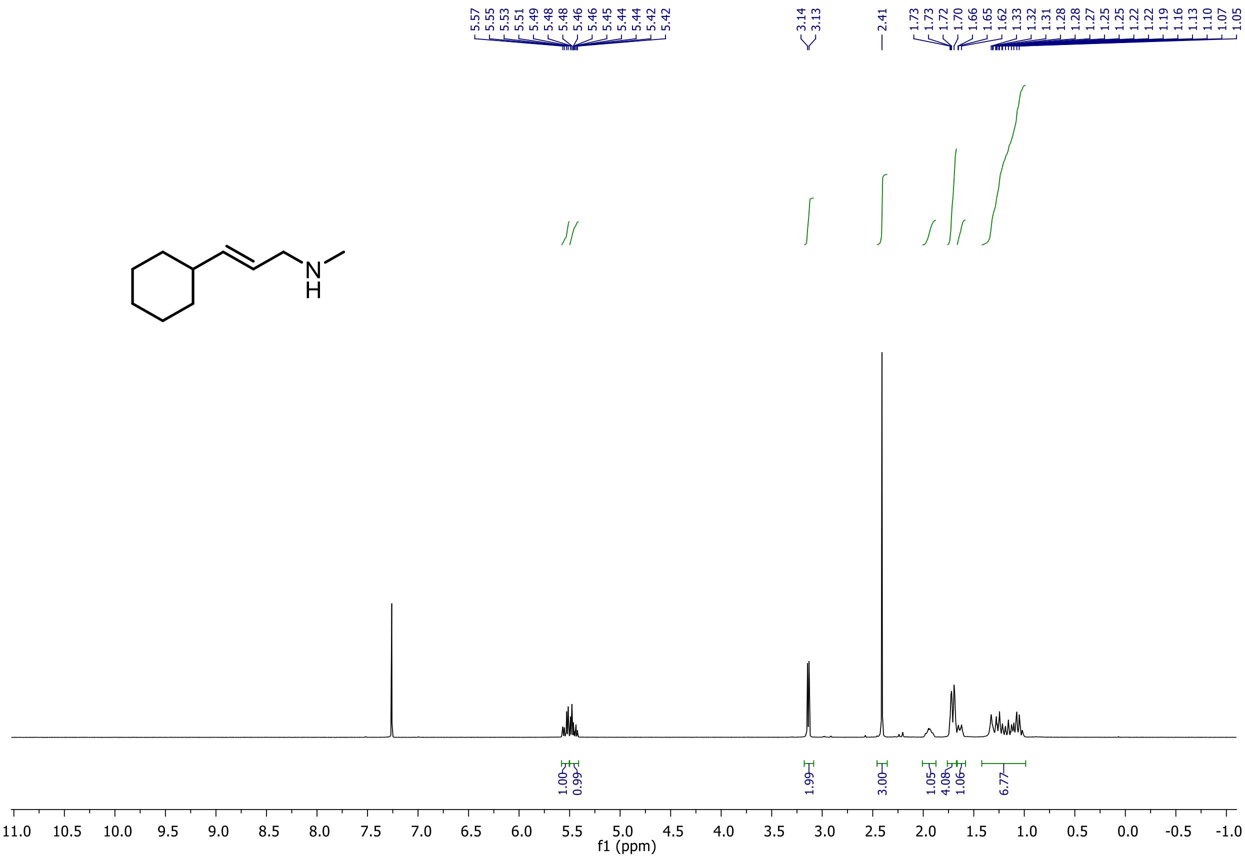
5.57
5.55
5.53
5.51
5.49
5.48
5.48
5.46
5.46
5.45
5.44
5.44
5.42
5.42
3.14
3.13
2.41
1.73
1.73
1.72
1.70
1.66
1.65
1.62
1.33
1.32
1.31
1.28
1.27
1.25
1.25
1.22
1.22
1.19
1.16
1.13
1.10
1.07
1.05
N
H
1.00
0.99
1.99
3.00
1.05
4.08
1.06
6.77
11.0 10.5 10.0 9.5 9.0 8.5 8.0 7.5 7.0 6.5 6.0 5.5 5.0 4.5 4.0 3.5 3.0 2.5 2.0 1.5 1.0 0.5 0.0 -0.5 -1.0
f1 (ppm)

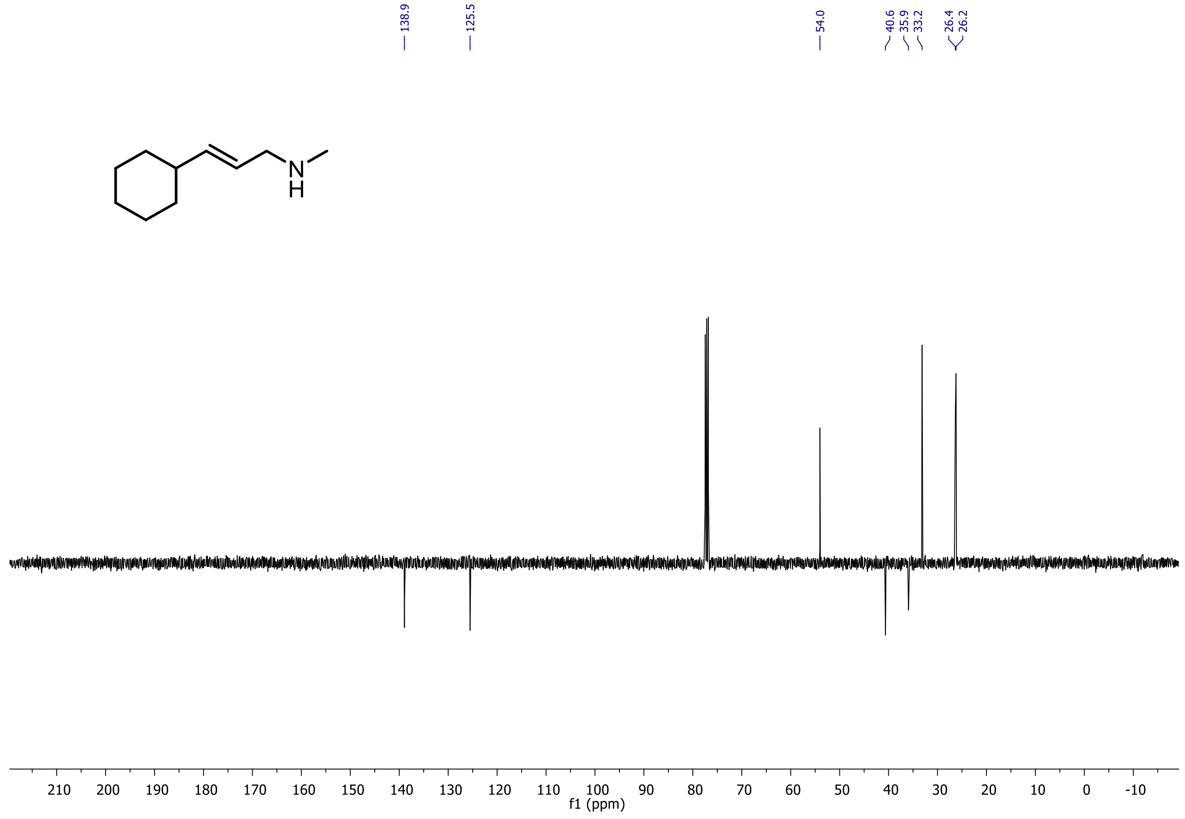
138.9
125.5
54.0
40.6
35.9
33.2
26.4
26.2
N
H
210 200 190 180 170 160 150 140 130 120 110 100 90 80 70 60 50 40 30 20 10 0 -10
f1 (ppm)

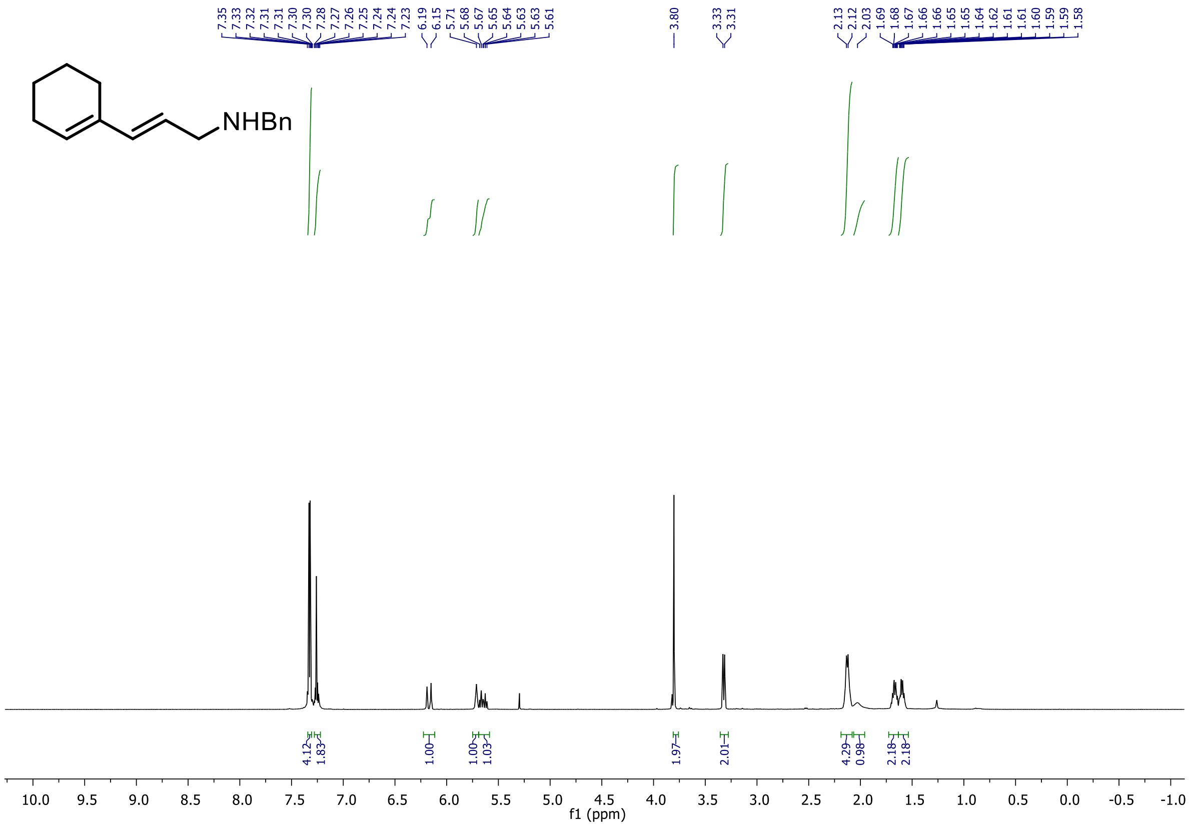
NHBn
7.35
7.33
7.32
7.31
7.31
7.30
7.28
7.27
7.26
7.25
7.24
7.24
7.23
6.19
6.15
5.71
5.68
5.67
5.65
5.64
5.63
5.63
5.61
3.80
3.33
3.31
2.13
2.12
2.03
1.69
1.68
1.67
1.66
1.66
1.65
1.65
1.64
1.62
1.61
1.61
1.60
1.59
1.59
1.58
4.12
1.83
1.00
1.00
1.03
1.97
2.01
4.29
0.98
2.18
2.18
f1 (ppm)

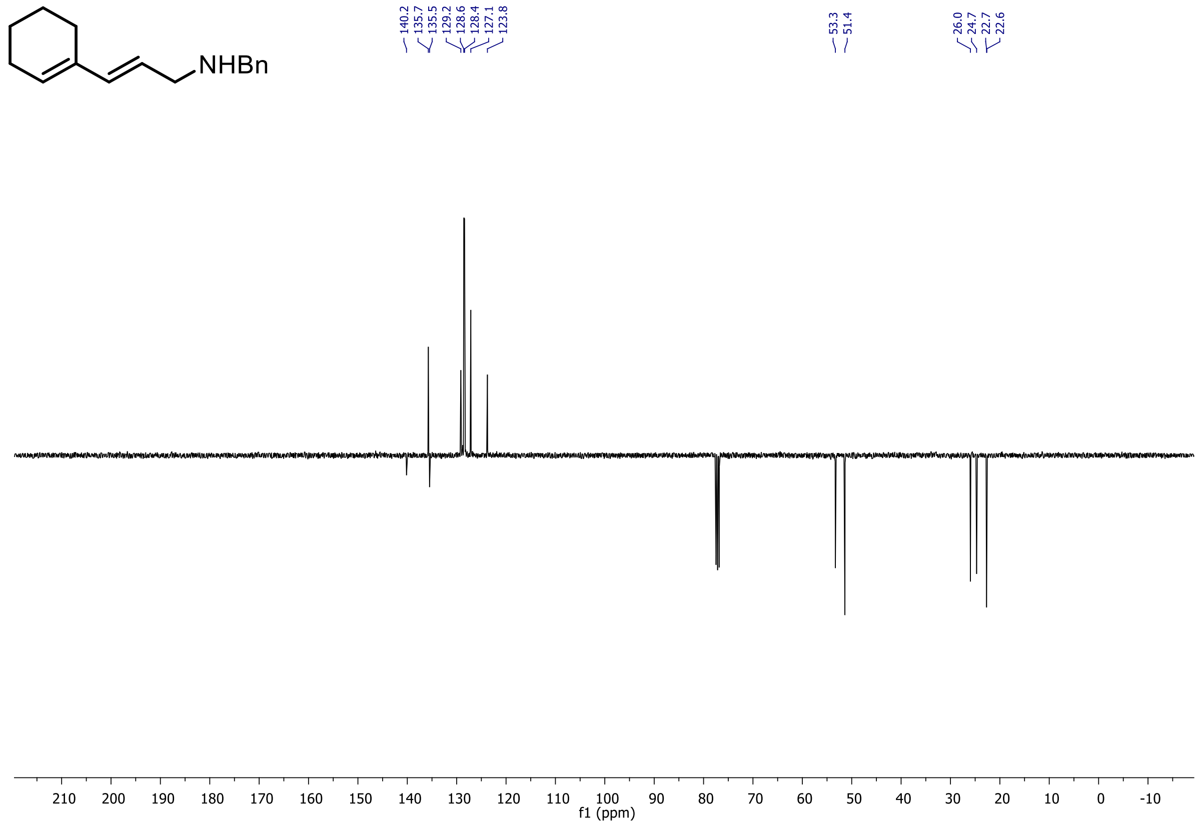
NHBn
140.2
135.7
135.5
129.2
128.6
128.4
127.1
123.8
53.3
51.4
26.0
24.7
22.7
22.6
f1 (ppm)

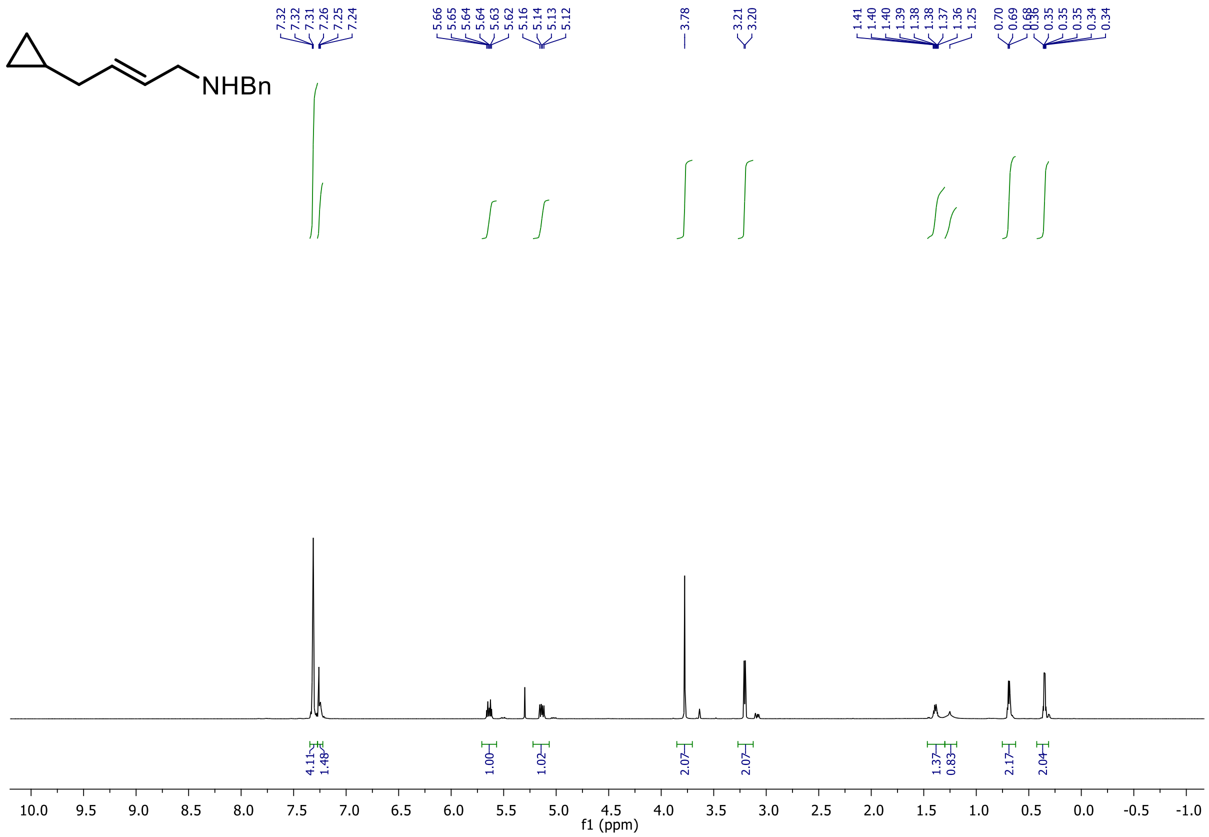
NHBn
7.32
7.32
7.31
7.26
7.25
7.24
5.66
5.65
5.64
5.64
5.63
5.62
5.16
5.14
5.13
5.12
3.78
3.21
3.20
1.41
1.40
1.39
1.38
1.38
1.37
1.36
1.25
0.70
0.69
0.35
0.35
0.34
0.34
4.11
1.48
1.00
1.02
2.07
2.07
1.37
0.83
2.17
2.04
f1 (ppm)

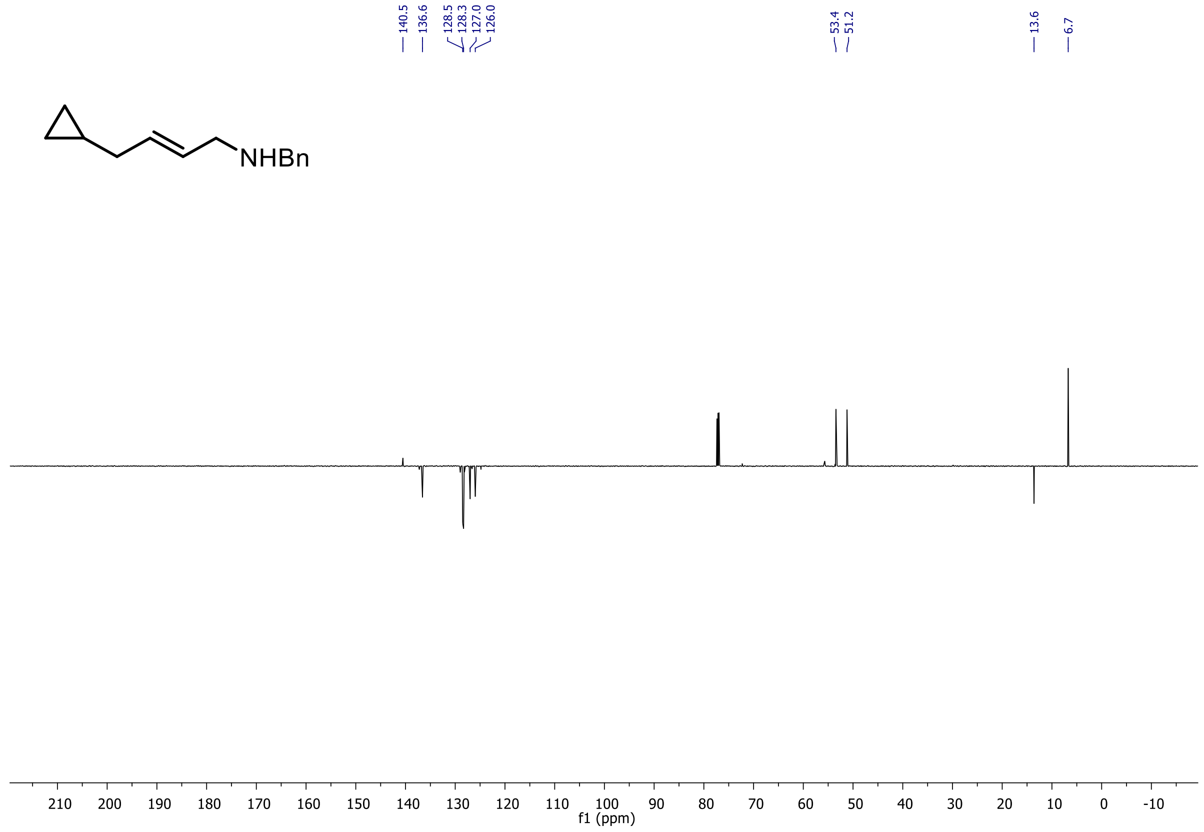
140.5
136.6
128.5
128.3
127.0
126.0
53.4
51.2
13.6
6.7
NHBn
f1 (ppm)

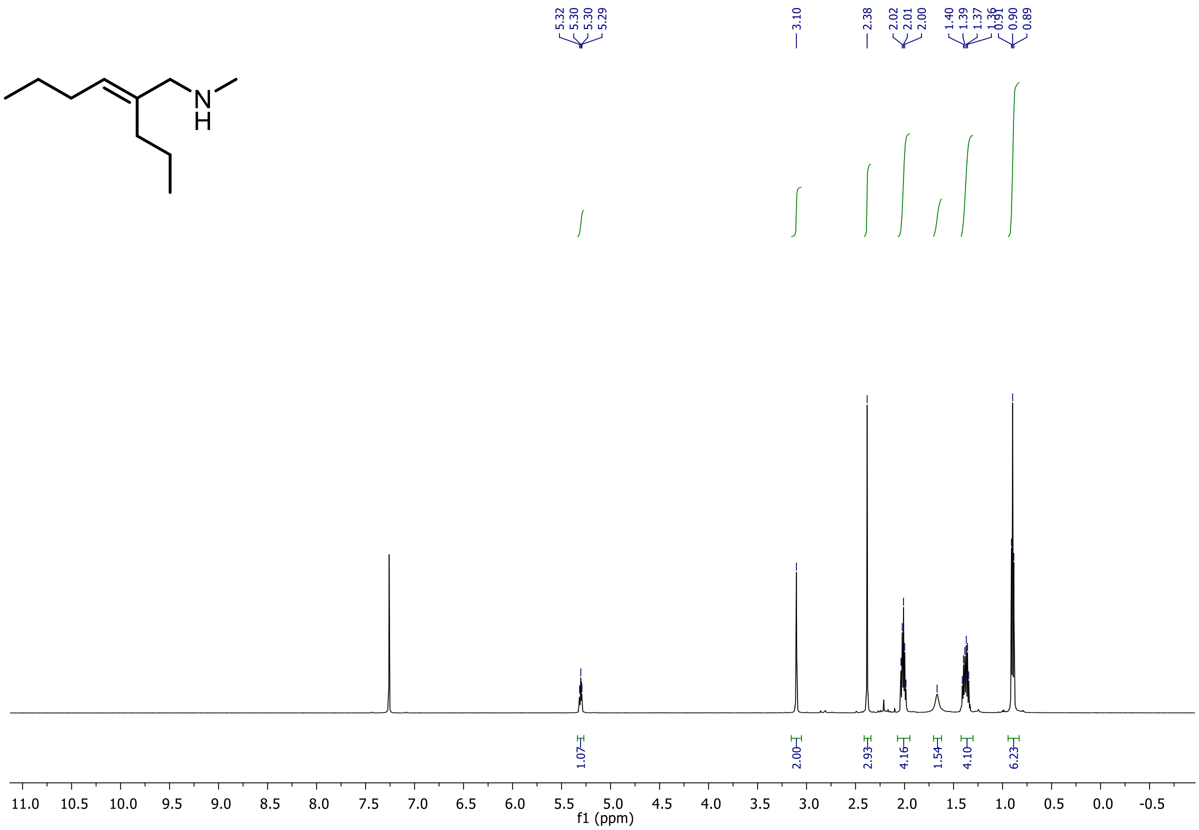

5.32
5.30
5.30
5.29
3.10
2.38
2.02
2.01
2.00
1.40
1.39
1.37
1.36
0.91
0.90
0.89
1.07
2.00
2.93
4.16
1.54
4.10
6.23
11.0 10.5 10.0 9.5 9.0 8.5 8.0 7.5 7.0 6.5 6.0 5.5 5.0 4.5 4.0 3.5 3.0 2.5 2.0 1.5 1.0 0.5 0.0 -0.5
f1 (ppm)

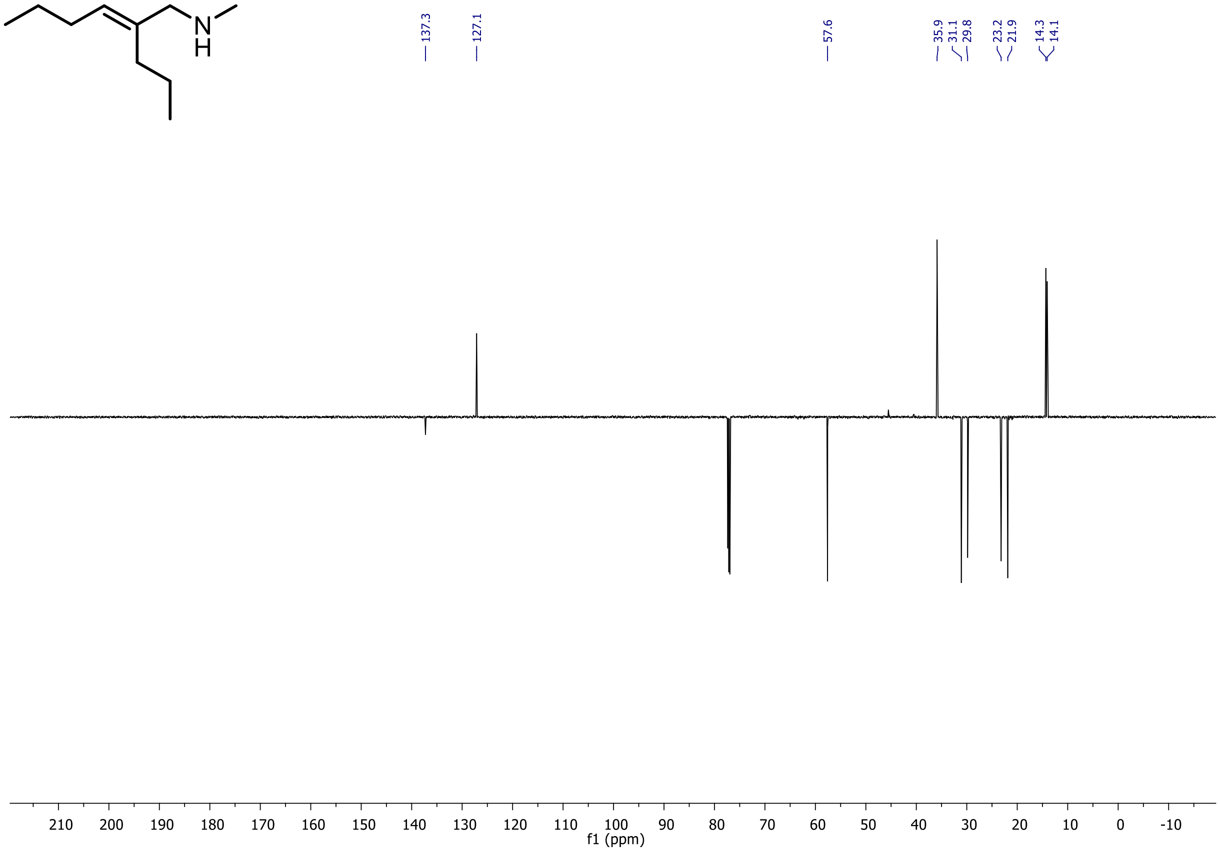

137.3
127.1
57.6
35.9
31.1
29.8
23.2
21.9
14.3
14.1
210 200 190 180 170 160 150 140 130 120 110 100 90 80 70 60 50 40 30 20 10 0 -10
f1 (ppm)

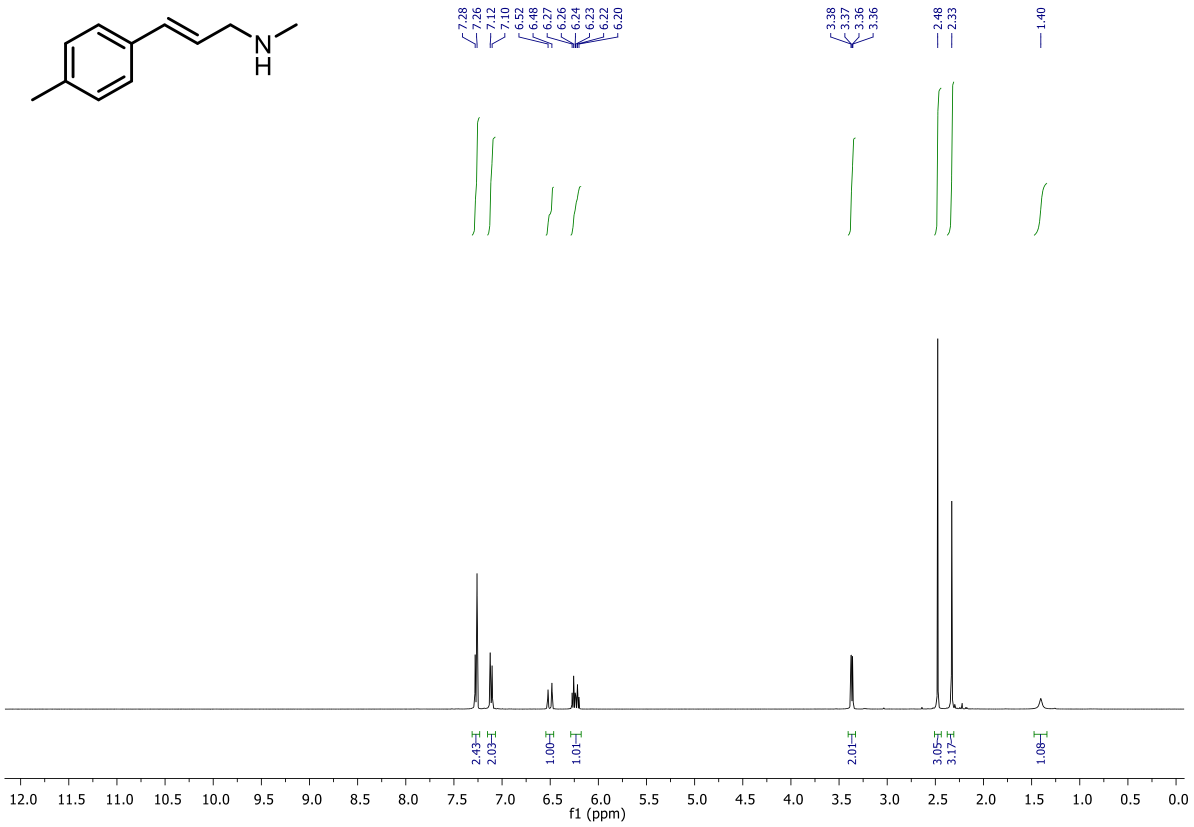
7.28
7.26
7.12
7.10
6.52
6.48
6.27
6.26
6.24
6.23
6.22
6.20
3.38
3.37
3.36
3.36
2.48
2.33
1.40
2.43
2.03
1.00
1.01
2.01
3.05
3.17
1.08
f1 (ppm)

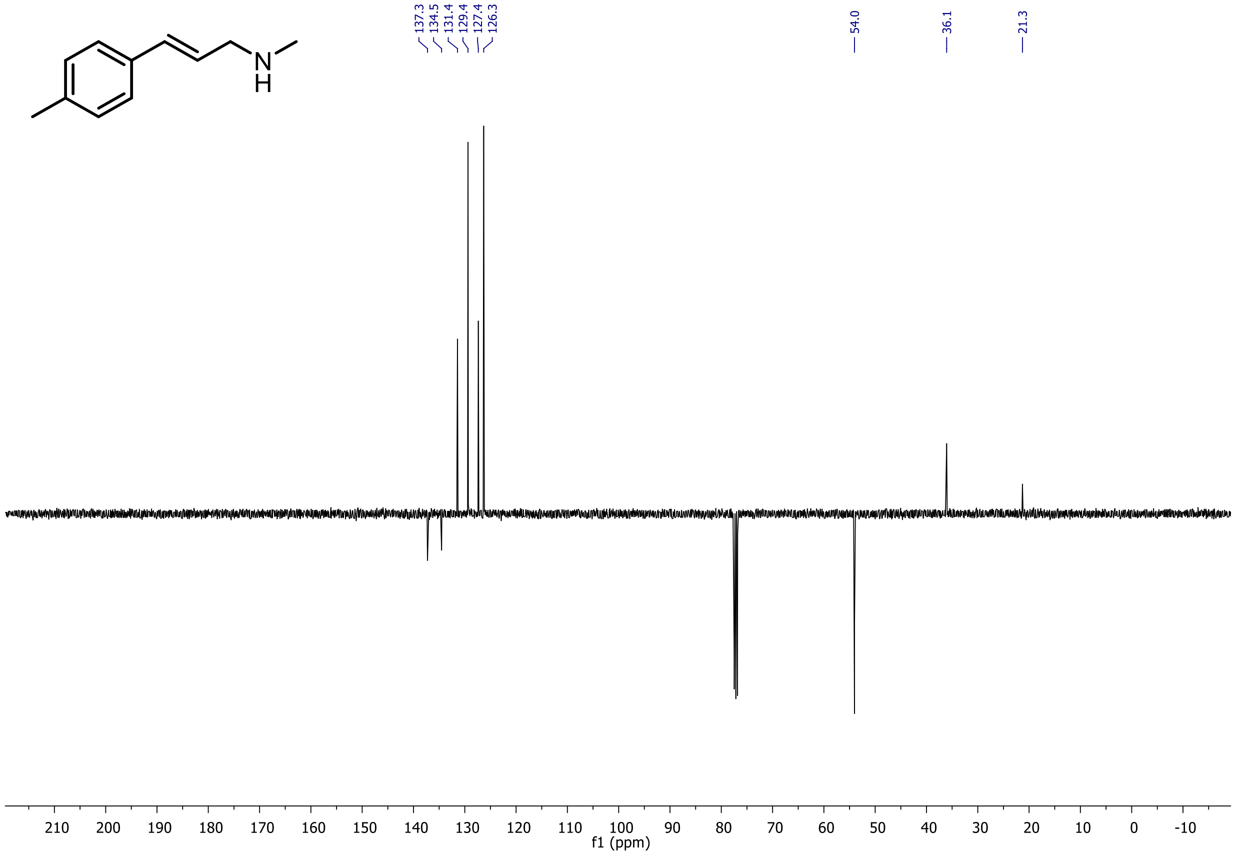
137.3
134.5
131.4
129.4
127.4
126.3
54.0
36.1
21.3
f1 (ppm)

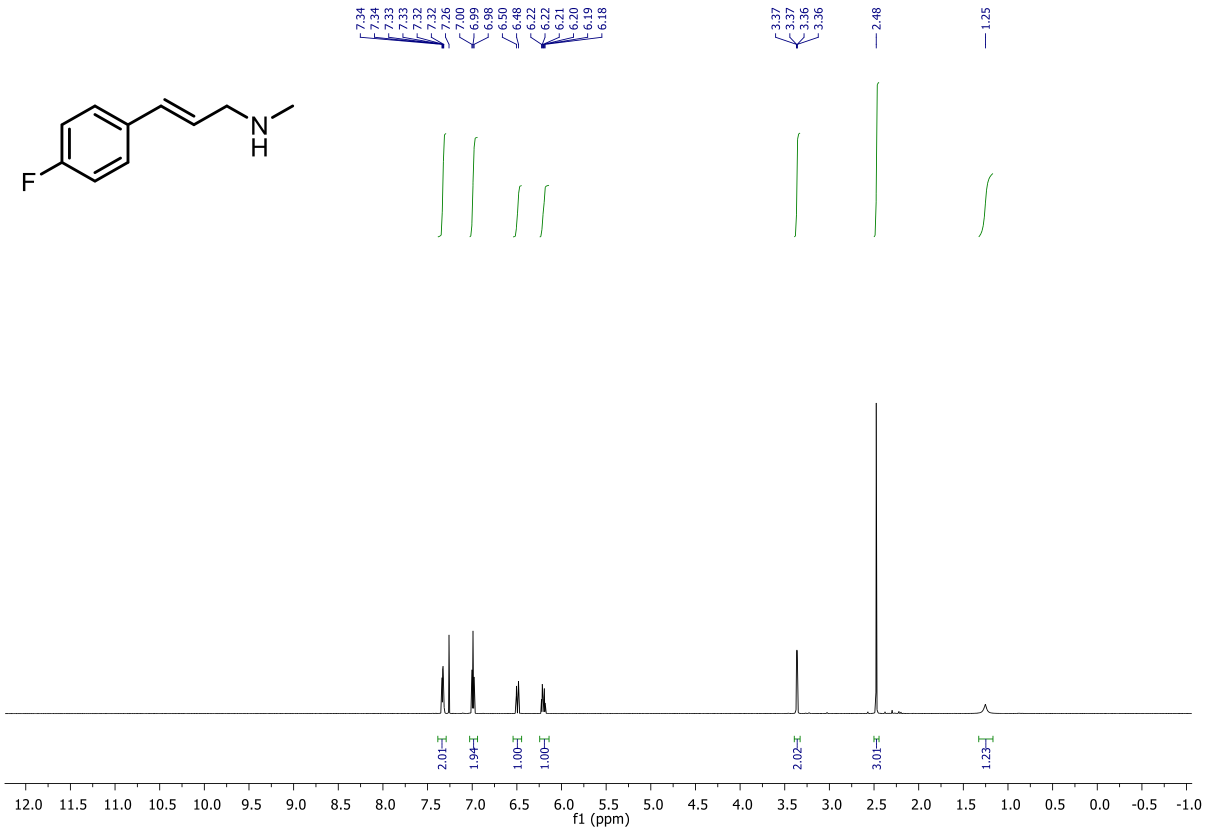
7.34
7.34
7.33
7.33
7.32
7.32
7.26
7.00
6.99
6.98
6.50
6.48
6.22
6.22
6.21
6.20
6.19
6.18
3.37
3.37
3.36
3.36
2.48
1.25
F
N
H
2.01
1.94
1.00
1.00
2.02
3.01
1.23
12.0 11.5 11.0 10.5 10.0 9.5 9.0 8.5 8.0 7.5 7.0 6.5 6.0 5.5 5.0 4.5 4.0 3.5 3.0 2.5 2.0 1.5 1.0 0.5 0.0 -0.5 -1.0
f1 (ppm)

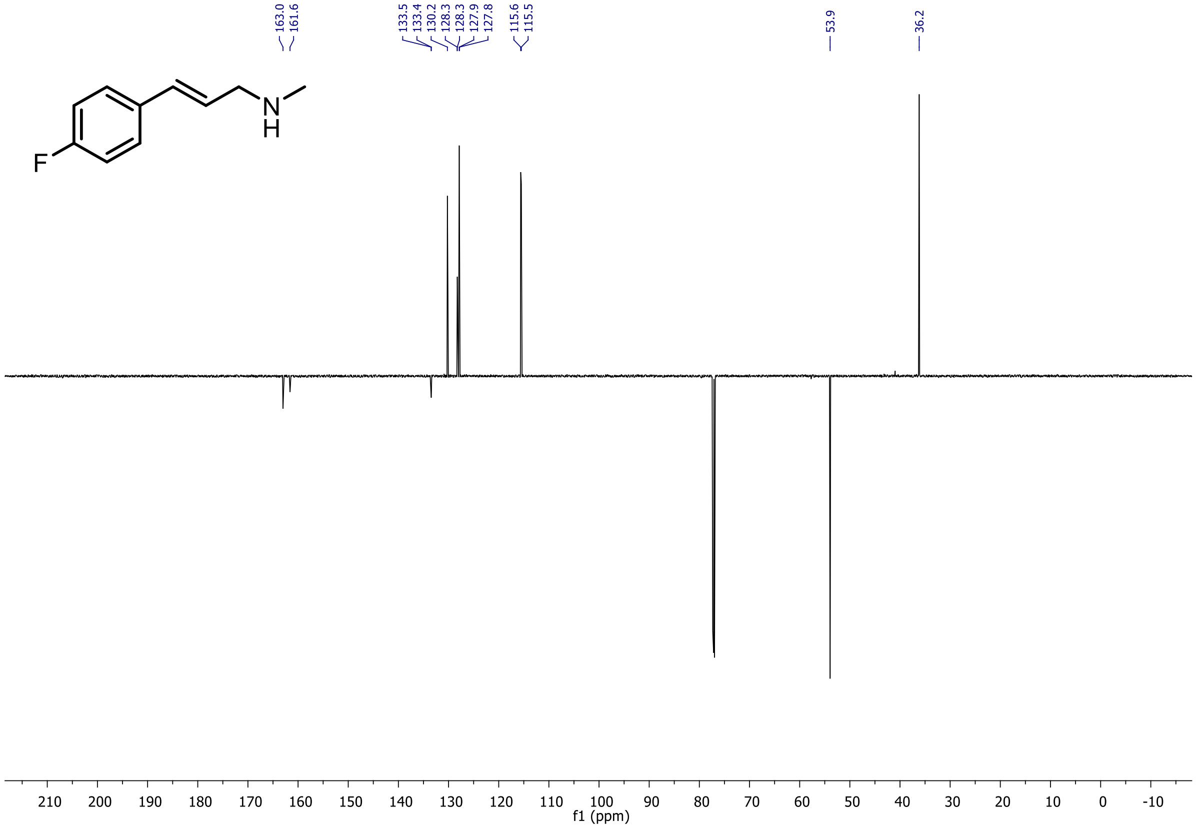
163.0
161.6
133.5
133.4
130.2
128.3
127.9
127.8
115.6
115.5
53.9
36.2
F
N
H
210 200 190 180 170 160 150 140 130 120 110 100 90 80 70 60 50 40 30 20 10 0 -10
f1 (ppm)

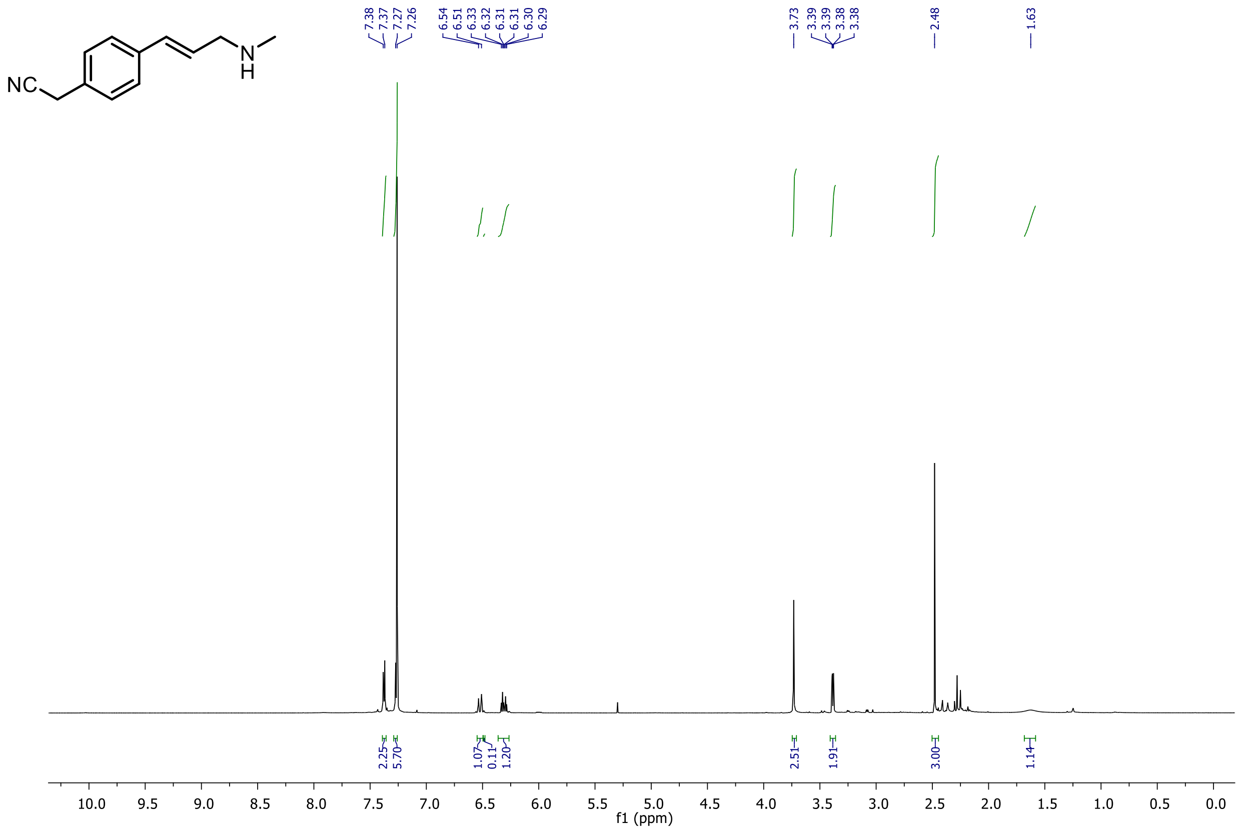
NC
N
H
7.38
7.37
7.27
7.26
6.54
6.51
6.33
6.32
6.31
6.31
6.30
6.29
3.73
3.39
3.39
3.38
3.38
2.48
1.63
2.25
5.70
1.07
0.11
1.20
2.51
1.91
3.00
1.14
f1 (ppm)

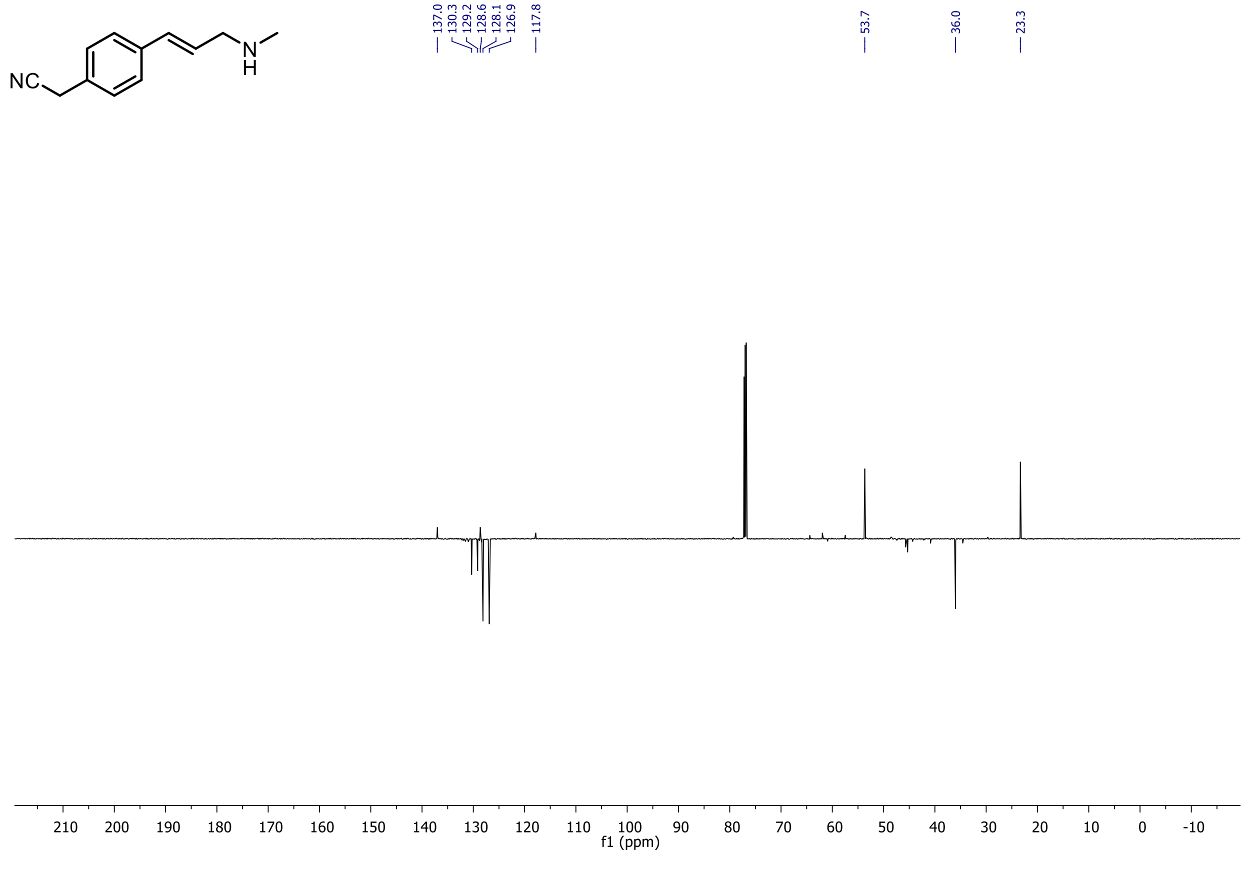
NC
N
H
137.0
130.3
129.2
128.6
128.1
126.9
117.8
53.7
36.0
23.3
f1 (ppm)

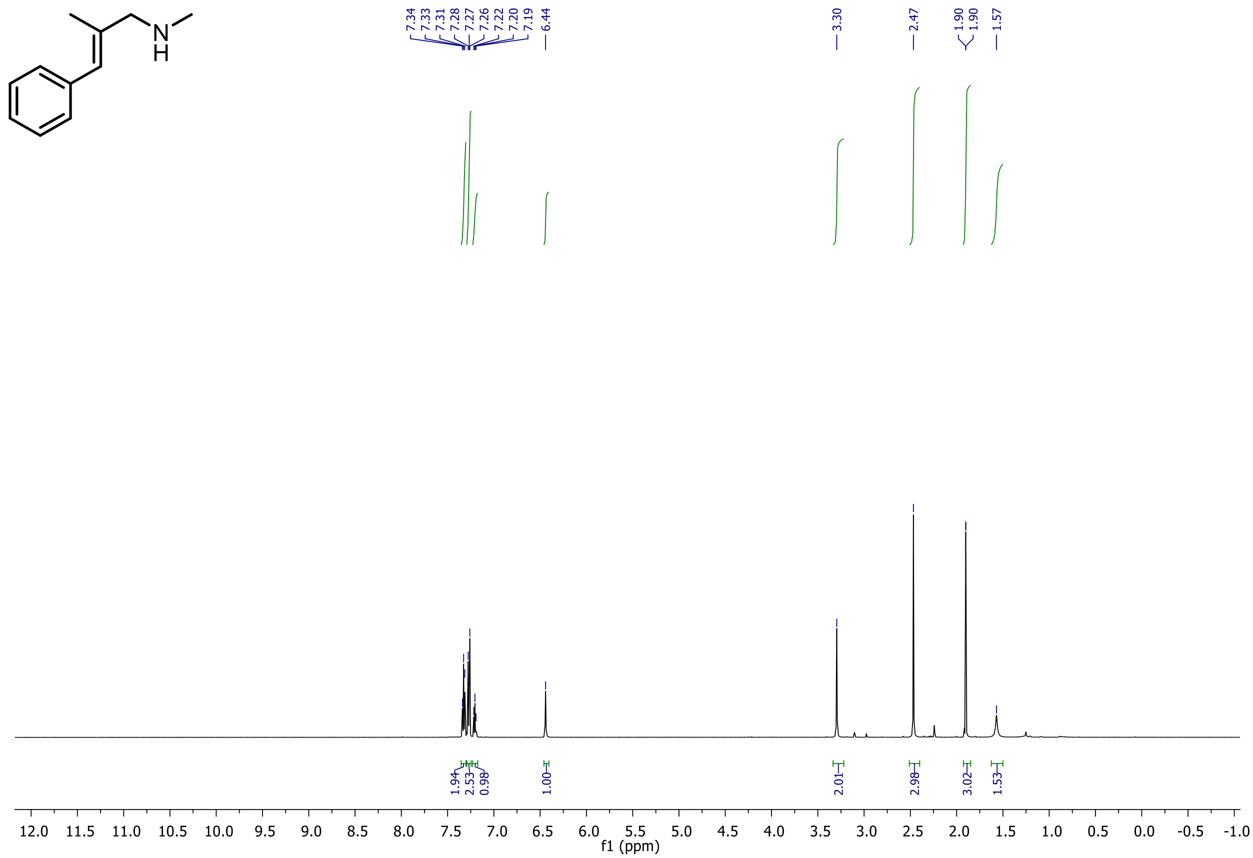
7.34
7.33
7.31
7.28
7.27
7.26
7.22
7.20
7.19
6.44
3.30
2.47
1.90
1.90
1.57
1.94
2.53
0.98
1.00
2.01
2.98
3.02
1.53
f1 (ppm)

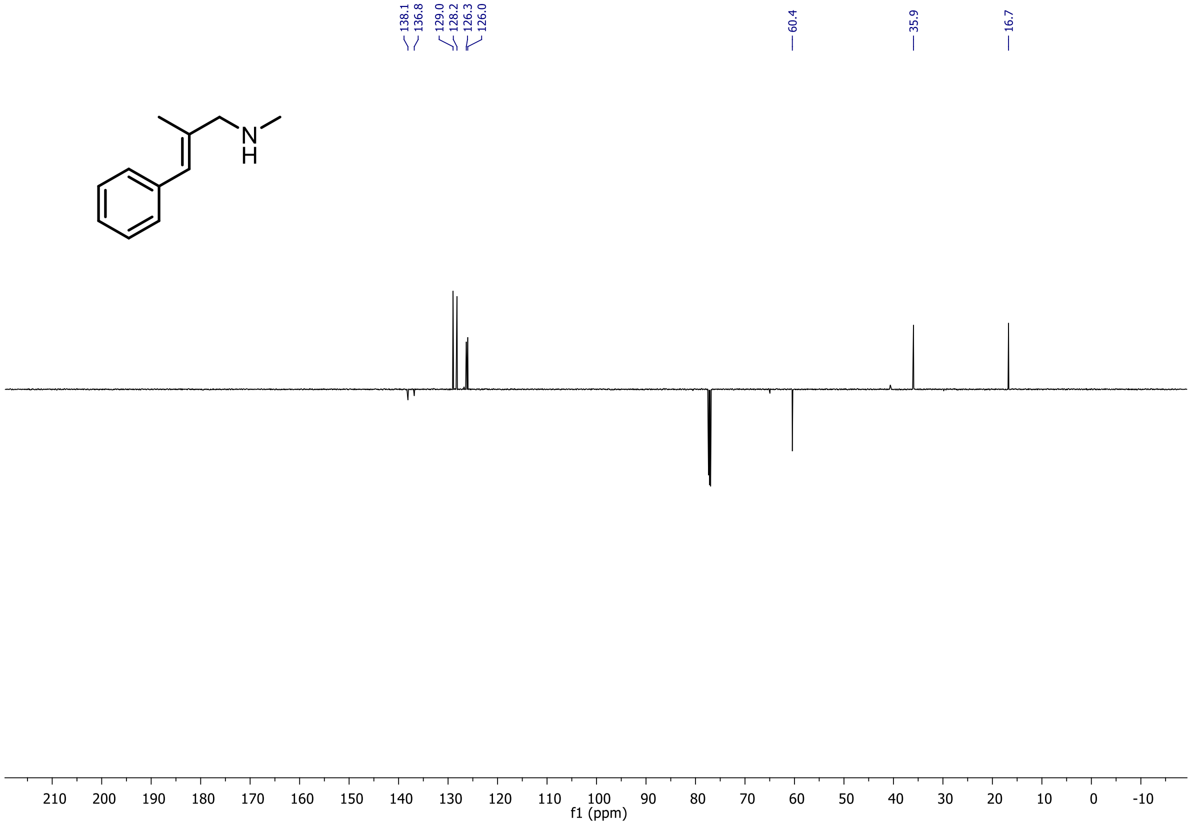
138.1
136.8
129.0
128.2
126.3
126.0
60.4
35.9
16.7
f1 (ppm)

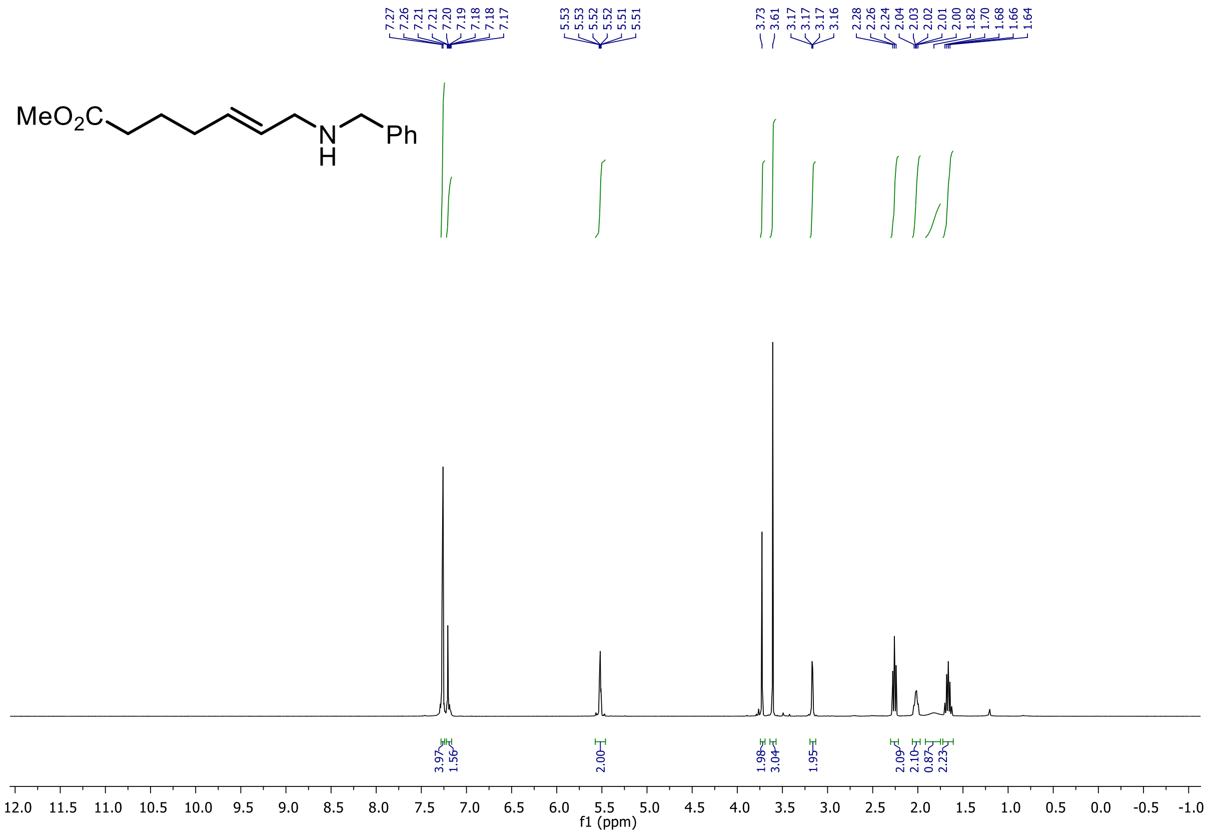
MeO2C
N
H
Ph
f1 (ppm)

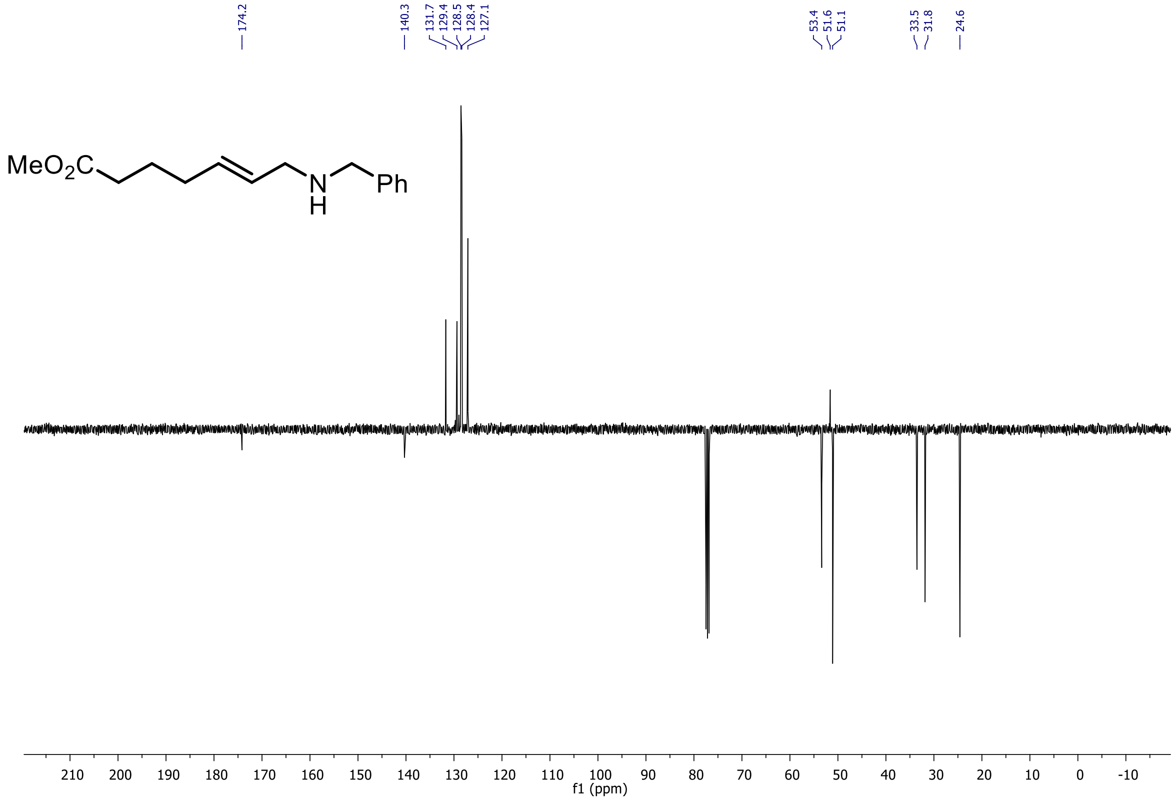
MeO2C
N
H
Ph
f1 (ppm)

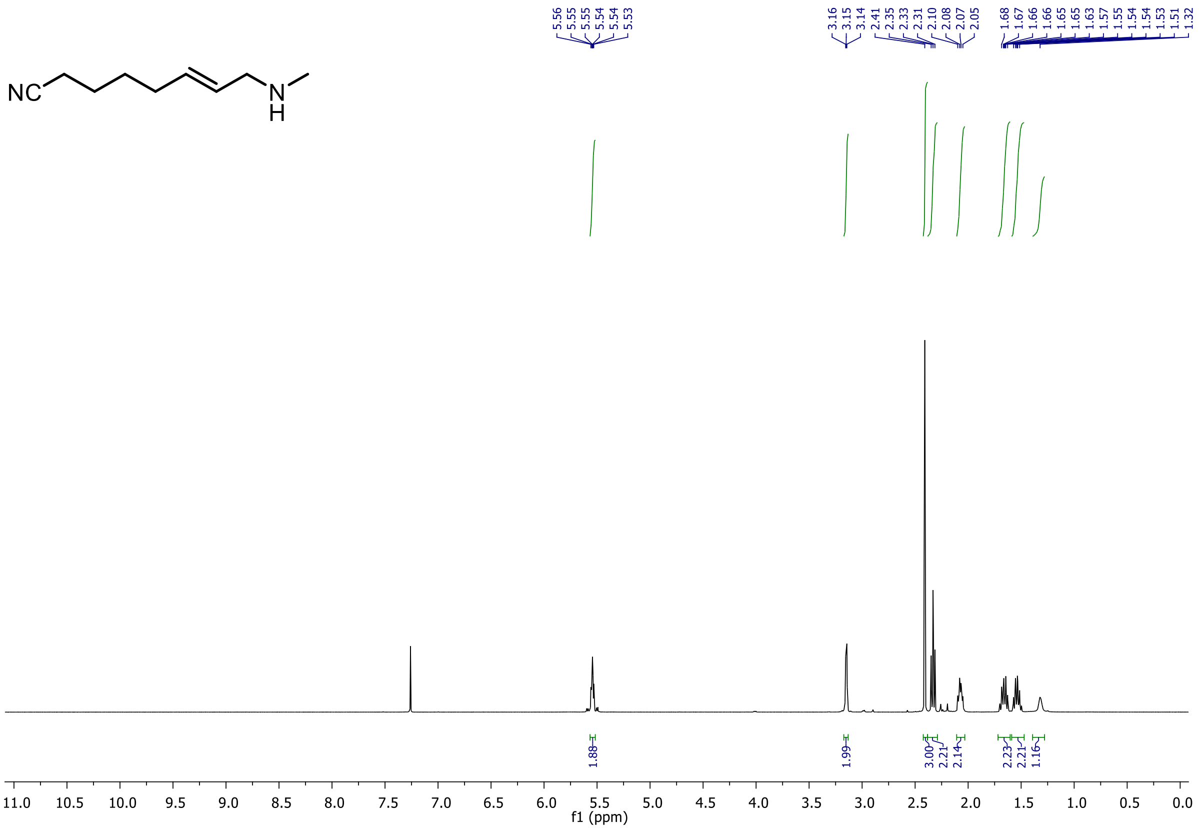

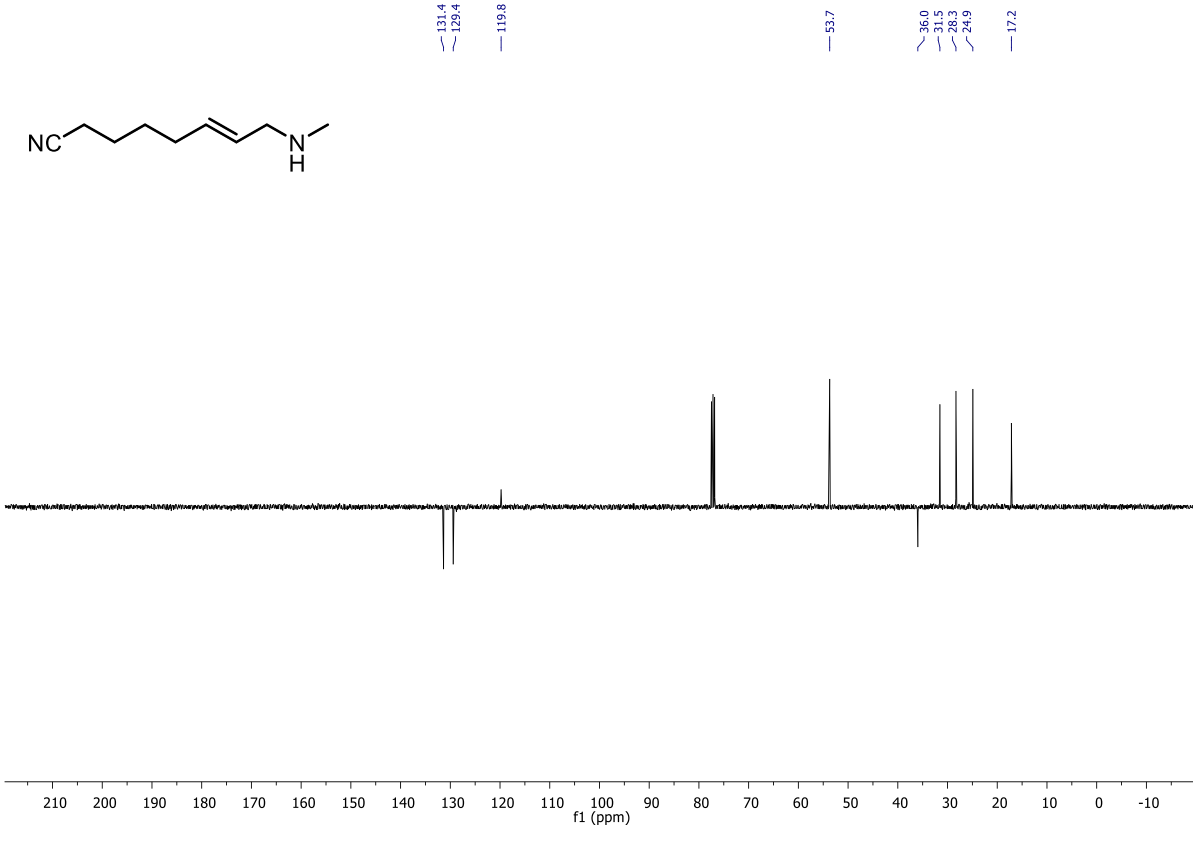

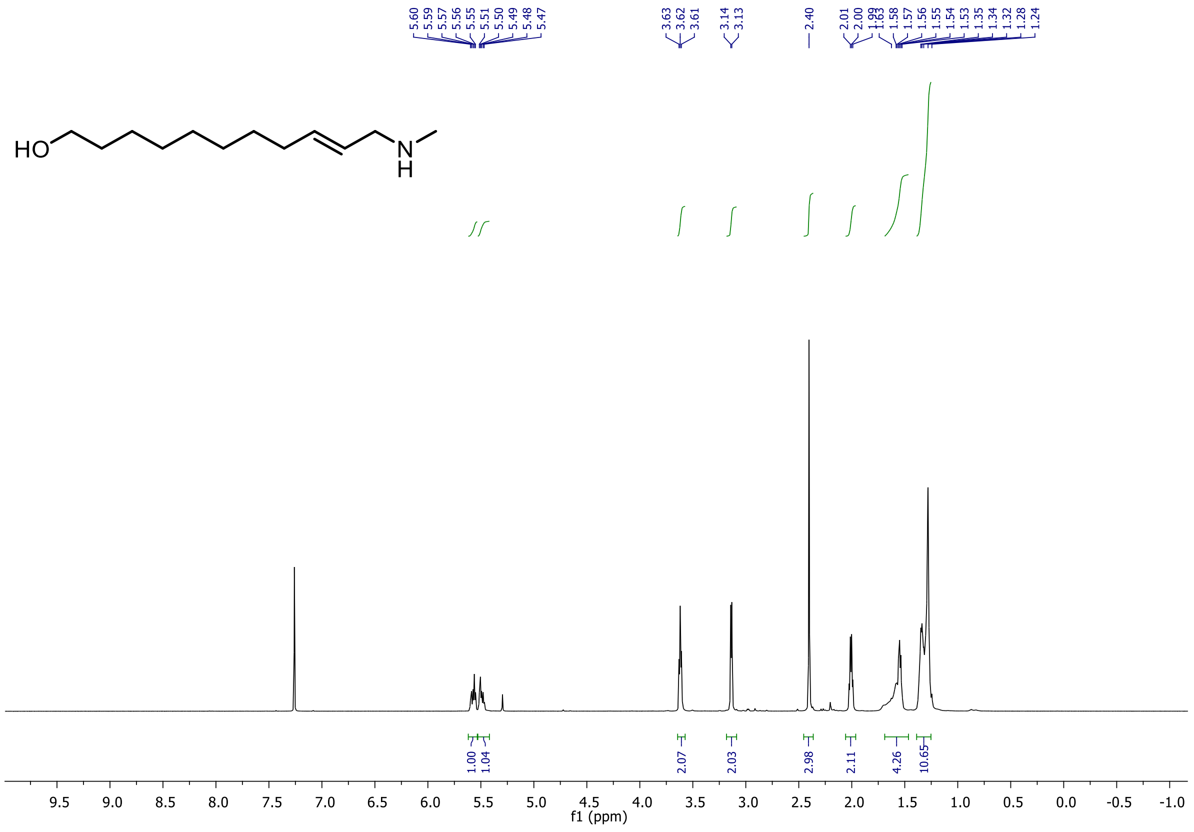

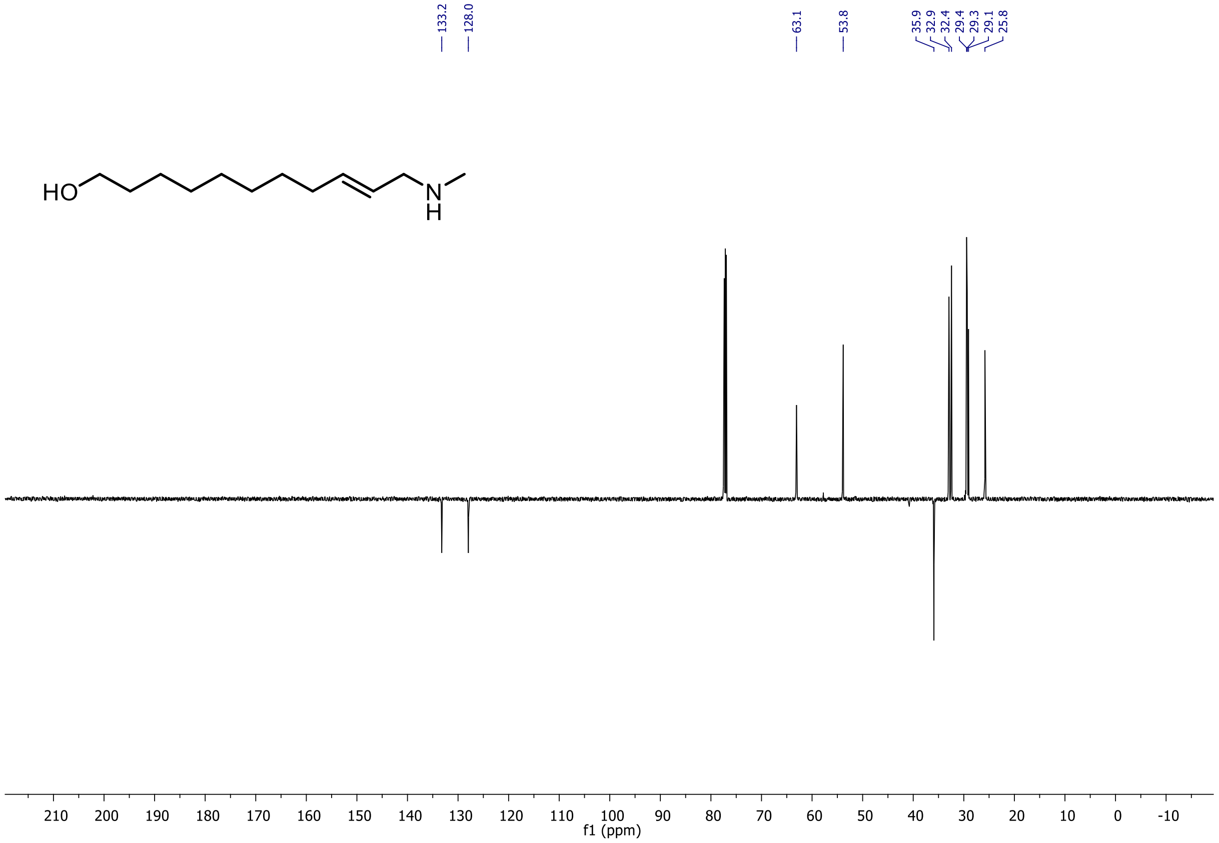

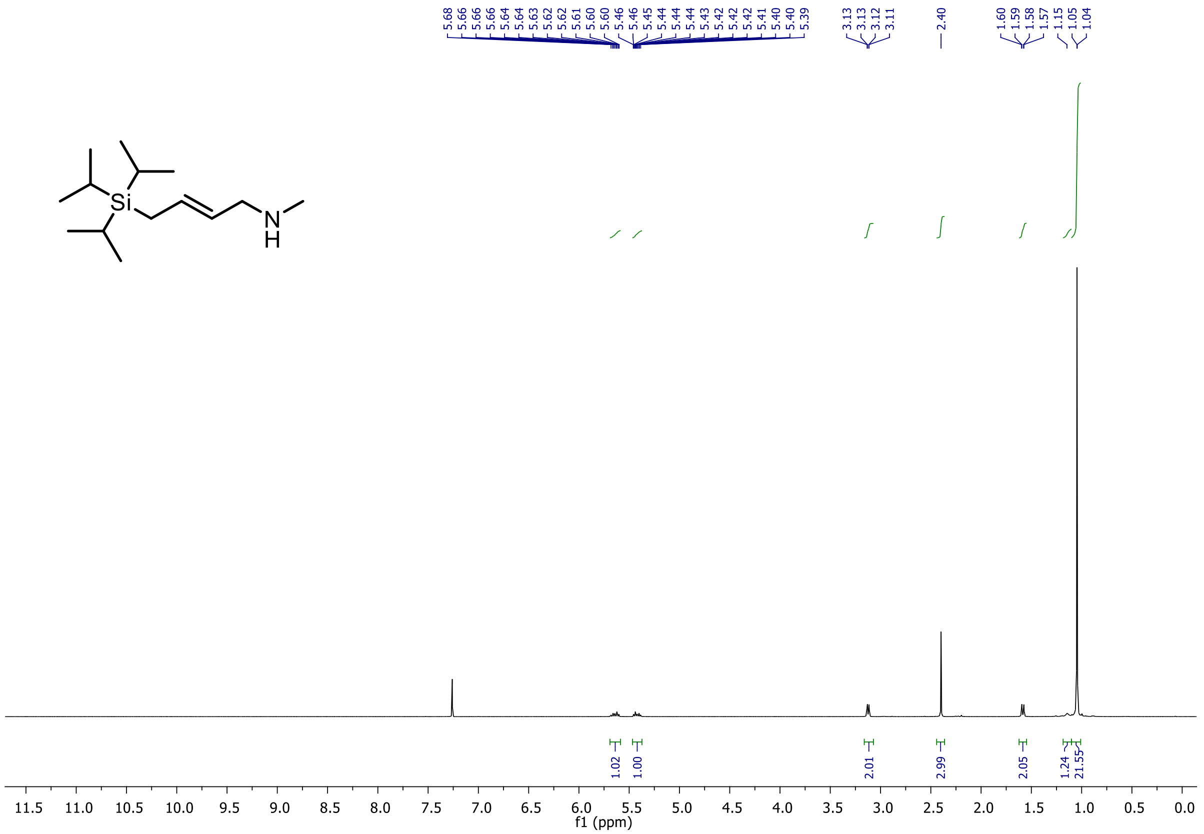
Si
N
H
f1 (ppm)

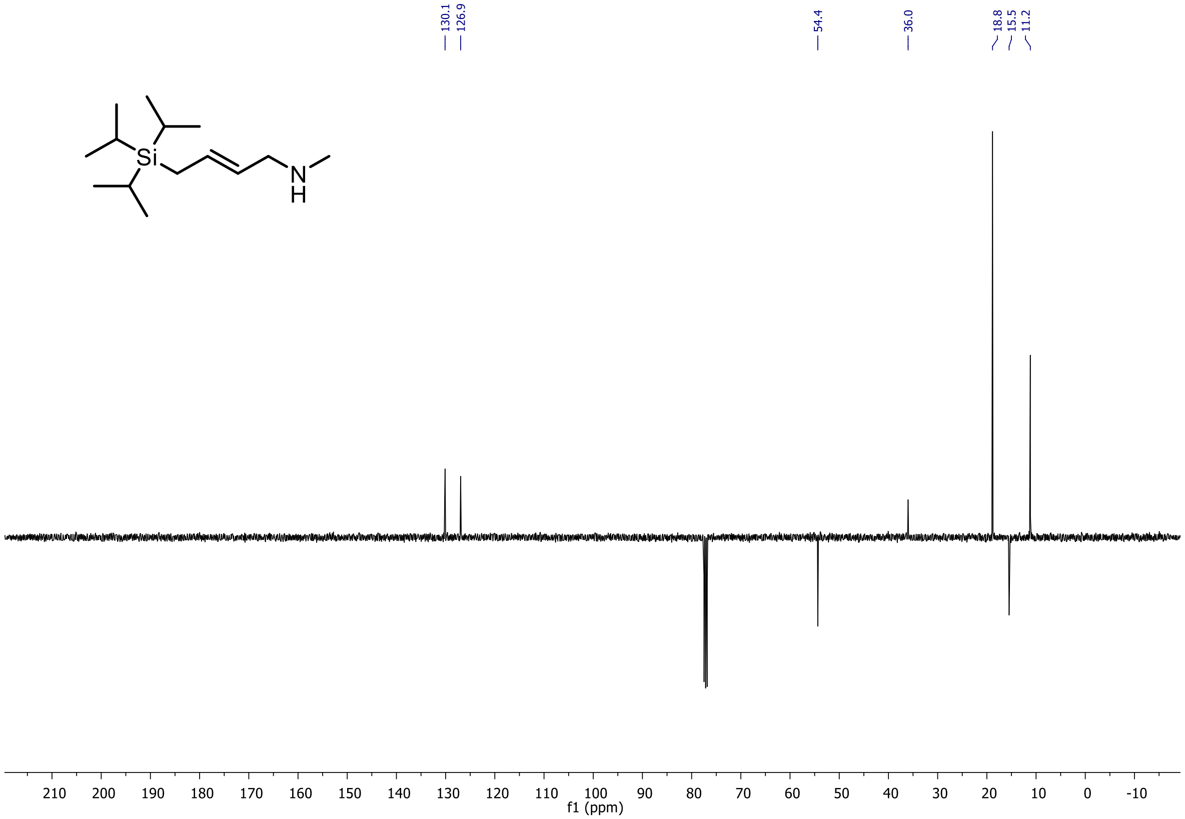
Si
N
H
f1 (ppm)

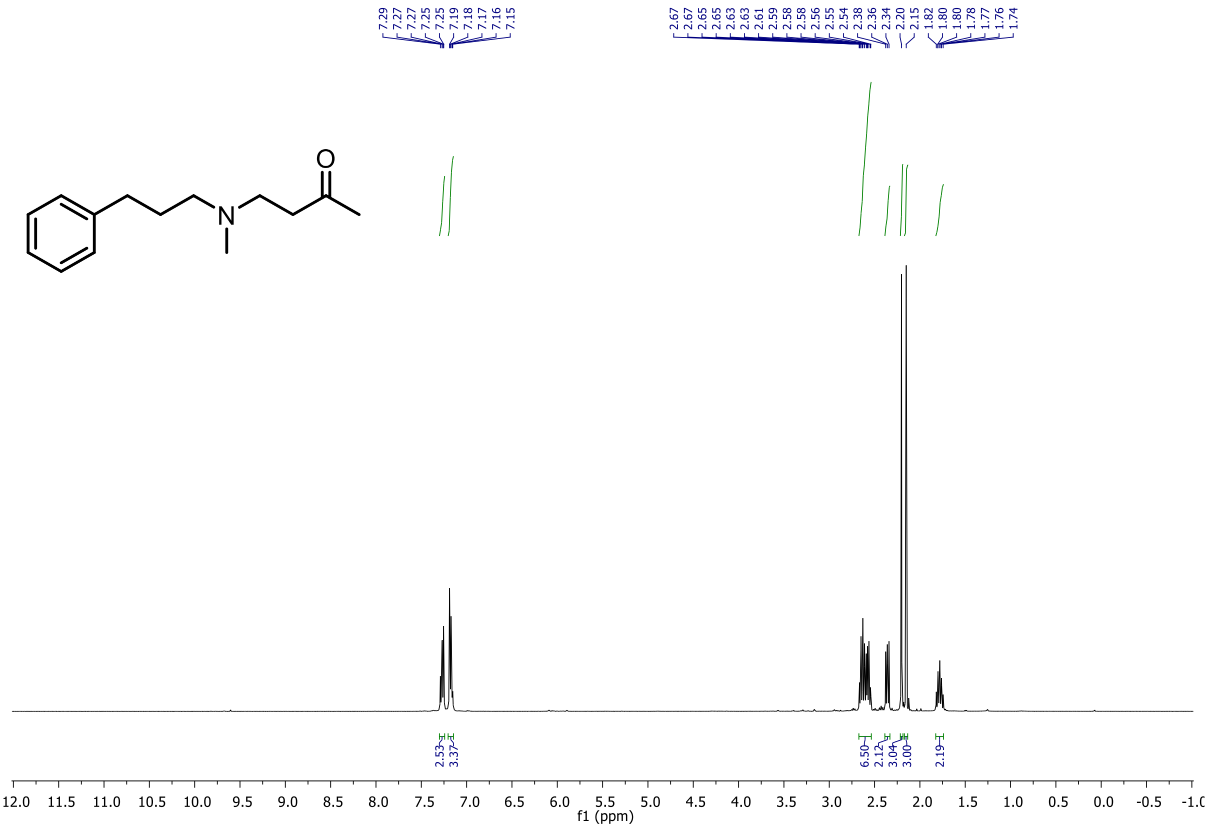

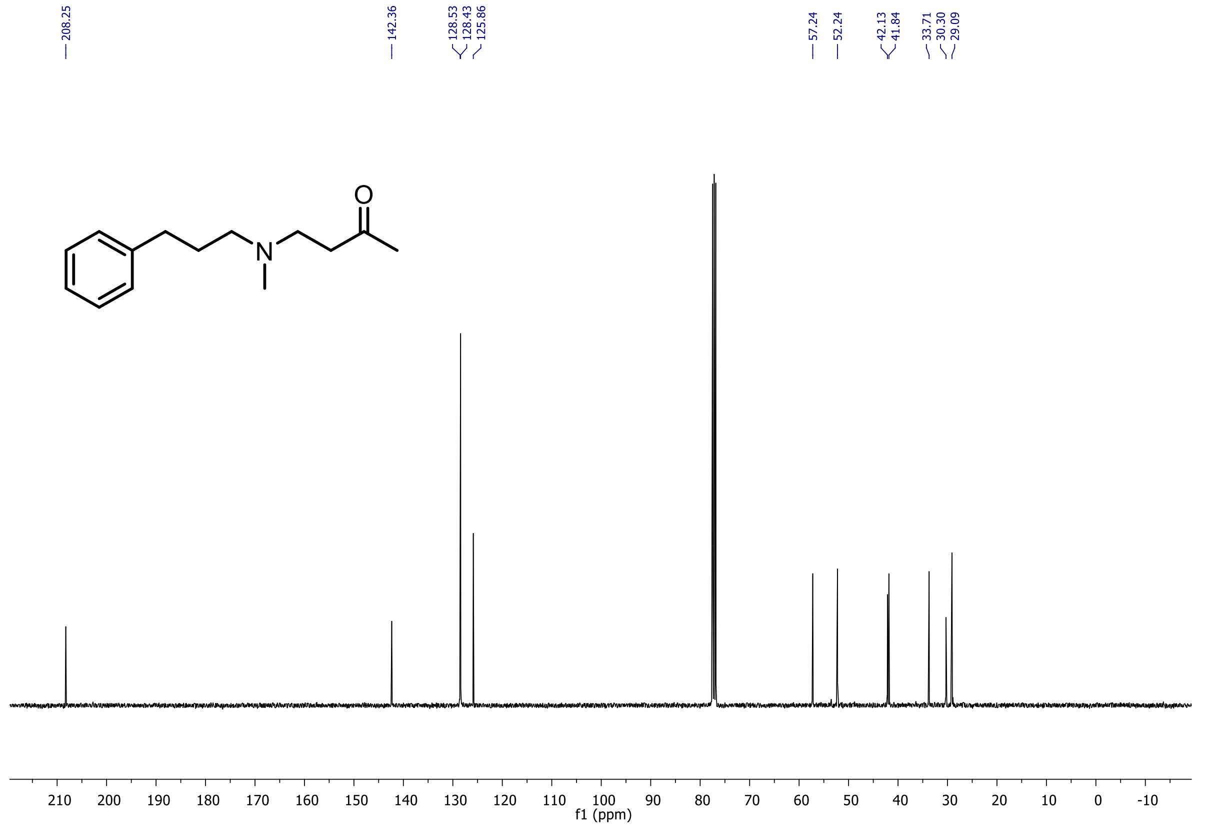

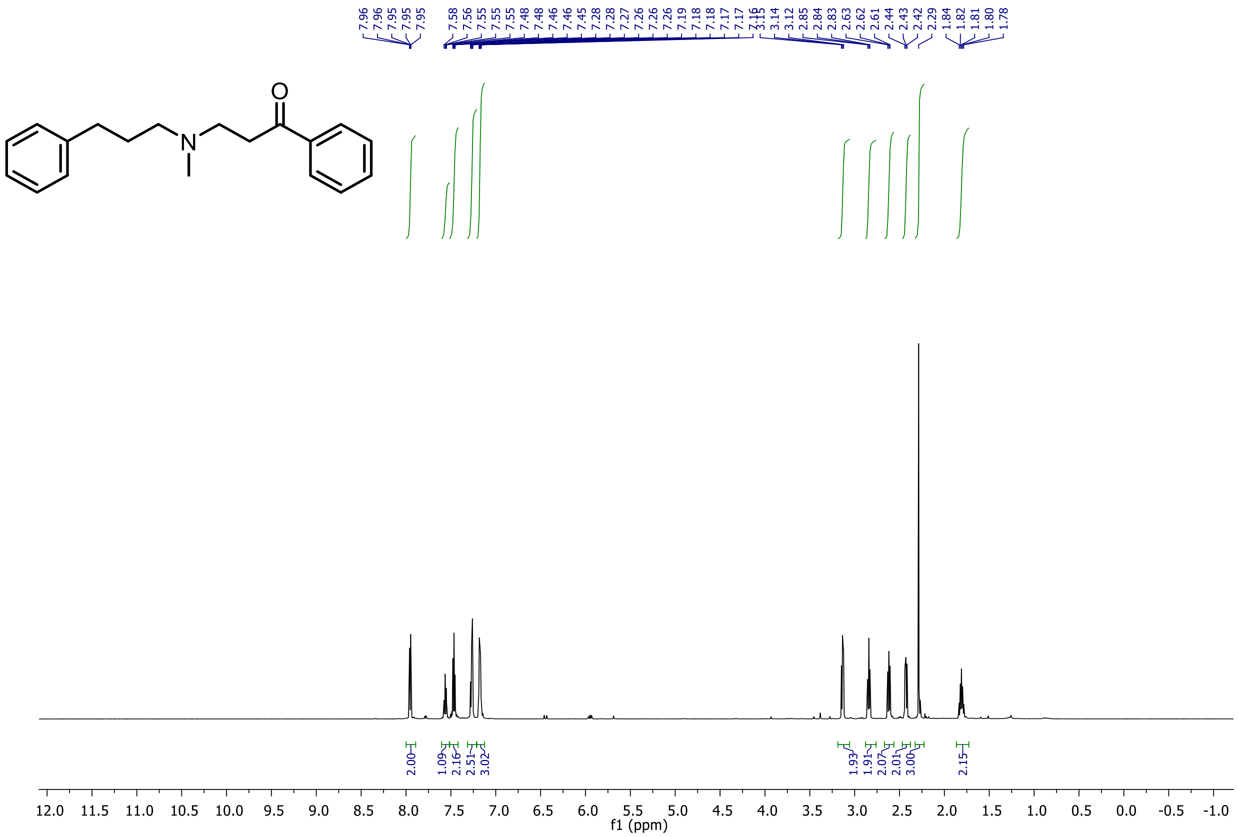
7.96 7.96 7.95 7.95 7.95 7.58 7.56 7.55 7.55 7.55 7.48 7.48 7.46 7.46 7.45 7.28 7.28 7.27 7.26 7.26 7.26 7.19 7.18 7.18 7.17 7.17 7.16 3.15 3.14 3.12 2.85 2.84 2.83 2.63 2.62 2.61 2.44 2.43 2.42 2.29 1.84 1.82 1.81 1.80 1.78
O
N
2.00 1.09 2.16 2.51 3.02 1.93 1.91 2.07 2.01 3.00 2.15
f1 (ppm)

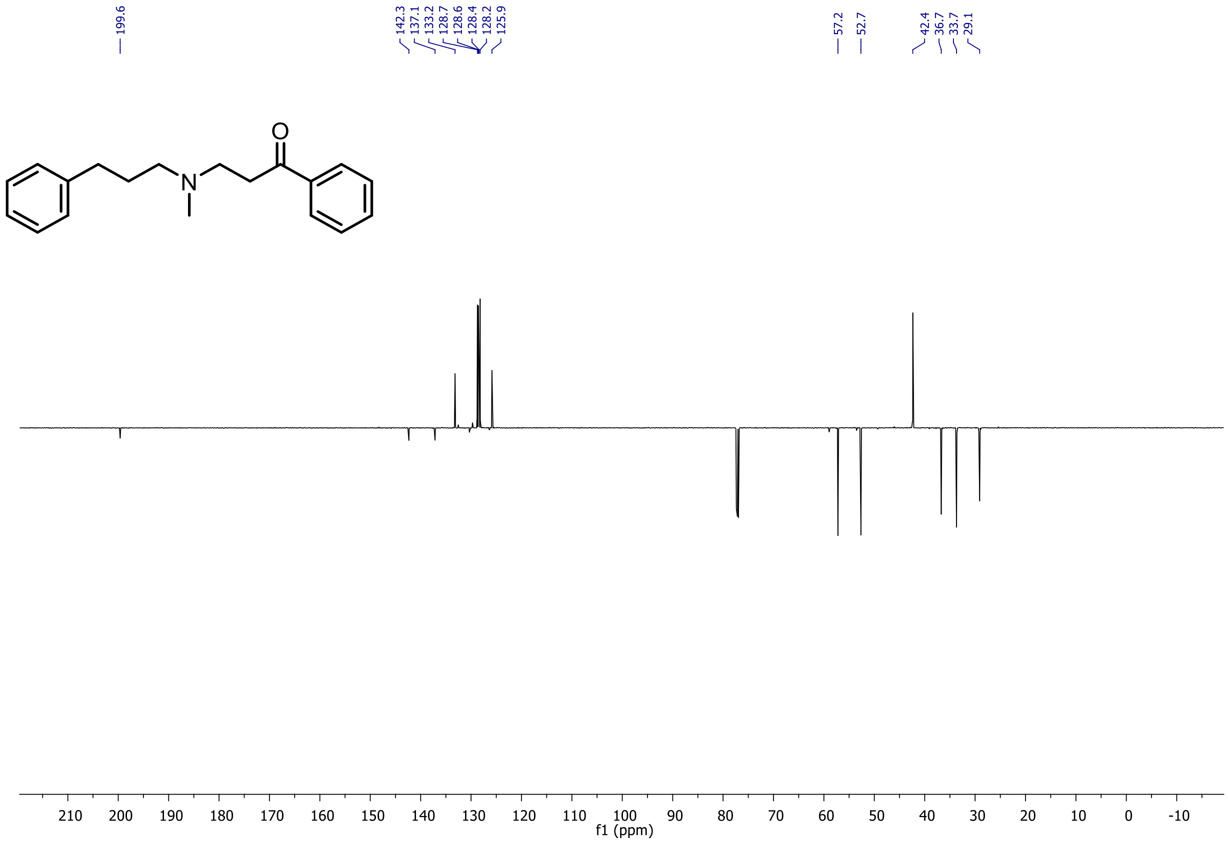
199.6 142.3 137.1 133.2 128.7 128.6 128.4 128.2 125.9 57.2 52.7 42.4 36.7 33.7 29.1
O
N
f1 (ppm)

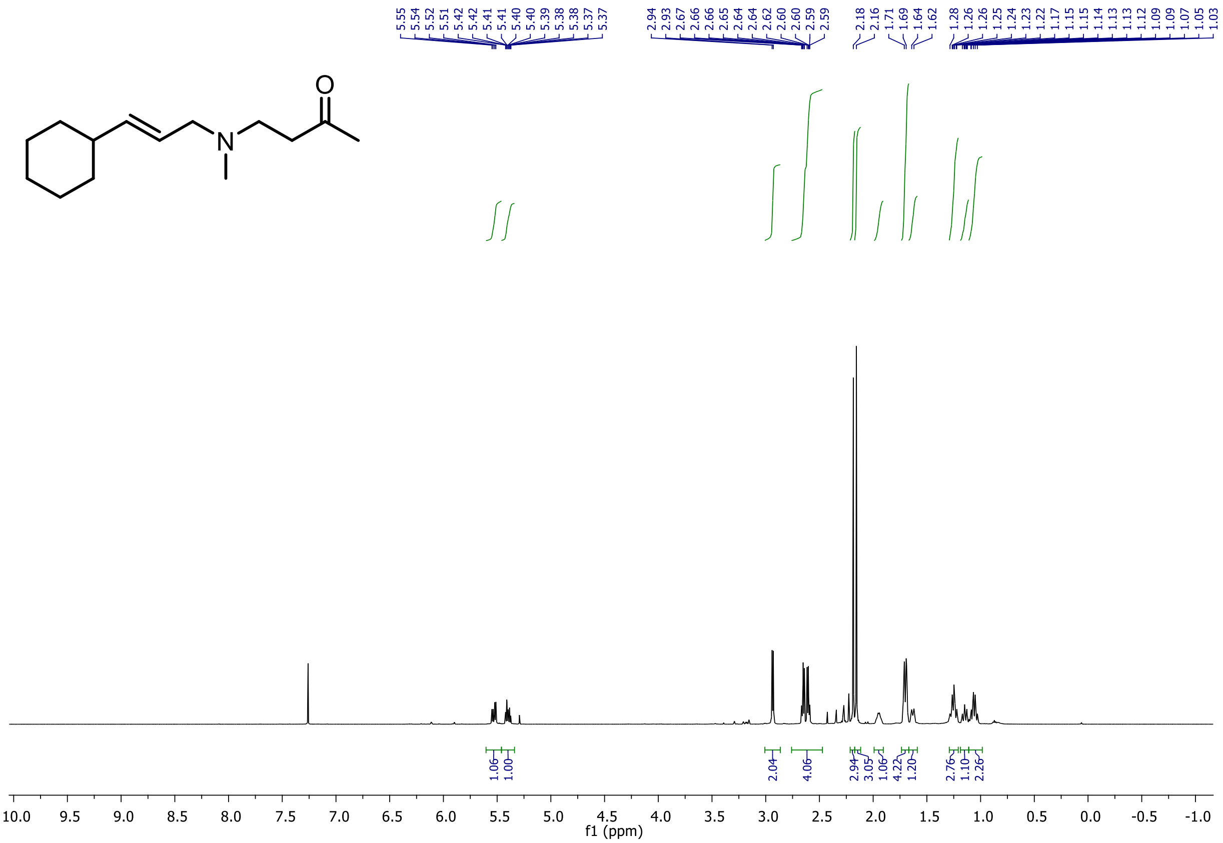

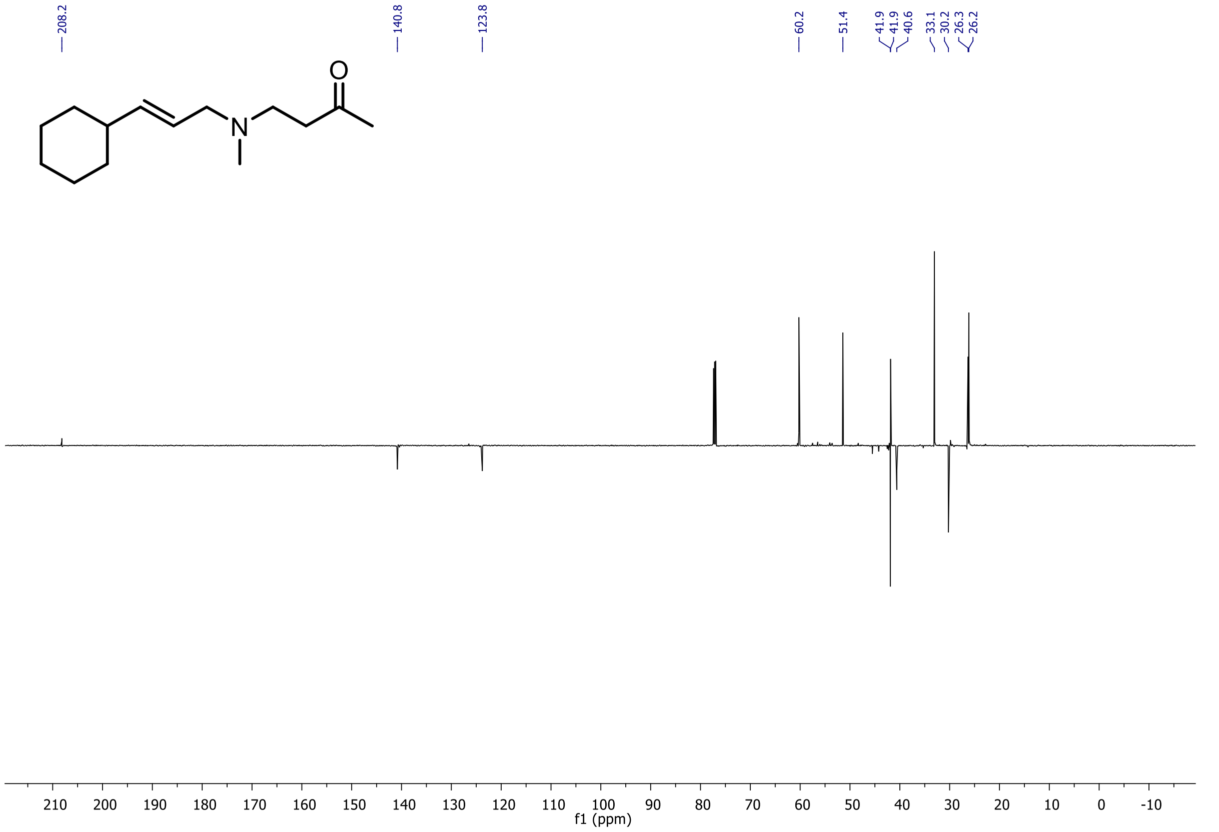

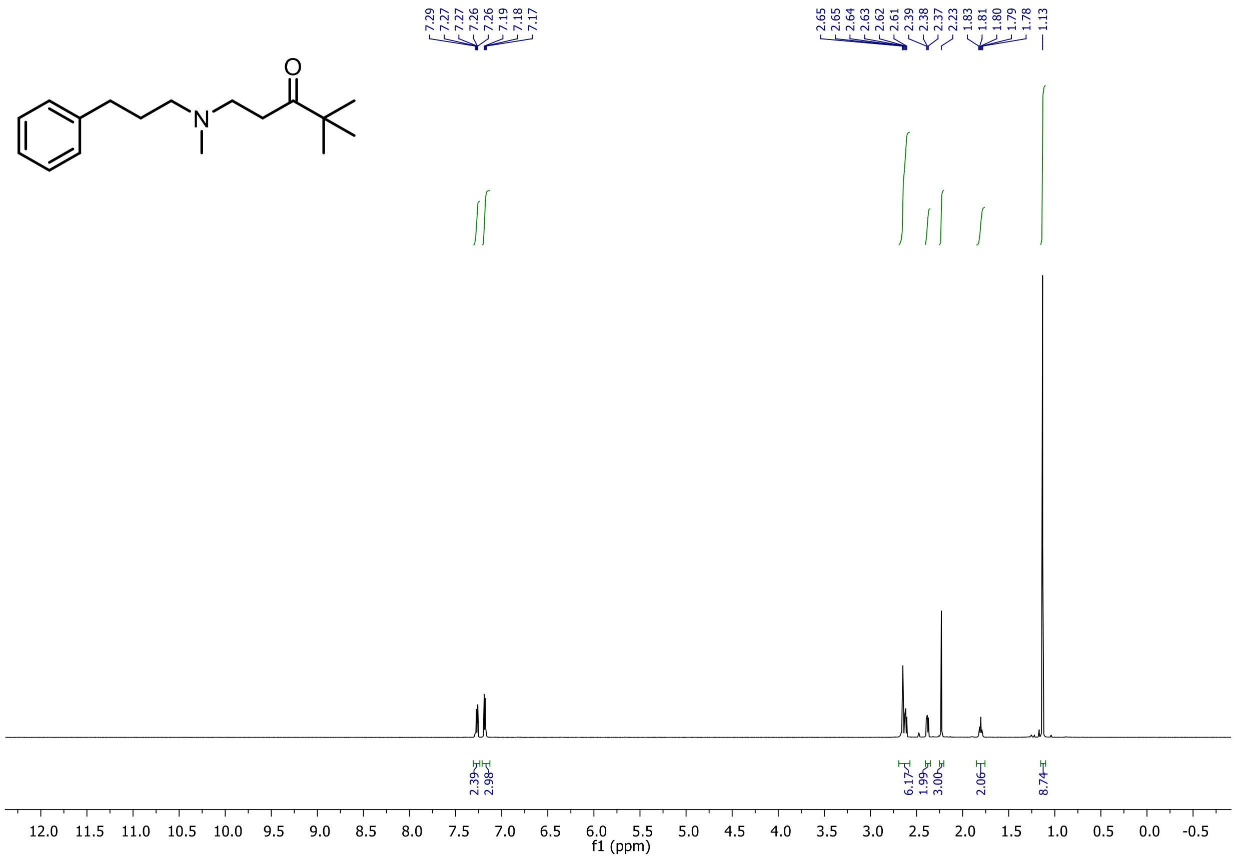
7.29
7.27
7.27
7.26
7.26
7.19
7.18
7.17
2.65
2.65
2.64
2.63
2.62
2.61
2.39
2.38
2.37
2.23
1.83
1.81
1.80
1.79
1.78
1.13
O
N
2.39
2.98
6.17
1.99
3.00
2.06
8.74
12.0 11.5 11.0 10.5 10.0 9.5 9.0 8.5 8.0 7.5 7.0 6.5 6.0 5.5 5.0 4.5 4.0 3.5 3.0 2.5 2.0 1.5 1.0 0.5 0.0 -0.5
f1 (ppm)

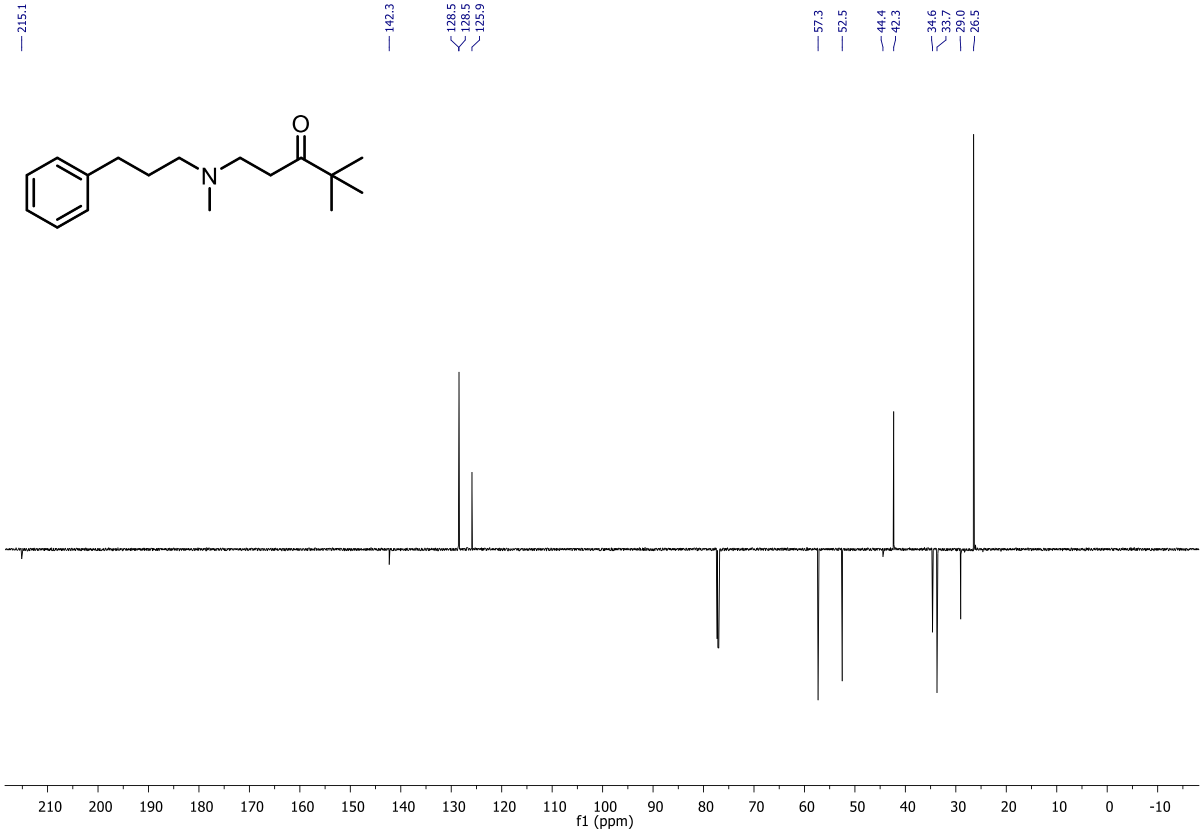
215.1
142.3
128.5
128.5
125.9
57.3
52.5
44.4
42.3
34.6
33.7
29.0
26.5
O
N
210 200 190 180 170 160 150 140 130 120 110 100 90 80 70 60 50 40 30 20 10 0 -10
f1 (ppm)

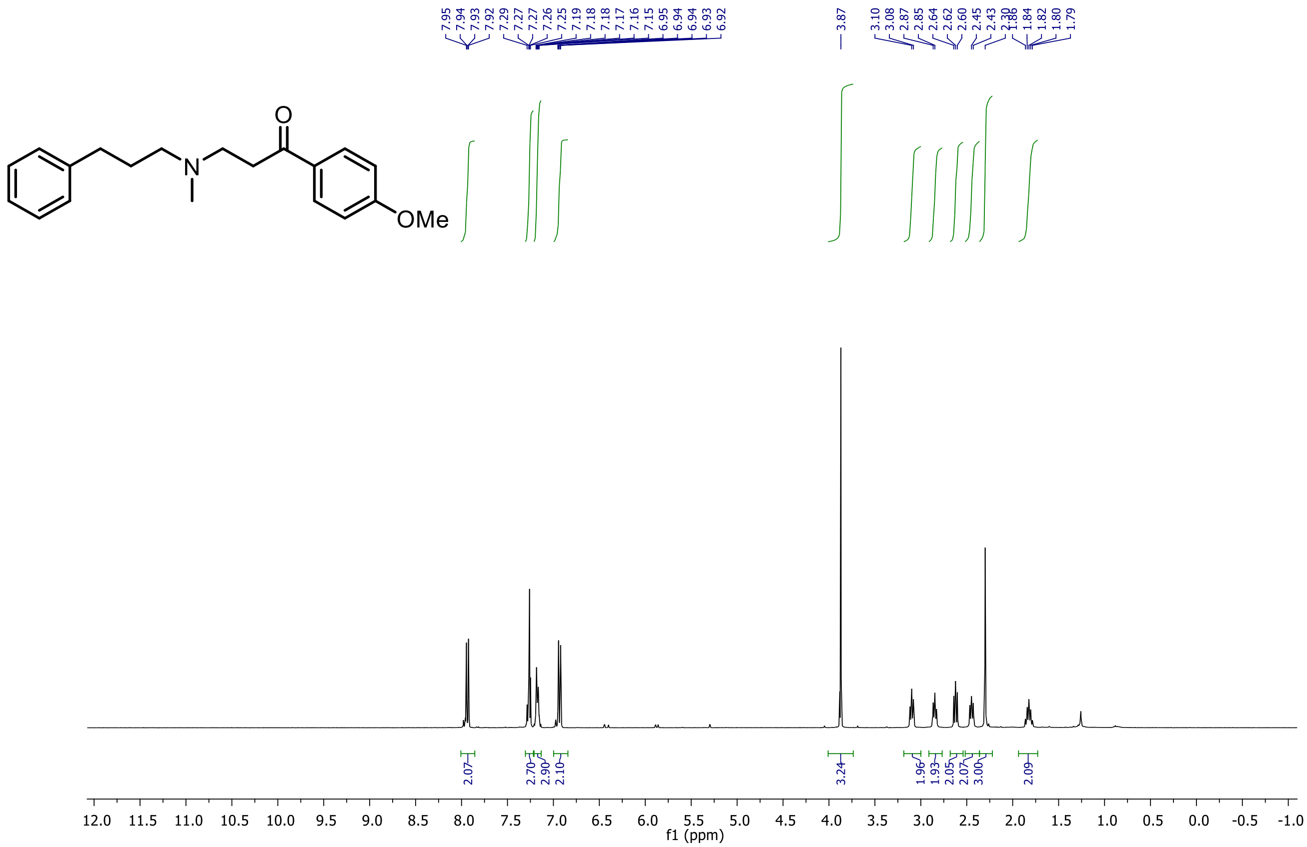
7.95 7.94 7.93 7.92 7.29 7.27 7.27 7.26 7.25 7.19 7.18 7.18 7.17 7.16 7.15 6.95 6.94 6.94 6.93 6.92
3.87
3.10 3.08 2.87 2.85 2.64 2.62 2.60 2.45 2.43 2.30 1.86 1.84 1.82 1.80 1.79
O
N
OMe
2.07
2.70 2.90 2.10
3.24
1.96 1.93 2.05 2.07 3.00
2.09
12.0 11.5 11.0 10.5 10.0 9.5 9.0 8.5 8.0 7.5 7.0 6.5 6.0 5.5 5.0 4.5 4.0 3.5 3.0 2.5 2.0 1.5 1.0 0.5 0.0 -0.5 -1.0
f1 (ppm)

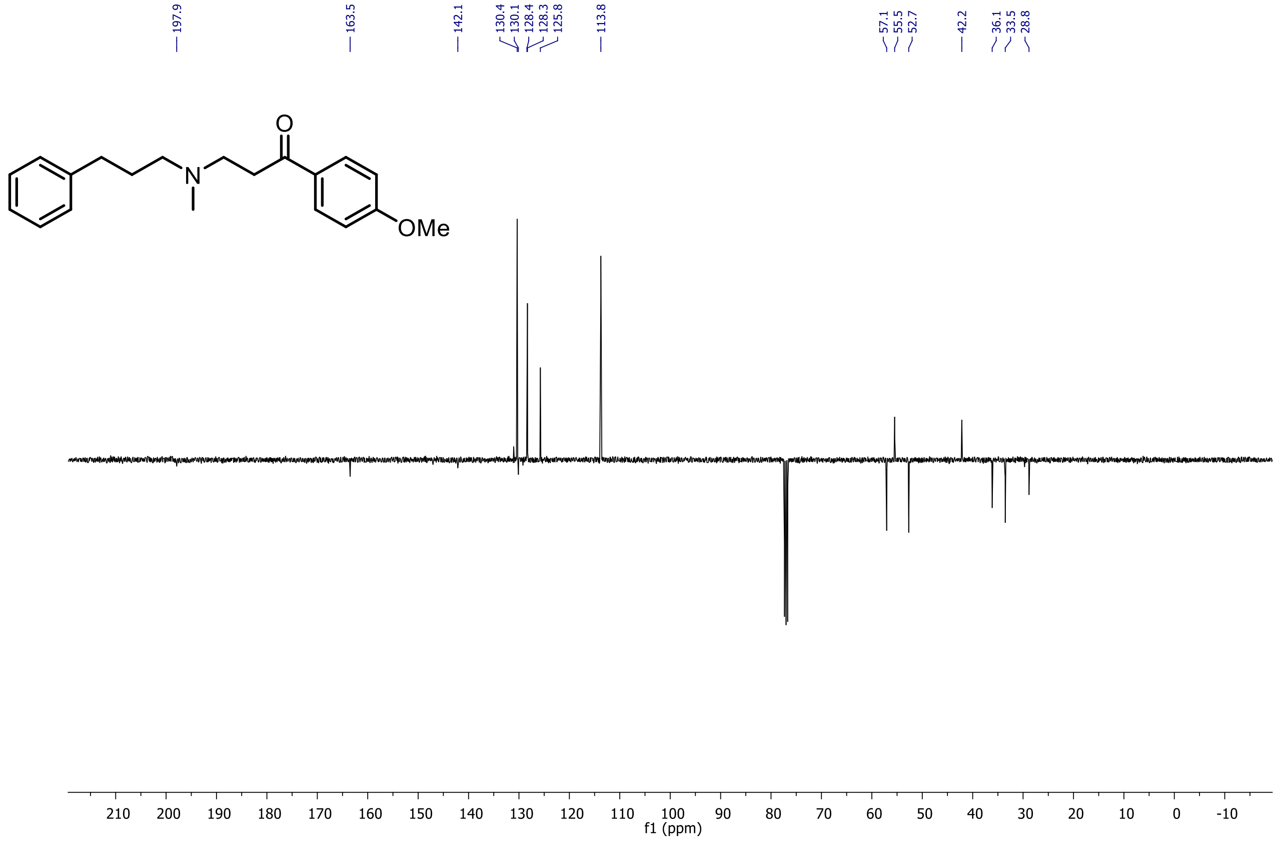
197.9
163.5
142.1
130.4 130.1 128.4 128.3 125.8
113.8
57.1 55.5 52.7
42.2
36.1 33.5 28.8
O
N
OMe
210 200 190 180 170 160 150 140 130 120 110 100 90 80 70 60 50 40 30 20 10 0 -10
f1 (ppm)

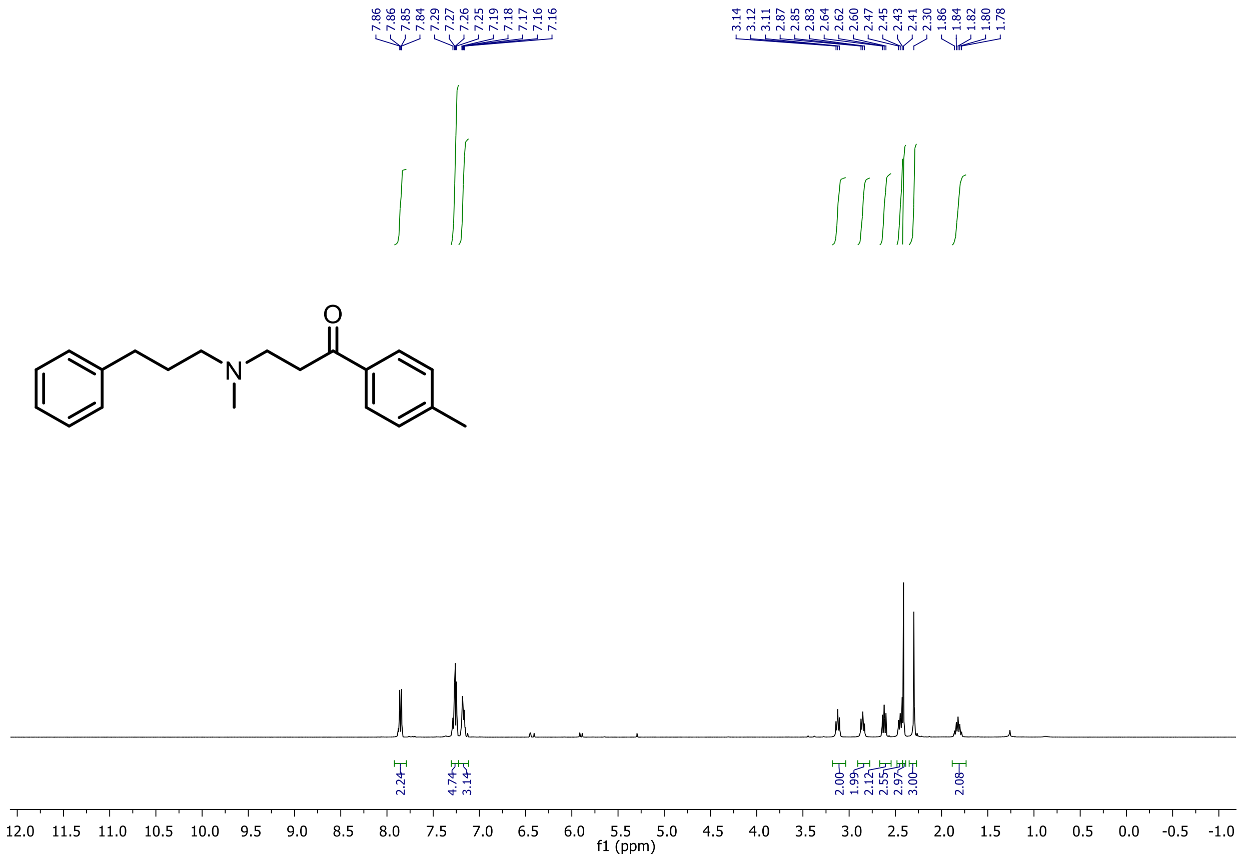

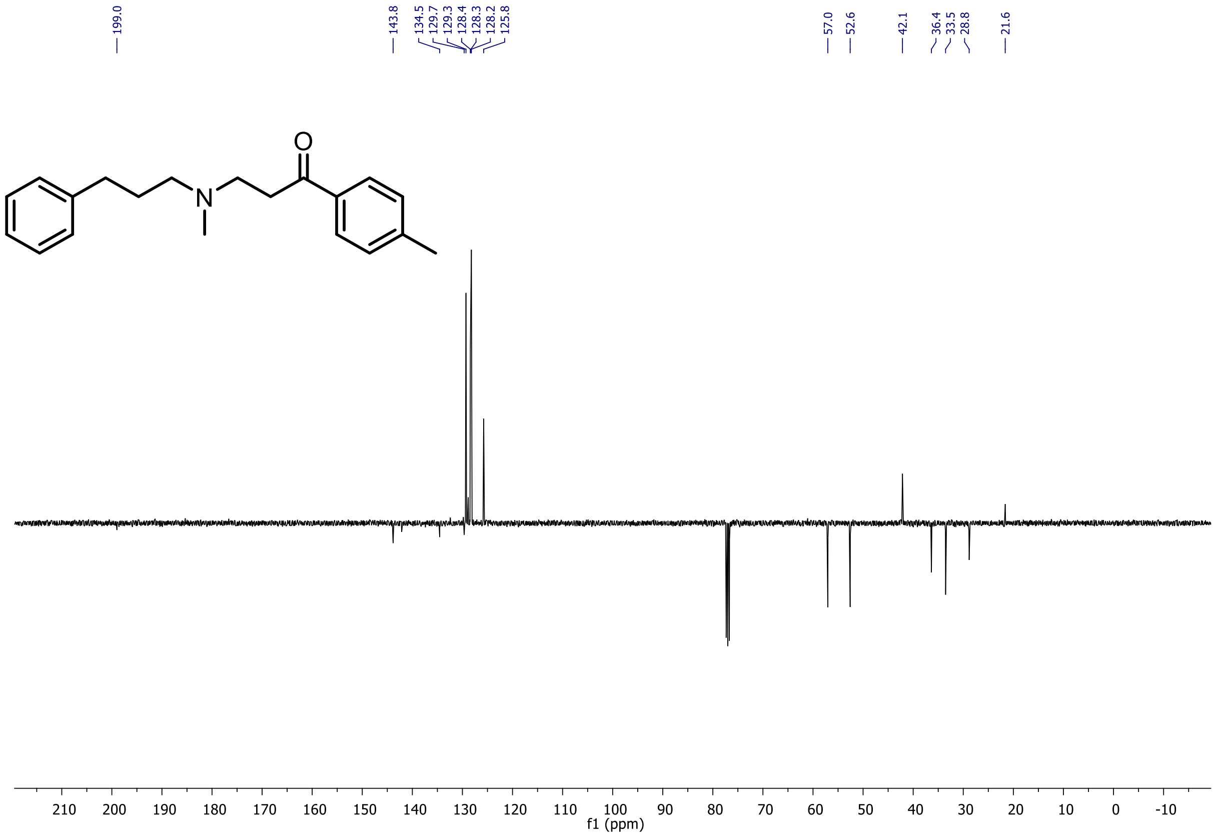

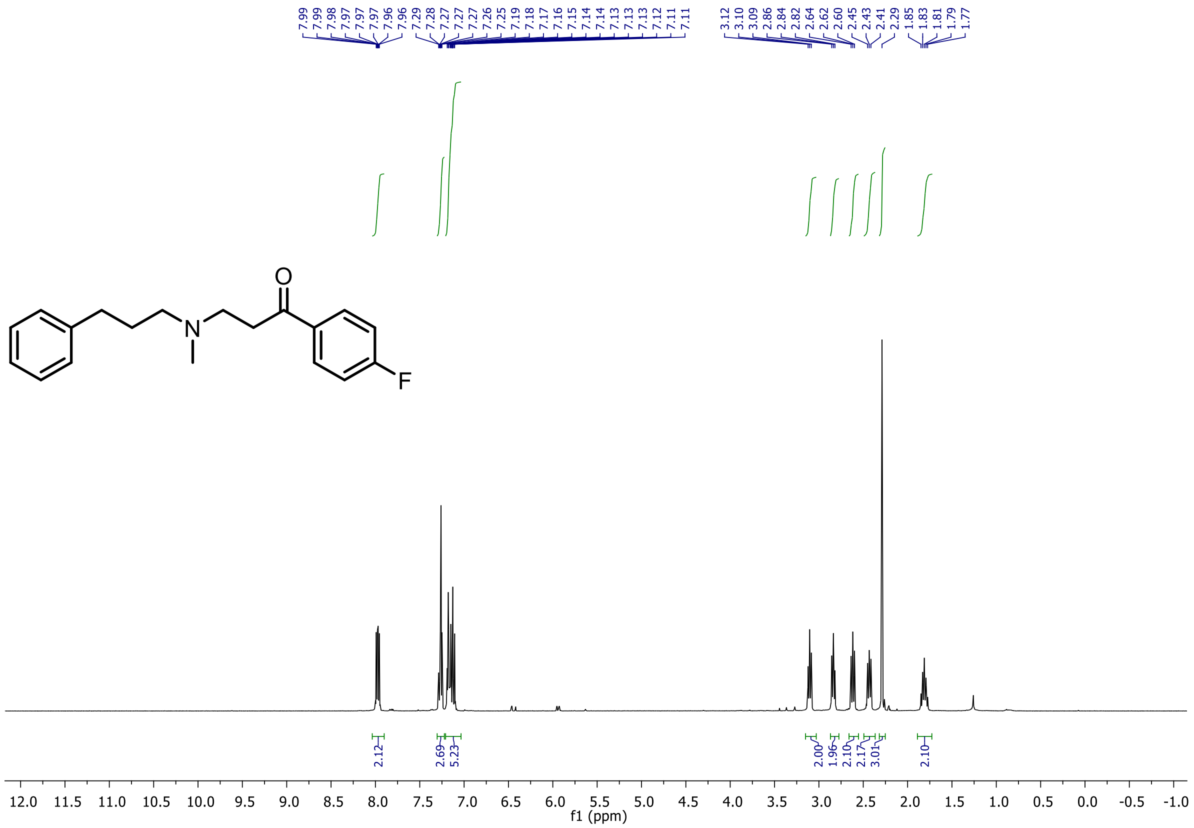
7.99 7.99 7.98 7.97 7.97 7.97 7.96 7.96 7.29 7.28 7.27 7.27 7.27 7.26 7.25 7.19 7.18 7.17 7.16 7.15 7.14 7.14 7.13 7.13 7.13 7.12 7.11 7.11
3.12 3.10 3.09 2.86 2.84 2.82 2.64 2.62 2.60 2.45 2.43 2.41 2.29 1.85 1.83 1.81 1.79 1.77
O
N
F
2.12 2.69 5.23 2.00 1.96 2.10 2.17 3.01 2.10
f1 (ppm)

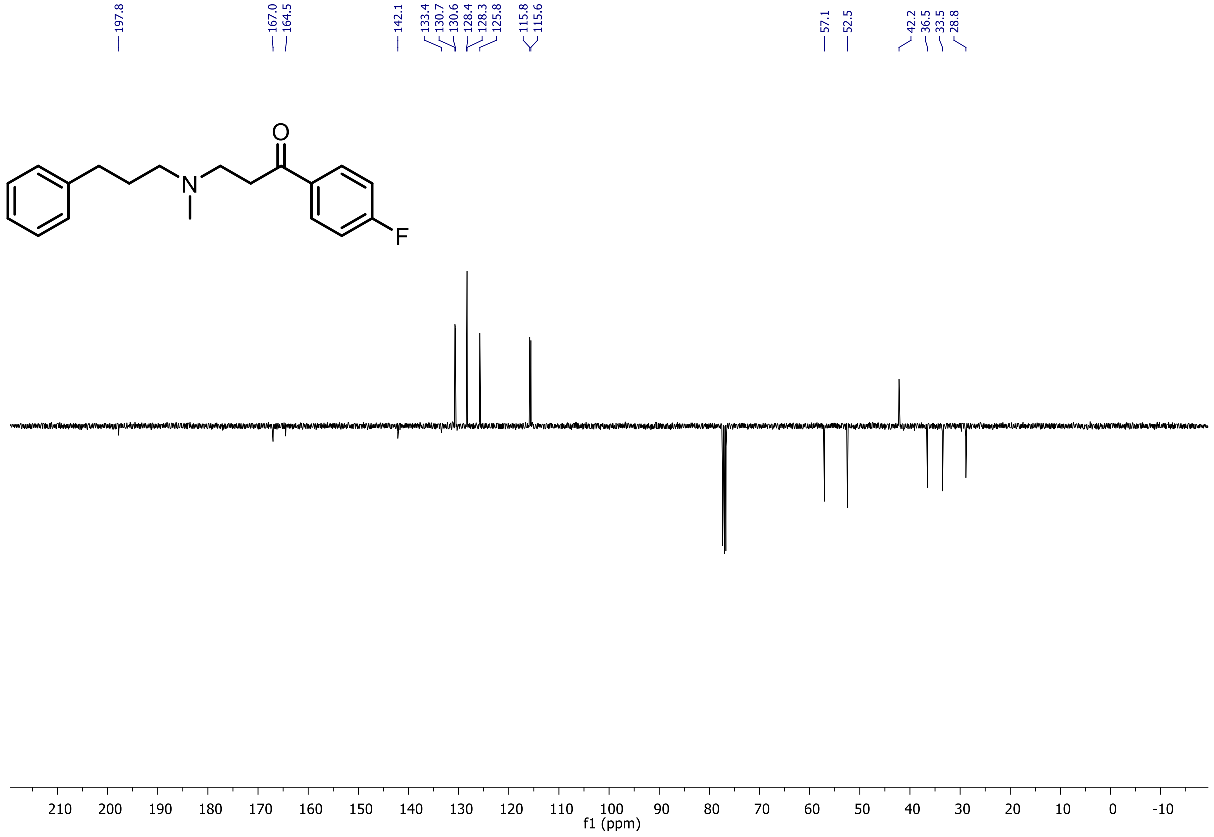
197.8 167.0 164.5 142.1 133.4 130.7 130.6 128.4 128.3 125.8 115.8 115.6 57.1 52.5 42.2 36.5 33.5 28.8
O
N
F
f1 (ppm)

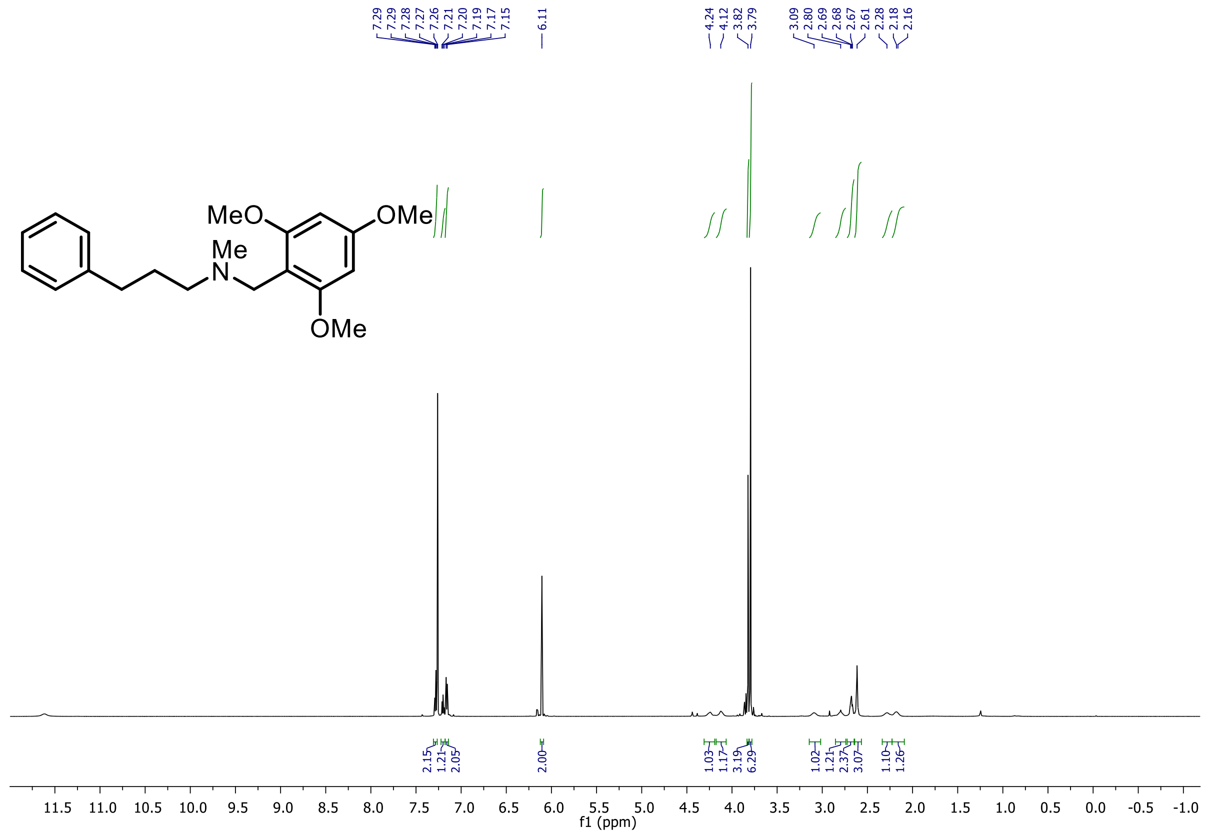

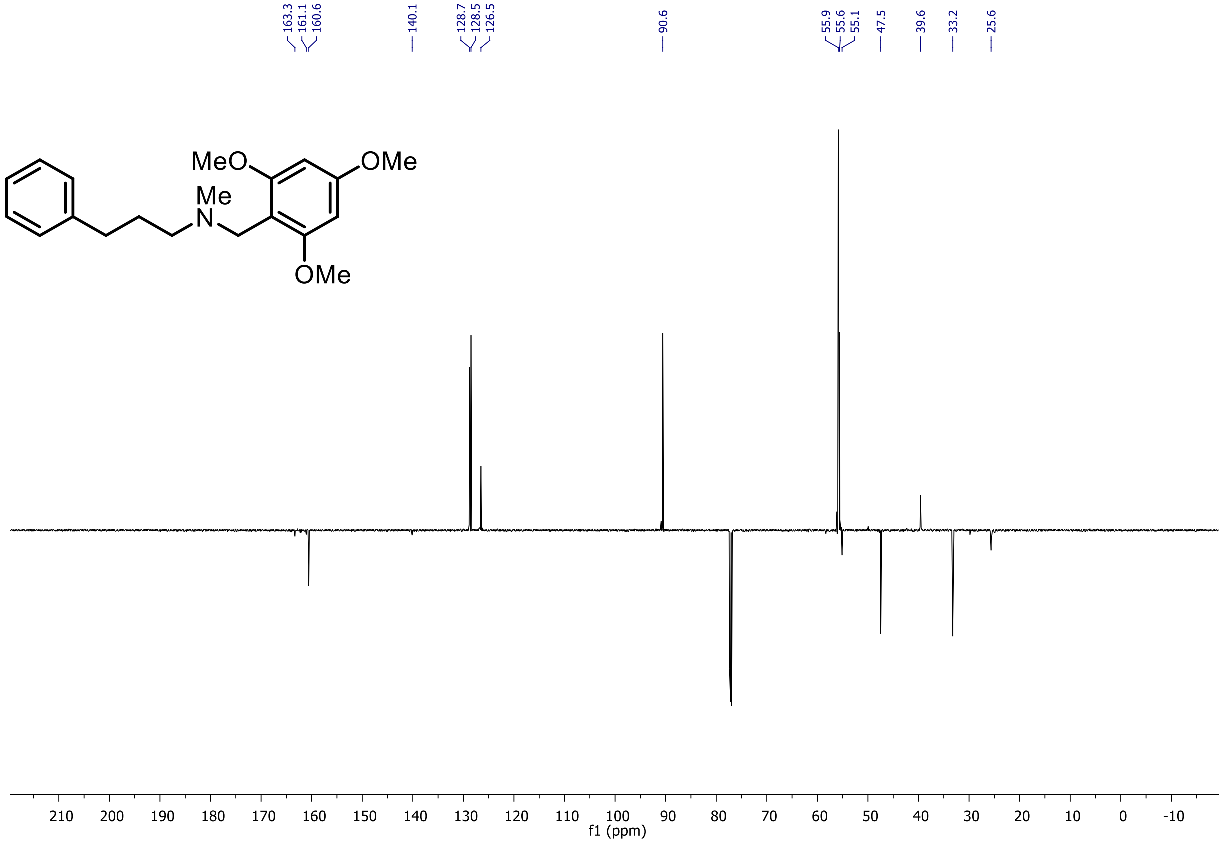

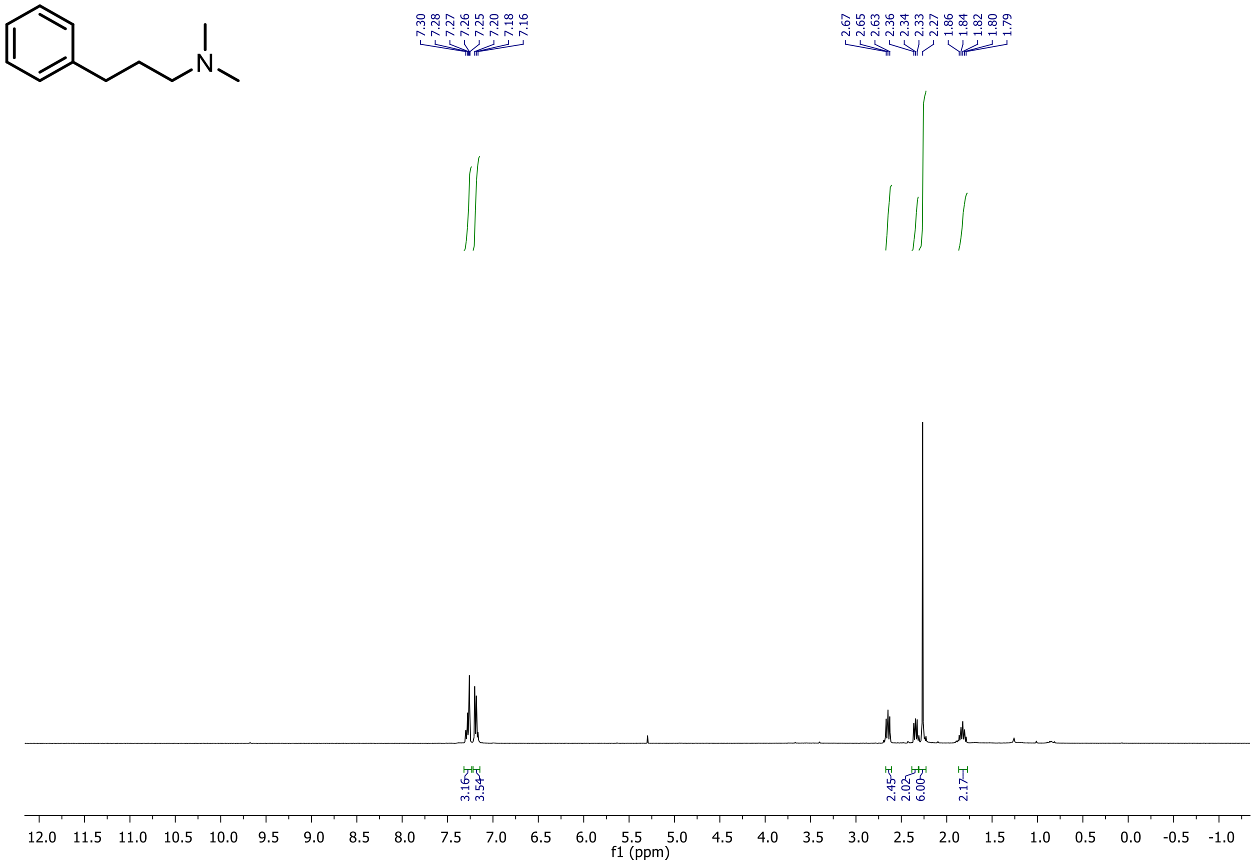
7.30
7.28
7.27
7.26
7.25
7.20
7.18
7.16
2.67
2.65
2.63
2.36
2.34
2.33
2.27
1.86
1.84
1.82
1.80
1.79
3.16
3.54
2.45
2.02
6.00
2.17
12.0 11.5 11.0 10.5 10.0 9.5 9.0 8.5 8.0 7.5 7.0 6.5 6.0 5.5 5.0 4.5 4.0 3.5 3.0 2.5 2.0 1.5 1.0 0.5 0.0 -0.5 -1.0
f1 (ppm)

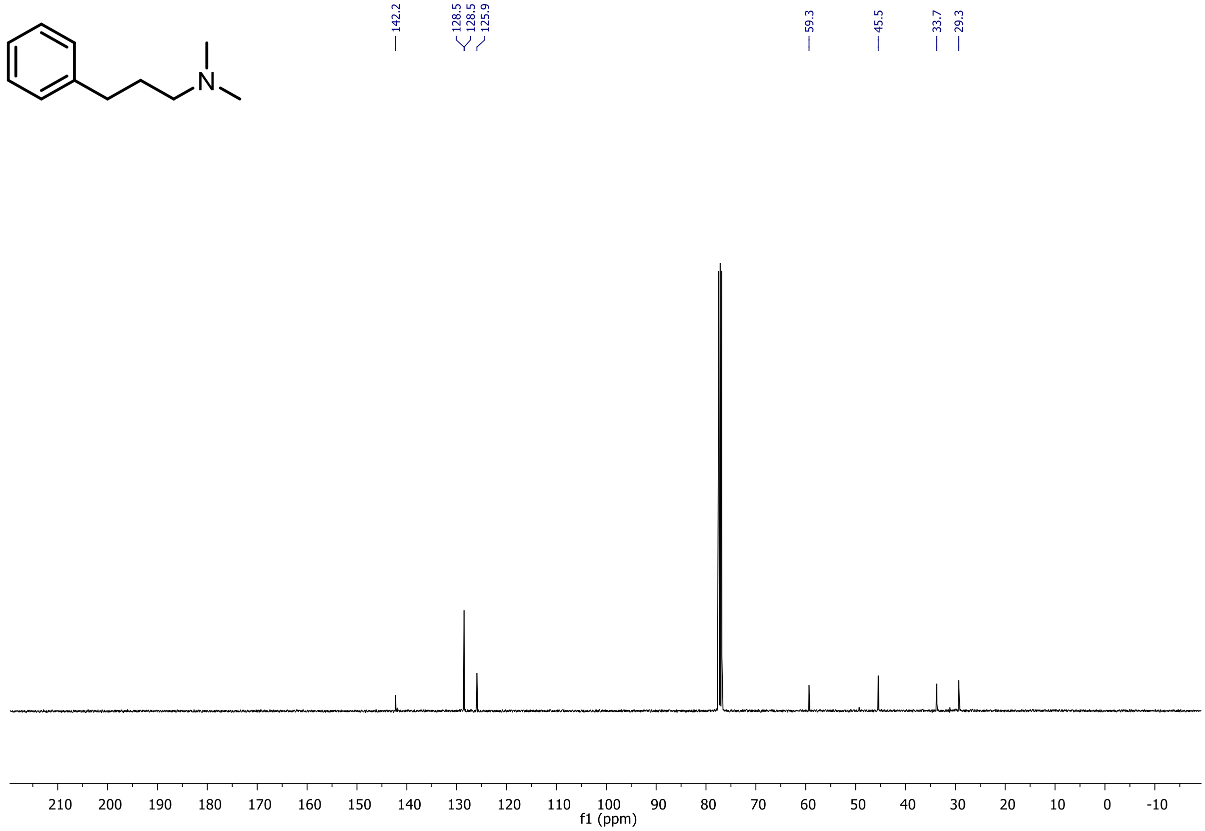
142.2
128.5
128.5
125.9
59.3
45.5
33.7
29.3
210 200 190 180 170 160 150 140 130 120 110 100 90 80 70 60 50 40 30 20 10 0 -10
f1 (ppm)

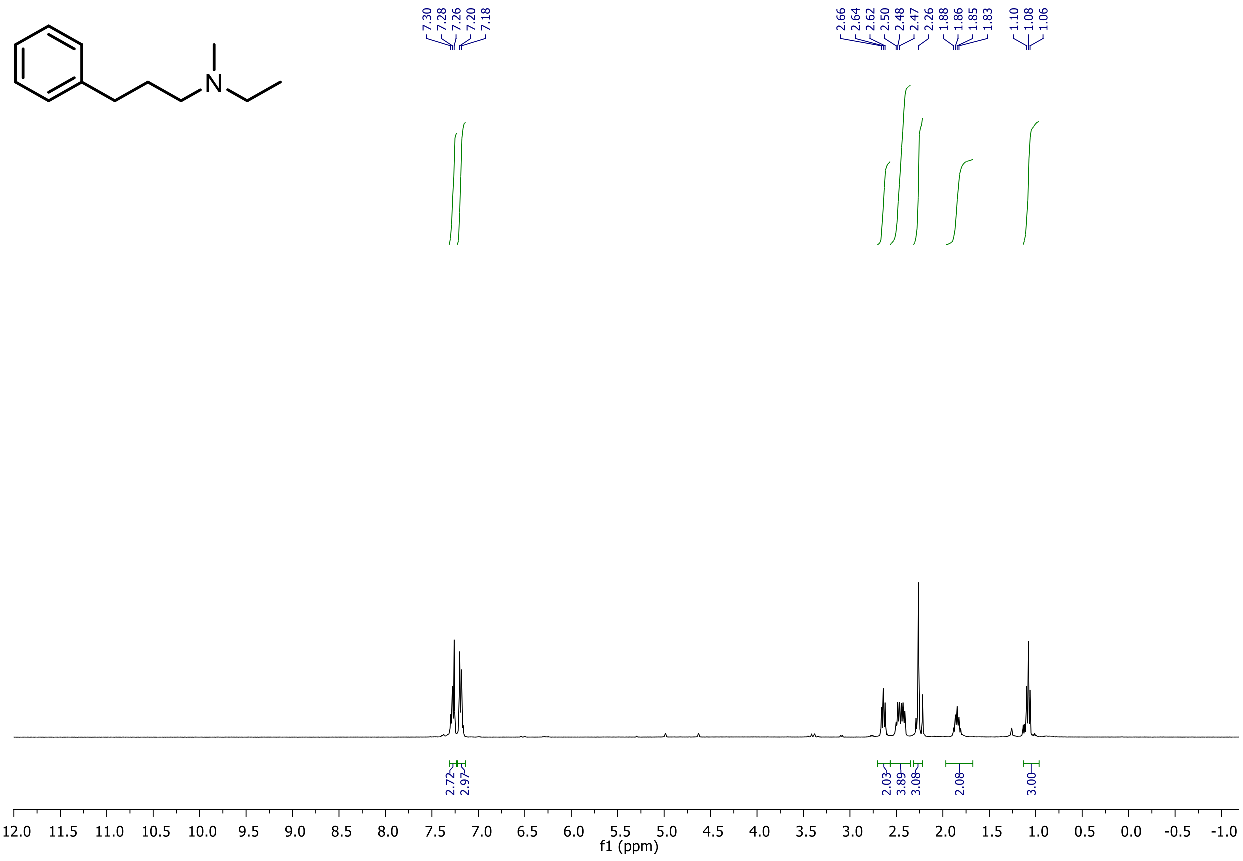
7.30
7.28
7.26
7.20
7.18
2.66
2.64
2.62
2.50
2.48
2.47
2.26
1.88
1.86
1.85
1.83
1.10
1.08
1.06
2.72
2.97
2.03
3.89
3.08
2.08
3.00
12.0 11.5 11.0 10.5 10.0 9.5 9.0 8.5 8.0 7.5 7.0 6.5 6.0 5.5 5.0 4.5 4.0 3.5 3.0 2.5 2.0 1.5 1.0 0.5 0.0 -0.5 -1.0
f1 (ppm)

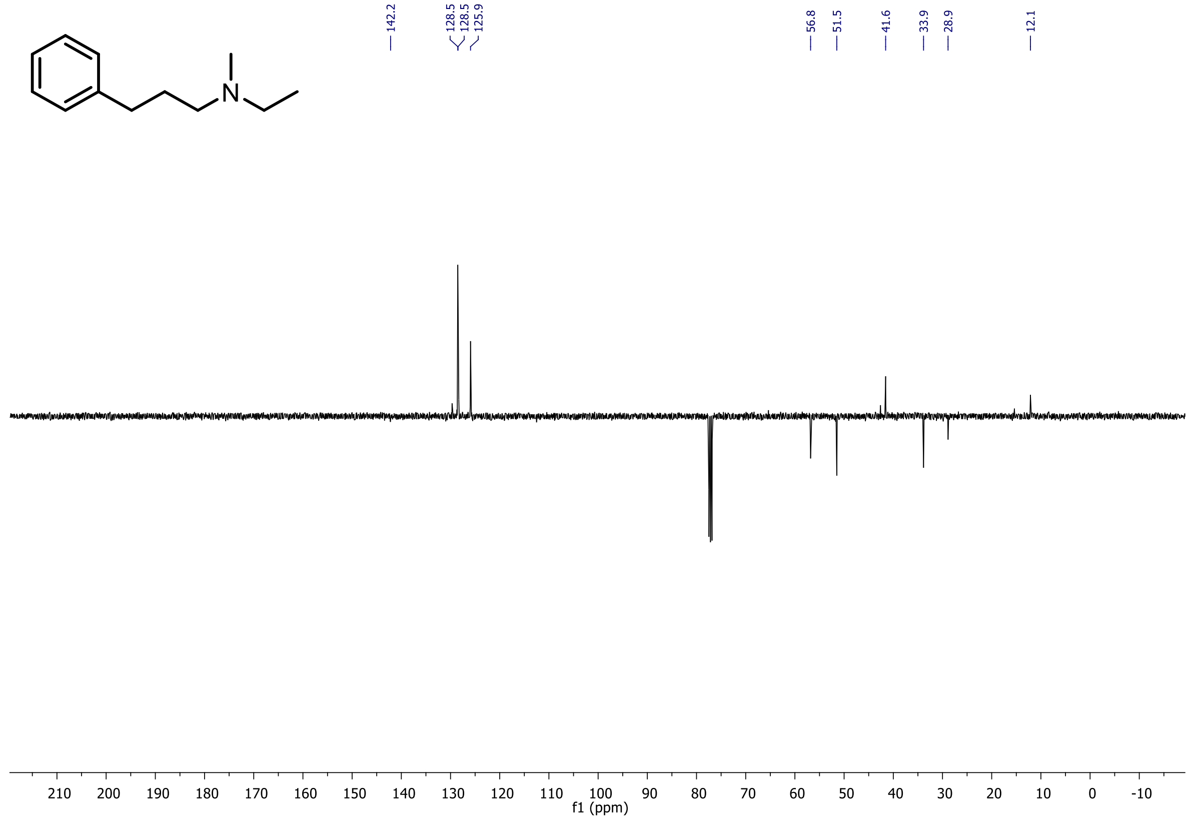
142.2
128.5
128.5
125.9
56.8
51.5
41.6
33.9
28.9
12.1
210 200 190 180 170 160 150 140 130 120 110 100 90 80 70 60 50 40 30 20 10 0 -10
f1 (ppm)

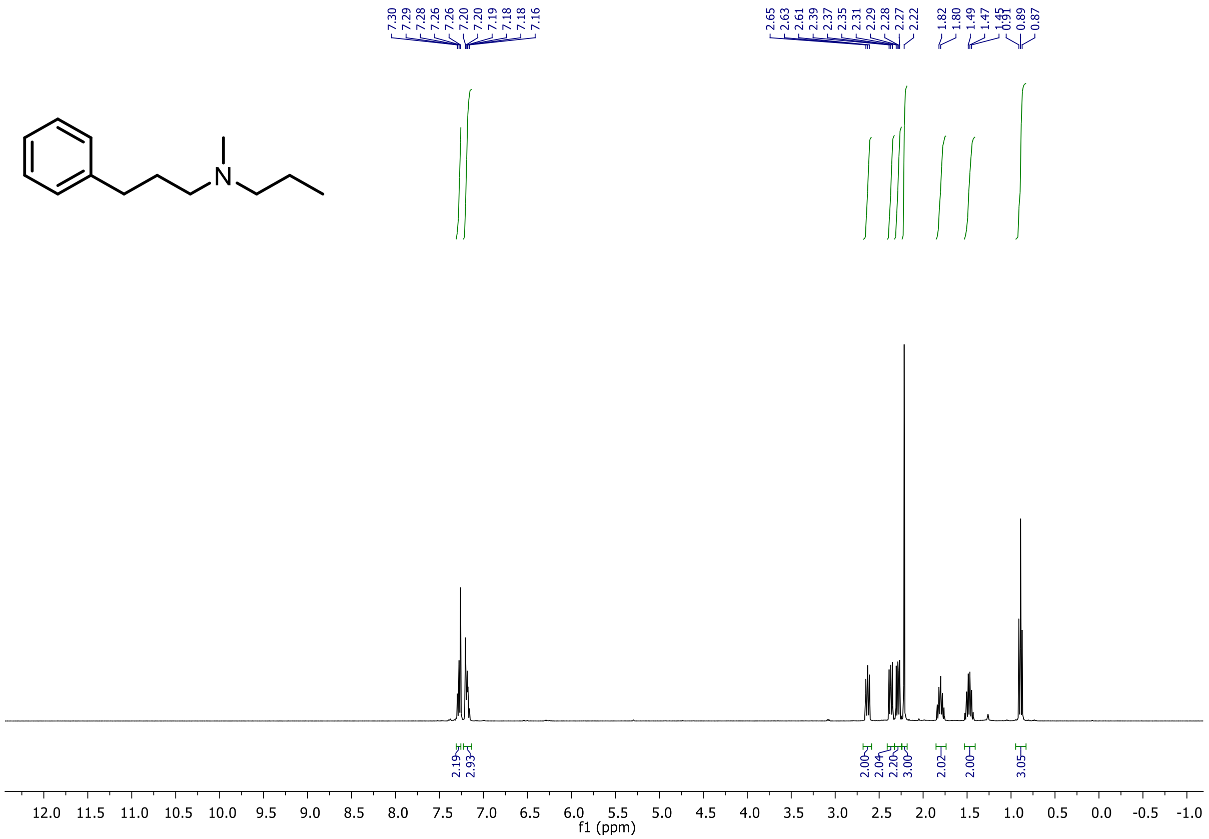

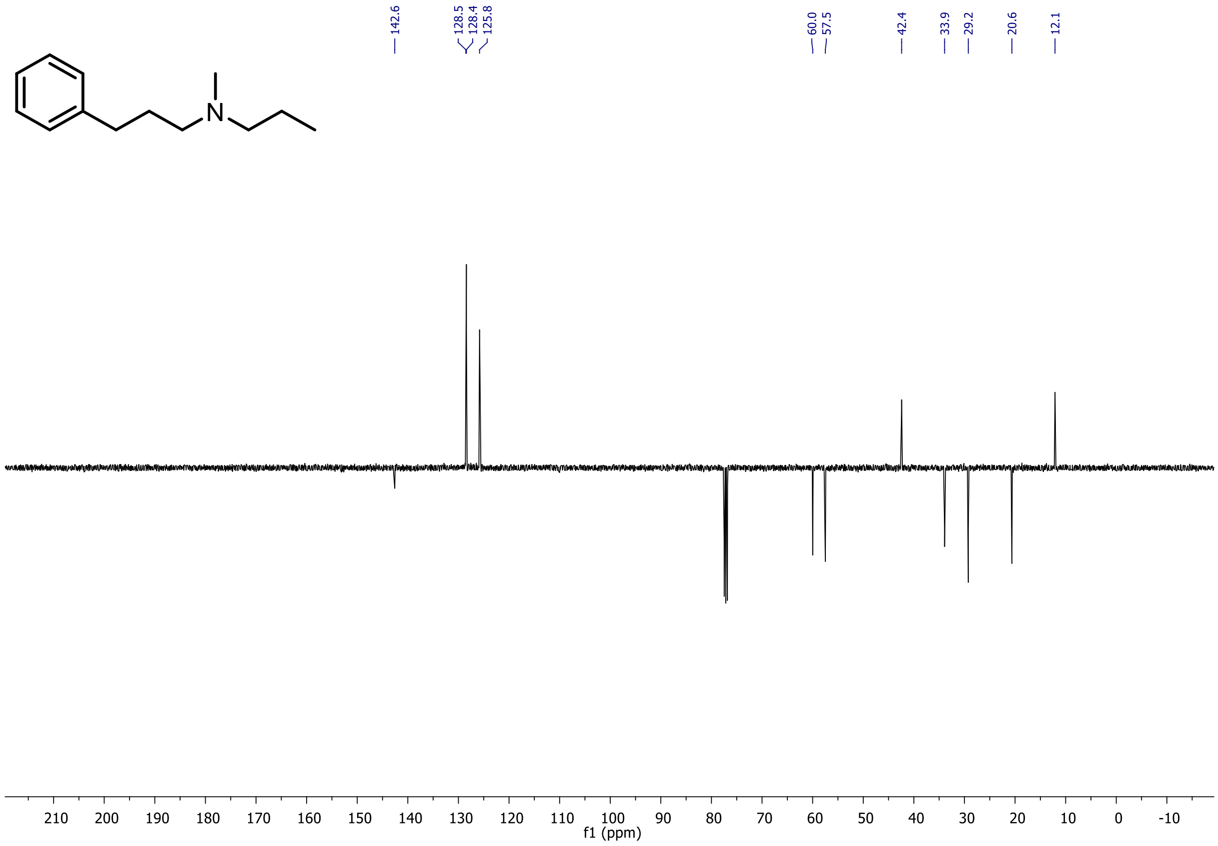

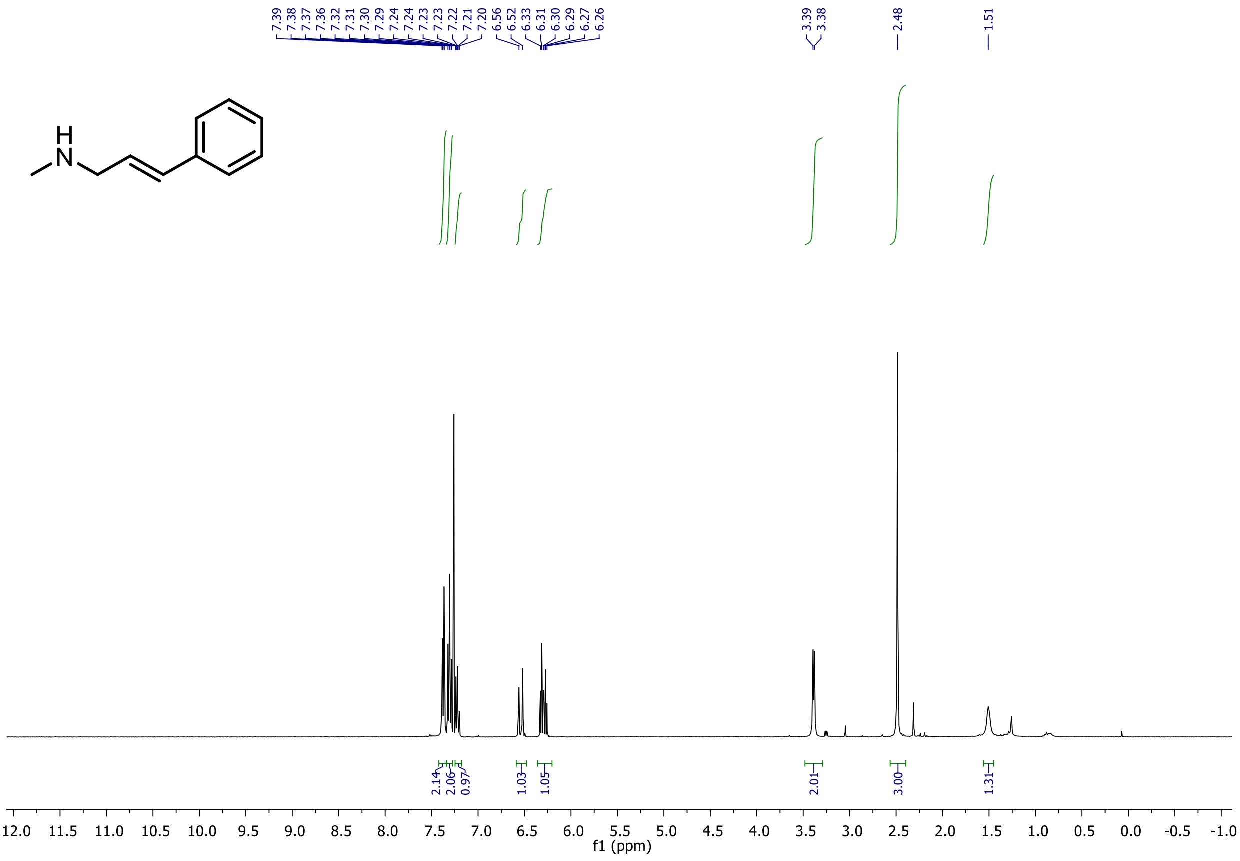

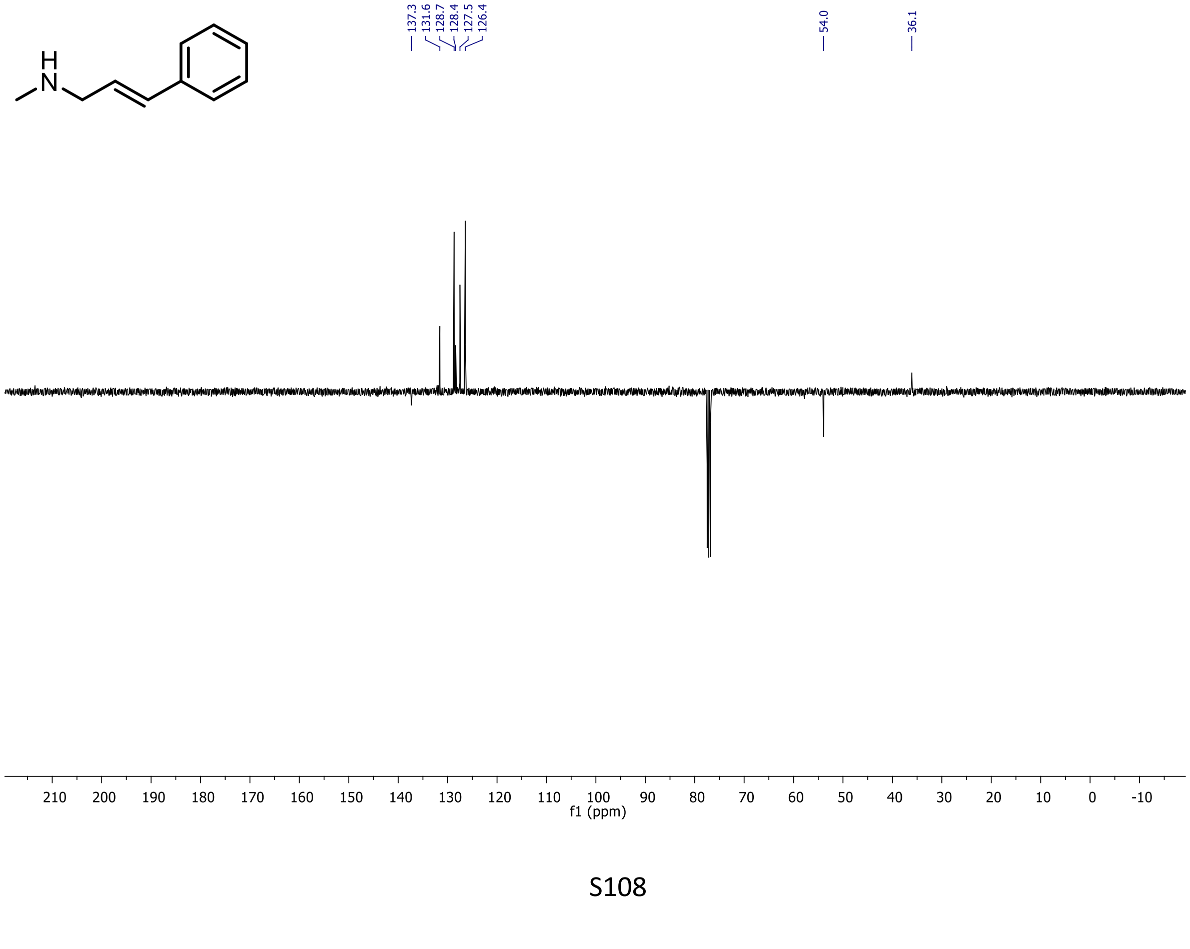

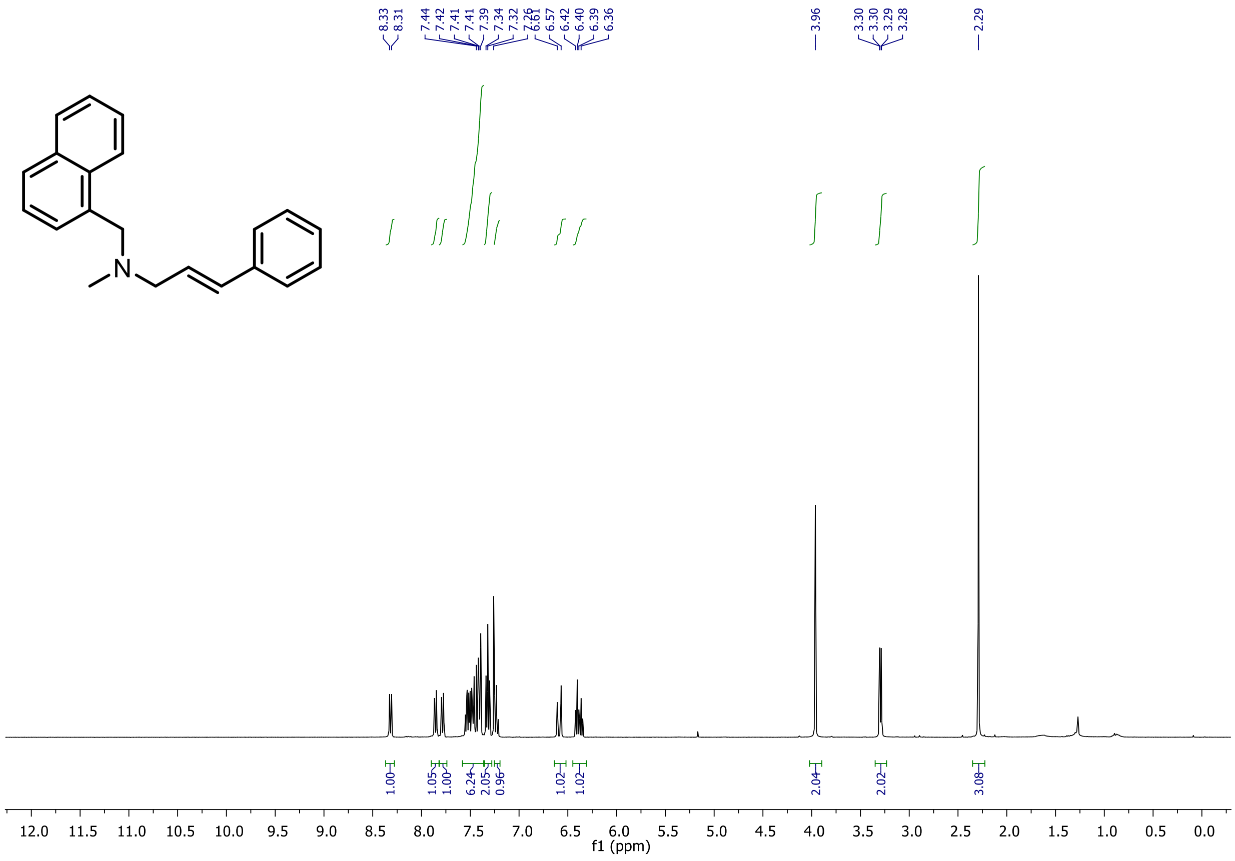

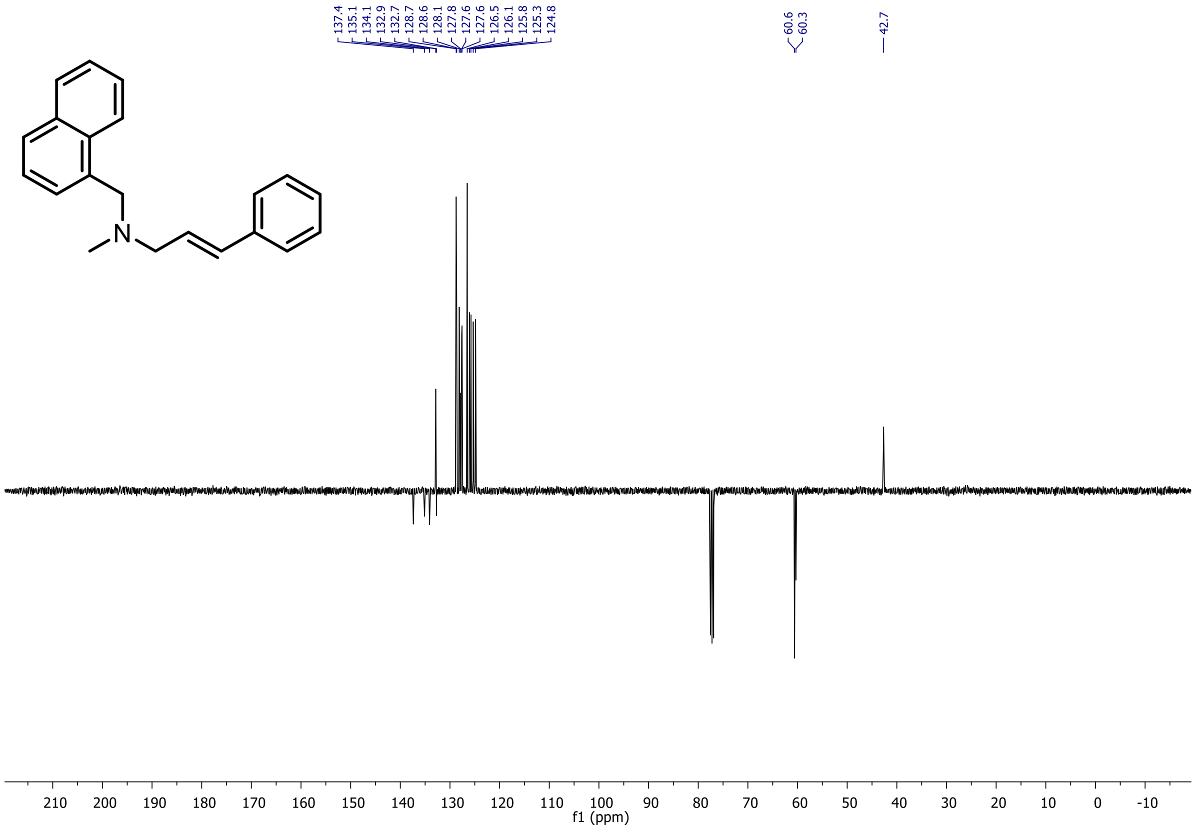

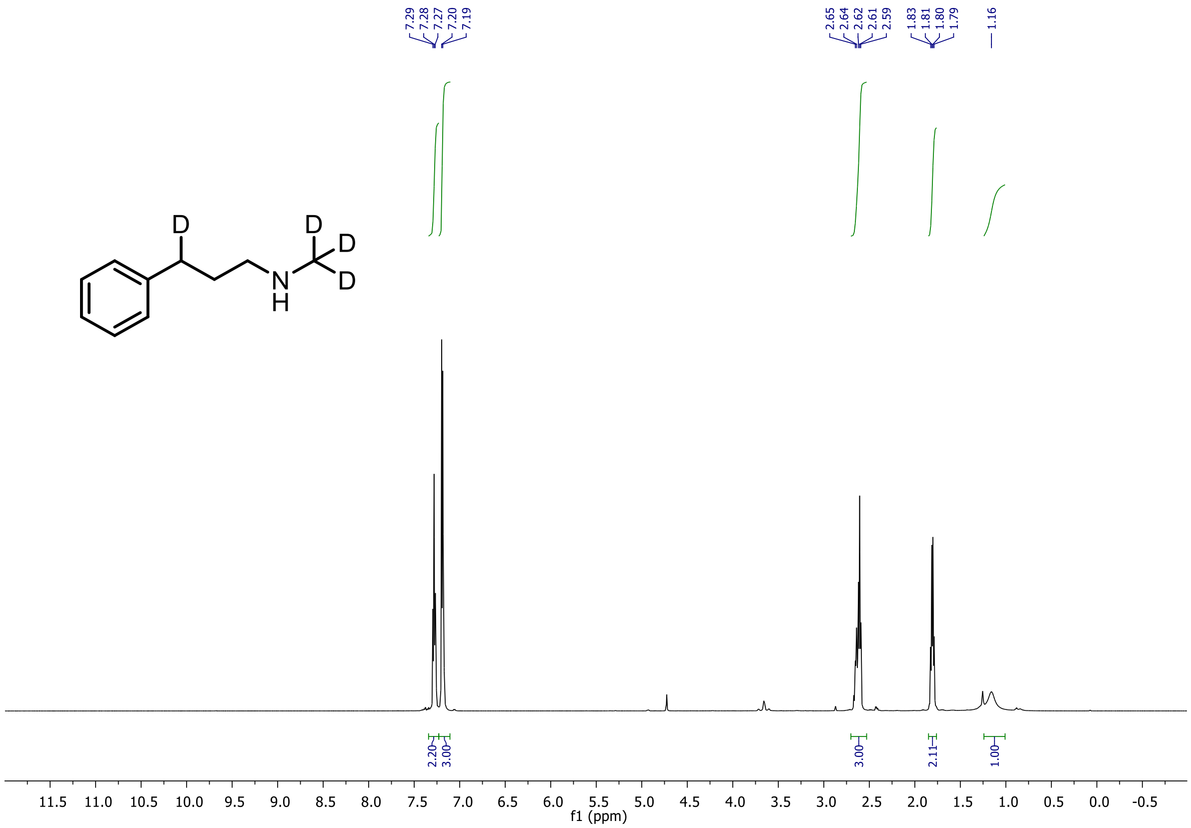
7.29
7.28
7.27
7.20
7.19
2.65
2.64
2.62
2.61
2.59
1.83
1.81
1.80
1.79
1.16
2.20
3.00
3.00
2.11
1.00
f1 (ppm)

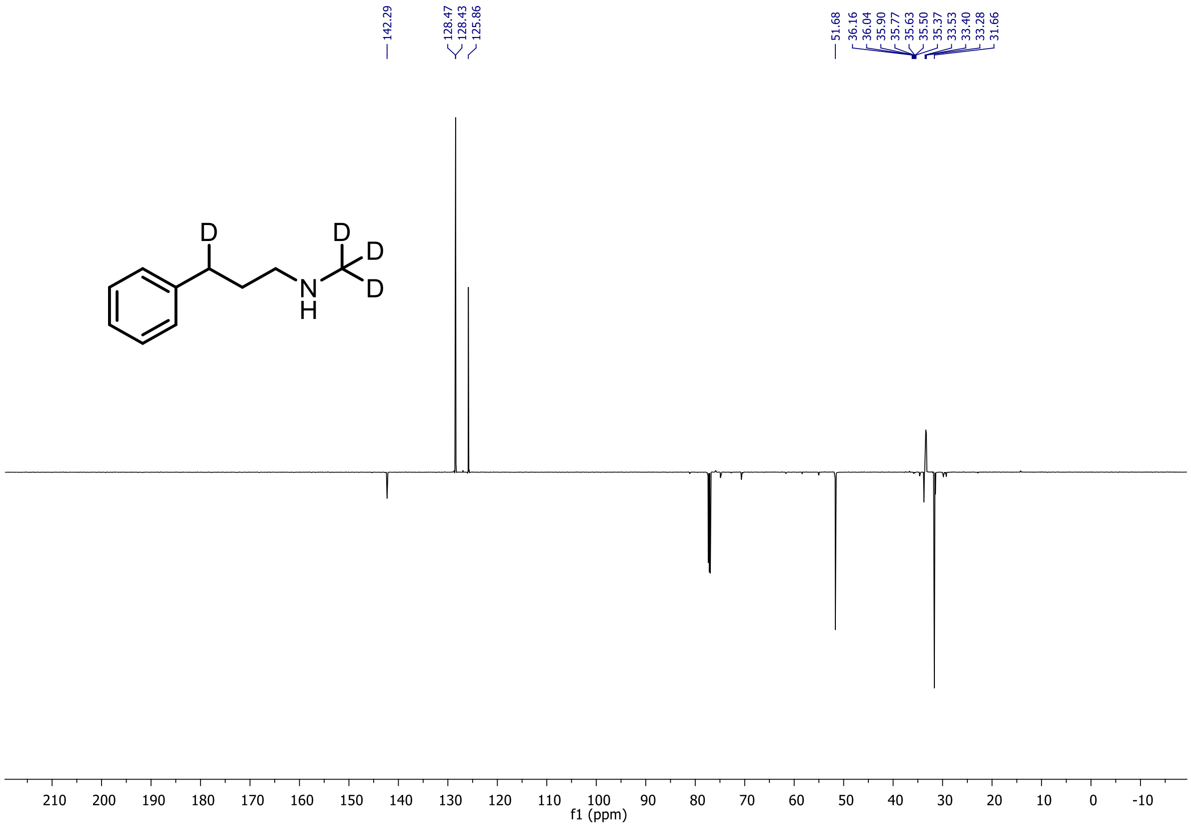
142.29
128.47
128.43
125.86
51.68
36.16
36.04
35.90
35.77
35.63
35.50
35.37
33.53
33.40
33.28
31.66
f1 (ppm)

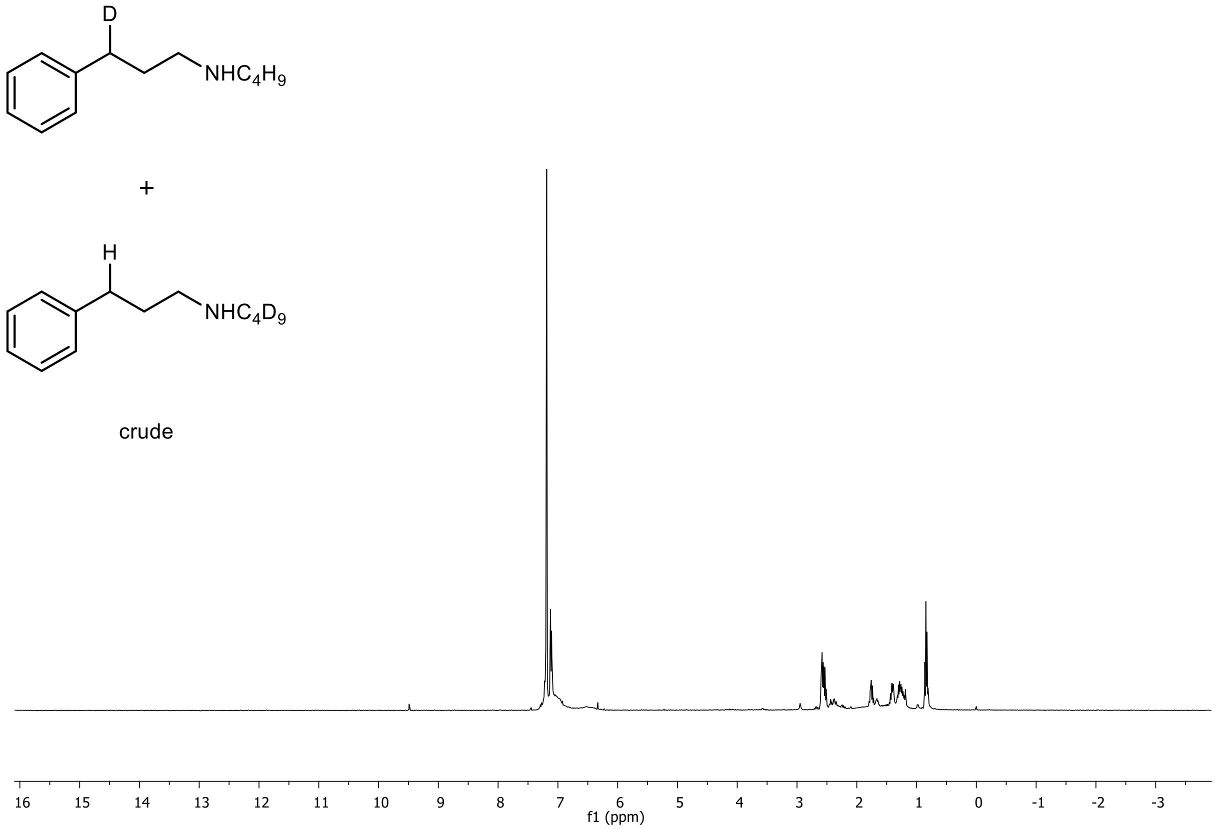
D
NHC_4H_9
+
H
NHC_4D_9
crude
16 15 14 13 12 11 10 9 8 7 6 5 4 3 2 1 0 -1 -2 -3
f1 (ppm)

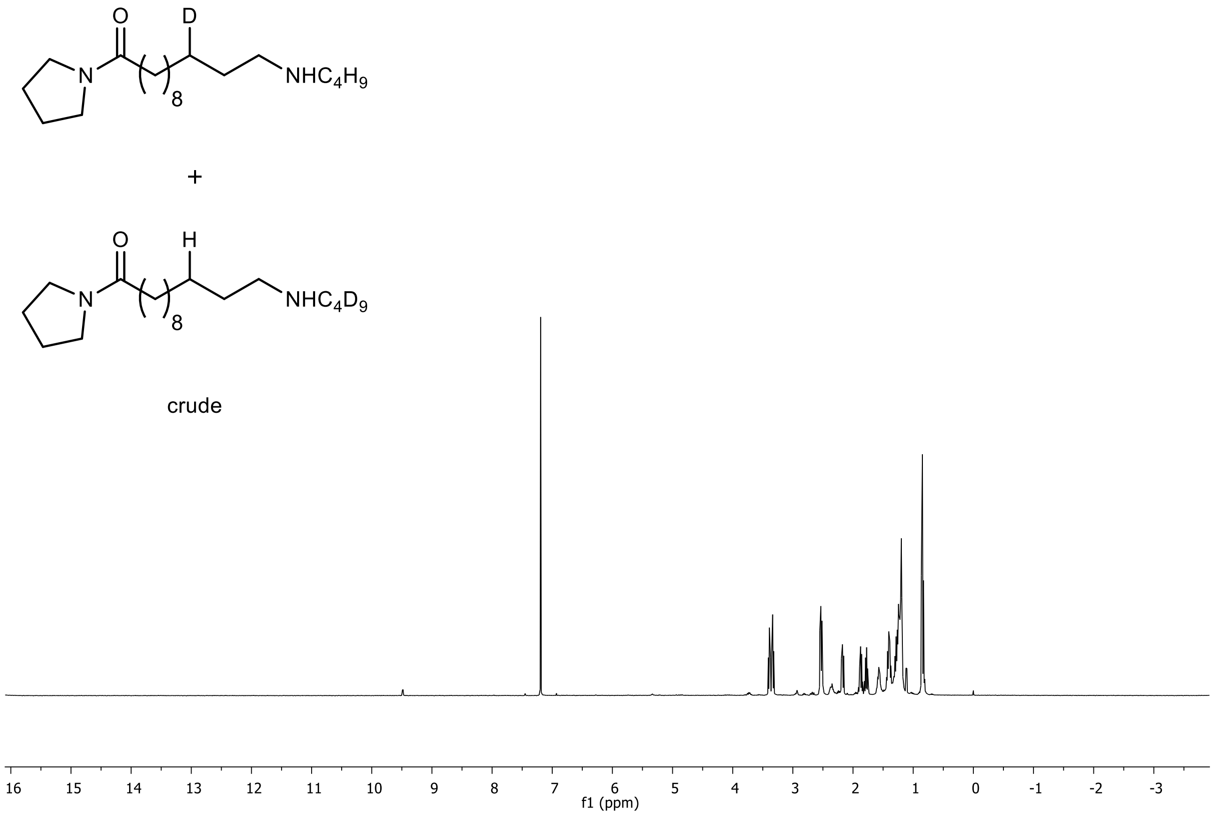
O
D
N
8
NHC_4H_9
+
O
H
N
8
NHC_4D_9
crude
16 15 14 13 12 11 10 9 8 7 6 5 4 3 2 1 0 -1 -2 -3
f1 (ppm)

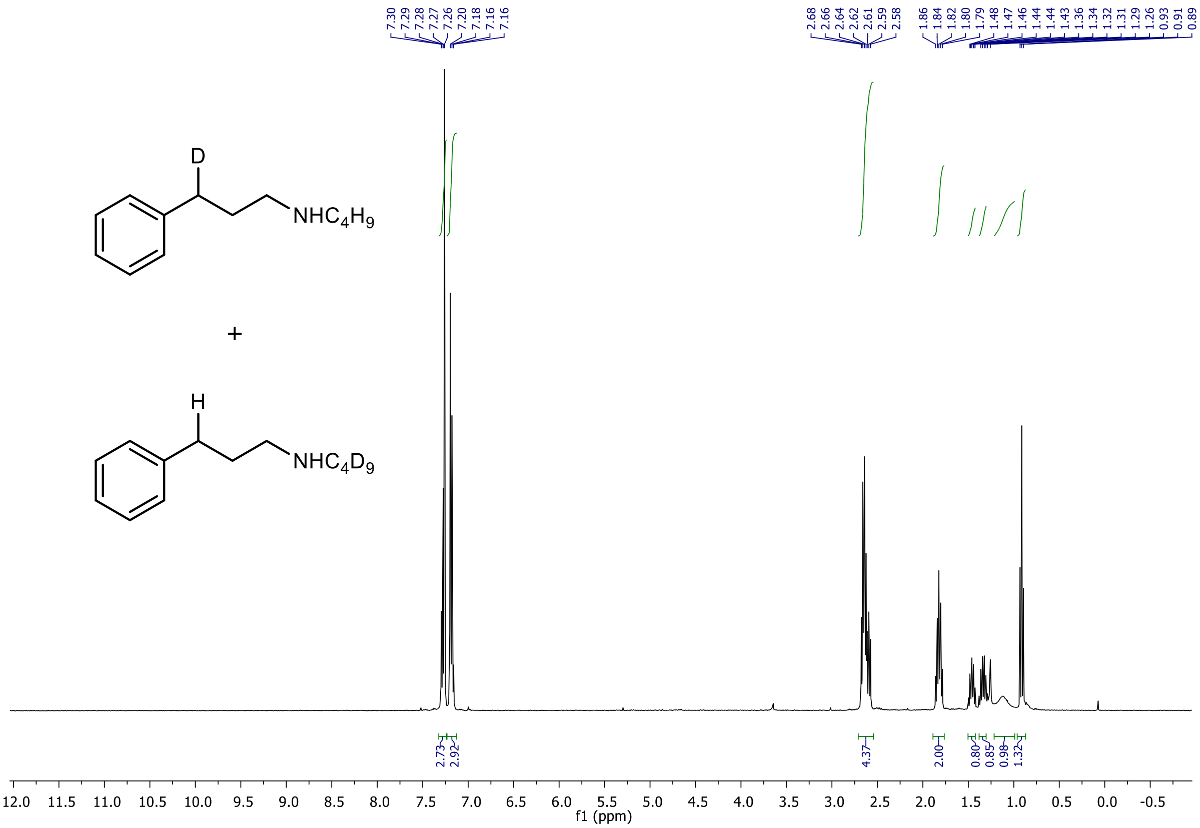

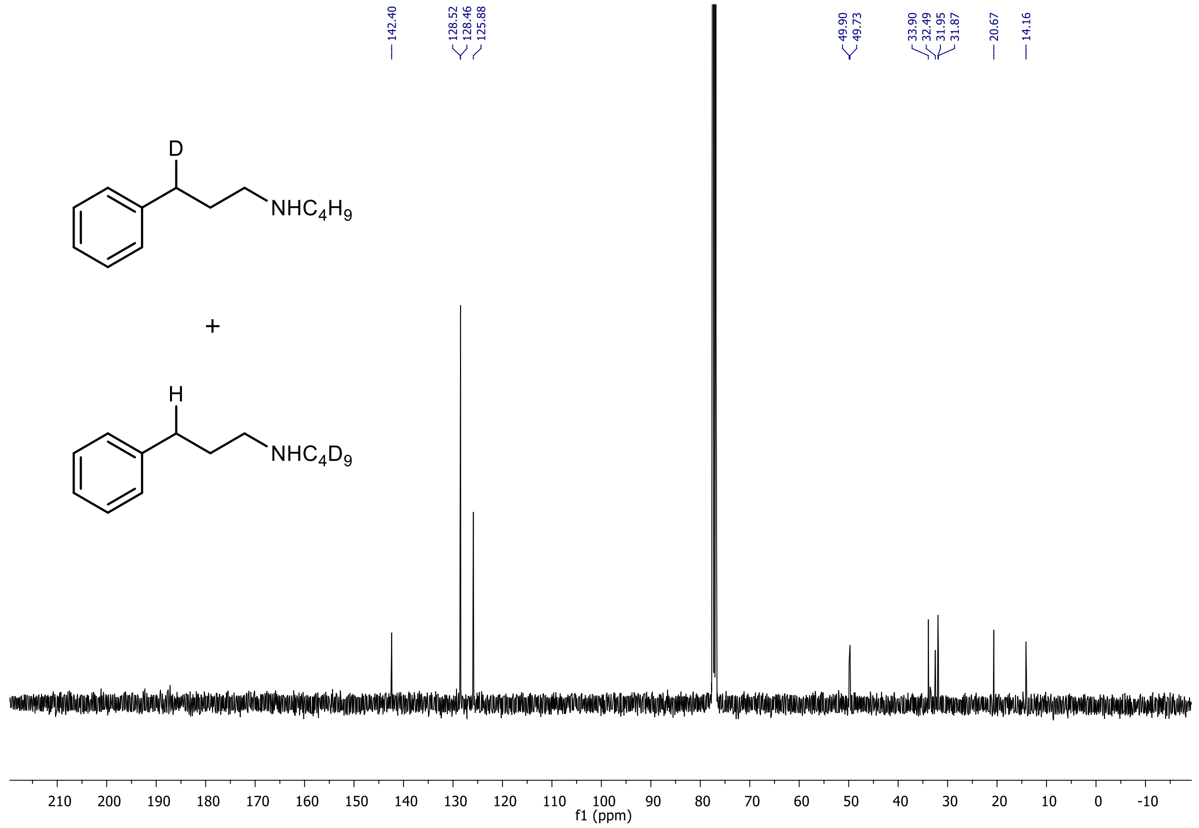

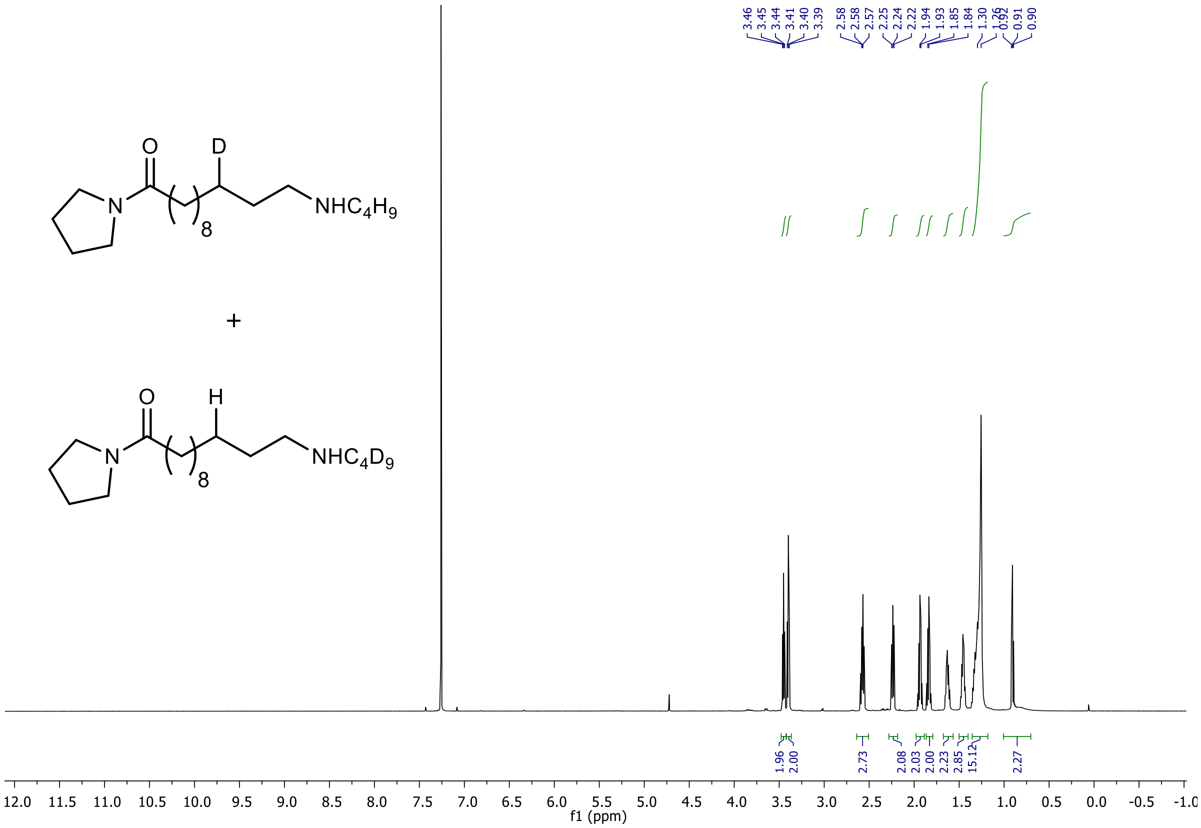

3.46
3.45
3.44
3.41
3.40
3.39
2.58
2.58
2.57
2.25
2.24
2.22
1.94
1.93
1.85
1.84
1.30
0.91
0.90
O
D
N
NHC4H9
8
+
O
H
N
NHC4D9
8
1.96
2.00
2.73
2.08
2.03
2.00
2.23
2.85
15.12
2.27
12.0 11.5 11.0 10.5 10.0 9.5 9.0 8.5 8.0 7.5 7.0 6.5 6.0 5.5 5.0 4.5 4.0 3.5 3.0 2.5 2.0 1.5 1.0 0.5 0.0 -0.5 -1.0
f1 (ppm)

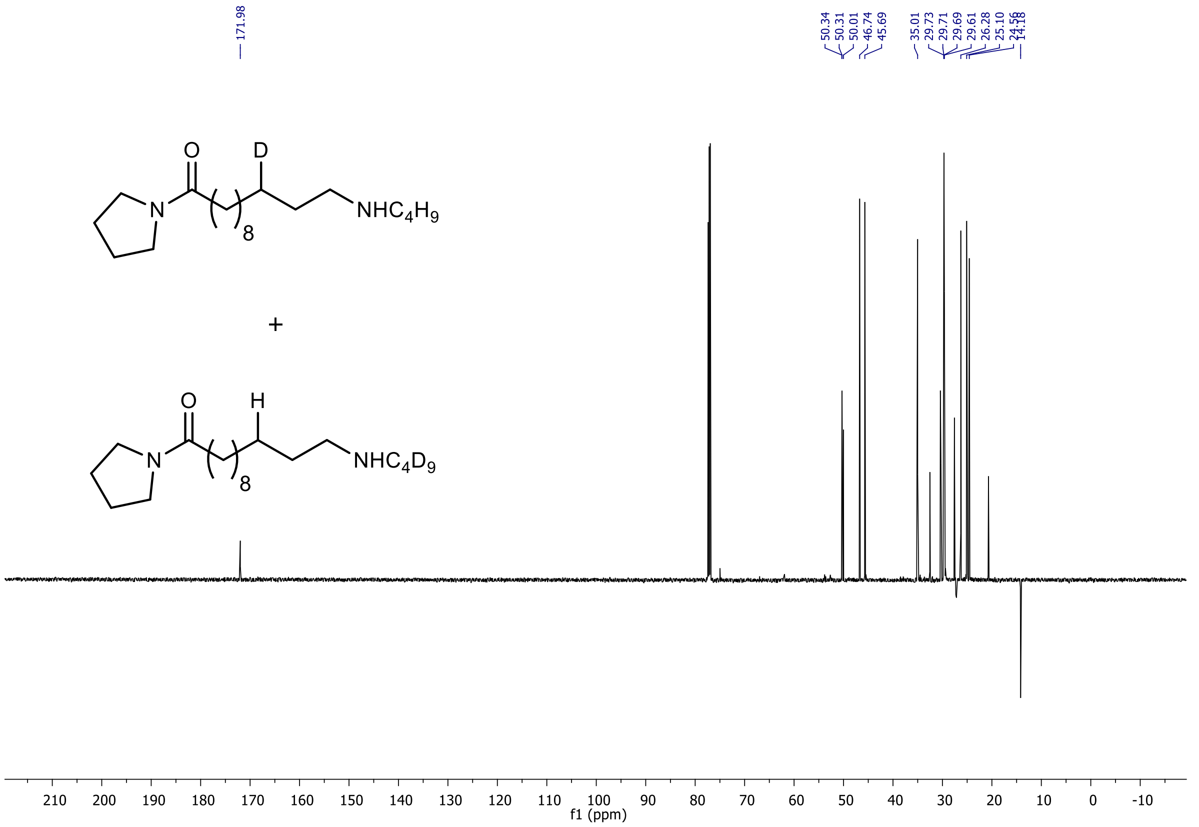

171.98
50.34
50.31
50.01
46.74
45.69
35.01
29.73
29.71
29.69
29.61
26.28
25.10
14.18
O
D
N
NHC4H9
8
+
O
H
N
NHC4D9
8
210 200 190 180 170 160 150 140 130 120 110 100 90 80 70 60 50 40 30 20 10 0 -10
f1 (ppm)

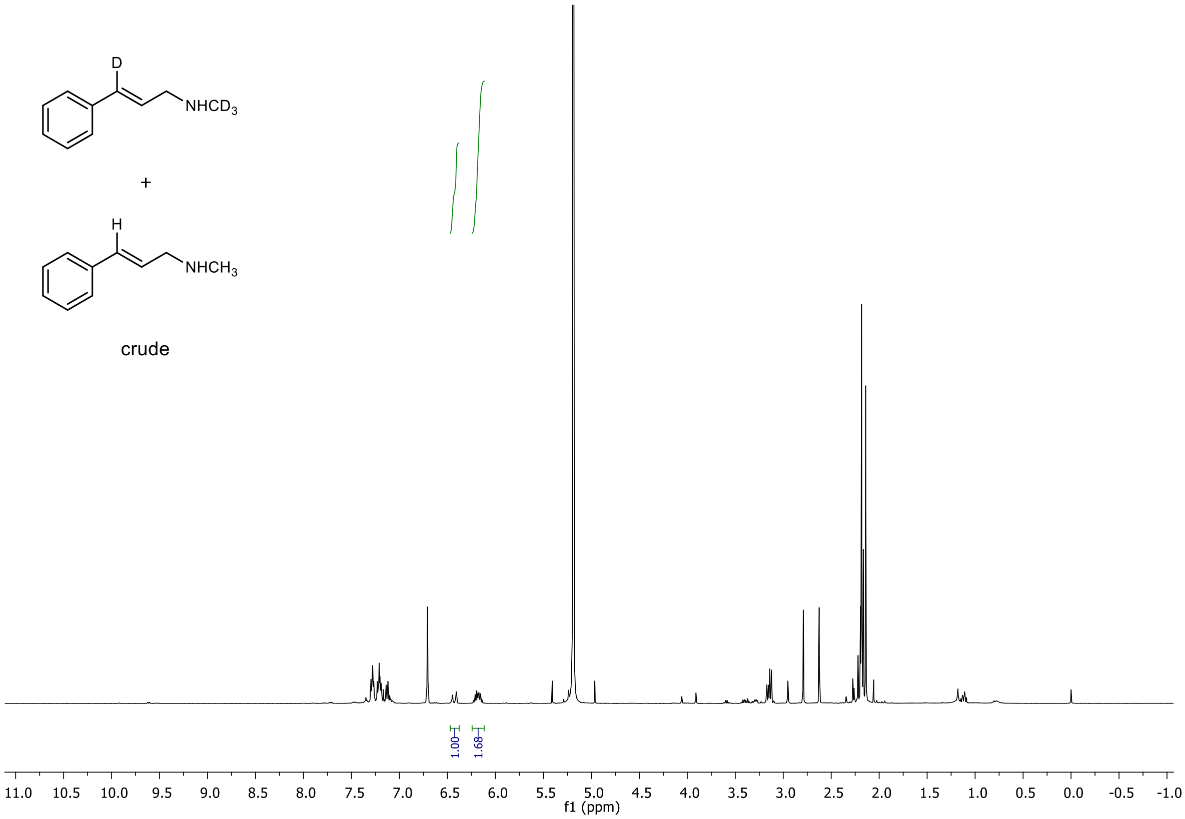
D
NHCD$_3$
+
H
NHCH$_3$
crude
1.00
1.68
11.0 10.5 10.0 9.5 9.0 8.5 8.0 7.5 7.0 6.5 6.0 5.5 5.0 4.5 4.0 3.5 3.0 2.5 2.0 1.5 1.0 0.5 0.0 -0.5 -1.0
f1 (ppm)

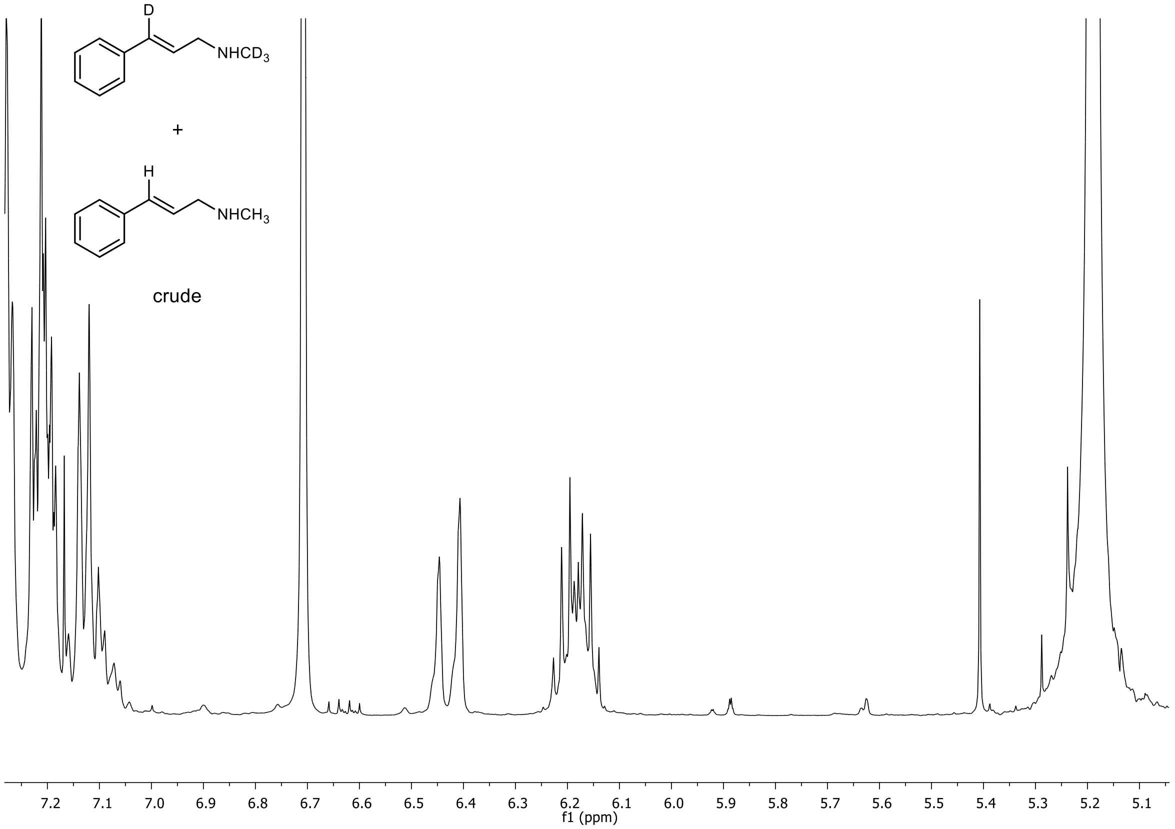
D
NHCD$_3$
+
H
NHCH$_3$
crude
7.2 7.1 7.0 6.9 6.8 6.7 6.6 6.5 6.4 6.3 6.2 6.1 6.0 5.9 5.8 5.7 5.6 5.5 5.4 5.3 5.2 5.1
f1 (ppm)

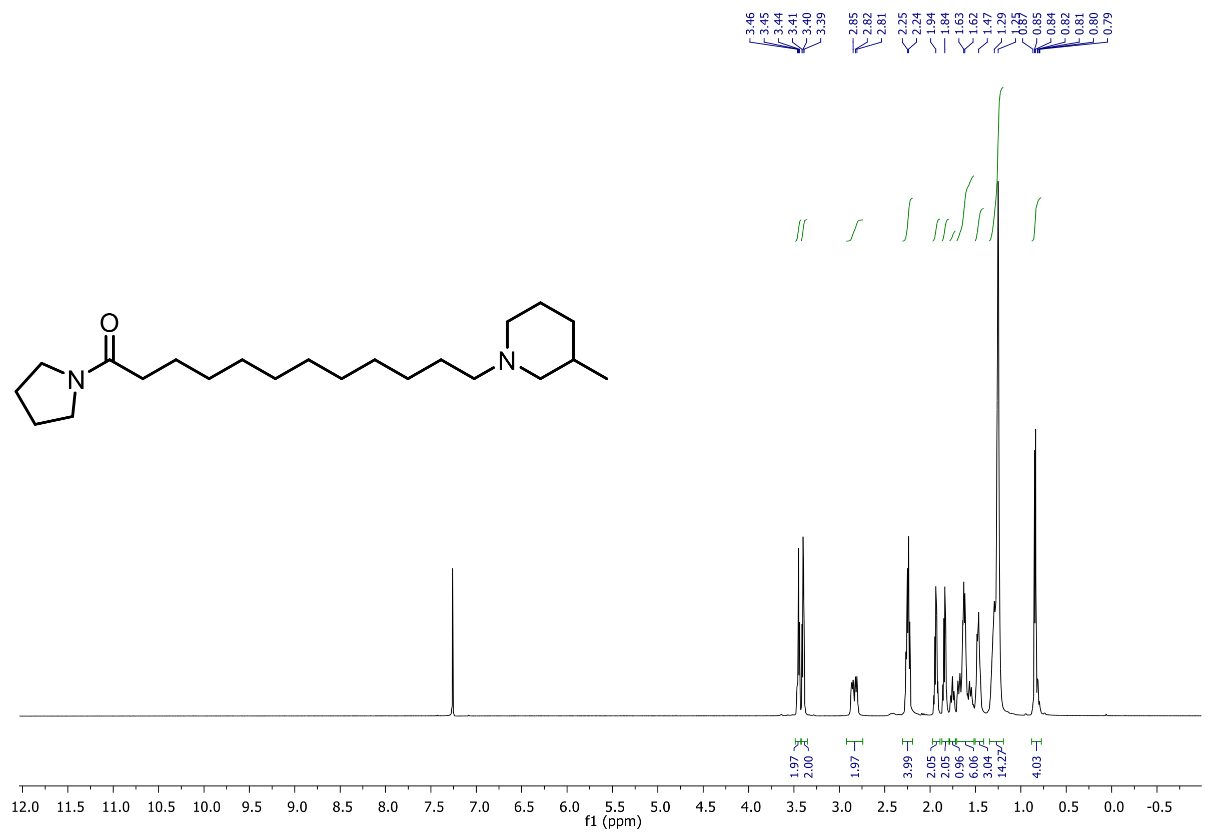

3.46
3.45
3.44
3.41
3.40
3.39
2.85
2.82
2.81
2.25
2.24
1.94
1.84
1.63
1.62
1.47
1.29
1.25
0.87
0.85
0.84
0.82
0.81
0.80
0.79
1.97
2.00
1.97
3.99
2.05
2.05
0.96
6.06
3.04
14.27
4.03
O
N
N
f1 (ppm)

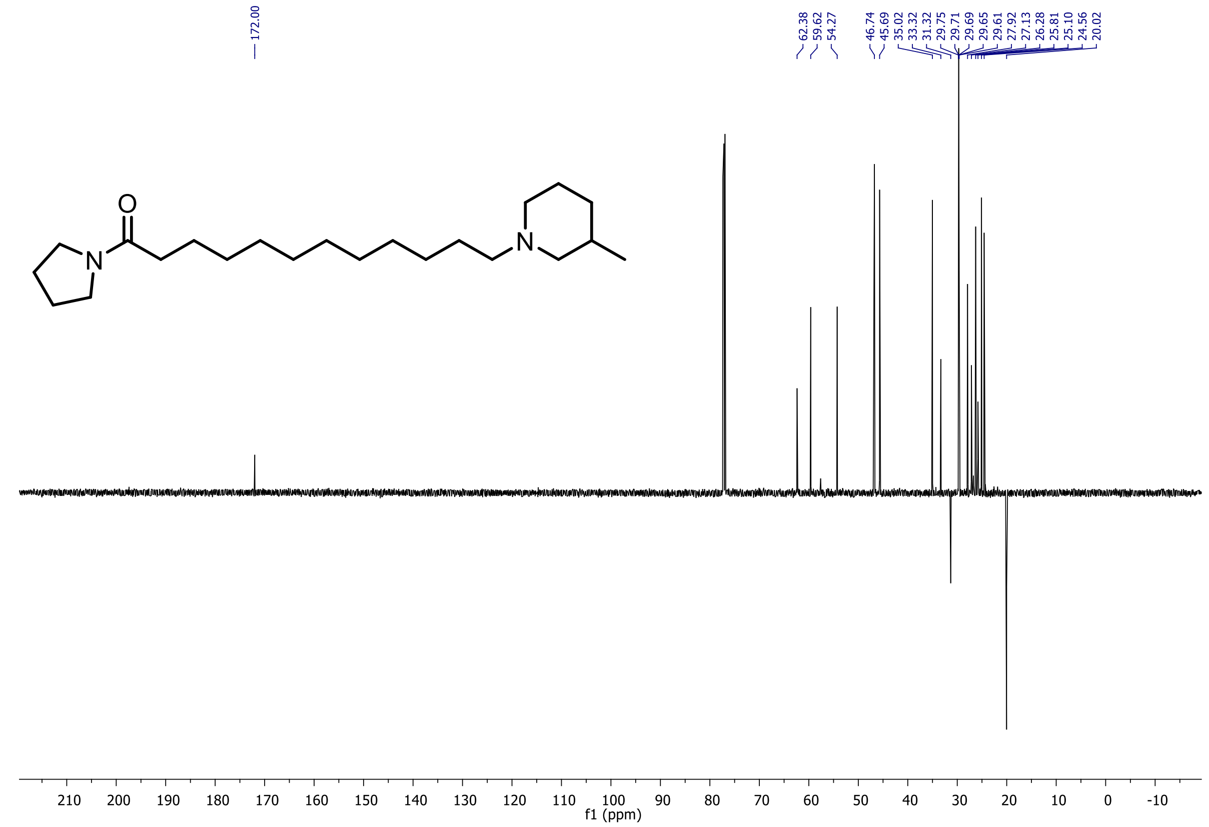

172.00
62.38
59.62
54.27
46.74
45.69
35.02
33.32
31.32
29.75
29.71
29.69
29.65
29.61
27.92
27.13
26.28
25.81
25.10
24.56
20.02
O
N
N
f1 (ppm)

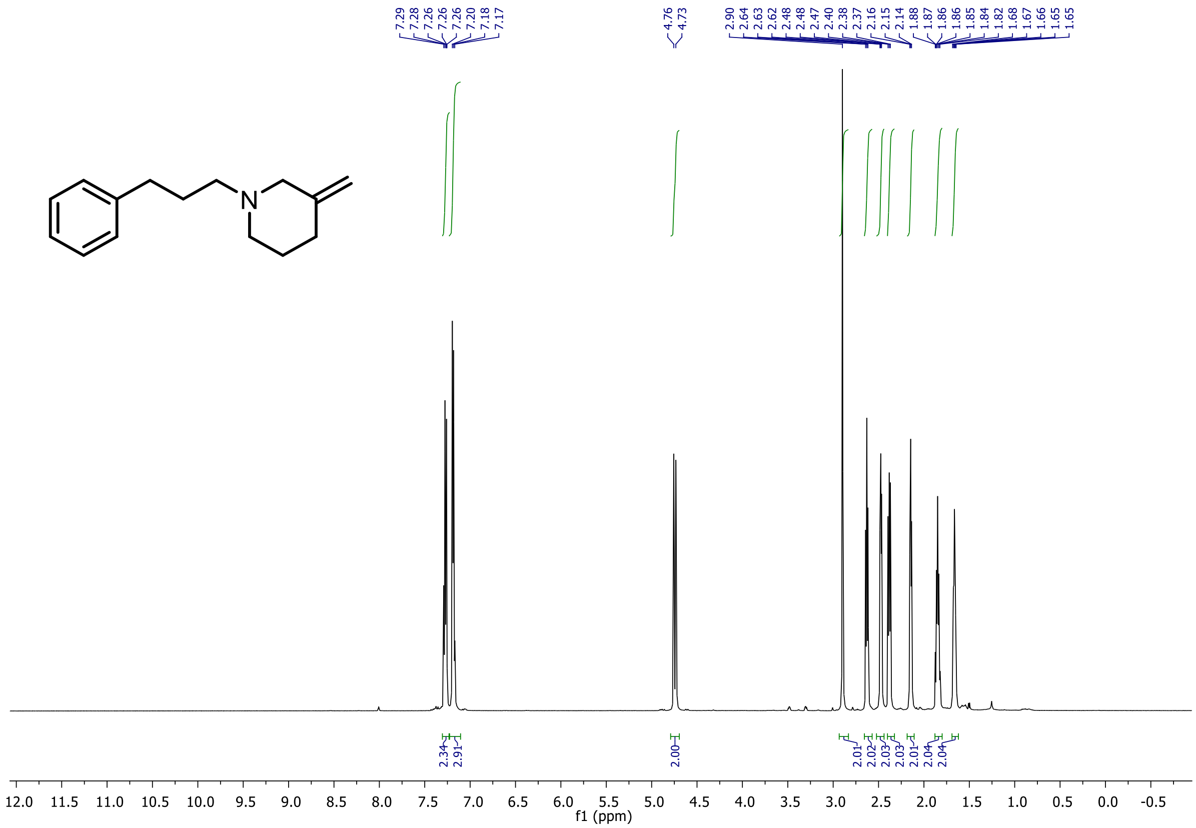
7.29
7.28
7.26
7.26
7.26
7.20
7.18
7.17
4.76
4.73
2.90
2.64
2.63
2.62
2.48
2.48
2.47
2.40
2.38
2.37
2.16
2.15
2.14
1.88
1.87
1.86
1.86
1.85
1.84
1.82
1.68
1.67
1.66
1.65
1.65
N
2.34
2.91
2.00
2.01
2.02
2.03
2.03
2.01
2.04
2.04
12.0 11.5 11.0 10.5 10.0 9.5 9.0 8.5 8.0 7.5 7.0 6.5 6.0 5.5 5.0 4.5 4.0 3.5 3.0 2.5 2.0 1.5 1.0 0.5 0.0 -0.5
f1 (ppm)

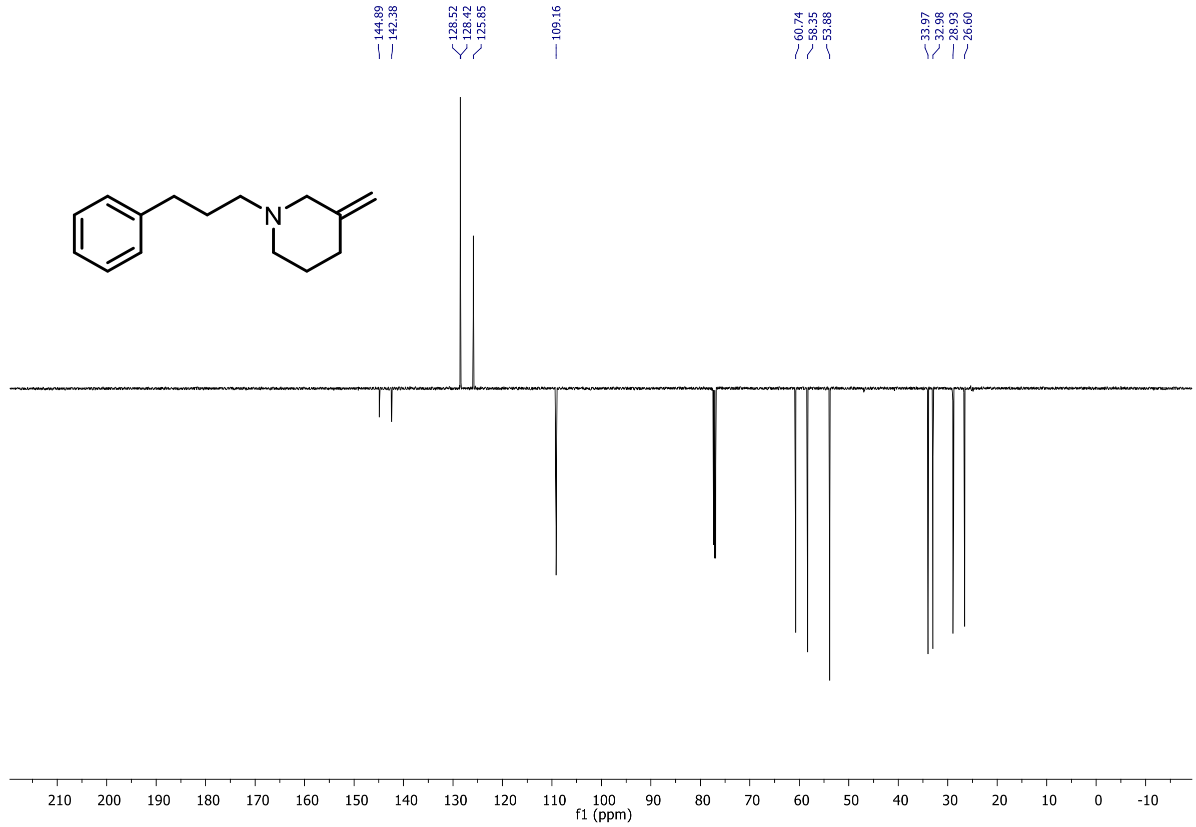
144.89
142.38
128.52
128.42
125.85
109.16
60.74
58.35
53.88
33.97
32.98
28.93
26.60
N
210 200 190 180 170 160 150 140 130 120 110 100 90 80 70 60 50 40 30 20 10 0 -10
f1 (ppm)

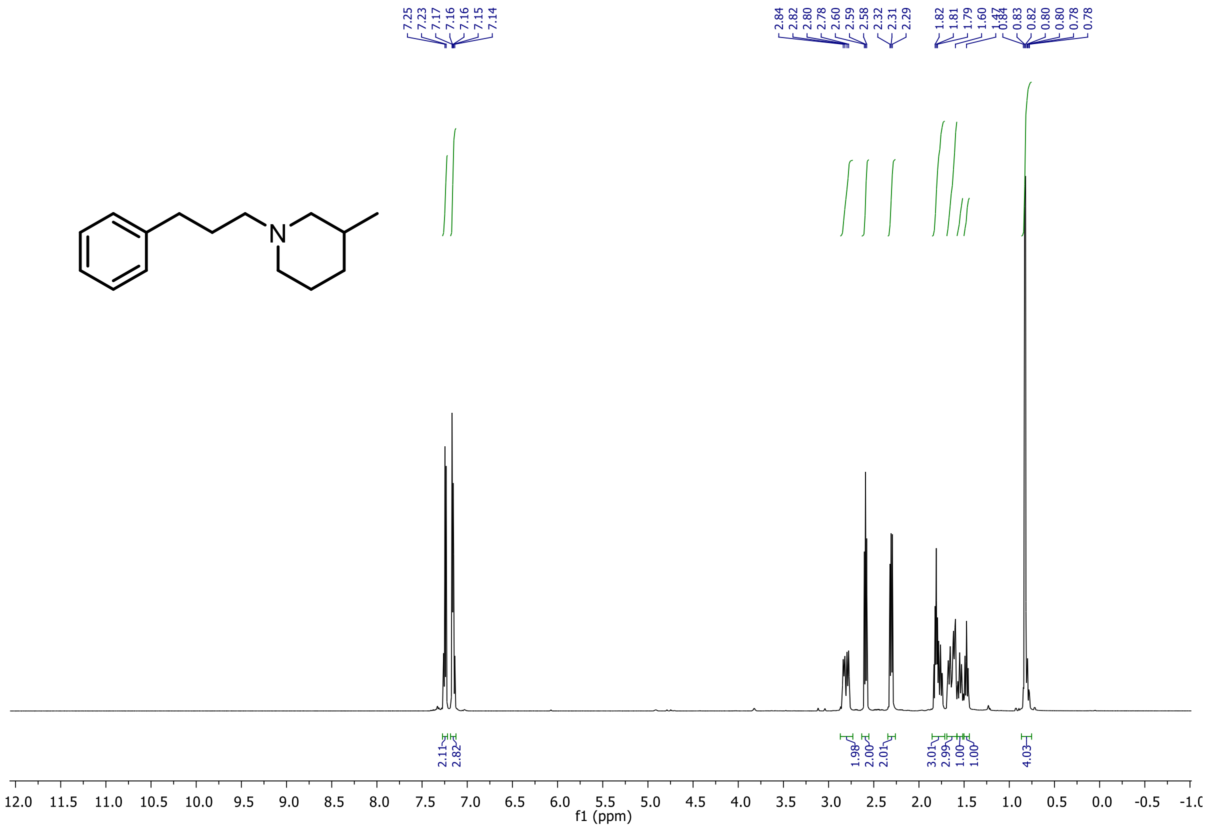
7.25
7.23
7.17
7.16
7.16
7.15
7.14
2.84
2.82
2.80
2.78
2.60
2.59
2.58
2.32
2.31
2.29
1.82
1.81
1.79
1.60
1.47
0.84
0.83
0.82
0.80
0.80
0.78
0.78
N
2.11
2.82
1.98
2.00
2.01
3.01
2.99
1.00
1.00
4.03
f1 (ppm)

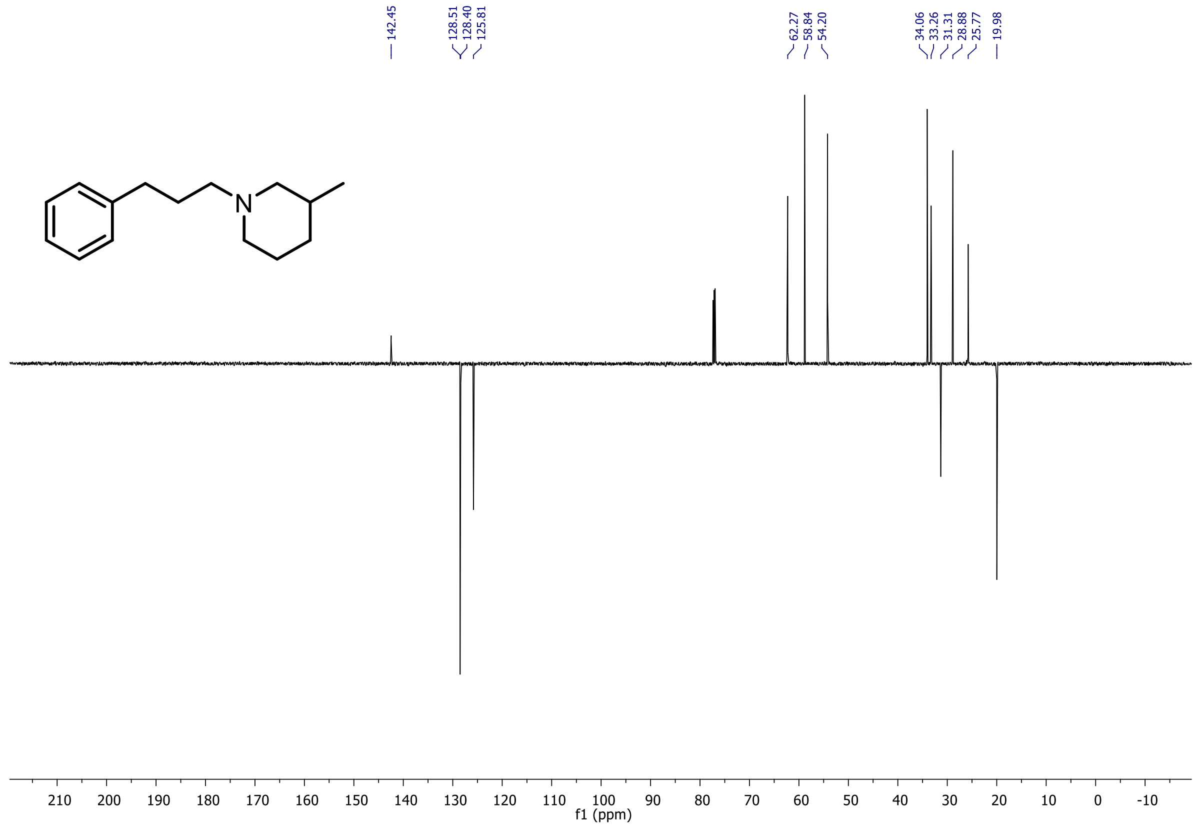
142.45
128.51
128.40
125.81
62.27
58.84
54.20
34.06
33.26
31.31
28.88
25.77
19.98
N
f1 (ppm)

4 References

[1] Porzelle, A. & Williams, C. M. Direct Access to Functionalized Cyclic Enones Using Mannich, Morita-Baylis-Hillman and Elimination Reactions. *Synthesis* **18**, 3025–3030 (2006).

[2] Jensen, N. Tryptamines as Ligands and Modulators of the Serotonin 5-HT2A Receptor and the Isolation of Aeruginascin from the Hallucinogenic Mushroom Inocybe aeruginascens Göttingen, 2004– https://d-nb.info/973960833/34

[3] Zhang, F., Das, S., Walkinshaw, A. J., Casitas, A., Taylor, M., Suero, M. G. & Gaunt, M. J. Cu-Catalyzed Cascades to Carbocycles: Union of Diaryliodonium Salts with Alkenes or Alkynes Exploiting Remote Carbocations. *J. Am. Chem. Soc*. **136**, 8851–8854 (2014).

[4] Ramin, M. A., Le Bourdon, G., Heuzé, K., Degueil, M., Buffeteau, T., Bennetau, B. & Vellutini, L. Epoxy-Terminated Self-Assembled Monolayers Containing Internal Urea or Amide Groups. *Langmuir* **31**, 2783–2789 (2015).

[5] Beauchamp, L. M., Tuttle, J. V., Rodriguez, M. E. & Sznaidman, M. L. Guanine, Pyrazolo[3,4-d]pyrimidine, and Triazolo[4,5-d]pyrimidine (8-Azaguanine) Phosphonate Acyclic Derivatives as Inhibitors of Purine Nucleoside Phosphorylase. *J. Med. Chem*. **39**, 949–956 (1996).

[6] Imai, M., Tanaka, M., Nagumo, S., Kawahara, N. & Suemune, H. Nitrile-Promoted Rh-Catalyzed Intermolecular Hydroacylation of Olefins with Salicylaldehyde. *J. Org. Chem.* **72**, 2543–2546 (2007).

[7] Bergmann, D. J., Campi, E. M., Roy Jackson, W., Patti A. F. & Saylik D. Synthesis of Medium and Large Cyclic Amines in Rhodium-Catalysed Reactions of Aminoalkenes with H2/CO. *Australian J. Chem.* **53**, 835–844 (2000).
